# Shifts in rhizosphere microbial communities in *Oplopanax elatus* Nakai are related to soil chemical properties under different growth conditions

**DOI:** 10.1038/s41598-022-15340-1

**Published:** 2022-07-07

**Authors:** Wanying Li, Xiujuan Lei, Rui Zhang, Qingjun Cao, He Yang, Nanqi Zhang, Shuangli Liu, Yingping Wang

**Affiliations:** 1 College of Chinese Medicinal Materials, Jilin Agriculture University, Changchun, 130118 People’s Republic of China; 2 Jilin Academy of Agriculture Science, Changchun, 130033 People’s Republic of China; 3 National and Local Joint Engineering Research Center for Ginseng Breeding and Development, Changchun, 130118 People’s Republic of China

**Keywords:** Plant sciences, Plant ecology, Plant physiology, High-throughput screening, Next-generation sequencing, RNA sequencing

## Abstract

Plant growth environment plays an important role in shaping soil microbial communities. To understand the response of soil rhizosphere microbial communities in *Oplopanax elatus* Nakai plant to a changed growth conditions from natural habitation to cultivation after transplant. Here, a comparative study of soil chemical properties and microbial community using high-throughput sequencing was conducted under cultivated conditions (CT) and natural conditions (WT), in Changbai Mountain, Northeast of China. The results showed that rhizosphere soil in CT had higher pH and lower content of soil organic matter (SOM) and available nitrogen compared to WT. These changes influenced rhizosphere soil microbial communities, resulting in higher soil bacterial and fungi richness and diversity in CT soil, and increased the relative abundance of bacterial phyla Acidobacteria, Chloroflexi, Gemmatimonadetes, Firmicutes and Patescibacteria, and the fungi phyla Mortierellomycota and Zoopagomycota, while decreased bacterial phyla Actinobacteria, WPS-2, Gemmatimonadetes, and Verrucomicrobia, and the fungi phyla Ascomycota, and Basidiomycota. Redundancy analysis analysis indicated soil pH and SOM were the primarily environmental drivers in shaping the rhizosphere soil microbial community in *O. elatus* under varied growth conditions. Therefore, more attention on soil nutrition management especially organic fertilizer inputs should be paid in *O. elatus* cultivation.

## Introduction

*Oplopana elatus* Nakai, is a perennial deciduous shrub plant that belongs to the family Araliaceae, which is a member of ginseng genus^1^. The rhizomes and stems with roots have a long history of use as an ethnomedicine and are valued for their neurasthenic^2^, diabetes mellitus, hypophysis, rheumatism, and cardiovascular properties^2,3^. However, as *O. elatus* requires an altitude of 1400–2100 m, rainfall of about 1200 mm, and habitat moisture of 80%, it is only distributed in east Russia, north of the Korea Peninsula, and the Changbai Mountains of China^4^. Uncontrolled harvesting driven by intense demand has endangered its existence worldwide^5,6^. Therefore, artificial breeding may be an indispensable way to protect and utilize the wildlife resource of *O. elatus*^5^. In China, *O. elatus* has been listed as a national second-grade protected plant species. Seeds of *O. elatus* belong to the post-embryonic dormant type, which need to be physiologically matured before germination^6^. Consequently, the long dormancy time, low seed germination rate, and poor seed setting^1^, which is the main reason for depletion of *O. elatus* in wild population leading to its vulnerable condition^2^. Recently, asexual and sexual reproduction methods have been explores in *O. elatus* breeding. However, low germination rate remains a major limitation. Currently, the seed priming methods, which involves the combination of indoor artificial controlled temperature pretreatment and outdoor changed temperature treatment by natural burial, could increase the seed germination rate of *O. elatus* from less than 30% in nature to nearly 70%^7^. Those encouraging progress in artificial cultivation of *O. elatus* are of great interest for the conservation and utilization in *O. elatus* in China^2^. However, the relations of *O. elatus* plant growth and development between natural and cultivated conditions remain unclear.

Soil is a fundamental condition for plant growth and is a key component of agricultural productivity. Soil physical and chemical properties, as well as the vast rhizosphere microbial community, play a vital role in maintaining a functional balance in agroecological systems^8,9^. In terrestrial ecosystems, plants can influence the soil organic compounds and nutrient cycling they grow in, and via these chemical changes in the soil they can significantly affect other plants that subsequently grow in this soil; a phenomenon called “plant–soil feedback” effect^10,11^. In forest or agrosystem, the rhizosphere is one of the most active areas of microbial activity for the host plant. Rhizosphere microorganisms generally play a key role in participating in this feedback regulation because they can absorb organic substances from host plants and also transport nutrients and water from the soil to the plants^12^, such as some nitrogen-fixing bacteria, cyanobacteria, actinomycetes, which consequently enhances the availability of many essential nutrients and improves the stability of terrestrial ecosystems^11^. Moreover, some rhizosphere microorganisms can provide several beneficial functions for plant growth^13^, including by improving plant stress resistance, resisting soil-borne diseases, and regulating the plant immune response to enhance plant grow^14,15^. Hence, the rhizosphere microbiome has a great contribution to plant survival and growth during the initial stage under new artificial growth condition. Therefore, a better understanding the response of the rhizosphere microbial community of *O. elatus* to changed environments is of great interests.

In addition, the soil environment is involved in the feedback regulation of the plant-soil interface^16,17^, particularly the soil physical and chemical properties, which play a key role in shaping soil microbial structure and diversity^18,19^. Previous studies have demonstrated the process of domestication is the result of plant–microbe coevolution, and the plant-microbial interactions have been proved to contribute to the evolution of terrestrial plants^12,20^. Among the numerous environmental factors, soil pH is regarded as the primary driving factor affecting microbe structure and function. This phenomenon has been confirmed in farmland ecosystems^21–23^, as well as in grassland and forest ecosystem^17^. However, the driving factors may differ among various ecosystems due to variances in landscapes, land use type, latitude and longitude, and disturbance intensity^24,25^. As known, land use and human activities under cultivated conditions are higher than the wild growing environment of *O. elatus,* which may have a direct effect on plant growth and subsequently on soil microbial communities^26^. However, little is known about the associated soil microbe community respond to the shifted growth environment between cultivated and natural conditions in *O. elatus* plant*.*

Thus, the objective of the present study was: (I) to examine how rhizosphere microbial community structure, diversity, and the associated microorganisms respond to varied growth environments under cultivated and natural conditions; (II) to analyze the relationship between the characteristics of the soil microbial community and soil chemical properties and identify factors driving factors driving the rhizosphere soil microbial community change in the artificial cultivation of *O*. *elatus*.

## Results

### Variance analysis of soil physicochemical properties

The soil chemical properties of the six samples of each treatment are shown in Table 1. The ANOVA for rhizosphere soil properties showed significant (*P* < 0.05) differences in pH, SOM (soil organic matter), and AN (Available nitrogen) in samples from WT and CT. According to the results, soil pH was 4.92 in CT, which was significantly higher than in WT. In addition, soil samples in CT had a significantly lower SOM and AN content, while no significant (*P* > 0.05) differences in AP (Available phosphate) and AK (Available potassium) content were observed between soil samples from WT and CT (Table 1).

**Table1:** Chemical properties of sampled soils under cultivated conditions (CT) and wild growth conditions (WT).

Treatment	pH	SOM (g kg^−1^)	AN (mg kg^−1^)	AP (mg kg^−1^)	AK (mg kg^−1^)
WT	4.27 ± 0.09b	261.24 ± 45.6a	1061.53 ± 88.07a	37.58 ± 4.61a	215.3 ± 17.68a
CT	4.92 ± 4.92a	91.29 ± 30.63b	470.75 ± 135.84b	49.98 ± 12.66a	227.17 ± 60.12a

Values are means ± Standard error (S.E)

Values within the same column followed different letters mean differences significant at 0.05 or 0.01 level. SOM = Soil organic matter, AN = Available nitrogen, AP = Available phosphate, AK = Available potassium; CT = cultivated conditions, WT = wild condition (WT).

### Sequencing analysis and alpha diversity

**Figure 1:**
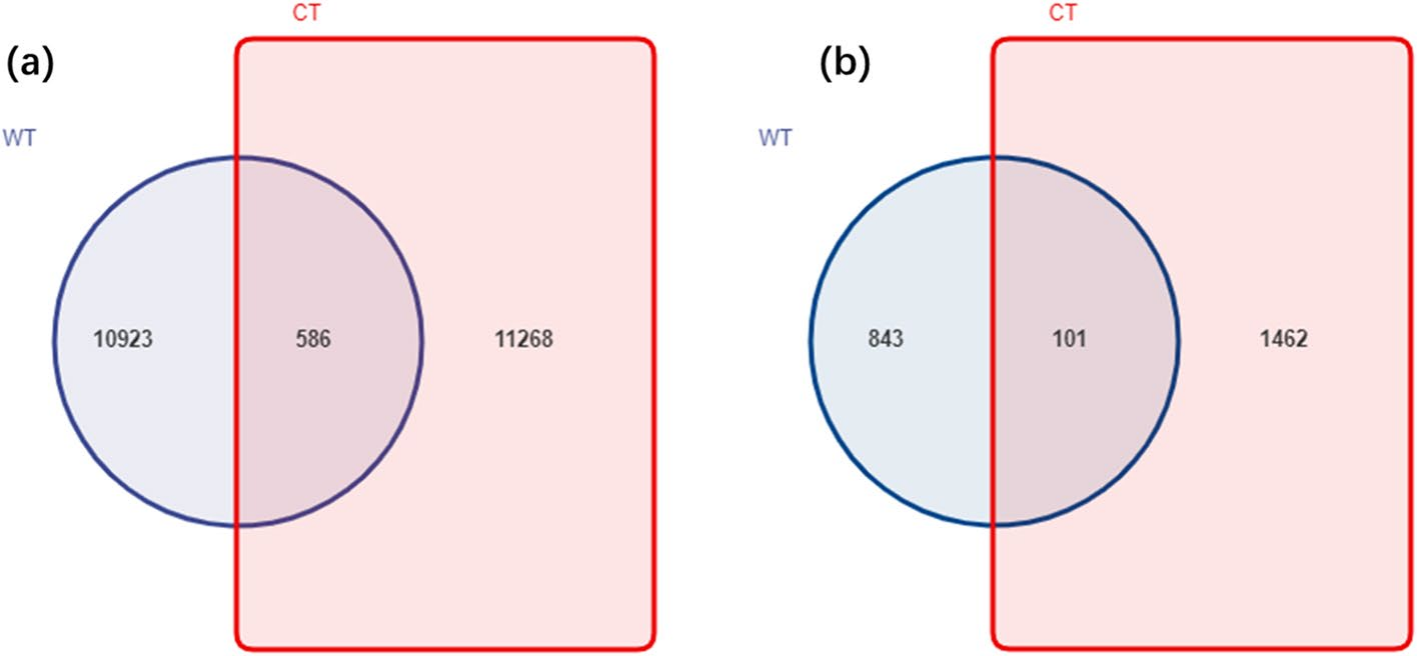
Venn diagram showing the number of unique OTUs of (**a**) bacteria and (**b**) fungi within *O*. *elatus* rhizosphere communities under cultivated (CT) and wild conditions (WT).

Using an Illumina MiSeq HTS platform, a total number of 667,662 merged sequences for bacteria and 55,279 for fungi were obtained (Supplemental Table 1). The number of operational taxonomic units (OTUs) in CT and WT was 11,268 and 10,923 for bacteria rhizosphere communities, respectively, and the number of combined OTUs in all samples was 586 (Fig. 1). Correspondingly, the total number of OTUs was 1,462 and 843 in CT and WT for the fungi community, and the number of combined OTUs in all samples was 101.

To assess the diversity and evenness of microbial populations variations of soil samples from CT and WT, the Chao_1,Observed_species, Pielou_e, and Shannon index were analyzed (Table 2). For bacteria communities, significantly higher Chao_1, Observed_species, and Shannon index values were found in CT compared to WT. Correspondingly, the Chao_1, Observed _species, Pielou_e and Shannon index showed a similar trend in the fungi communities under the two treatments, which indicated that cultivated conditions had significant effect on the soil bacterial and fungi richness and diversity.

**Table 2:** Estimation of bacteria and fungi community diversity under cultivated cultivations (CT) and wild growth conditions (WT).

Category	Treatment	Chao_1	Observed_species	Pielou_e	Shannon index
Bacteria	WT	2746.02 ± 101.9b	2545.72 ± 75.11b	0.8623 ± 0.008a	9.75 ± 0.12b
	CT	3156.33 ± 147.1a	2927.92 ± 137.0a	0.8652 ± 0.006a	9.96 ± 0.11a
Fungi	WT	216.49 ± 29.41B	214.48 ± 29.34B	0.4596 ± 0.087A	3.47 ± 0.79B
	CT	374.08 ± 39.97A	373.48 ± 39.87A	0.6387 ± 0.023A	5.43 ± 0.24A

### Soil microbial community structure and composition

To evaluate the taxonomic diversity of the bacterial and fungal communities in the 12 samples from CT and WT, the taxonomic composition of the microbial communities was classified into different taxonomic levels (from phylum to genus) by blasting the Greengenes database (Release 13.8) for bacteria and the Silva database (Release 132) for fungi. The bacterial community samples were classified into 934 genera in total, which belonged to 32 phyla, 90 classes, 257 orders, and 454 families (Supplemental Table 2–5), while fungi community samples were classified into 541 genera in total, which belonged to 16 phyla, 34 classes, 116 orders, and 272 families (Supplemental Table 2–5).

**Figure 2:**
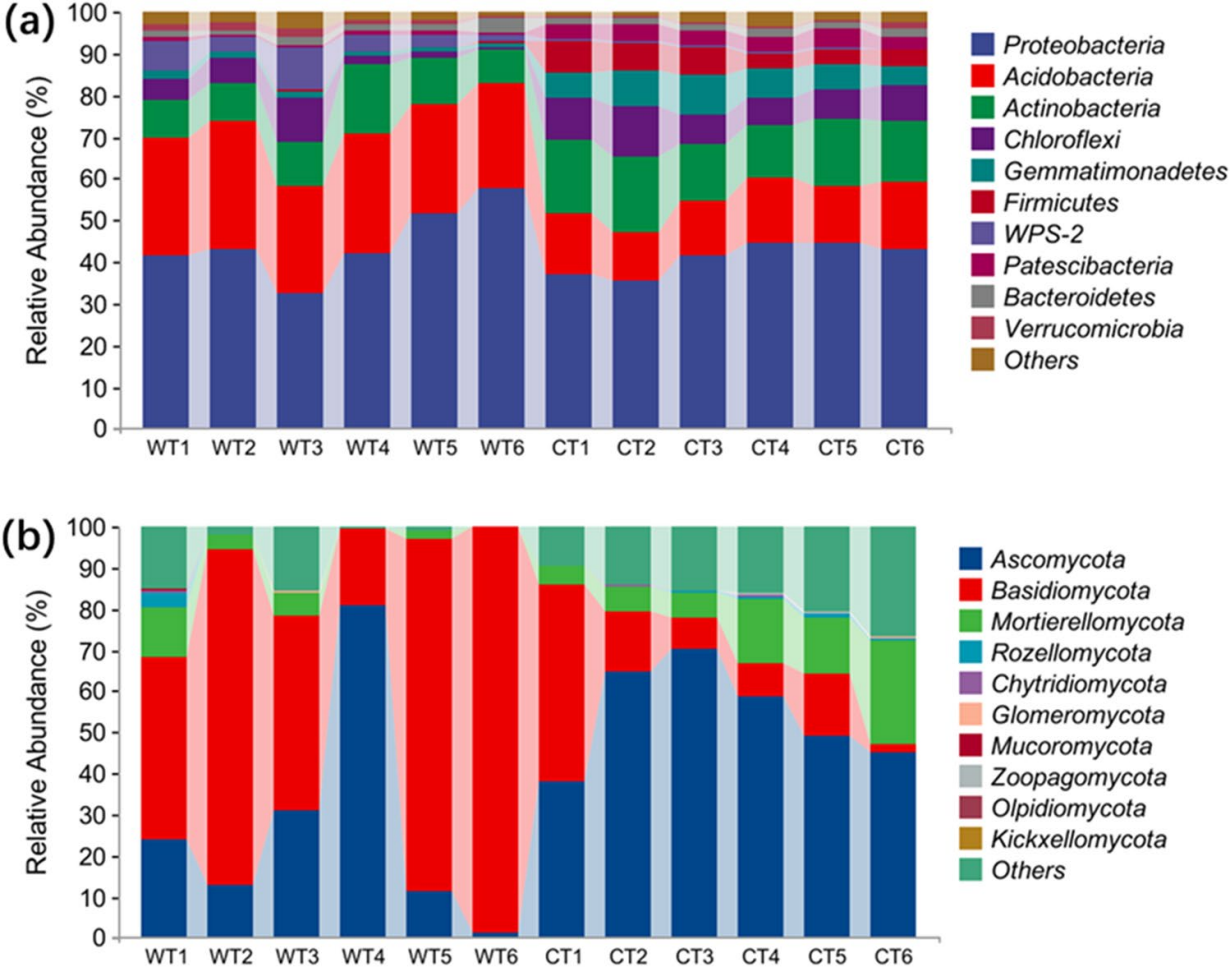
Relative abundance of soil bacterial phyla (**a**) and fungi phyla (**b**) from rhizosphere soil sampled under cultivated (CT) and wild conditions (WT).

At the bactiral phylum level, Proteobacteria (32.37–57.69%), Acidobacteria (12.57–31.05%), *Actinobacteria* (8.06–16.74%), WPS-2 (1.68–9.86%), Chloroflexi (0.85–11.98%), Gemmatimonadetes (0.91–8.42%), Bacteroidetes (0.84–1.84%, and Verrucomicrobia (0.52–2.22%) were the 10 dominant phyla, accounting for approximately 41–98% of the relative abundance of the bacterial communities (Fig. 2a). Compared to WT, the relative bundance of Acidobacteria*,* Chloroflexi, Gemmatimonadetes, Firmicutes and Patescibacteria in CT was significantly enhanced, while that of Actinobacteria, WPS-2, Gemmatimonadetes, and Verrucomicrobia was deceased, and that of Proteobacteria and Bacteroidetes was unchanged.

At the fungi phylum level, Ascomycota (11.15–80.79%), Basidiomycota (2.44–81.7%), Mortierellomycota (0.18–25.07%), and Rozellomycota (0.12–9.86%) were the four dominant phyla, accounting for approximately 72–99% of the relative abundance of the fungi communities (Fig. 2b); In addition, unclassified_Fungi and unidentified were also the predominant phyla, accounting for 0.3–26.72%. Compared to WT, the relative abundance of Mortierellomycota and *Zoopagomycota* in CT was significantly enhanced, while that of Ascomycota, and Basidiomycota was significantly decreased.

**Figure 3:**
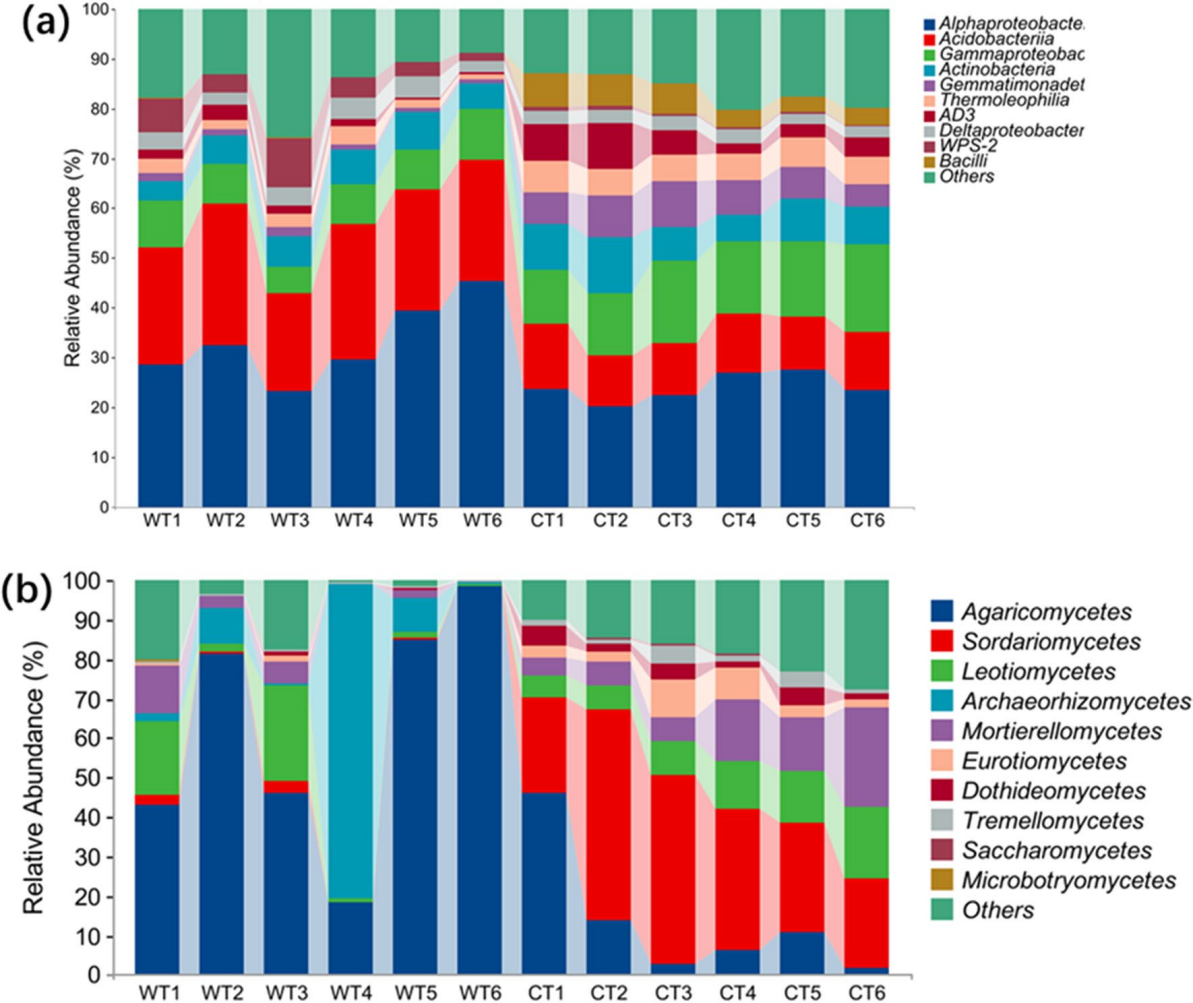
Relative abundance of the 10 most abundant soil bacterial classes (**a**) and fungi classes (**b**) from the rhizosphere soil sampling under cultivated (CT) and wild conditions (WT).

At the bactirial class level, a total of 14 classes had a relative abundance > 1% for each sample, among the 10 most abundant classes (Fig. 3a), the relative abundance of Gammaproteobacteria, Actinobacteria, AD3, Bacilli, Gemmatimonadetes, Deltaproteobacteria, and Thermoleophilia were greatly increased in CT, while that of Alphaproteobacteria, Acidobacteriia and WPS-2 was decreased and that of Bacteroidia and Subgroup_6 were was unchanged, compared to WT.

At the fungi class level, a total of 10 classes had a relative abundance > 1% for each sample, among the 10 most abundant classes, the relative of Leotiomycetes, Mortierellomycetes, Sordariomycetes, unidentified and unclassified_Fungi were greatly increased in CT, while that of Agaricomycetes and Archaeorhizomycetes was significantly decreased, compared to WT (Fig. 3b).

### Relationships of the microbial communities among the different rhizosphere soil samples

**Figure 4:**
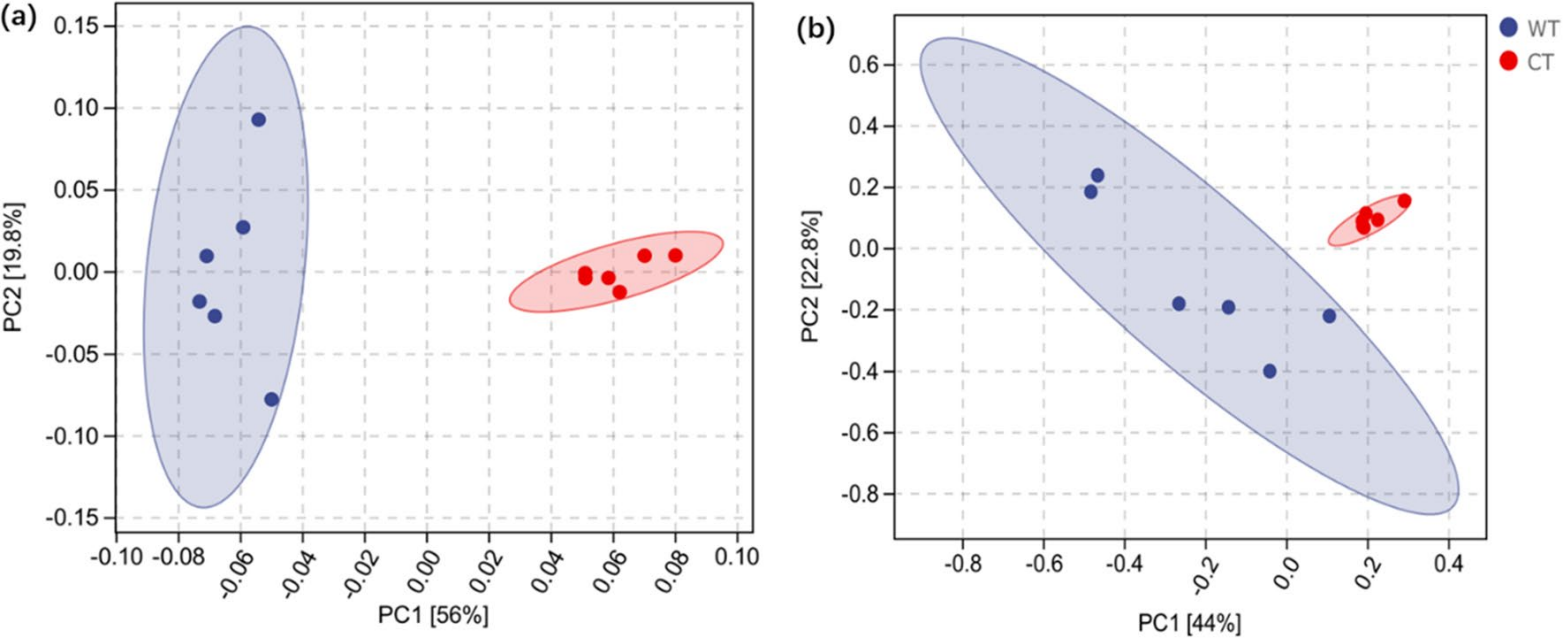
Beta diversity analysis of (**a**) bacteria and (**b**) fungi communities in in *O. elatus* rhizosphere soil from cultivated (CT) and wild (WT)conditions by using principal component analysis (PCA) based on Bray–Curtis distances.

**Figure 5:**
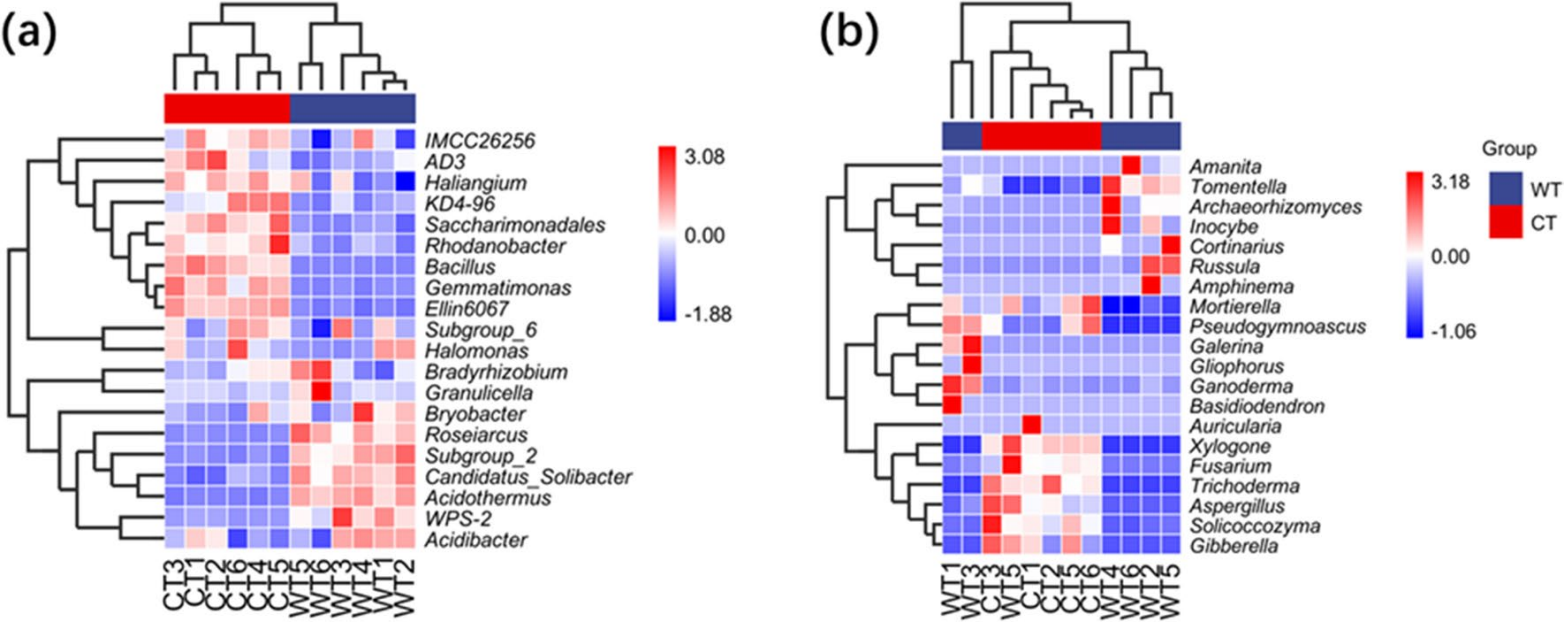
Hierarchical clustering heatmap of the relative abundance of the 20 most abundant (**a**) bacterial and (**b**) fungi genera, using Euclidean distance. Rows represent different samples, and the column shows the relative percentage of each microbial genus. The relative abundance of each bacterial genus is depicted by the color intensity in the figure below; CT = cultivated conditions, WT = wild conditions. Hierarchical clustering heatmap were created using the online program (https://www.genescloud.cn/chart/HeatMap).

To investigate the relationships of the rhizosphere soil basic characteristics and microbial community properties, principal components analysis (PCA) was performed among samples from CT and WT at the genus level. For bacterial communities, 12 samples were divided into two groups, the PCA score plot showed that the six samples from CT grouped to the right of the graph along the first principal component (PC1), accounting for 56% of the variation, while the other six samples from WT closely clustered on the left of PC2, which represented 19.8% of the total variations (Fig. 4a). For fungal communities, the axes of PC1 and PC2 explained 44% and 22.8% of the sample variance (Fig. 4b). A hierarchically clustered heatmap was further constructed to evaluate the relationships among the samples at the genus level based on the top 20 most abundant microbial communities. The result showed that the 12 soil samples could be separated into two groups (Fig. 5a). The most abundant genera were *AD3* (9.28%) in CT (in red) and *Subgroup_2* (10.10%) in WT, respectively. The most abundant bacterial taxonomic groups of *IMCC26256*, *AD3*, *Haliangium*, *KD4-96*, *Saccharimonadales*, *Rhodanobacter*, *Bacillus*, *Gemmatimonas*, *Ellin6067*, *Subgroup_6*, *Halomonas* were detected as increased in CT, while *Bradyrhizobium*, *Granulicella*, *Bryobacter*, *Roseiarcus*, *Subgroup_2*, *Candidatus_Solibacter*, *Acidothermus*, *WPS-2*, and *Acidibacter* were deceased. For fungi, the most abundant genus of *Trichoderma*, *Mortierella*, *Pseudogymnoascus*, *Xylogone*, *Aspergillus*, *Auricularia*, *Fusarium*, *Solicoccozyma*, and *Gibberella* were increased in CT, while *Archaeorhizomyces*, *Amanita*, *Russula*, *Cortinarius*, *Galerina*, *Ganoderma*, *Inocybe*, *Basidiodendron*, *Gliophorus*, *Amphinema*, and *Tomentella* were decrease (Fig. 5b). The heatmap analyses (Fig. 4) agree with the results of the PCA (Fig. 5a), with both analyses indicating that soil samples in CT had a great effect on the rhizosphere soil characteristics and microbial communities.

### Correlation analysis of soil microbial community with soil chemical properties

**Figure 6:**
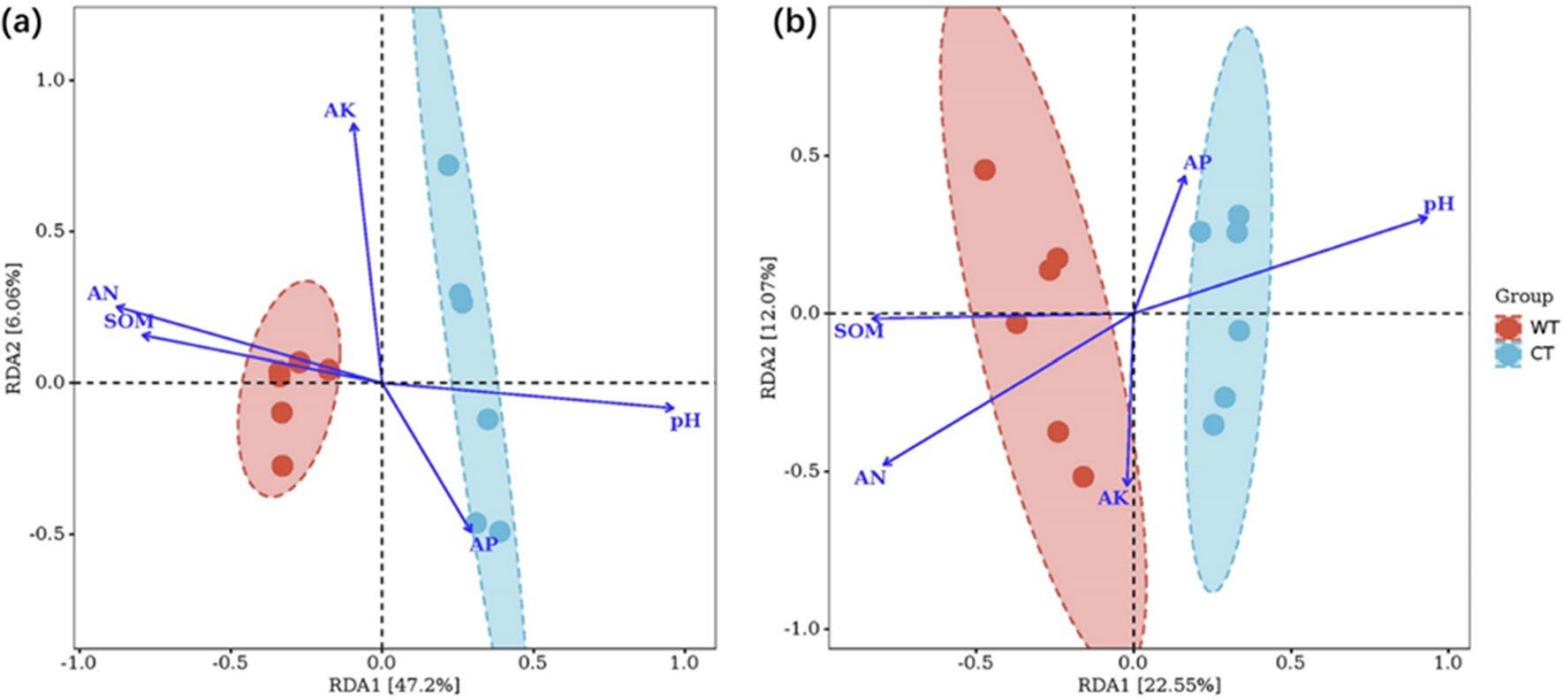
Redundancy analysis (RDA) plot showing the relationship between soil bacterial (**a**) and fungal (**b**) community structure and the soil properties explained by soil pH, soil organic matter content (SOM), available nitrogen (AN), available phosphorus (AP), available potassium (AK) under cultivated (CT) and wild conditions (WT). Arrows pointing in the same direction indicate positive correlations, and arrows pointing in opposite directions indicate negative correlations. The arrow length corresponds to the variance explained by the environmental variable.

The RDA was conducted to analyze the correlation between soil microbial community and soil chemical properties (Fig. 6). The output of the RDA explained 53.25% of the total variation of the bacterial community (Fig. 6a) and 34.62% of fungal community variation (Fig. 6b). For the bacteria community, the RDA analysis indicated soil pH played an important role in the structure of the bacterial community, followed by the content of AN, SOM, AP and AK. Of the five parameters, pH had large positive values on RDA1, and SOM has large positive values on RDA2 (Fig. 6a). For the fungal community, the soil chemical properties of SOM and pH had a major impact on the soil structure of the fungal community, followed by the content of AN, AK and AP, while pH had large positive values on RDA1, SOM, AN and AK have large positive values on RDA2 (Fig. 6b).

## Discussion

Artificial breeding is crucial to the protection and utilization of endangered wildlife resources. When plant transplanted form the natural habitats to a controlled growth environment, soil quality is one of the critical factors affecting plant growth and development. In the present study, ANOVA analysis revealed significant difference in soil pH, the content of SOM and AN between WT and CT. Compared to WT, soil samples in CT had a higher pH, but a lower content of soil SOM and AN. As known, land use patterns is one of the important factors affecting soil nutrient availability^27^. Compared to forest, the long-term low amount of carbon input and high deposition rate in cultivated conditions may be the main reason for lower SOM in CT conditions^28,29^. Moreover, soil in forest generally has high soil organic matter humification efficiency and the deposition rate of acidifying substance^30^, resulting in a lower soil pH in WT conditions. To date, cultivation trials have not been evaluated to understand the most favorable growing conditions and soil requirements for *O*. *elatus*^2^. Nevertheless, beneficial soil conditions with high soil organic matter and sufficient nutrient supply are favorable for plant growth. Our study showed that the current soil nutrient status in cultivated conditions can meet the demand of *O. elatus* plant growth in this region (Figure S1). However, more detailed information about the relationship on soil supply and *O. elatus* plant nutrient requirements should be investigated in future.

The environmental conditions under specific land use models have great influence on soil microbial communities and diversity^10^. It has been well documented the bacterial communities in soil with stable environment under natural habitats are more sensitive to changed soil conditions than those from more variable soil environment^31,32^. In the present study, significant differences in soil bacterial alpha diversity were observed between CT and WT, and the higher Chao_1, Observed_species, and Shannon index values were found in CT. This is in agreement with previous findings that land-use intensity increased the soil bacterial diversity ^22,33^. Normally, higher land use management exerts negative effects on the inhabiting microbe species and decreases the soil fungi diversity^34^. Previous literatures have shown that soil fungi diversity in forest system is mainly regarded for stand structure of plant canopy, decomposition stage of deadwood in ground surface, as well as anthropogenic disturbances^30,34^. To stimulate the natural habitat growth environment of *O. elatus* plant, organic fertilizer was added to the CT soil in this study. The additional organic compounds and residual fertilizer from the previous crop could result in the increase in fungal diversity under CT treatment. Similar results of agricultural management have been demonstrated in farmland ecosystems, such as short-term straw incorporation and manure application caused increase in soil microbial diversity^23,32^.

Plant growth environmental factors, particularly soil chemical properties of pH^21^, organic matter content, and C:N rate are the mainly driving force of soil microbial communities responded to the changed envrioment^35,36^. The results of our study revealed that the structure of soil bacterial and fungal communities differed between the CT and WT treatments. Compared to WT, the three dominant bacterial phyla of Acidobacteria, Chloroflexi, Gemmatimonadetes, and two non-dominant (Firmicutes, Patescibacteria) bacterial phyla, were enhanced, and the three dominant bacterial phyla of WPS-2, Gemmatimonadetes*,* and Verrucomicrobia and one non-dominant bacterial phyla of Actinobacteria were decreased. Members of the Acidobacteria bacterial phylum are pervasive and copiously distributed across nearly all ecosystems. They act as plant growth–promoting bacteria, and a shift in these rhizosphere soil microbiota reflects the plant-soil feedback regulation under CT conditions. A similar study in agroecosystem reported that continuous monoculture cultivation resulted in compositional changes in the soil microbiota in rice plants^37^. Chloroflexi and Firmicutes bacteria usually constitute a substantial proportion of the bacterial communities in rhizosphere soils and may play an important role in utilizing geochemical inputs such as sulfide from upslope weathering^16^. Gemmatimonas is a very slow-growing bacterium which is able to utilize substrates as sole carbon sources for energy. This is beneficial for plant growth under a changed habitat (e.g., soil pH, PM content) under CT conditions. This also corroborates with the previous study that cold and water-saturated environment induced a high relative abundance of novel Chloroflexi, which can act as a stable and resistant life-strategy in response to abiotic environmental conditions in alpine tundra wet meadow soil^38^.

At the fungal phylum level, the relative abundance of Mortierellomycota and Zoopagomycota in CT was significantly enhanced. Mortierellomycotina and Zoopagomycota are a group of early divring fungi that are frequently associated with plant rhizospheres^15^. The increase in rhizosphere Mortierellomycotina and Zoopagomycota may be associated with the microbial community structure resilience under changed environmental conditions^11^. Most soil-inhabiting Basidiomycota are associated with woody plants and live in colonized natural or relatively undisturbed forests^39^. The reduced relative abundence of Mortierellomycotina indicated the high intensity land use environment may unfavorable to some types of fungal microbes.

To our knowledge, soil quality has a significant effect on the aggregation of rhizosphere bacteria and fungi communities. In turn, soil microbial community composition is closely related to the changes of soil available nutrients^23^. In northeastern China, soil pH is regarded as the primary factor driving the distribution and function of microorganisms in farmland soils^21^. Increasing evidence based on the combination of 16S rRNA sequencing data and corresponding functional profiles from 150 forest and 150 grassland soils also proved that soil pH is the best predictor for bacterial community structure, diversity, and function in temperate grasslands and forests^17^. These results are consistent with the present study, in that soil pH played an important role in shaping the structure of the bacterial and fungi community under short-term domesticated cultivation (Fig. 6, Fig.S3). In agroecosystems, the availability of SOM is a complex bio-mediated process involving soil microbes, but it is also considered as an overarching edaphic factor dominating soil microbial diversity^40^. The RDA analysis indicated, SOM played a key role in influencing soil microbial community structure (Fig. S3 and S4). This result corroborates a previous study that found that the mineralization of SOM is a complex bio-mediated reaction in which organic substrates are converted into living biomass and mineral residues^25^, thus providing substance and energy to the soil microbiome. Therefore, more attention to soil nutrition management especially organic fertilizer inputs should be paid in O. elatus artificial cultivation.

## Conclusion

In this study, we compared the rhizosphere soil chemical properties and soil microbial communities of *O. elatus* plant growth in natural and cultivated conditions. We found the soil in cultivated conditions had higher pH and lower content of soil organic matter (SOM) compared to natural habitat. The changed growth environment caused by transplant significantly influenced the structure and diversity of rhizosphere soil microbial community. Compared to the natural habitat, the cultivated conditions resulted in a higher soil bacterial and fungi richness and diversity in Changbai Mountain, Northeast of China. Our study showed that 32 phyla, 90 classes, 257orders and 454 families in the rhizosphere bacterial community and, 16 phyla, 34 classes, 116 orders and 272 families in the fungal community were influenced by changed growth conditions. RDA analysis between soil microbial community and soil chemical properties revealed that soil pH and SOM may act as the main factor on shaping the rhizosphere soil microbial community of *O. elatus* in cultivated conditions.

## Materials and methods

### Site description and artificial cultivation experimental design

**Figure 7:**
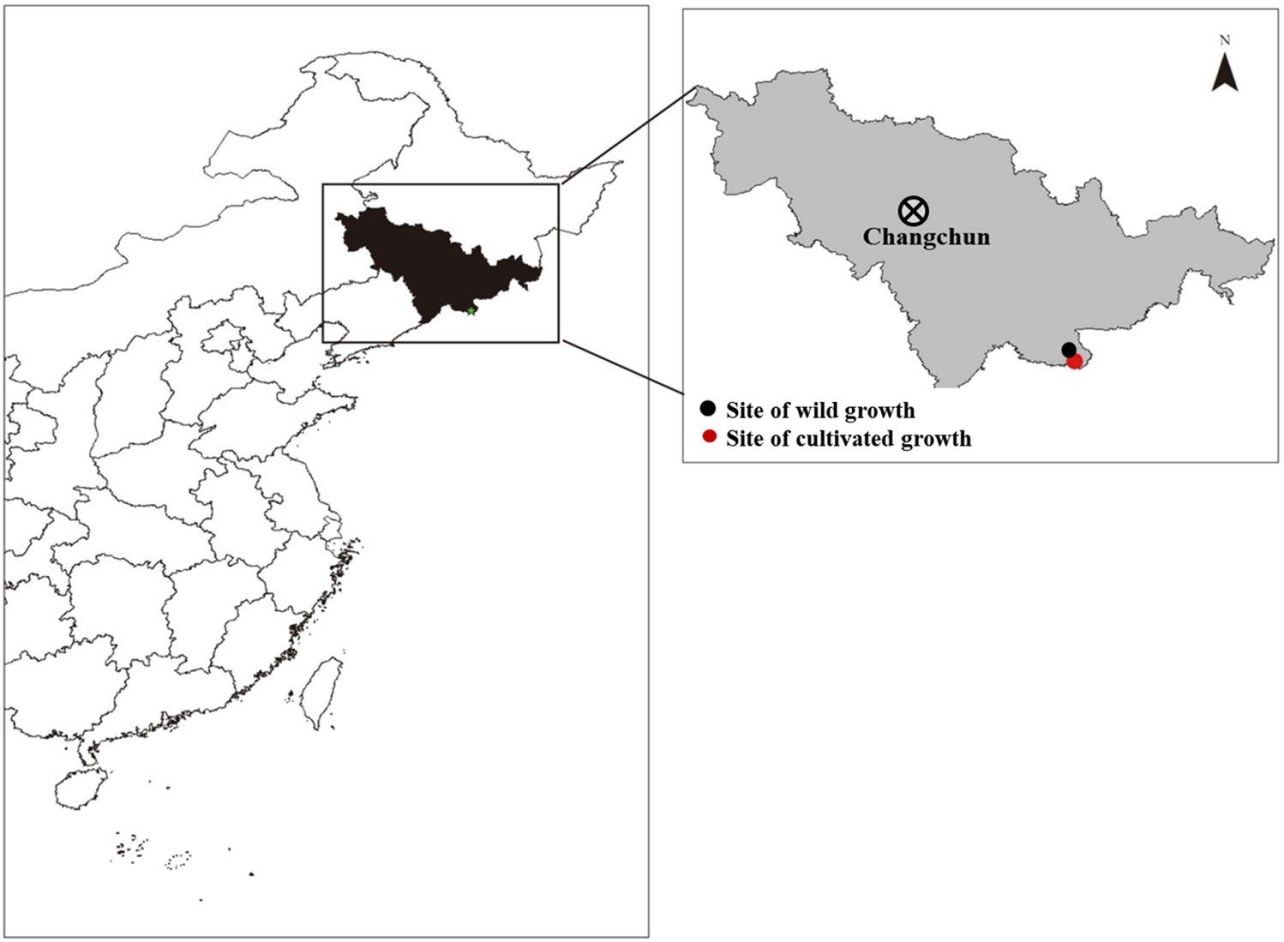
Location of the experimental sites. The black and red dots represent the sites where growled in cultivated and wild conditions.

The wild growth site was located in the forest of Shisidaogou town (Fig. 7), Changbai Korean Autonomous County (41°28′46″N, 128°2′23″), which belongs to the Changbai Mountain Nature Reserve of China, with an altitude of 836 m and an atmospheric pressure of 91.68 kPa. This region has a huge forest coverage of 87.9%. The climate is a north monsoon climate that is greatly impacted by topography, with a long cold winter and short, warm, and humid summer. The mean annual temperature ranges from − 7 °C to 3 °C, the lowest temperature in severe winter can reach − 40 °C, the annual sunshine duration is 2300 h, and the mean annual precipitation is 700–1400 mm.

The cultivated site is not far from the wild site (Fig. 7). The previous crop at the experimental site was maize with 2 years continuous planting. Before *O. elatus* cultivation, all the weeds and shrubs in the planting area were cut down and removed in the fall of last year, then plenty of burrow-shaped land with area of 1 m × 1 m, and 30–35 cm deep were prepared. After that about 80–100 kg organic manure (SOC 14.5%; AN 0.8%; AK 0.45%; pH 5.5) were added to the burrow for *O. elatus* planting.

### Seed germination and transplant

The seeds from wild plants soaked in warm water at 40 °C for 2 days, then transferred to 1% CuSO_4_ solution for 2 to 3 h, rinsed with five times with distilled water. Then, the seeds evenly mixed with the fine sand at the ratio of 1:3 (seed/sand).After that, the treated seeds were placed in a condition controlled incubator at the temperature of 18 ± 0.5 °C, humidity 60% for 4 months, and then put into another chamber at 4 °C for 3 months. In the spring of the following year, the treated seeds were put in a disc with 4 layers of filter paper for seed germinated at temperature of 25 °C. Finally, the seeds were sown in the soil in natural habitat site.

At the spring of 2020, the young seedlings of *O. elatus* were transplanted to the farm in cultivated conditions, then all the soil around the plants was covered with a layer of leaves, and the light transparency was controlled at 60%.

### Collection and preparation of rhizosphere soil

Soil sampling was carried out in July 2021 at the Changbai Mountain in Northeast China, two years after transplantation. Rhizosphere soil is defined as the portion of soil found adjacent to the roots of living plants and influenced by root activity. At each site, six healthy plants were randomly selected, then the soil was carefully excavated from around single plants down to approximately 25 cm (root depth most no more than 15 cm), the root was carefully and completely sampled, the loose soil was gently removed by shaking the roots, and the rhizosphere soil adhering to the roots was collected about 50–80 g separately from the root surface using a brush. Each soil sample was sieved through a 2-mm sieve after removing roots and rocks and divided into two parts: one of which is about 10 g was carefully placed into sterile ziplocked bags, immediately frozen, and stored at − 80 °C to extract total soil DNA for high-throughput sequencing and soil microbial biomass measurements, and the remaining parts was air-dried for measurements of available nutrients and pH.

### Soil chemical analysis

Soil organic carbon (SOC) was determined by the dichromate oxidation method and followed the instructions of a previous study^29^, and soil organic matter content (SOM) was calculated based on the content of SOC multiply a coefficient of 1.724^41,42^. Soil available nitrogen (AN) was measured by alkaline hydrolysis diffusion method and following the description of Chen et al^43^. Available phosphorus (AP) was determined by the molybdenum-antimony colorimetric method after sodium bicarbonate extraction^44^. and available potassium (AK) were extracted using 1 M NH_4_OAc, pH 7 solution and determined by a flame photometer (Sherwood Scientific, M410 C, UK)^42^. Soil pH was measured in a 1:5 soil: deionised water suspension using a pH meter (DDS-307, Wuxi Leixi Instrument Co., Ltd., China).

### DNA extraction and MiSeq sequencing

Soil DNA was extracted from each sample using MoBio PowerSoil DNA extraction kit (MoBio, Carlsbad, USA) according to the manufacturer’s protocol. The extracted DNA was determined quantitatively with a Qubit 2.0 Test Kit (Life, Waltham, MA, USA). Purified soil DNA was fully pooled together after quantitatively determination and then for downstream manipulations. The V3-V4 of bacterial 16 S rRNA genes were amplified with the following universal primer set: upstream primers 338F (5′-ACTCCTACGGGAGGCAGCA-3′) and downstream primers 806R (5′-GGACTACHVGGGTWTCTAAT-3′). For fungi, the primers ITS5 (5′-GGAAGTAAAAGTCGTAACAAGG -3′) and ITS2 (5′-GCTGCGTTCTTCATCGATGC-3′)^45^ were used to amplify the ITS_V1 region of the rDNA gene. PCR reactions were performed in 50 μL reaction mixtures containing 10 μL 5 × FastPfu Buffer, 5 μL 4 mM dNTPs, 2.0 μL primers, 25 ng pooled DNA and 1 U polymerase. The reaction conditions were programmed of an initial denaturing step at 94 °C for 3 min, five cycles of 30 s at 94 °C, 20 s at 45 °C, 30 s at 65 °C, 20 cycles of 94 °C for 20 s, 1 min at 72 °C, and a final cycle of 5 min at 72 °C. The PCR amplicons were purified with VAHTS DNA Clean Beads (Vazyme, Nanjing, China) following the manufacturer’s instructions. Six samples of each treatment and 200 ng purified products DNA of each sample were pooled and sequenced in an Illumina MiSeq High-Throughput Sequencing (HTS) platform (Illumina, San Diego, CA, USA) at Personal Biotechnology Co. Ltd Shanghai, China to determine soil microbial community composition.

### Statistical analyses

The sequenced data was performed using QIIME 2 2019.4^46^ with slight modification. Briefly, raw sequence data were demultiplexed using the demux plugin followed by primers cutting with cutadapt plugin. Sequences were then merged, filtered and dereplicated using functions of fastq_mergepairs, fastq_filter, and derep_fulllength in Vsearch. All the unique sequences were then clustered at 98% (via cluster_size) followed by chimera removing. At last, the non-chimera sequences were re-clustered at 97% to generate OTU representive sequences and OTU table. Representive sequences were aligned with mafft and used to construct a phylogeny with fasttree2^47^. Alpha-diversity metrics (Chao1, Observed species, Shannon, Pielou’s evenness), beta diversity metrics (Bray–Curtis dissimilarity) were estimated using the diversity plugin with samples were rarefied. Taxonomy was assigned to ASVs using the classify-sklearn naïve Bayes taxonomy classifier in feature-classifier plugin against the Silva v132 99% OTUs reference sequences^48^.

Venn diagrams were generated using the VENNY online program (http://bioinfogp.cnb.csic.es/tools/venny/). Hierarchical clustering heatmap of the relative abundance of the 20 most abundant bacterial and fungi genera were created using the online program (https://www.genescloud.cn/chart/HeatMap). Redundancy analysis (RDA) between soil microbe community structure and the soil chemical properties was conducted using genescloud tools (https://www.genescloud.cn); a free online platform for data analysis. All the basic statistical analyses were also performed by the genescloud tools, and one-way ANOVA of soil chemical properties between two treatments was conducted using IBM SPSS 24.0 statistical software (IBM Corp., Armonk, NY, USA).

## Supplementary Information

Supplementary Information 1.

Supplementary Information 2.

## Data Availability

The datasets analyzed during the current study are available in the Sequence Read Archive (SAR) NCBI database repository as below: (Sequences of bacteria BioProject ID: PRJNA812683) https://dataview.ncbi.nlm.nih.gov/object/PRJNA812683?reviewer=qnvu3r8e13peifgagudt4cp8s2 and (Sequences of fungi BioProject: PRJNA812689) https://dataview.ncbi.nlm.nih.gov/object/PRJNA812689?reviewer=e195r5i2r20qu0nbbnqcs0t5ne.
