# Supplementary material for: Shifts in rhizosphere microbial communities in Oplopanax elatus Nakai are related to soil chemical properties under different growth conditions
Source: Sci Rep. 2022 Jul 7;12:11485. doi: 10.1038/s41598-022-15340-1 (PMC9262954; doi:10.1038/s41598-022-15340-1)
Supplement: Supplementary file 1 — Supplementary Information 1. [file 41598_2022_15340_MOESM1_ESM.docx]

**Supplemental table legend**

**Supplemental table 1** Statistical table of sequencing amount per sample in (a) bacteria and (b) fungi

**Supplemental table 2** Soil (a) bacterial and (b) fungal_OTU_table at phylum

**Supplemental table 3** Soil (a) bacterial and (b) fungal_OTU_table at class

**Supplemental table 4** Soil (a) bacterial and (b) fungal_OTU_table at order

**Supplemental table 5** Soil (a) bacterial and (b) fungal_OTU_table at family

| **Supplemental table 1 a** Statistical table of sequencing amount per sample in bacteria | | | | | | |
| --- | --- | --- | --- | --- | --- | --- |
| **Sample ID** | **Input** | **Filtered** | **Denoised** | **Merged** | **Nonchimeric** | **Nonsingleton** |
| WT_1 | 97619 | 88052 | 84712 | 74275 | 68202 | 67247 |
| WT_2 | 83228 | 76089 | 72991 | 63249 | 56529 | 55694 |
| WT_3 | 95540 | 87497 | 84798 | 75741 | 71263 | 70626 |
| WT_4 | 81117 | 72371 | 68985 | 56010 | 49255 | 48165 |
| WT_5 | 82991 | 74522 | 70866 | 58060 | 50805 | 49939 |
| WT_6 | 73368 | 66384 | 62568 | 49793 | 41034 | 40000 |
| CT_1 | 73284 | 66225 | 62036 | 47636 | 40611 | 39188 |
| CT_2 | 93676 | 83589 | 79920 | 67117 | 59031 | 57635 |
| CT_3 | 96968 | 87019 | 83079 | 70507 | 63069 | 61561 |
| CT_4 | 83445 | 76140 | 72149 | 57775 | 51506 | 49775 |
| CT_5 | 88489 | 79556 | 74109 | 56271 | 48665 | 46402 |
| CT_6 | 90734 | 81998 | 77735 | 64288 | 57034 | 55039 |

**Supplemental table 1 b** Statistical table of sequencing amount per sample in fungi

| **SampleID** | **Input** | **Filtered** | **Denoised** | **Merged** | **Nonchimeric** | **Nonsingleton** |
| --- | --- | --- | --- | --- | --- | --- |
| WT_1 | 78737 | 70476 | 70107 | 69676 | 69239 | 69239 |
| WT_2 | 70111 | 64170 | 63916 | 63566 | 61509 | 61509 |
| WT_3 | 68567 | 61415 | 61179 | 60609 | 60241 | 60241 |
| WT_4 | 62643 | 55407 | 55139 | 54891 | 54339 | 54339 |
| WT_5 | 70365 | 62714 | 62302 | 62057 | 61644 | 61644 |
| WT_6 | 67910 | 62690 | 62543 | 62474 | 62458 | 62458 |
| CT_1 | 63350 | 50176 | 49402 | 48613 | 48034 | 48034 |
| CT_2 | 68761 | 45952 | 45420 | 44981 | 44287 | 44286 |
| CT_3 | 72547 | 52314 | 51895 | 51578 | 51346 | 51345 |
| CT_4 | 71878 | 52626 | 52263 | 51682 | 51206 | 51206 |
| CT_5 | 60241 | 51114 | 50604 | 49967 | 49742 | 49742 |
| CT_6 | 64921 | 49169 | 48890 | 47568 | 46670 | 46670 |

**Supplemental table 2a** Soil bacterial_OTU_table at phylum

| **Taxon** | **WT_1** | **WT_2** | **WT_3** | **WT_4** | **WT_5** | **WT_6** | **CT_1** | **CT_2** | **CT_3** | **CT_4** | **CT_5** | **CT_6** |
| --- | --- | --- | --- | --- | --- | --- | --- | --- | --- | --- | --- | --- |
| d__Bacteria;p__Acidobacteria | 0.281199 | 0.310446 | 0.258787 | 0.28774 | 0.261659 | 0.250225 | 0.144747 | 0.117714 | 0.131227 | 0.157389 | 0.132721 | 0.157631 |
| d__Bacteria;p__Actinobacteria | 0.092925 | 0.091518 | 0.102501 | 0.167404 | 0.112117 | 0.0806 | 0.177633 | 0.179487 | 0.134721 | 0.125726 | 0.163777 | 0.14738 |
| d__Bacteria;p__Armatimonadetes | 0.001681 | 0.000503 | 0.00092 | 0.001453 | 0.000961 | 0.00025 | 0.00074 | 0.000521 | 0.003364 | 0.002211 | 0.001423 | 0.003671 |
| d__Bacteria;p__Atribacteria | 0 | 0 | 0 | 0 | 0 | 0 | 5.11E-05 | 0 | 0 | 0 | 0 | 0 |
| d__Bacteria;p__BRC1 | 0 | 0 | 0 | 0 | 4.00E-05 | 0 | 0 | 5.21E-05 | 0.00026 | 0.000181 | 0 | 0.000545 |
| d__Bacteria;p__Bacteroidetes | 0.016479 | 0.008367 | 0.016257 | 0.016464 | 0.016881 | 0.03245 | 0.014273 | 0.017005 | 0.017451 | 0.019741 | 0.018439 | 0.016757 |
| d__Bacteria;p__Chlamydiae | 0.002231 | 0.003447 | 0.001303 | 0.001474 | 0.000441 | 0.000575 | 0.001506 | 0.001284 | 0.00351 | 0.002473 | 0.000906 | 0.002345 |
| d__Bacteria;p__Chloroflexi | 0.051846 | 0.060114 | 0.105999 | 0.018457 | 0.014257 | 0.008475 | 0.101672 | 0.119849 | 0.07213 | 0.064693 | 0.068539 | 0.08693 |
| d__Bacteria;p__Cyanobacteria | 0.002588 | 0.000916 | 0.001529 | 0.002388 | 0.000741 | 0.000525 | 0.000204 | 0.000972 | 0.000747 | 0.001669 | 0.000561 | 0.000254 |
| d__Bacteria;p__Deinococcus-Thermus | 8.92E-05 | 0.000126 | 0.000963 | 0 | 0 | 0 | 0 | 0 | 0 | 0 | 6.47E-05 | 0.000109 |
| d__Bacteria;p__Dependentiae | 0.004135 | 0.003968 | 0.002195 | 0.003862 | 0.001221 | 0.0008 | 0.002221 | 0.004616 | 0.010546 | 0.008544 | 0.007893 | 0.009996 |
| d__Bacteria;p__Elusimicrobia | 0.00528 | 0.003842 | 0.005268 | 0.004422 | 0.001982 | 0.000925 | 0.00097 | 0.000868 | 0.000666 | 0.001347 | 0.000949 | 0.000763 |
| d__Bacteria;p__Entotheonellaeota | 0 | 0 | 0 | 0 | 0 | 0 | 0 | 0 | 0 | 0 | 0 | 0 |
| d__Bacteria;p__FBP | 0 | 0 | 0 | 0 | 0 | 0 | 5.11E-05 | 0 | 0.000146 | 8.04E-05 | 0 | 0.0002 |
| d__Bacteria;p__FCPU426 | 0.000654 | 0.000251 | 0.000198 | 0.000561 | 0.000481 | 0.000725 | 0.000204 | 0.000399 | 0.000211 | 0.000101 | 0 | 0.000109 |
| d__Bacteria;p__Fibrobacteres | 8.92E-05 | 0 | 0 | 0 | 0 | 0 | 0.000383 | 0.000208 | 0.000114 | 0.000543 | 0.000194 | 0.000145 |
| d__Bacteria;p__Firmicutes | 0.002127 | 0.000539 | 0.003781 | 0.002159 | 0.001502 | 0.001875 | 0.072922 | 0.06703 | 0.065289 | 0.036508 | 0.034571 | 0.03764 |
| d__Bacteria;p__GAL15 | 0.000149 | 0.00061 | 0.003994 | 0.000208 | 6.01E-05 | 0 | 0 | 0 | 0 | 0 | 0 | 5.45E-05 |
| d__Bacteria;p__Gemmatimonadetes | 0.016836 | 0.012317 | 0.018552 | 0.009509 | 0.008651 | 0.009125 | 0.062454 | 0.084243 | 0.092571 | 0.071367 | 0.063967 | 0.045637 |
| d__Bacteria;p__Hydrogenedentes | 0 | 0 | 0 | 0 | 0 | 0 | 0 | 0 | 0 | 0.000181 | 0 | 0 |
| d__Bacteria;p__Latescibacteria | 0 | 0 | 7.08E-05 | 0 | 0 | 0 | 0.000102 | 0 | 3.25E-05 | 0.000583 | 0.000453 | 0.001636 |
| d__Bacteria;p__Margulisbacteria | 0 | 0 | 0 | 0 | 0 | 0 | 0 | 0 | 0 | 0 | 0 | 0 |
| d__Bacteria;p__Nitrospirae | 0.000297 | 0.000305 | 0.00364 | 0.000291 | 6.01E-05 | 0 | 0.00023 | 0.000139 | 0.000504 | 0.001568 | 0.001531 | 0.001927 |
| d__Bacteria;p__Omnitrophicaeota | 2.97E-05 | 0.000108 | 0.000113 | 0 | 0 | 0 | 0 | 0 | 0 | 0.000101 | 0 | 0 |
| d__Bacteria;p__Patescibacteria | 0.011943 | 0.004327 | 0.009658 | 0.008948 | 0.00841 | 0.006825 | 0.03401 | 0.038538 | 0.035585 | 0.037895 | 0.044772 | 0.03037 |
| d__Bacteria;p__Planctomycetes | 0.00879 | 0.010163 | 0.014261 | 0.004651 | 0.010573 | 0.007175 | 0.002017 | 0.001232 | 0.00221 | 0.002091 | 0.001984 | 0.002508 |
| d__Bacteria;p__Proteobacteria | 0.414206 | 0.428323 | 0.323718 | 0.419973 | 0.51635 | 0.576925 | 0.369565 | 0.35526 | 0.417244 | 0.44354 | 0.446774 | 0.43258 |
| d__Bacteria;p__Rokubacteria | 0.001443 | 0.001688 | 0.004631 | 0 | 0.000541 | 0.0002 | 0 | 0 | 0 | 0 | 0.000108 | 0.000291 |
| d__Bacteria;p__Spirochaetes | 0 | 5.39E-05 | 0 | 0 | 0 | 0 | 0.000102 | 6.94E-05 | 0.000195 | 0.000362 | 0.000151 | 0.000236 |
| d__Bacteria;p__Tenericutes | 0 | 0 | 0 | 0 | 0 | 0 | 0 | 0 | 0 | 0 | 0 | 0 |
| d__Bacteria;p__Verrucomicrobia | 0.01514 | 0.019086 | 0.022177 | 0.008263 | 0.013256 | 0.0052 | 0.005004 | 0.002655 | 0.004923 | 0.006493 | 0.004486 | 0.015612 |
| d__Bacteria;p__WPS-2 | 0.068697 | 0.038119 | 0.098564 | 0.040195 | 0.029236 | 0.016775 | 0.006996 | 0.006299 | 0.004079 | 0.003357 | 0.003364 | 0.002399 |
| d__Bacteria;p__WS2 | 0 | 0.000144 | 0 | 0 | 0 | 0 | 0 | 0.000243 | 0.000325 | 0.000342 | 0.000345 | 0.000782 |
| d__Bacteria;p__unclassified_Bacteria | 0.001145 | 0.000718 | 0.00092 | 0.001578 | 0.000581 | 0.00035 | 0.001941 | 0.001319 | 0.00195 | 0.010916 | 0.002027 | 0.00149 |

**Supplemental table 2b** Soil fungal_OTU_table at phylum

| Taxon | WT_1 | WT_2 | WT_3 | WT_4 | WT_5 | WT_6 | CT_1 | CT_2 | CT_3 | CT_4 | CT_5 | CT_6 |
| --- | --- | --- | --- | --- | --- | --- | --- | --- | --- | --- | --- | --- |
| d__Fungi;p__Ascomycota | 0.23972 | 0.128453 | 0.308743 | 0.807946 | 0.111495 | 0.010199 | 0.378815 | 0.644922 | 0.701159 | 0.586943 | 0.491617 | 0.448597 |
| d__Fungi;p__Basidiomycota | 0.442236 | 0.817035 | 0.472519 | 0.185134 | 0.857439 | 0.989161 | 0.479265 | 0.148512 | 0.07769 | 0.080049 | 0.149511 | 0.024384 |
| d__Fungi;p__Blastocladiomycota | 0 | 0 | 0 | 0 | 0 | 0 | 0 | 0 | 0 | 0 | 0 | 0 |
| d__Fungi;p__Chytridiomycota | 0.004073 | 0 | 0.001544 | 0 | 6.49E-05 | 0 | 0.000187 | 0.000135 | 0 | 0.007499 | 0 | 0.000343 |
| d__Fungi;p__GS01 | 0 | 0 | 0 | 0 | 0 | 0 | 0 | 0 | 0 | 0 | 0 | 0 |
| d__Fungi;p__Glomeromycota | 0.003842 | 0.000829 | 0.001577 | 3.68E-05 | 0 | 0 | 0.000333 | 0.000294 | 0.000234 | 0.000234 | 0.000804 | 0.002507 |
| d__Fungi;p__Kickxellomycota | 0 | 0 | 0.000647 | 0 | 0 | 0 | 0 | 0 | 7.79E-05 | 0 | 0 | 0 |
| d__Fungi;p__Monoblepharomycota | 0 | 0 | 0 | 0 | 0 | 0 | 0 | 0 | 0 | 0 | 0 | 0 |
| d__Fungi;p__Mortierellomycota | 0.120915 | 0.032792 | 0.056506 | 0.001785 | 0.020764 | 0.00024 | 0.04578 | 0.06169 | 0.061564 | 0.155548 | 0.138434 | 0.250654 |
| d__Fungi;p__Mucoromycota | 0.001257 | 0 | 0.000282 | 0.00057 | 0.000649 | 0 | 0.001353 | 0.000587 | 0.000623 | 0.00043 | 0.001468 | 0.000664 |
| d__Fungi;p__Olpidiomycota | 0 | 0 | 0.00073 | 0 | 0 | 0 | 0 | 0 | 0.001617 | 0 | 0 | 0 |
| d__Fungi;p__Rozellomycota | 0.034735 | 0.002829 | 0.000398 | 0.001435 | 0.000552 | 0 | 0.000666 | 0.001174 | 0.000818 | 0.003789 | 0.008243 | 0.005464 |
| d__Fungi;p__Zoopagomycota | 0 | 0 | 0 | 0 | 0 | 0 | 0 | 0 | 0 | 0.003086 | 0.001829 | 0.000214 |
| d__Fungi;p__unclassified_Fungi | 0.10419 | 0.005755 | 0.040321 | 0.00276 | 0.007462 | 0.0004 | 0.035621 | 0.065506 | 0.074632 | 0.08208 | 0.145149 | 0.147204 |
| d__Fungi;p__unidentified | 0.049033 | 0.012307 | 0.116731 | 0.000331 | 0.001574 | 0 | 0.05798 | 0.07718 | 0.081585 | 0.080342 | 0.062945 | 0.11997 |

**Supplemental table 3a** Soil bacterial_OTU_table at class

| Taxon | **WT_1** | **WT_2** | **WT_3** | **WT_4** | **WT_5** | **WT_6** | **CT_1** | **CT_2** | **CT_3** | **CT_4** | **CT_5** | **CT_6** |
| --- | --- | --- | --- | --- | --- | --- | --- | --- | --- | --- | --- | --- |
| d__Bacteria;p__Acidobacteria;c__AT-s3-28 | 0 | 0 | 0.000113 | 0 | 0 | 0 | 0 | 0 | 0 | 0 | 0 | 0 |
| d__Bacteria;p__Acidobacteria;c__Acidobacteriia | 0.235525 | 0.284411 | 0.195967 | 0.271753 | 0.244318 | 0.244075 | 0.132593 | 0.101855 | 0.105733 | 0.119112 | 0.106539 | 0.11701 |
| d__Bacteria;p__Acidobacteria;c__Blastocatellia_(Subgroup_4) | 0.000134 | 0.000503 | 0.003923 | 6.23E-05 | 0.000901 | 0.0002 | 0.000664 | 0.000555 | 0.001917 | 0.006835 | 0.003451 | 0.006307 |
| d__Bacteria;p__Acidobacteria;c__Holophagae | 0.011854 | 0.008277 | 0.022389 | 0.001453 | 0.002823 | 0.000975 | 0.000204 | 0.000486 | 0.001349 | 0.003357 | 0.001682 | 0.004671 |
| d__Bacteria;p__Acidobacteria;c__Subgroup_11 | 0 | 0 | 0 | 0 | 0 | 0 | 0 | 0 | 0 | 0 | 0 | 0 |
| d__Bacteria;p__Acidobacteria;c__Subgroup_15 | 0 | 5.39E-05 | 0.000156 | 6.23E-05 | 0 | 0 | 0.000281 | 0.000121 | 0.000325 | 0.000462 | 0.000194 | 0.000454 |
| d__Bacteria;p__Acidobacteria;c__Subgroup_17 | 0.000134 | 0 | 0.000439 | 0 | 0.00032 | 0 | 0 | 0.000191 | 9.75E-05 | 0.000342 | 0 | 0.000309 |
| d__Bacteria;p__Acidobacteria;c__Subgroup_22 | 0 | 0 | 0 | 0 | 0 | 0 | 0 | 0 | 0 | 0 | 0 | 0 |
| d__Bacteria;p__Acidobacteria;c__Subgroup_25 | 0 | 0 | 0 | 0 | 0 | 0 | 0 | 0 | 0 | 0 | 0 | 7.27E-05 |
| d__Bacteria;p__Acidobacteria;c__Subgroup_5 | 0.004135 | 0.001688 | 0.002351 | 0.001952 | 0.000821 | 0.000275 | 0.000332 | 0.000399 | 0.00039 | 0.001387 | 0 | 0.000309 |
| d__Bacteria;p__Acidobacteria;c__Subgroup_6 | 0.022354 | 0.012982 | 0.031368 | 0.011627 | 0.011674 | 0.004275 | 0.010673 | 0.014038 | 0.021254 | 0.025813 | 0.020596 | 0.028244 |
| d__Bacteria;p__Acidobacteria;c__Thermoanaerobaculia | 0.006485 | 0.002424 | 0.002011 | 0.000664 | 0.000801 | 0.000425 | 0 | 0 | 0.00013 | 6.03E-05 | 0.000259 | 0.0002 |
| d__Bacteria;p__Acidobacteria;c__unclassified_Acidobacteria | 0.00058 | 0.000108 | 7.08E-05 | 0.000166 | 0 | 0 | 0 | 6.94E-05 | 3.25E-05 | 2.01E-05 | 0 | 5.45E-05 |
| d__Bacteria;p__Actinobacteria;c__Acidimicrobiia | 0.025046 | 0.015334 | 0.01487 | 0.059442 | 0.019924 | 0.019125 | 0.020529 | 0.015027 | 0.012788 | 0.018917 | 0.016908 | 0.015376 |
| d__Bacteria;p__Actinobacteria;c__Actinobacteria | 0.039145 | 0.058121 | 0.060951 | 0.069926 | 0.074771 | 0.0516 | 0.09363 | 0.111988 | 0.067239 | 0.053475 | 0.085792 | 0.074826 |
| d__Bacteria;p__Actinobacteria;c__Coriobacteriia | 0 | 0 | 0 | 0 | 0 | 0 | 0 | 0 | 0 | 0 | 0 | 0 |
| d__Bacteria;p__Actinobacteria;c__MB-A2-108 | 0 | 0 | 0.000878 | 0 | 0 | 0 | 0 | 0 | 0 | 0.000181 | 0.000712 | 0.001836 |
| d__Bacteria;p__Actinobacteria;c__Thermoleophilia | 0.028734 | 0.018063 | 0.025788 | 0.037994 | 0.017401 | 0.0098 | 0.063398 | 0.052454 | 0.054678 | 0.053093 | 0.060322 | 0.055343 |
| d__Bacteria;p__Actinobacteria;c__unclassified_Actinobacteria | 0 | 0 | 1.42E-05 | 4.15E-05 | 2.00E-05 | 7.50E-05 | 7.66E-05 | 1.74E-05 | 1.62E-05 | 6.03E-05 | 4.31E-05 | 0 |
| d__Bacteria;p__Armatimonadetes;c__Armatimonadia | 0.000134 | 3.59E-05 | 0.000269 | 0.000498 | 0.00014 | 0 | 0.00023 | 0.000174 | 0.000926 | 0.000402 | 0.00069 | 0.000418 |
| d__Bacteria;p__Armatimonadetes;c__Chthonomonadetes | 0.000625 | 0.000359 | 0.000524 | 0.000561 | 0.00024 | 0.00025 | 0.000204 | 0.000104 | 0.000991 | 0.000623 | 0.000388 | 0.000909 |
| d__Bacteria;p__Armatimonadetes;c__Fimbriimonadia | 0.000297 | 0.000108 | 7.08E-05 | 0.000311 | 0.000581 | 0 | 0.00023 | 6.94E-05 | 0.000942 | 0.000684 | 0 | 0.00129 |
| d__Bacteria;p__Armatimonadetes;c__uncultured | 0.000625 | 0 | 5.66E-05 | 8.30E-05 | 0 | 0 | 7.66E-05 | 0.000174 | 0.000504 | 0.000503 | 0.000345 | 0.001054 |
| d__Bacteria;p__Atribacteria;c__Caldatribacteriia | 0 | 0 | 0 | 0 | 0 | 0 | 5.11E-05 | 0 | 0 | 0 | 0 | 0 |
| d__Bacteria;p__BRC1;c__BRC1 | 0 | 0 | 0 | 0 | 4.00E-05 | 0 | 0 | 5.21E-05 | 0.00026 | 0.000181 | 0 | 0.000545 |
| d__Bacteria;p__Bacteroidetes;c__Bacteroidia | 0.015319 | 0.007739 | 0.01596 | 0.015551 | 0.01672 | 0.0324 | 0.013635 | 0.016102 | 0.016704 | 0.018978 | 0.017879 | 0.016157 |
| d__Bacteria;p__Bacteroidetes;c__Ignavibacteria | 0.00116 | 0.000628 | 0.000297 | 0.000914 | 0.00016 | 5.00E-05 | 0.000638 | 0.000902 | 0.000747 | 0.000764 | 0.000561 | 0.0006 |
| d__Bacteria;p__Chlamydiae;c__Chlamydiae | 0.002231 | 0.003447 | 0.001303 | 0.001474 | 0.000441 | 0.000575 | 0.001506 | 0.001284 | 0.00351 | 0.002473 | 0.000906 | 0.002345 |
| d__Bacteria;p__Chloroflexi;c__AD3 | 0.017461 | 0.030255 | 0.01715 | 0.012955 | 0.003204 | 0.0033 | 0.074276 | 0.092832 | 0.048097 | 0.019158 | 0.026441 | 0.038858 |
| d__Bacteria;p__Chloroflexi;c__Anaerolineae | 0.000134 | 0.000664 | 0.003569 | 4.15E-05 | 0.001442 | 0.000575 | 0.001583 | 0.001562 | 0.003396 | 0.005971 | 0.00386 | 0.006579 |
| d__Bacteria;p__Chloroflexi;c__Chloroflexia | 7.44E-05 | 7.18E-05 | 0.000227 | 0 | 0 | 5.00E-05 | 0.003958 | 0.004026 | 0.003364 | 0.002674 | 0.001941 | 0.001981 |
| d__Bacteria;p__Chloroflexi;c__Dehalococcoidia | 0.000134 | 0.000359 | 0.000113 | 0 | 0.00026 | 0.000275 | 0.001864 | 0.001527 | 0.001495 | 0.002332 | 0.002588 | 0.003253 |
| d__Bacteria;p__Chloroflexi;c__Gitt-GS-136 | 0 | 0 | 0 | 0 | 0 | 0 | 0.000383 | 0.000174 | 0 | 0 | 0.000561 | 0.002345 |
| d__Bacteria;p__Chloroflexi;c__JG30-KF-CM66 | 0.003793 | 0.002639 | 0.003526 | 0.001744 | 0.001061 | 0.00045 | 0.002732 | 0.002707 | 0.002047 | 0.003578 | 0.003429 | 0.00369 |
| d__Bacteria;p__Chloroflexi;c__KD4-96 | 0.003227 | 0.004273 | 0.006882 | 0.001017 | 0.001842 | 0.0005 | 0.007354 | 0.008121 | 0.006353 | 0.021249 | 0.022106 | 0.021465 |
| d__Bacteria;p__Chloroflexi;c__Ktedonobacteria | 0.023603 | 0.019302 | 0.066205 | 0.001765 | 0.004345 | 0.002675 | 0.004111 | 0.003123 | 0.002827 | 0.005508 | 0.002782 | 0.001854 |
| d__Bacteria;p__Chloroflexi;c__OLB14 | 0 | 0 | 0 | 0 | 0 | 0 | 0.000562 | 0.000954 | 0.001186 | 0.000824 | 0.000561 | 0.000981 |
| d__Bacteria;p__Chloroflexi;c__P2-11E | 0 | 0 | 0 | 0 | 0 | 0 | 0 | 0 | 0 | 0 | 4.31E-05 | 0.000127 |
| d__Bacteria;p__Chloroflexi;c__SHA-26 | 0 | 0 | 0 | 0 | 0 | 0 | 0 | 0 | 8.12E-05 | 0 | 0 | 0 |
| d__Bacteria;p__Chloroflexi;c__TK10 | 0.003421 | 0.00255 | 0.008327 | 0.000893 | 0.002103 | 0.00065 | 0.004851 | 0.004824 | 0.003217 | 0.003176 | 0.004184 | 0.005798 |
| d__Bacteria;p__Chloroflexi;c__unclassified_Chloroflexi | 0 | 0 | 0 | 4.15E-05 | 0 | 0 | 0 | 0 | 6.50E-05 | 0.000221 | 4.31E-05 | 0 |
| d__Bacteria;p__Cyanobacteria;c__Melainabacteria | 0.002231 | 0.000844 | 0.001529 | 0.002242 | 0.000421 | 0.00035 | 0.000204 | 0.000833 | 0.000601 | 0.001427 | 0.000453 | 0.000254 |
| d__Bacteria;p__Cyanobacteria;c__Oxyphotobacteria | 0 | 0 | 0 | 0 | 0.00014 | 0 | 0 | 0 | 0 | 0 | 0 | 0 |
| d__Bacteria;p__Cyanobacteria;c__Sericytochromatia | 0.000357 | 7.18E-05 | 0 | 0.000145 | 0.00018 | 0.000175 | 0 | 0.000139 | 0.000146 | 0.000241 | 0.000108 | 0 |
| d__Bacteria;p__Deinococcus-Thermus;c__Deinococci | 8.92E-05 | 0.000126 | 0.000963 | 0 | 0 | 0 | 0 | 0 | 0 | 0 | 6.47E-05 | 0.000109 |
| d__Bacteria;p__Dependentiae;c__Babeliae | 0.004135 | 0.003968 | 0.002195 | 0.003862 | 0.001221 | 0.0008 | 0.002221 | 0.004616 | 0.010546 | 0.008544 | 0.007893 | 0.009996 |
| d__Bacteria;p__Elusimicrobia;c__Elusimicrobia | 0.001755 | 0.001383 | 0.000836 | 0.000623 | 0.000441 | 0.000525 | 0.00046 | 0.000243 | 0.00013 | 6.03E-05 | 0.000237 | 5.45E-05 |
| d__Bacteria;p__Elusimicrobia;c__Lineage_IIa | 0.003376 | 0.002244 | 0.004376 | 0.003675 | 0.001542 | 0.0004 | 0.00046 | 0.000538 | 0.000471 | 0.001206 | 0.000647 | 0.000654 |
| d__Bacteria;p__Elusimicrobia;c__Lineage_IIb | 0.000149 | 0.000215 | 5.66E-05 | 0.000125 | 0 | 0 | 5.11E-05 | 8.68E-05 | 6.50E-05 | 8.04E-05 | 6.47E-05 | 5.45E-05 |
| d__Bacteria;p__Elusimicrobia;c__Lineage_IIc | 0 | 0 | 0 | 0 | 0 | 0 | 0 | 0 | 0 | 0 | 0 | 0 |
| d__Bacteria;p__Entotheonellaeota;c__Entotheonellia | 0 | 0 | 0 | 0 | 0 | 0 | 0 | 0 | 0 | 0 | 0 | 0 |
| d__Bacteria;p__FBP;c__FBP | 0 | 0 | 0 | 0 | 0 | 0 | 5.11E-05 | 0 | 0.000146 | 8.04E-05 | 0 | 0.0002 |
| d__Bacteria;p__FCPU426;c__FCPU426 | 0.000654 | 0.000251 | 0.000198 | 0.000561 | 0.000481 | 0.000725 | 0.000204 | 0.000399 | 0.000211 | 0.000101 | 0 | 0.000109 |
| d__Bacteria;p__Fibrobacteres;c__Fibrobacteria | 8.92E-05 | 0 | 0 | 0 | 0 | 0 | 0.000383 | 0.000208 | 0.000114 | 0.000543 | 0.000194 | 0.000145 |
| d__Bacteria;p__Firmicutes;c__Bacilli | 0.00119 | 0.000144 | 0.001076 | 0.001329 | 0.000641 | 0.001175 | 0.069373 | 0.063664 | 0.061275 | 0.035201 | 0.030754 | 0.035405 |
| d__Bacteria;p__Firmicutes;c__Clostridia | 0.000357 | 0.000305 | 0.002351 | 0.000311 | 0.00032 | 0.00055 | 0.003472 | 0.003297 | 0.003802 | 0.001045 | 0.002976 | 0.001799 |
| d__Bacteria;p__Firmicutes;c__Erysipelotrichia | 0.00058 | 8.98E-05 | 9.91E-05 | 0.000457 | 0.000541 | 0.00015 | 0 | 0 | 0.000211 | 0 | 0.000453 | 0.000436 |
| d__Bacteria;p__Firmicutes;c__Limnochordia | 0 | 0 | 0 | 0 | 0 | 0 | 0 | 0 | 0 | 0 | 8.63E-05 | 0 |
| d__Bacteria;p__Firmicutes;c__Negativicutes | 0 | 0 | 0 | 0 | 0 | 0 | 7.66E-05 | 0 | 0 | 0 | 0 | 0 |
| d__Bacteria;p__Firmicutes;c__unclassified_Firmicutes | 0 | 0 | 0.000255 | 6.23E-05 | 0 | 0 | 0 | 6.94E-05 | 0 | 0.000261 | 0.000302 | 0 |
| d__Bacteria;p__GAL15;c__GAL15 | 0.000149 | 0.00061 | 0.003994 | 0.000208 | 6.01E-05 | 0 | 0 | 0 | 0 | 0 | 0 | 5.45E-05 |
| d__Bacteria;p__Gemmatimonadetes;c__BD2-11_terrestrial_group | 0 | 0 | 0 | 0 | 0 | 0 | 0 | 0.000104 | 0.000325 | 0.000322 | 0 | 7.27E-05 |
| d__Bacteria;p__Gemmatimonadetes;c__Gemmatimonadetes | 0.016836 | 0.012317 | 0.018552 | 0.009509 | 0.008651 | 0.009125 | 0.062454 | 0.084017 | 0.092246 | 0.070925 | 0.063859 | 0.045437 |
| d__Bacteria;p__Gemmatimonadetes;c__Longimicrobia | 0 | 0 | 0 | 0 | 0 | 0 | 0 | 0 | 0 | 0 | 0 | 3.63E-05 |
| d__Bacteria;p__Gemmatimonadetes;c__S0134_terrestrial_group | 0 | 0 | 0 | 0 | 0 | 0 | 0 | 0.000121 | 0 | 0.000121 | 0.000108 | 9.09E-05 |
| d__Bacteria;p__Hydrogenedentes;c__Hydrogenedentia | 0 | 0 | 0 | 0 | 0 | 0 | 0 | 0 | 0 | 0.000181 | 0 | 0 |
| d__Bacteria;p__Latescibacteria;c__Latescibacteria | 0 | 0 | 7.08E-05 | 0 | 0 | 0 | 0.000102 | 0 | 3.25E-05 | 0.000583 | 0.000453 | 0.001636 |
| d__Bacteria;p__Margulisbacteria;c__Margulisbacteria | 0 | 0 | 0 | 0 | 0 | 0 | 0 | 0 | 0 | 0 | 0 | 0 |
| d__Bacteria;p__Nitrospirae;c__Nitrospira | 0.000297 | 0.000305 | 0.00364 | 0.000291 | 6.01E-05 | 0 | 0.00023 | 0.000139 | 0.000504 | 0.001568 | 0.001531 | 0.001927 |
| d__Bacteria;p__Omnitrophicaeota;c__Omnitrophia | 0 | 0 | 0 | 0 | 0 | 0 | 0 | 0 | 0 | 0 | 0 | 0 |
| d__Bacteria;p__Omnitrophicaeota;c__Omnitrophicaeota | 2.97E-05 | 0.000108 | 0.000113 | 0 | 0 | 0 | 0 | 0 | 0 | 0.000101 | 0 | 0 |
| d__Bacteria;p__Patescibacteria;c__ABY1 | 0 | 0 | 0 | 0 | 0 | 0 | 0.000128 | 0 | 0 | 4.02E-05 | 0 | 9.09E-05 |
| d__Bacteria;p__Patescibacteria;c__Berkelbacteria | 0.000164 | 0 | 0 | 4.15E-05 | 4.00E-05 | 7.50E-05 | 0.002834 | 0.002429 | 0.008953 | 0.008021 | 0.001553 | 0.002944 |
| d__Bacteria;p__Patescibacteria;c__Gracilibacteria | 0 | 0.000108 | 0.000113 | 0 | 0 | 0 | 0 | 0.000104 | 0.000617 | 0.000281 | 0.000194 | 3.63E-05 |
| d__Bacteria;p__Patescibacteria;c__Kazania | 0 | 0 | 0 | 0 | 0 | 0 | 0 | 0 | 0.000309 | 0.000322 | 0.000323 | 0 |
| d__Bacteria;p__Patescibacteria;c__Microgenomatia | 0 | 0.000126 | 0.000269 | 0 | 0.00018 | 0 | 0.000153 | 3.47E-05 | 0.000227 | 0.000261 | 0.000216 | 0.000672 |
| d__Bacteria;p__Patescibacteria;c__Parcubacteria | 0.003213 | 0.000844 | 0.003399 | 0.001059 | 0.000741 | 0.001125 | 0.005209 | 0.004251 | 0.005541 | 0.007559 | 0.003213 | 0.003217 |
| d__Bacteria;p__Patescibacteria;c__Saccharimonadia | 0.008448 | 0.003142 | 0.005565 | 0.007765 | 0.007449 | 0.005625 | 0.02515 | 0.031303 | 0.018589 | 0.018777 | 0.03841 | 0.021937 |
| d__Bacteria;p__Patescibacteria;c__WS6_(Dojkabacteria) | 0 | 3.59E-05 | 0.000198 | 0 | 0 | 0 | 0.000255 | 0 | 0.000179 | 0.000161 | 6.47E-05 | 0.000345 |
| d__Bacteria;p__Patescibacteria;c__WWE3 | 0.000119 | 7.18E-05 | 0.000113 | 8.30E-05 | 0 | 0 | 0.000281 | 0.000416 | 0.00117 | 0.002473 | 0.000798 | 0.001127 |
| d__Bacteria;p__Planctomycetes;c__BD7-11 | 0.000446 | 0.000664 | 0.000255 | 0.000415 | 0.000441 | 0.0001 | 0.000434 | 8.68E-05 | 0.000341 | 0.000281 | 0.000302 | 0.000291 |
| d__Bacteria;p__Planctomycetes;c__OM190 | 0 | 0 | 0.000269 | 0 | 0 | 0 | 0 | 0 | 0 | 0 | 0 | 0.000109 |
| d__Bacteria;p__Planctomycetes;c__Phycisphaerae | 4.46E-05 | 5.39E-05 | 4.25E-05 | 0.000166 | 4.00E-05 | 0 | 7.66E-05 | 0.000555 | 8.12E-05 | 0.000523 | 0.000561 | 0 |
| d__Bacteria;p__Planctomycetes;c__Pla4_lineage | 0 | 0 | 0 | 0 | 0 | 0 | 0 | 0 | 0.000179 | 0 | 0 | 0 |
| d__Bacteria;p__Planctomycetes;c__Planctomycetacia | 0.008299 | 0.009444 | 0.013524 | 0.003882 | 0.010032 | 0.007075 | 0.001302 | 0.000468 | 0.001609 | 0.001206 | 0.001035 | 0.002017 |
| d__Bacteria;p__Planctomycetes;c__unclassified_Planctomycetes | 0 | 0 | 0 | 0 | 0 | 0 | 0 | 0 | 0 | 0 | 0 | 0 |
| d__Bacteria;p__Planctomycetes;c__vadinHA49 | 0 | 0 | 0.00017 | 0.000187 | 6.01E-05 | 0 | 0.000204 | 0.000121 | 0 | 8.04E-05 | 8.63E-05 | 9.09E-05 |
| d__Bacteria;p__Proteobacteria;c__Alphaproteobacteria | 0.285542 | 0.324667 | 0.233268 | 0.296751 | 0.394301 | 0.45385 | 0.235567 | 0.201645 | 0.223668 | 0.26846 | 0.275341 | 0.234492 |
| d__Bacteria;p__Proteobacteria;c__Deltaproteobacteria | 0.035323 | 0.023755 | 0.037174 | 0.043745 | 0.042632 | 0.021775 | 0.026759 | 0.027624 | 0.028923 | 0.029813 | 0.020618 | 0.021465 |
| d__Bacteria;p__Proteobacteria;c__Gammaproteobacteria | 0.093297 | 0.079811 | 0.053276 | 0.079373 | 0.079297 | 0.1011 | 0.106932 | 0.125835 | 0.164506 | 0.145126 | 0.150686 | 0.176551 |
| d__Bacteria;p__Proteobacteria;c__unclassified_Proteobacteria | 4.46E-05 | 8.98E-05 | 0 | 0.000104 | 0.00012 | 0.0002 | 0.000306 | 0.000156 | 0.000146 | 0.000141 | 0.000129 | 7.27E-05 |
| d__Bacteria;p__Rokubacteria;c__NC10 | 0.001443 | 0.001688 | 0.004631 | 0 | 0.000541 | 0.0002 | 0 | 0 | 0 | 0 | 0.000108 | 0.000291 |
| d__Bacteria;p__Spirochaetes;c__Leptospirae | 0 | 0 | 0 | 0 | 0 | 0 | 0.000102 | 6.94E-05 | 8.12E-05 | 0.000362 | 0.000151 | 3.63E-05 |
| d__Bacteria;p__Spirochaetes;c__Spirochaetia | 0 | 5.39E-05 | 0 | 0 | 0 | 0 | 0 | 0 | 0.000114 | 0 | 0 | 0.0002 |
| d__Bacteria;p__Tenericutes;c__Mollicutes | 0 | 0 | 0 | 0 | 0 | 0 | 0 | 0 | 0 | 0 | 0 | 0 |
| d__Bacteria;p__Verrucomicrobia;c__Verrucomicrobiae | 0.01514 | 0.019086 | 0.022177 | 0.008263 | 0.013256 | 0.0052 | 0.005004 | 0.002655 | 0.004923 | 0.006493 | 0.004486 | 0.015612 |
| d__Bacteria;p__WPS-2;c__WPS-2 | 0.068697 | 0.038119 | 0.098564 | 0.040195 | 0.029236 | 0.016775 | 0.006996 | 0.006299 | 0.004079 | 0.003357 | 0.003364 | 0.002399 |
| d__Bacteria;p__WS2;c__WS2 | 0 | 0.000144 | 0 | 0 | 0 | 0 | 0 | 0.000243 | 0.000325 | 0.000342 | 0.000345 | 0.000782 |
| d__Bacteria;p__unclassified_Bacteria;c__unclassified_Bacteria | 0.001145 | 0.000718 | 0.00092 | 0.001578 | 0.000581 | 0.00035 | 0.001941 | 0.001319 | 0.00195 | 0.010916 | 0.002027 | 0.00149 |

**Supplemental table 3b** Soil fungal_OTU_table at class

| Taxon | **WT_1** | **WT_2** | **WT_3** | **WT_4** | **WT_5** | **WT_6** | **CT_1** | **CT_2** | **CT_3** | **CT_4** | **CT_5** | **CT_6** |
| --- | --- | --- | --- | --- | --- | --- | --- | --- | --- | --- | --- | --- |
| d__Fungi;p__Ascomycota;c__Archaeorhizomycetes | 0.022921 | 0.090637 | 0.006408 | 0.796463 | 0.088946 | 0.007909 | 0.000208 | 0.000135 | 0 | 0.000234 | 0.001146 | 0 |
| d__Fungi;p__Ascomycota;c__Dothideomycetes | 0.001906 | 8.13E-05 | 0.008317 | 0.000865 | 0.000357 | 9.61E-05 | 0.050298 | 0.020932 | 0.040101 | 0.013631 | 0.047264 | 0.013692 |
| d__Fungi;p__Ascomycota;c__Eurotiomycetes | 0.002282 | 0.001024 | 0.01323 | 0.000258 | 0.000795 | 0.000112 | 0.0295 | 0.02599 | 0.093232 | 0.079678 | 0.027763 | 0.020206 |
| d__Fungi;p__Ascomycota;c__GS35 | 0 | 0 | 0 | 0 | 0 | 0.000144 | 0 | 0 | 0 | 0 | 0 | 0 |
| d__Fungi;p__Ascomycota;c__GS37 | 0 | 0 | 0 | 0 | 0 | 0 | 0 | 0 | 0 | 0 | 0 | 0 |
| d__Fungi;p__Ascomycota;c__Geoglossomycetes | 0 | 0 | 0 | 0 | 0 | 0 | 0 | 0 | 0 | 0 | 0 | 0 |
| d__Fungi;p__Ascomycota;c__Laboulbeniomycetes | 0 | 0 | 0 | 0 | 0 | 0 | 0 | 0 | 0 | 0 | 0 | 0 |
| d__Fungi;p__Ascomycota;c__Lecanoromycetes | 0 | 0 | 0 | 0 | 0 | 0 | 0 | 0 | 0 | 0 | 0.000181 | 0 |
| d__Fungi;p__Ascomycota;c__Leotiomycetes | 0.185488 | 0.019688 | 0.240783 | 0.008631 | 0.014032 | 0.00048 | 0.05265 | 0.059116 | 0.088207 | 0.124497 | 0.127799 | 0.184358 |
| d__Fungi;p__Ascomycota;c__Orbiliomycetes | 0 | 0 | 0.004465 | 0 | 0 | 0 | 0.002061 | 0 | 0 | 0 | 0.000322 | 0 |
| d__Fungi;p__Ascomycota;c__Pezizomycetes | 0 | 0 | 0.001394 | 7.36E-05 | 0 | 0 | 0 | 0 | 3.90E-05 | 0.000156 | 0.000342 | 0 |
| d__Fungi;p__Ascomycota;c__Saccharomycetes | 0.001387 | 0.001301 | 0.002971 | 0.000828 | 0.001379 | 0.001329 | 0.002852 | 0.002077 | 0.002746 | 0.001621 | 0.002292 | 0.001671 |
| d__Fungi;p__Ascomycota;c__Sordariomycetes | 0.024394 | 0.003187 | 0.029863 | 0.000791 | 0.005434 | 3.20E-05 | 0.240767 | 0.536671 | 0.475061 | 0.35574 | 0.277854 | 0.227791 |
| d__Fungi;p__Ascomycota;c__Taphrinomycetes | 0 | 0 | 0 | 0 | 0 | 0 | 0 | 0 | 0 | 0 | 0 | 0 |
| d__Fungi;p__Ascomycota;c__Xylonomycetes | 0 | 0 | 0 | 3.68E-05 | 0 | 0 | 0 | 0 | 0 | 0 | 0 | 0 |
| d__Fungi;p__Ascomycota;c__unclassified_Ascomycota | 0.001343 | 0 | 0.001311 | 0 | 0.000552 | 9.61E-05 | 0.000479 | 0 | 0.001772 | 0.011385 | 0.006654 | 0.000879 |
| d__Fungi;p__Ascomycota;c__unidentified | 0 | 0.012535 | 0 | 0 | 0 | 0 | 0 | 0 | 0 | 0 | 0 | 0 |
| d__Fungi;p__Basidiomycota;c__Agaricomycetes | 0.430379 | 0.813962 | 0.461712 | 0.182447 | 0.846992 | 0.987159 | 0.462672 | 0.13607 | 0.028825 | 0.062629 | 0.109344 | 0.015042 |
| d__Fungi;p__Basidiomycota;c__Cystobasidiomycetes | 0 | 0 | 0 | 0 | 0 | 0 | 0 | 0 | 0 | 0 | 0.000342 | 0 |
| d__Fungi;p__Basidiomycota;c__Geminibasidiomycetes | 0 | 0 | 0 | 0 | 0.00013 | 0 | 0 | 0 | 0 | 0 | 0 | 0 |
| d__Fungi;p__Basidiomycota;c__Malasseziomycetes | 0 | 0 | 0.002606 | 0 | 0 | 0 | 0 | 0 | 0.000428 | 0 | 0 | 0 |
| d__Fungi;p__Basidiomycota;c__Microbotryomycetes | 0.003813 | 8.13E-05 | 0 | 0.000221 | 0.00146 | 0.000464 | 0.000167 | 0.001377 | 0 | 0 | 0.000744 | 0 |
| d__Fungi;p__Basidiomycota;c__Pucciniomycetes | 0 | 0.000163 | 0 | 0 | 0 | 0 | 0 | 0 | 0 | 0 | 0 | 0 |
| d__Fungi;p__Basidiomycota;c__Tremellomycetes | 0.003943 | 0.002683 | 0.004349 | 0.002356 | 0.006294 | 0.001537 | 0.016259 | 0.010658 | 0.048437 | 0.01742 | 0.037916 | 0.009214 |
| d__Fungi;p__Basidiomycota;c__Ustilaginomycetes | 0 | 0 | 0 | 0 | 0 | 0 | 0 | 0 | 0 | 0 | 0 | 0 |
| d__Fungi;p__Basidiomycota;c__Wallemiomycetes | 0 | 0 | 0 | 0 | 0 | 0 | 0 | 0.000406 | 0 | 0 | 0 | 0 |
| d__Fungi;p__Basidiomycota;c__unclassified_Basidiomycota | 0.004102 | 0.000146 | 0.003851 | 0.00011 | 0.002563 | 0 | 0.000167 | 0 | 0 | 0 | 0.001166 | 0.000129 |
| d__Fungi;p__Blastocladiomycota;c__Blastocladiomycetes | 0 | 0 | 0 | 0 | 0 | 0 | 0 | 0 | 0 | 0 | 0 | 0 |
| d__Fungi;p__Chytridiomycota;c__Chytridiomycetes | 0 | 0 | 0 | 0 | 0 | 0 | 0.000187 | 0.000135 | 0 | 0 | 0 | 0 |
| d__Fungi;p__Chytridiomycota;c__Rhizophlyctidomycetes | 0 | 0 | 0 | 0 | 0 | 0 | 0 | 0 | 0 | 0.007499 | 0 | 0 |
| d__Fungi;p__Chytridiomycota;c__Rhizophydiomycetes | 0.000289 | 0 | 0.000714 | 0 | 0 | 0 | 0 | 0 | 0 | 0 | 0 | 0 |
| d__Fungi;p__Chytridiomycota;c__unclassified_Chytridiomycota | 0 | 0 | 0 | 0 | 0 | 0 | 0 | 0 | 0 | 0 | 0 | 0 |
| d__Fungi;p__Chytridiomycota;c__unidentified | 0.003784 | 0 | 0.00083 | 0 | 6.49E-05 | 0 | 0 | 0 | 0 | 0 | 0 | 0.000343 |
| d__Fungi;p__GS01;c__unidentified | 0 | 0 | 0 | 0 | 0 | 0 | 0 | 0 | 0 | 0 | 0 | 0 |
| d__Fungi;p__Glomeromycota;c__Archaeosporomycetes | 0.000635 | 0.000829 | 0.00083 | 0 | 0 | 0 | 0 | 0 | 0 | 0 | 0.000181 | 0.000236 |
| d__Fungi;p__Glomeromycota;c__Glomeromycetes | 0.00091 | 0 | 0 | 3.68E-05 | 0 | 0 | 0 | 0 | 0 | 0.000234 | 0 | 0 |
| d__Fungi;p__Glomeromycota;c__Paraglomeromycetes | 0 | 0 | 0 | 0 | 0 | 0 | 0.000333 | 0.000294 | 0.000234 | 0 | 0.000322 | 0.000921 |
| d__Fungi;p__Glomeromycota;c__unclassified_Glomeromycota | 0.002239 | 0 | 0.000747 | 0 | 0 | 0 | 0 | 0 | 0 | 0 | 0.000302 | 0.00135 |
| d__Fungi;p__Glomeromycota;c__unidentified | 5.78E-05 | 0 | 0 | 0 | 0 | 0 | 0 | 0 | 0 | 0 | 0 | 0 |
| d__Fungi;p__Kickxellomycota;c__GS19 | 0 | 0 | 0 | 0 | 0 | 0 | 0 | 0 | 0 | 0 | 0 | 0 |
| d__Fungi;p__Kickxellomycota;c__Kickxellomycetes | 0 | 0 | 0.000647 | 0 | 0 | 0 | 0 | 0 | 7.79E-05 | 0 | 0 | 0 |
| d__Fungi;p__Monoblepharomycota;c__Monoblepharidomycetes | 0 | 0 | 0 | 0 | 0 | 0 | 0 | 0 | 0 | 0 | 0 | 0 |
| d__Fungi;p__Monoblepharomycota;c__Sanchytriomycetes | 0 | 0 | 0 | 0 | 0 | 0 | 0 | 0 | 0 | 0 | 0 | 0 |
| d__Fungi;p__Mortierellomycota;c__Mortierellomycetes | 0.120915 | 0.032792 | 0.056506 | 0.001785 | 0.020764 | 0.00024 | 0.04578 | 0.06169 | 0.061564 | 0.155548 | 0.138434 | 0.250654 |
| d__Fungi;p__Mucoromycota;c__Mucoromycetes | 0.000188 | 0 | 0.000282 | 0.000258 | 9.73E-05 | 0 | 0.000229 | 6.77E-05 | 0.000623 | 0.00043 | 0.000744 | 0.000664 |
| d__Fungi;p__Mucoromycota;c__Umbelopsidomycetes | 0.001069 | 0 | 0 | 0.000313 | 0.000552 | 0 | 0.001124 | 0.000519 | 0 | 0 | 0.000724 | 0 |
| d__Fungi;p__Olpidiomycota;c__Olpidiomycetes | 0 | 0 | 0.00073 | 0 | 0 | 0 | 0 | 0 | 0.001617 | 0 | 0 | 0 |
| d__Fungi;p__Rozellomycota;c__Rozellomycotina_cls_Incertae_sedis | 0 | 0.001398 | 0.000166 | 0 | 0.000146 | 0 | 0 | 0.0007 | 0 | 0.001953 | 0.000905 | 0.005464 |
| d__Fungi;p__Rozellomycota;c__unidentified | 0.034735 | 0.001431 | 0.000232 | 0.001435 | 0.000406 | 0 | 0.000666 | 0.000474 | 0.000818 | 0.001836 | 0.007338 | 0 |
| d__Fungi;p__Zoopagomycota;c__Zoopagomycetes | 0 | 0 | 0 | 0 | 0 | 0 | 0 | 0 | 0 | 0.003086 | 0.001829 | 0.000214 |
| d__Fungi;p__unclassified_Fungi;c__unclassified_Fungi | 0.10419 | 0.005755 | 0.040321 | 0.00276 | 0.007462 | 0.0004 | 0.035621 | 0.065506 | 0.074632 | 0.08208 | 0.145149 | 0.147204 |
| d__Fungi;p__unidentified;c__unidentified | 0.049033 | 0.012307 | 0.116731 | 0.000331 | 0.001574 | 0 | 0.05798 | 0.07718 | 0.081585 | 0.080342 | 0.062945 | 0.11997 |

**upplemental table 4a** Soil bacterial_OTU_table at order

| Taxon | **WT_1** | **WT_2** | **WT_3** | **WT_4** | **WT_5** | **WT_6** | **CT_1** | **CT_2** | **CT_3** | **CT_4** | **CT_5** | **CT_6** |
| --- | --- | --- | --- | --- | --- | --- | --- | --- | --- | --- | --- | --- |
| d__Bacteria;p__Acidobacteria;c__AT-s3-28;o__AT-s3-28 | 0 | 0 | 0.000113 | 0 | 0 | 0 | 0 | 0 | 0 | 0 | 0 | 0 |
| d__Bacteria;p__Acidobacteria;c__Acidobacteriia;o__Acidobacteriales | 0.087928 | 0.091572 | 0.076316 | 0.095214 | 0.095116 | 0.15115 | 0.082344 | 0.053929 | 0.050453 | 0.041212 | 0.052773 | 0.064048 |
| d__Bacteria;p__Acidobacteria;c__Acidobacteriia;o__Solibacterales | 0.067121 | 0.088771 | 0.068598 | 0.096107 | 0.08168 | 0.0495 | 0.03401 | 0.03479 | 0.040655 | 0.060471 | 0.042745 | 0.041911 |
| d__Bacteria;p__Acidobacteria;c__Acidobacteriia;o__Subgroup_12 | 0.00113 | 0.000503 | 0.000496 | 0.001163 | 0.00016 | 0.0001 | 0 | 0 | 0 | 0 | 0 | 0 |
| d__Bacteria;p__Acidobacteria;c__Acidobacteriia;o__Subgroup_13 | 0.002097 | 0.002532 | 0.001827 | 0.000893 | 0.00036 | 0.00035 | 0.000664 | 0.000798 | 0.000877 | 0.000945 | 0.000367 | 0.000727 |
| d__Bacteria;p__Acidobacteria;c__Acidobacteriia;o__Subgroup_2 | 0.077249 | 0.101016 | 0.04873 | 0.078376 | 0.066902 | 0.042975 | 0.015575 | 0.012337 | 0.013747 | 0.016485 | 0.010654 | 0.010323 |
| d__Bacteria;p__Acidobacteria;c__Acidobacteriia;o__unclassified_Acidobacteriia | 0 | 1.80E-05 | 0 | 0 | 0.0001 | 0 | 0 | 0 | 0 | 0 | 0 | 0 |
| d__Bacteria;p__Acidobacteria;c__Blastocatellia_(Subgroup_4);o__11-24 | 4.46E-05 | 0 | 5.66E-05 | 6.23E-05 | 0.000661 | 5.00E-05 | 5.11E-05 | 0.000278 | 0.000617 | 0.000804 | 0.000151 | 0.000164 |
| d__Bacteria;p__Acidobacteria;c__Blastocatellia_(Subgroup_4);o__Blastocatellales | 0 | 3.59E-05 | 0.000212 | 0 | 8.01E-05 | 0.00015 | 0.000613 | 0.000278 | 0.0013 | 0.005629 | 0.002782 | 0.005034 |
| d__Bacteria;p__Acidobacteria;c__Blastocatellia_(Subgroup_4);o__DS-100 | 0 | 0 | 0 | 0 | 0 | 0 | 0 | 0 | 0 | 0 | 0 | 0 |
| d__Bacteria;p__Acidobacteria;c__Blastocatellia_(Subgroup_4);o__Elev-16S-573 | 0 | 0 | 0 | 0 | 6.01E-05 | 0 | 0 | 0 | 0 | 0.000121 | 0 | 9.09E-05 |
| d__Bacteria;p__Acidobacteria;c__Blastocatellia_(Subgroup_4);o__Pyrinomonadales | 8.92E-05 | 0.000467 | 0.003654 | 0 | 0.0001 | 0 | 0 | 0 | 0 | 0.000281 | 0.000518 | 0.000963 |
| d__Bacteria;p__Acidobacteria;c__Blastocatellia_(Subgroup_4);o__unclassified_Blastocatellia_(Subgroup_4) | 0 | 0 | 0 | 0 | 0 | 0 | 0 | 0 | 0 | 0 | 0 | 5.45E-05 |
| d__Bacteria;p__Acidobacteria;c__Holophagae;o__Holophagales | 0 | 0 | 0 | 0 | 0 | 0 | 0.000204 | 0.000312 | 0.000829 | 0.000844 | 0.000216 | 0.000182 |
| d__Bacteria;p__Acidobacteria;c__Holophagae;o__Subgroup_7 | 0.011854 | 0.008277 | 0.022389 | 0.001453 | 0.002823 | 0.000975 | 0 | 0.000174 | 0.00052 | 0.002513 | 0.001467 | 0.004489 |
| d__Bacteria;p__Acidobacteria;c__Subgroup_11;o__Subgroup_11 | 0 | 0 | 0 | 0 | 0 | 0 | 0 | 0 | 0 | 0 | 0 | 0 |
| d__Bacteria;p__Acidobacteria;c__Subgroup_15;o__Subgroup_15 | 0 | 5.39E-05 | 0.000156 | 6.23E-05 | 0 | 0 | 0.000281 | 0.000121 | 0.000325 | 0.000462 | 0.000194 | 0.000454 |
| d__Bacteria;p__Acidobacteria;c__Subgroup_17;o__Subgroup_17 | 0.000134 | 0 | 0.000439 | 0 | 0.00032 | 0 | 0 | 0.000191 | 9.75E-05 | 0.000342 | 0 | 0.000309 |
| d__Bacteria;p__Acidobacteria;c__Subgroup_22;o__Subgroup_22 | 0 | 0 | 0 | 0 | 0 | 0 | 0 | 0 | 0 | 0 | 0 | 0 |
| d__Bacteria;p__Acidobacteria;c__Subgroup_25;o__Subgroup_25 | 0 | 0 | 0 | 0 | 0 | 0 | 0 | 0 | 0 | 0 | 0 | 7.27E-05 |
| d__Bacteria;p__Acidobacteria;c__Subgroup_5;o__Subgroup_5 | 0.004135 | 0.001688 | 0.002351 | 0.001952 | 0.000821 | 0.000275 | 0.000332 | 0.000399 | 0.00039 | 0.001387 | 0 | 0.000309 |
| d__Bacteria;p__Acidobacteria;c__Subgroup_6;o__Subgroup_6 | 0.022265 | 0.012856 | 0.031283 | 0.01144 | 0.011634 | 0.00405 | 0.01052 | 0.013881 | 0.021205 | 0.025571 | 0.019928 | 0.028044 |
| d__Bacteria;p__Acidobacteria;c__Subgroup_6;o__Unknown_Order | 0 | 0 | 0 | 0 | 0 | 0 | 5.11E-05 | 0 | 0 | 0 | 0.000129 | 5.45E-05 |
| d__Bacteria;p__Acidobacteria;c__Subgroup_6;o__unclassified_Subgroup_6 | 8.92E-05 | 0.000126 | 8.50E-05 | 0.000187 | 4.00E-05 | 0.000225 | 0.000102 | 0.000156 | 4.87E-05 | 0.000241 | 0.000539 | 0.000145 |
| d__Bacteria;p__Acidobacteria;c__Thermoanaerobaculia;o__Thermoanaerobaculales | 0.006485 | 0.002424 | 0.002011 | 0.000664 | 0.000801 | 0.000425 | 0 | 0 | 0.00013 | 6.03E-05 | 0.000259 | 0.0002 |
| d__Bacteria;p__Acidobacteria;c__unclassified_Acidobacteria;o__unclassified_Acidobacteria | 0.00058 | 0.000108 | 7.08E-05 | 0.000166 | 0 | 0 | 0 | 6.94E-05 | 3.25E-05 | 2.01E-05 | 0 | 5.45E-05 |
| d__Bacteria;p__Actinobacteria;c__Acidimicrobiia;o__Acidimicrobiales | 0 | 0 | 0 | 0 | 0 | 0 | 0 | 0 | 0 | 0 | 0 | 0 |
| d__Bacteria;p__Actinobacteria;c__Acidimicrobiia;o__IMCC26256 | 0.011318 | 0.006248 | 0.010083 | 0.018686 | 0.009932 | 0.00475 | 0.018435 | 0.012476 | 0.010903 | 0.016746 | 0.014967 | 0.013867 |
| d__Bacteria;p__Actinobacteria;c__Acidimicrobiia;o__Microtrichales | 0.000297 | 0 | 0.000538 | 0.000249 | 0.000701 | 0.000225 | 0 | 0.000538 | 0.001056 | 0.000824 | 0.000453 | 0.000818 |
| d__Bacteria;p__Actinobacteria;c__Acidimicrobiia;o__unclassified_Acidimicrobiia | 0.000699 | 0.000539 | 0.00017 | 0.001682 | 0.00038 | 0 | 0 | 0.000278 | 4.87E-05 | 0 | 0.000216 | 0 |
| d__Bacteria;p__Actinobacteria;c__Acidimicrobiia;o__uncultured | 0.012731 | 0.008547 | 0.004079 | 0.038825 | 0.008911 | 0.01415 | 0.002094 | 0.001735 | 0.00078 | 0.001347 | 0.001272 | 0.000691 |
| d__Bacteria;p__Actinobacteria;c__Actinobacteria;o__Bifidobacteriales | 0 | 0 | 0 | 6.23E-05 | 0 | 5.00E-05 | 0 | 0 | 6.50E-05 | 0 | 0 | 0 |
| d__Bacteria;p__Actinobacteria;c__Actinobacteria;o__Catenulisporales | 0.000193 | 0.000144 | 0.000142 | 0.000664 | 0.001582 | 0.000875 | 0.001404 | 0.001301 | 0.001251 | 0.000482 | 0.001768 | 0.000291 |
| d__Bacteria;p__Actinobacteria;c__Actinobacteria;o__Corynebacteriales | 0.000387 | 0.001329 | 0.001204 | 0.002201 | 0.007589 | 0.003 | 0.000664 | 0.000382 | 0.000552 | 0.000523 | 0.00427 | 0.001509 |
| d__Bacteria;p__Actinobacteria;c__Actinobacteria;o__Frankiales | 0.037137 | 0.055625 | 0.051562 | 0.060895 | 0.053766 | 0.0415 | 0.01966 | 0.017543 | 0.014478 | 0.012323 | 0.017296 | 0.00976 |
| d__Bacteria;p__Actinobacteria;c__Actinobacteria;o__Kineosporiales | 0 | 0 | 0.000184 | 0 | 6.01E-05 | 0.00025 | 0.001149 | 0.000607 | 0.000341 | 0.000583 | 0.001229 | 0.000418 |
| d__Bacteria;p__Actinobacteria;c__Actinobacteria;o__Micrococcales | 0.000565 | 0.000251 | 0.006854 | 0.000706 | 0.000781 | 0.001925 | 0.028239 | 0.056445 | 0.014413 | 0.013087 | 0.022408 | 0.044438 |
| d__Bacteria;p__Actinobacteria;c__Actinobacteria;o__Micromonosporales | 0 | 0 | 0.000198 | 0 | 0.002683 | 0.001875 | 0.004341 | 0.005483 | 0.00364 | 0.002091 | 0.00509 | 0.002435 |
| d__Bacteria;p__Actinobacteria;c__Actinobacteria;o__PeM15 | 0 | 0 | 0 | 0 | 0 | 0 | 0 | 0 | 0 | 0 | 0 | 0 |
| d__Bacteria;p__Actinobacteria;c__Actinobacteria;o__Propionibacteriales | 0 | 0 | 9.91E-05 | 0 | 0.00024 | 2.50E-05 | 0.003192 | 0.002742 | 0.00325 | 0.004041 | 0.007074 | 0.00309 |
| d__Bacteria;p__Actinobacteria;c__Actinobacteria;o__Pseudonocardiales | 0.000387 | 0.000305 | 0.000368 | 0.004734 | 0.001221 | 0.0006 | 0.004392 | 0.002603 | 0.00351 | 0.003176 | 0.006944 | 0.003817 |
| d__Bacteria;p__Actinobacteria;c__Actinobacteria;o__Streptomycetales | 0.000342 | 0.000359 | 0.000142 | 0.000457 | 0.006528 | 0.000725 | 0.002553 | 0.001891 | 0.0013 | 0.000925 | 0.00619 | 0.002017 |
| d__Bacteria;p__Actinobacteria;c__Actinobacteria;o__Streptosporangiales | 0 | 7.18E-05 | 0.000142 | 0.000125 | 0.00014 | 0.000475 | 0.024946 | 0.021117 | 0.021871 | 0.014756 | 0.01184 | 0.006525 |
| d__Bacteria;p__Actinobacteria;c__Actinobacteria;o__unclassified_Actinobacteria | 0.000134 | 3.59E-05 | 5.66E-05 | 8.30E-05 | 0.00018 | 0.0003 | 0.003089 | 0.001874 | 0.002567 | 0.001488 | 0.001682 | 0.000527 |
| d__Bacteria;p__Actinobacteria;c__Coriobacteriia;o__Coriobacteriales | 0 | 0 | 0 | 0 | 0 | 0 | 0 | 0 | 0 | 0 | 0 | 0 |
| d__Bacteria;p__Actinobacteria;c__MB-A2-108;o__MB-A2-108 | 0 | 0 | 0.000878 | 0 | 0 | 0 | 0 | 0 | 0 | 0.000181 | 0.000712 | 0.001836 |
| d__Bacteria;p__Actinobacteria;c__Thermoleophilia;o__Gaiellales | 0.01694 | 0.008565 | 0.015167 | 0.009613 | 0.007549 | 0.00325 | 0.047542 | 0.044195 | 0.047187 | 0.044388 | 0.04915 | 0.0464 |
| d__Bacteria;p__Actinobacteria;c__Thermoleophilia;o__Solirubrobacterales | 0.011794 | 0.009498 | 0.010621 | 0.028382 | 0.009852 | 0.00655 | 0.015856 | 0.008259 | 0.007491 | 0.008705 | 0.011085 | 0.008797 |
| d__Bacteria;p__Actinobacteria;c__Thermoleophilia;o__unclassified_Thermoleophilia | 0 | 0 | 0 | 0 | 0 | 0 | 0 | 0 | 0 | 0 | 0 | 0 |
| d__Bacteria;p__Actinobacteria;c__Thermoleophilia;o__uncultured | 0 | 0 | 0 | 0 | 0 | 0 | 0 | 0 | 0 | 0 | 8.63E-05 | 0.000145 |
| d__Bacteria;p__Actinobacteria;c__unclassified_Actinobacteria;o__unclassified_Actinobacteria | 0 | 0 | 1.42E-05 | 4.15E-05 | 2.00E-05 | 7.50E-05 | 7.66E-05 | 1.74E-05 | 1.62E-05 | 6.03E-05 | 4.31E-05 | 0 |
| d__Bacteria;p__Armatimonadetes;c__Armatimonadia;o__Armatimonadales | 0.000134 | 3.59E-05 | 0.000269 | 0.000498 | 0.00014 | 0 | 0.00023 | 0.000174 | 0.000926 | 0.000402 | 0.00069 | 0.000418 |
| d__Bacteria;p__Armatimonadetes;c__Chthonomonadetes;o__Chthonomonadales | 0.000625 | 0.000359 | 0.000524 | 0.000561 | 0.00024 | 0.00025 | 0.000204 | 0.000104 | 0.000991 | 0.000623 | 0.000388 | 0.000909 |
| d__Bacteria;p__Armatimonadetes;c__Fimbriimonadia;o__Fimbriimonadales | 0.000297 | 0.000108 | 7.08E-05 | 0.000311 | 0.000581 | 0 | 0.00023 | 6.94E-05 | 0.000942 | 0.000684 | 0 | 0.00129 |
| d__Bacteria;p__Armatimonadetes;c__uncultured;o__uncultured | 0.000625 | 0 | 5.66E-05 | 8.30E-05 | 0 | 0 | 7.66E-05 | 0.000174 | 0.000504 | 0.000503 | 0.000345 | 0.001054 |
| d__Bacteria;p__Atribacteria;c__Caldatribacteriia;o__Caldatribacteriales | 0 | 0 | 0 | 0 | 0 | 0 | 5.11E-05 | 0 | 0 | 0 | 0 | 0 |
| d__Bacteria;p__BRC1;c__BRC1;o__BRC1 | 0 | 0 | 0 | 0 | 4.00E-05 | 0 | 0 | 5.21E-05 | 0.00026 | 0.000181 | 0 | 0.000545 |
| d__Bacteria;p__Bacteroidetes;c__Bacteroidia;o__Bacteroidales | 0.000104 | 0 | 0.001076 | 0 | 0 | 0.000175 | 0.000204 | 0.000121 | 0.000179 | 0.000101 | 0.000151 | 0.000291 |
| d__Bacteria;p__Bacteroidetes;c__Bacteroidia;o__Bacteroidetes_VC2.1_Bac22 | 0 | 0 | 0 | 0 | 0 | 0 | 0 | 0 | 0 | 0 | 0 | 0 |
| d__Bacteria;p__Bacteroidetes;c__Bacteroidia;o__Chitinophagales | 0.009504 | 0.005063 | 0.011414 | 0.009717 | 0.010813 | 0.006925 | 0.008962 | 0.011296 | 0.010302 | 0.010454 | 0.010589 | 0.011905 |
| d__Bacteria;p__Bacteroidetes;c__Bacteroidia;o__Cytophagales | 0 | 0.000126 | 0.000581 | 0.000457 | 0.000981 | 0.0004 | 0.000281 | 0.000694 | 0.001592 | 0.002352 | 0.000647 | 0.000727 |
| d__Bacteria;p__Bacteroidetes;c__Bacteroidia;o__Flavobacteriales | 0 | 0.000108 | 5.66E-05 | 0 | 0.00012 | 7.50E-05 | 0 | 0.000104 | 0.000179 | 0 | 0.00028 | 0 |
| d__Bacteria;p__Bacteroidetes;c__Bacteroidia;o__SM1A07 | 0 | 0 | 0 | 0 | 0 | 0 | 0 | 0 | 6.50E-05 | 4.02E-05 | 4.31E-05 | 0 |
| d__Bacteria;p__Bacteroidetes;c__Bacteroidia;o__Sphingobacteriales | 0.005711 | 0.002442 | 0.002832 | 0.005336 | 0.004806 | 0.024825 | 0.004187 | 0.003887 | 0.004387 | 0.006031 | 0.006125 | 0.003235 |
| d__Bacteria;p__Bacteroidetes;c__Bacteroidia;o__unclassified_Bacteroidia | 0 | 0 | 0 | 4.15E-05 | 0 | 0 | 0 | 0 | 0 | 0 | 4.31E-05 | 0 |
| d__Bacteria;p__Bacteroidetes;c__Ignavibacteria;o__Kryptoniales | 0 | 0 | 0 | 0 | 0 | 0 | 0 | 0 | 0 | 4.02E-05 | 0 | 0 |
| d__Bacteria;p__Bacteroidetes;c__Ignavibacteria;o__OPB56 | 0.00116 | 0.000628 | 0.000198 | 0.000914 | 0.00016 | 5.00E-05 | 0.000638 | 0.000902 | 0.000747 | 0.000322 | 0.000388 | 0.000291 |
| d__Bacteria;p__Bacteroidetes;c__Ignavibacteria;o__SJA-28 | 0 | 0 | 9.91E-05 | 0 | 0 | 0 | 0 | 0 | 0 | 0.000402 | 0.000173 | 0.000309 |
| d__Bacteria;p__Chlamydiae;c__Chlamydiae;o__Chlamydiales | 0.002231 | 0.003447 | 0.001303 | 0.001474 | 0.000441 | 0.000575 | 0.001506 | 0.001284 | 0.00351 | 0.002473 | 0.000906 | 0.002345 |
| d__Bacteria;p__Chloroflexi;c__AD3;o__AD3 | 0.017461 | 0.030255 | 0.01715 | 0.012955 | 0.003204 | 0.0033 | 0.074276 | 0.092832 | 0.048097 | 0.019158 | 0.026441 | 0.038858 |
| d__Bacteria;p__Chloroflexi;c__Anaerolineae;o__Anaerolineae | 0 | 0 | 0 | 0 | 0 | 0 | 0 | 0 | 0 | 0 | 0 | 0 |
| d__Bacteria;p__Chloroflexi;c__Anaerolineae;o__Anaerolineales | 0 | 0.000413 | 0.000552 | 0 | 0.00024 | 5.00E-05 | 7.66E-05 | 0.000399 | 0.000666 | 0.001367 | 0.000863 | 0.000963 |
| d__Bacteria;p__Chloroflexi;c__Anaerolineae;o__Caldilineales | 0 | 0 | 0 | 0 | 0 | 0 | 0 | 0 | 8.12E-05 | 0 | 0 | 0.0002 |
| d__Bacteria;p__Chloroflexi;c__Anaerolineae;o__RBG-13-54-9 | 0 | 0 | 0.000651 | 0 | 0 | 0 | 0 | 5.21E-05 | 0 | 0 | 0.000884 | 0.000872 |
| d__Bacteria;p__Chloroflexi;c__Anaerolineae;o__SBR1031 | 0.000134 | 0.000251 | 0.002365 | 4.15E-05 | 0.001201 | 0.000525 | 0.001506 | 0.001111 | 0.002649 | 0.004604 | 0.002114 | 0.004544 |
| d__Bacteria;p__Chloroflexi;c__Anaerolineae;o__unclassified_Anaerolineae | 0 | 0 | 0 | 0 | 0 | 0 | 0 | 0 | 0 | 0 | 0 | 0 |
| d__Bacteria;p__Chloroflexi;c__Chloroflexia;o__Chloroflexales | 0 | 0 | 0 | 0 | 0 | 5.00E-05 | 7.66E-05 | 0 | 0 | 6.03E-05 | 0 | 0 |
| d__Bacteria;p__Chloroflexi;c__Chloroflexia;o__Elev-1554 | 7.44E-05 | 7.18E-05 | 0.000227 | 0 | 0 | 0 | 0.000485 | 0.000226 | 0.000341 | 0.000221 | 0 | 0.000109 |
| d__Bacteria;p__Chloroflexi;c__Chloroflexia;o__Kallotenuales | 0 | 0 | 0 | 0 | 0 | 0 | 0 | 0 | 0 | 0 | 0 | 5.45E-05 |
| d__Bacteria;p__Chloroflexi;c__Chloroflexia;o__Thermomicrobiales | 0 | 0 | 0 | 0 | 0 | 0 | 0.003396 | 0.0038 | 0.003022 | 0.002392 | 0.001941 | 0.001817 |
| d__Bacteria;p__Chloroflexi;c__Dehalococcoidia;o__S085 | 0.000134 | 0.000323 | 0.000113 | 0 | 0.00026 | 0.000275 | 0.001864 | 0.001527 | 0.001495 | 0.002231 | 0.002588 | 0.003253 |
| d__Bacteria;p__Chloroflexi;c__Dehalococcoidia;o__SAR202_clade | 0 | 3.59E-05 | 0 | 0 | 0 | 0 | 0 | 0 | 0 | 0.000101 | 0 | 0 |
| d__Bacteria;p__Chloroflexi;c__Dehalococcoidia;o__unclassified_Dehalococcoidia | 0 | 0 | 0 | 0 | 0 | 0 | 0 | 0 | 0 | 0 | 0 | 0 |
| d__Bacteria;p__Chloroflexi;c__Gitt-GS-136;o__Gitt-GS-136 | 0 | 0 | 0 | 0 | 0 | 0 | 0.000383 | 0.000174 | 0 | 0 | 0.000561 | 0.002345 |
| d__Bacteria;p__Chloroflexi;c__JG30-KF-CM66;o__JG30-KF-CM66 | 0.003793 | 0.002639 | 0.003526 | 0.001744 | 0.001061 | 0.00045 | 0.002732 | 0.002707 | 0.002047 | 0.003578 | 0.003429 | 0.00369 |
| d__Bacteria;p__Chloroflexi;c__KD4-96;o__KD4-96 | 0.003227 | 0.004273 | 0.006882 | 0.001017 | 0.001842 | 0.0005 | 0.007354 | 0.008121 | 0.006353 | 0.021249 | 0.022106 | 0.021465 |
| d__Bacteria;p__Chloroflexi;c__Ktedonobacteria;o__B10-SB3A | 0 | 0 | 0 | 0 | 0 | 0 | 0 | 8.68E-05 | 0 | 0 | 0 | 0 |
| d__Bacteria;p__Chloroflexi;c__Ktedonobacteria;o__B12-WMSP1 | 4.46E-05 | 0 | 0.000411 | 0.000228 | 0 | 0 | 0 | 8.68E-05 | 0 | 0 | 0 | 0 |
| d__Bacteria;p__Chloroflexi;c__Ktedonobacteria;o__C0119 | 0 | 0 | 0 | 0 | 0 | 0 | 0.000613 | 0.000295 | 0.00026 | 0.000744 | 0.001035 | 0.0004 |
| d__Bacteria;p__Chloroflexi;c__Ktedonobacteria;o__Ktedonobacterales | 0.023558 | 0.019302 | 0.065794 | 0.001536 | 0.004345 | 0.002675 | 0.003498 | 0.002655 | 0.002567 | 0.004764 | 0.001747 | 0.001454 |
| d__Bacteria;p__Chloroflexi;c__OLB14;o__OLB14 | 0 | 0 | 0 | 0 | 0 | 0 | 0.000562 | 0.000954 | 0.001186 | 0.000824 | 0.000561 | 0.000981 |
| d__Bacteria;p__Chloroflexi;c__P2-11E;o__P2-11E | 0 | 0 | 0 | 0 | 0 | 0 | 0 | 0 | 0 | 0 | 4.31E-05 | 0.000127 |
| d__Bacteria;p__Chloroflexi;c__SHA-26;o__SHA-26 | 0 | 0 | 0 | 0 | 0 | 0 | 0 | 0 | 8.12E-05 | 0 | 0 | 0 |
| d__Bacteria;p__Chloroflexi;c__TK10;o__TK10 | 0.003421 | 0.00255 | 0.008327 | 0.000893 | 0.002103 | 0.00065 | 0.004851 | 0.004824 | 0.003217 | 0.003176 | 0.004184 | 0.005798 |
| d__Bacteria;p__Chloroflexi;c__unclassified_Chloroflexi;o__unclassified_Chloroflexi | 0 | 0 | 0 | 4.15E-05 | 0 | 0 | 0 | 0 | 6.50E-05 | 0.000221 | 4.31E-05 | 0 |
| d__Bacteria;p__Cyanobacteria;c__Melainabacteria;o__Obscuribacterales | 0.002186 | 0.000844 | 0.001459 | 0.002159 | 0.00036 | 0.00035 | 0.000204 | 0.000833 | 0.000504 | 0.000764 | 0.000453 | 0.000254 |
| d__Bacteria;p__Cyanobacteria;c__Melainabacteria;o__Vampirovibrionales | 4.46E-05 | 0 | 7.08E-05 | 8.30E-05 | 6.01E-05 | 0 | 0 | 0 | 9.75E-05 | 0.000663 | 0 | 0 |
| d__Bacteria;p__Cyanobacteria;c__Oxyphotobacteria;o__Nostocales | 0 | 0 | 0 | 0 | 0 | 0 | 0 | 0 | 0 | 0 | 0 | 0 |
| d__Bacteria;p__Cyanobacteria;c__Oxyphotobacteria;o__Synechococcales | 0 | 0 | 0 | 0 | 0.00014 | 0 | 0 | 0 | 0 | 0 | 0 | 0 |
| d__Bacteria;p__Cyanobacteria;c__Sericytochromatia;o__Sericytochromatia | 0.000357 | 7.18E-05 | 0 | 0.000145 | 0.00018 | 0.000175 | 0 | 0.000139 | 0.000146 | 0.000241 | 0.000108 | 0 |
| d__Bacteria;p__Deinococcus-Thermus;c__Deinococci;o__Thermales | 8.92E-05 | 0.000126 | 0.000963 | 0 | 0 | 0 | 0 | 0 | 0 | 0 | 6.47E-05 | 0.000109 |
| d__Bacteria;p__Dependentiae;c__Babeliae;o__Babeliales | 0.004135 | 0.003968 | 0.002195 | 0.003862 | 0.001221 | 0.0008 | 0.002221 | 0.004616 | 0.010546 | 0.008544 | 0.007893 | 0.009996 |
| d__Bacteria;p__Elusimicrobia;c__Elusimicrobia;o__Elusimicrobia | 2.97E-05 | 0.000359 | 0.000184 | 8.30E-05 | 0 | 0 | 0 | 0 | 0 | 0 | 0 | 0 |
| d__Bacteria;p__Elusimicrobia;c__Elusimicrobia;o__FCPU453 | 0.000283 | 0.000162 | 0.000156 | 0 | 0 | 0 | 0 | 0 | 0 | 0 | 0 | 0 |
| d__Bacteria;p__Elusimicrobia;c__Elusimicrobia;o__Lineage_IV | 0.001443 | 0.000862 | 0.000496 | 0.00054 | 0.000441 | 0.000525 | 0.00046 | 0.000243 | 4.87E-05 | 6.03E-05 | 0.000237 | 5.45E-05 |
| d__Bacteria;p__Elusimicrobia;c__Elusimicrobia;o__MVP-88 | 0 | 0 | 0 | 0 | 0 | 0 | 0 | 0 | 4.87E-05 | 0 | 0 | 0 |
| d__Bacteria;p__Elusimicrobia;c__Elusimicrobia;o__unclassified_Elusimicrobia | 0 | 0 | 0 | 0 | 0 | 0 | 0 | 0 | 3.25E-05 | 0 | 0 | 0 |
| d__Bacteria;p__Elusimicrobia;c__Lineage_IIa;o__Lineage_IIa | 0.003376 | 0.002244 | 0.004376 | 0.003675 | 0.001542 | 0.0004 | 0.00046 | 0.000538 | 0.000471 | 0.001206 | 0.000647 | 0.000654 |
| d__Bacteria;p__Elusimicrobia;c__Lineage_IIb;o__Lineage_IIb | 0.000149 | 0.000215 | 5.66E-05 | 0.000125 | 0 | 0 | 5.11E-05 | 8.68E-05 | 6.50E-05 | 8.04E-05 | 6.47E-05 | 5.45E-05 |
| d__Bacteria;p__Elusimicrobia;c__Lineage_IIc;o__Lineage_IIc | 0 | 0 | 0 | 0 | 0 | 0 | 0 | 0 | 0 | 0 | 0 | 0 |
| d__Bacteria;p__Entotheonellaeota;c__Entotheonellia;o__Entotheonellales | 0 | 0 | 0 | 0 | 0 | 0 | 0 | 0 | 0 | 0 | 0 | 0 |
| d__Bacteria;p__FBP;c__FBP;o__FBP | 0 | 0 | 0 | 0 | 0 | 0 | 5.11E-05 | 0 | 0.000146 | 8.04E-05 | 0 | 0.0002 |
| d__Bacteria;p__FCPU426;c__FCPU426;o__FCPU426 | 0.000654 | 0.000251 | 0.000198 | 0.000561 | 0.000481 | 0.000725 | 0.000204 | 0.000399 | 0.000211 | 0.000101 | 0 | 0.000109 |
| d__Bacteria;p__Fibrobacteres;c__Fibrobacteria;o__Fibrobacterales | 8.92E-05 | 0 | 0 | 0 | 0 | 0 | 0.000383 | 0.000208 | 0.000114 | 0.000543 | 0.000194 | 0.000145 |
| d__Bacteria;p__Firmicutes;c__Bacilli;o__Bacillales | 0.000833 | 5.39E-05 | 0.000793 | 0.001329 | 0.000641 | 0.000825 | 0.069169 | 0.062154 | 0.058968 | 0.034095 | 0.030366 | 0.034587 |
| d__Bacteria;p__Firmicutes;c__Bacilli;o__Lactobacillales | 0.000357 | 8.98E-05 | 0.000283 | 0 | 0 | 0.000275 | 0 | 0.001353 | 0.002161 | 0.001065 | 0.000173 | 0.000782 |
| d__Bacteria;p__Firmicutes;c__Bacilli;o__unclassified_Bacilli | 0 | 0 | 0 | 0 | 0 | 7.50E-05 | 0.000204 | 0.000156 | 0.000146 | 4.02E-05 | 0.000216 | 3.63E-05 |
| d__Bacteria;p__Firmicutes;c__Clostridia;o__Clostridiales | 0.000357 | 0.000269 | 0.002351 | 0.000311 | 0.00032 | 0.00055 | 0.003472 | 0.003175 | 0.003721 | 0.001045 | 0.002976 | 0.001799 |
| d__Bacteria;p__Firmicutes;c__Clostridia;o__DTU014 | 0 | 0 | 0 | 0 | 0 | 0 | 0 | 0 | 8.12E-05 | 0 | 0 | 0 |
| d__Bacteria;p__Firmicutes;c__Clostridia;o__unclassified_Clostridia | 0 | 3.59E-05 | 0 | 0 | 0 | 0 | 0 | 0.000121 | 0 | 0 | 0 | 0 |
| d__Bacteria;p__Firmicutes;c__Erysipelotrichia;o__Erysipelotrichales | 0.00058 | 8.98E-05 | 9.91E-05 | 0.000457 | 0.000541 | 0.00015 | 0 | 0 | 0.000211 | 0 | 0.000453 | 0.000436 |
| d__Bacteria;p__Firmicutes;c__Limnochordia;o__Limnochordales | 0 | 0 | 0 | 0 | 0 | 0 | 0 | 0 | 0 | 0 | 8.63E-05 | 0 |
| d__Bacteria;p__Firmicutes;c__Negativicutes;o__Selenomonadales | 0 | 0 | 0 | 0 | 0 | 0 | 7.66E-05 | 0 | 0 | 0 | 0 | 0 |
| d__Bacteria;p__Firmicutes;c__unclassified_Firmicutes;o__unclassified_Firmicutes | 0 | 0 | 0.000255 | 6.23E-05 | 0 | 0 | 0 | 6.94E-05 | 0 | 0.000261 | 0.000302 | 0 |
| d__Bacteria;p__GAL15;c__GAL15;o__GAL15 | 0.000149 | 0.00061 | 0.003994 | 0.000208 | 6.01E-05 | 0 | 0 | 0 | 0 | 0 | 0 | 5.45E-05 |
| d__Bacteria;p__Gemmatimonadetes;c__BD2-11_terrestrial_group;o__BD2-11_terrestrial_group | 0 | 0 | 0 | 0 | 0 | 0 | 0 | 0.000104 | 0.000325 | 0.000322 | 0 | 7.27E-05 |
| d__Bacteria;p__Gemmatimonadetes;c__Gemmatimonadetes;o__Gemmatimonadales | 0.016836 | 0.012317 | 0.018552 | 0.009509 | 0.008651 | 0.009125 | 0.062454 | 0.084017 | 0.092246 | 0.070925 | 0.063859 | 0.045437 |
| d__Bacteria;p__Gemmatimonadetes;c__Longimicrobia;o__Longimicrobiales | 0 | 0 | 0 | 0 | 0 | 0 | 0 | 0 | 0 | 0 | 0 | 3.63E-05 |
| d__Bacteria;p__Gemmatimonadetes;c__S0134_terrestrial_group;o__S0134_terrestrial_group | 0 | 0 | 0 | 0 | 0 | 0 | 0 | 0.000121 | 0 | 0.000121 | 0.000108 | 9.09E-05 |
| d__Bacteria;p__Hydrogenedentes;c__Hydrogenedentia;o__Hydrogenedentiales | 0 | 0 | 0 | 0 | 0 | 0 | 0 | 0 | 0 | 0.000181 | 0 | 0 |
| d__Bacteria;p__Latescibacteria;c__Latescibacteria;o__Latescibacteria | 0 | 0 | 7.08E-05 | 0 | 0 | 0 | 0.000102 | 0 | 3.25E-05 | 0.000583 | 0.000453 | 0.001636 |
| d__Bacteria;p__Margulisbacteria;c__Margulisbacteria;o__Margulisbacteria | 0 | 0 | 0 | 0 | 0 | 0 | 0 | 0 | 0 | 0 | 0 | 0 |
| d__Bacteria;p__Nitrospirae;c__Nitrospira;o__Nitrospirales | 0.000297 | 0.000305 | 0.00364 | 0.000291 | 6.01E-05 | 0 | 0.00023 | 0.000139 | 0.000504 | 0.001568 | 0.001531 | 0.001927 |
| d__Bacteria;p__Omnitrophicaeota;c__Omnitrophia;o__Omnitrophales | 0 | 0 | 0 | 0 | 0 | 0 | 0 | 0 | 0 | 0 | 0 | 0 |
| d__Bacteria;p__Omnitrophicaeota;c__Omnitrophicaeota;o__Omnitrophicaeota | 2.97E-05 | 0.000108 | 0.000113 | 0 | 0 | 0 | 0 | 0 | 0 | 0.000101 | 0 | 0 |
| d__Bacteria;p__Patescibacteria;c__ABY1;o__Candidatus_Kerfeldbacteria | 0 | 0 | 0 | 0 | 0 | 0 | 0 | 0 | 0 | 0 | 0 | 0 |
| d__Bacteria;p__Patescibacteria;c__ABY1;o__Candidatus_Magasanikbacteria | 0 | 0 | 0 | 0 | 0 | 0 | 0.000128 | 0 | 0 | 0 | 0 | 9.09E-05 |
| d__Bacteria;p__Patescibacteria;c__ABY1;o__unclassified_ABY1 | 0 | 0 | 0 | 0 | 0 | 0 | 0 | 0 | 0 | 4.02E-05 | 0 | 0 |
| d__Bacteria;p__Patescibacteria;c__Berkelbacteria;o__Berkelbacteria | 0.000164 | 0 | 0 | 4.15E-05 | 4.00E-05 | 7.50E-05 | 0.002834 | 0.002429 | 0.008953 | 0.008021 | 0.001553 | 0.002944 |
| d__Bacteria;p__Patescibacteria;c__Gracilibacteria;o__Candidatus_Peregrinibacteria | 0 | 0.000108 | 0 | 0 | 0 | 0 | 0 | 0 | 0 | 0 | 0.000129 | 0 |
| d__Bacteria;p__Patescibacteria;c__Gracilibacteria;o__Candidatus_Peribacteria | 0 | 0 | 0.000113 | 0 | 0 | 0 | 0 | 0.000104 | 4.87E-05 | 0.000281 | 6.47E-05 | 3.63E-05 |
| d__Bacteria;p__Patescibacteria;c__Gracilibacteria;o__Gracilibacteria | 0 | 0 | 0 | 0 | 0 | 0 | 0 | 0 | 0.000569 | 0 | 0 | 0 |
| d__Bacteria;p__Patescibacteria;c__Kazania;o__Kazania | 0 | 0 | 0 | 0 | 0 | 0 | 0 | 0 | 0.000309 | 0.000322 | 0.000323 | 0 |
| d__Bacteria;p__Patescibacteria;c__Microgenomatia;o__Candidatus_Daviesbacteria | 0 | 0 | 0 | 0 | 0 | 0 | 0 | 0 | 6.50E-05 | 0 | 0 | 0 |
| d__Bacteria;p__Patescibacteria;c__Microgenomatia;o__Candidatus_Levybacteria | 0 | 0.000126 | 0.000212 | 0 | 0.00018 | 0 | 0 | 0 | 0.00013 | 0 | 0.000129 | 0.000182 |
| d__Bacteria;p__Patescibacteria;c__Microgenomatia;o__Candidatus_Pacebacteria | 0 | 0 | 0 | 0 | 0 | 0 | 0 | 0 | 0 | 0.000221 | 4.31E-05 | 0.000236 |
| d__Bacteria;p__Patescibacteria;c__Microgenomatia;o__Candidatus_Woesebacteria | 0 | 0 | 5.66E-05 | 0 | 0 | 0 | 0.000153 | 3.47E-05 | 3.25E-05 | 4.02E-05 | 4.31E-05 | 0.000254 |
| d__Bacteria;p__Patescibacteria;c__Microgenomatia;o__Candidatus_Woykebacteria | 0 | 0 | 0 | 0 | 0 | 0 | 0 | 0 | 0 | 0 | 0 | 0 |
| d__Bacteria;p__Patescibacteria;c__Microgenomatia;o__Microgenomatia | 0 | 0 | 0 | 0 | 0 | 0 | 0 | 0 | 0 | 0 | 0 | 0 |
| d__Bacteria;p__Patescibacteria;c__Microgenomatia;o__unclassified_Microgenomatia | 0 | 0 | 0 | 0 | 0 | 0 | 0 | 0 | 0 | 0 | 0 | 0 |
| d__Bacteria;p__Patescibacteria;c__Parcubacteria;o__Candidatus_Adlerbacteria | 4.46E-05 | 0 | 0 | 0 | 0 | 0 | 0 | 0 | 0 | 0 | 0 | 0 |
| d__Bacteria;p__Patescibacteria;c__Parcubacteria;o__Candidatus_Azambacteria | 0.000535 | 0 | 8.50E-05 | 0 | 0 | 0 | 0 | 0 | 0 | 0 | 0 | 3.63E-05 |
| d__Bacteria;p__Patescibacteria;c__Parcubacteria;o__Candidatus_Jorgensenbacteria | 0.000744 | 0.000503 | 0.001175 | 0.000768 | 0.00014 | 0.000375 | 0.001277 | 0.001249 | 0.001462 | 0.001749 | 0.001359 | 0.001018 |
| d__Bacteria;p__Patescibacteria;c__Parcubacteria;o__Candidatus_Kaiserbacteria | 0.000178 | 0 | 0.000241 | 0 | 0.00012 | 0.000325 | 0.000945 | 0.000364 | 0.000731 | 0.000885 | 0.000173 | 0.000345 |
| d__Bacteria;p__Patescibacteria;c__Parcubacteria;o__Candidatus_Liptonbacteria | 0 | 0 | 0 | 0 | 0 | 0 | 0 | 0 | 0 | 0 | 0 | 0 |
| d__Bacteria;p__Patescibacteria;c__Parcubacteria;o__Candidatus_Moranbacteria | 0 | 0 | 0 | 0 | 0 | 0 | 0 | 0 | 0 | 0 | 0 | 7.27E-05 |
| d__Bacteria;p__Patescibacteria;c__Parcubacteria;o__Candidatus_Nomurabacteria | 2.97E-05 | 0 | 0 | 0 | 0 | 5.00E-05 | 0 | 0 | 0 | 0 | 0 | 0 |
| d__Bacteria;p__Patescibacteria;c__Parcubacteria;o__Candidatus_Staskawiczbacteria | 0 | 0 | 7.08E-05 | 0 | 0 | 0 | 0 | 0 | 4.87E-05 | 6.03E-05 | 0 | 0 |
| d__Bacteria;p__Patescibacteria;c__Parcubacteria;o__Candidatus_Yanofskybacteria | 0.000149 | 0 | 0 | 0 | 0 | 0 | 0.000102 | 0.000104 | 9.75E-05 | 0.000221 | 0 | 7.27E-05 |
| d__Bacteria;p__Patescibacteria;c__Parcubacteria;o__Candidatus_Zambryskibacteria | 0 | 0 | 0 | 0 | 0 | 0 | 0 | 0 | 0 | 0 | 0 | 0 |
| d__Bacteria;p__Patescibacteria;c__Parcubacteria;o__Parcubacteria | 0.001532 | 0.000341 | 0.001827 | 0.000291 | 0.000481 | 0.000375 | 0.002885 | 0.002499 | 0.003201 | 0.004362 | 0.001682 | 0.00149 |
| d__Bacteria;p__Patescibacteria;c__Parcubacteria;o__unclassified_Parcubacteria | 0 | 0 | 0 | 0 | 0 | 0 | 0 | 3.47E-05 | 0 | 0.000281 | 0 | 0.000182 |
| d__Bacteria;p__Patescibacteria;c__Saccharimonadia;o__Saccharimonadales | 0.008448 | 0.003142 | 0.005565 | 0.007765 | 0.007449 | 0.005625 | 0.02515 | 0.031303 | 0.018589 | 0.018777 | 0.03841 | 0.021937 |
| d__Bacteria;p__Patescibacteria;c__WS6_(Dojkabacteria);o__WS6_(Dojkabacteria) | 0 | 3.59E-05 | 0.000198 | 0 | 0 | 0 | 0.000255 | 0 | 0.000179 | 0.000161 | 6.47E-05 | 0.000345 |
| d__Bacteria;p__Patescibacteria;c__WWE3;o__WWE3 | 0.000119 | 7.18E-05 | 0.000113 | 8.30E-05 | 0 | 0 | 0.000281 | 0.000416 | 0.00117 | 0.002473 | 0.000798 | 0.001127 |
| d__Bacteria;p__Planctomycetes;c__BD7-11;o__BD7-11 | 0.000446 | 0.000664 | 0.000255 | 0.000415 | 0.000441 | 0.0001 | 0.000434 | 8.68E-05 | 0.000341 | 0.000281 | 0.000302 | 0.000291 |
| d__Bacteria;p__Planctomycetes;c__OM190;o__OM190 | 0 | 0 | 0.000269 | 0 | 0 | 0 | 0 | 0 | 0 | 0 | 0 | 0.000109 |
| d__Bacteria;p__Planctomycetes;c__Phycisphaerae;o__Phycisphaerales | 4.46E-05 | 5.39E-05 | 4.25E-05 | 6.23E-05 | 4.00E-05 | 0 | 0 | 0.000382 | 8.12E-05 | 0.000523 | 8.63E-05 | 0 |
| d__Bacteria;p__Planctomycetes;c__Phycisphaerae;o__Tepidisphaerales | 0 | 0 | 0 | 0.000104 | 0 | 0 | 7.66E-05 | 0.000174 | 0 | 0 | 0.000474 | 0 |
| d__Bacteria;p__Planctomycetes;c__Pla4_lineage;o__Pla4_lineage | 0 | 0 | 0 | 0 | 0 | 0 | 0 | 0 | 0.000179 | 0 | 0 | 0 |
| d__Bacteria;p__Planctomycetes;c__Planctomycetacia;o__Gemmatales | 0.006187 | 0.006518 | 0.009432 | 0.002886 | 0.005527 | 0.002725 | 0.000409 | 5.21E-05 | 0.000731 | 0.000543 | 0.000539 | 0.001072 |
| d__Bacteria;p__Planctomycetes;c__Planctomycetacia;o__Isosphaerales | 0.002112 | 0.002927 | 0.004093 | 0.000997 | 0.004505 | 0.00435 | 0.000894 | 0.000416 | 0.000877 | 0.000663 | 0.000496 | 0.000945 |
| d__Bacteria;p__Planctomycetes;c__Planctomycetacia;o__Pirellulales | 0 | 0 | 0 | 0 | 0 | 0 | 0 | 0 | 0 | 0 | 0 | 0 |
| d__Bacteria;p__Planctomycetes;c__Planctomycetacia;o__Planctomycetales | 0 | 0 | 0 | 0 | 0 | 0 | 0 | 0 | 0 | 0 | 0 | 0 |
| d__Bacteria;p__Planctomycetes;c__unclassified_Planctomycetes;o__unclassified_Planctomycetes | 0 | 0 | 0 | 0 | 0 | 0 | 0 | 0 | 0 | 0 | 0 | 0 |
| d__Bacteria;p__Planctomycetes;c__vadinHA49;o__vadinHA49 | 0 | 0 | 0.00017 | 0.000187 | 6.01E-05 | 0 | 0.000204 | 0.000121 | 0 | 8.04E-05 | 8.63E-05 | 9.09E-05 |
| d__Bacteria;p__Proteobacteria;c__Alphaproteobacteria;o__Acetobacterales | 0.012746 | 0.006536 | 0.009672 | 0.02234 | 0.039949 | 0.100575 | 0.008528 | 0.005934 | 0.006386 | 0.006132 | 0.005543 | 0.003053 |
| d__Bacteria;p__Proteobacteria;c__Alphaproteobacteria;o__Azospirillales | 0.000164 | 0.000162 | 0.000297 | 0.000789 | 0.001262 | 0.00155 | 0.000511 | 0.000278 | 0.000487 | 0.000784 | 0.000992 | 0.000782 |
| d__Bacteria;p__Proteobacteria;c__Alphaproteobacteria;o__Caedibacterales | 0 | 0 | 0 | 0.000104 | 0 | 0 | 0 | 0 | 0 | 0 | 0 | 0 |
| d__Bacteria;p__Proteobacteria;c__Alphaproteobacteria;o__Caulobacterales | 0.0152 | 0.010109 | 0.005381 | 0.010817 | 0.010052 | 0.0149 | 0.009498 | 0.009561 | 0.011651 | 0.011519 | 0.013673 | 0.013013 |
| d__Bacteria;p__Proteobacteria;c__Alphaproteobacteria;o__Dongiales | 0 | 0 | 0 | 4.15E-05 | 0 | 0.000625 | 0.001992 | 0.002325 | 0.003071 | 0.004986 | 0.002178 | 0.00229 |
| d__Bacteria;p__Proteobacteria;c__Alphaproteobacteria;o__Elsterales | 0.033999 | 0.063508 | 0.052468 | 0.031268 | 0.026853 | 0.02255 | 0.022495 | 0.022037 | 0.023805 | 0.031904 | 0.02588 | 0.023827 |
| d__Bacteria;p__Proteobacteria;c__Alphaproteobacteria;o__Holosporales | 0.000208 | 0.000467 | 0.000212 | 0.000415 | 0 | 0 | 0.000766 | 0.000312 | 0.000877 | 0.00201 | 0.000733 | 0.001454 |
| d__Bacteria;p__Proteobacteria;c__Alphaproteobacteria;o__Micavibrionales | 0 | 0 | 0 | 0 | 6.01E-05 | 0.00015 | 0.000562 | 0.00033 | 0.00013 | 0.000342 | 0.000216 | 0.000418 |
| d__Bacteria;p__Proteobacteria;c__Alphaproteobacteria;o__Micropepsales | 0.021476 | 0.01352 | 0.009474 | 0.020139 | 0.01654 | 0.02225 | 0.035236 | 0.030348 | 0.038559 | 0.038056 | 0.035197 | 0.02559 |
| d__Bacteria;p__Proteobacteria;c__Alphaproteobacteria;o__Paracaedibacterales | 0.000342 | 0 | 0.000142 | 0.000291 | 0 | 5.00E-05 | 0 | 3.47E-05 | 0.000114 | 0.000382 | 0.000453 | 3.63E-05 |
| d__Bacteria;p__Proteobacteria;c__Alphaproteobacteria;o__Parvibaculales | 0 | 0 | 0 | 0 | 0 | 0 | 0 | 0 | 0.000114 | 4.02E-05 | 8.63E-05 | 0 |
| d__Bacteria;p__Proteobacteria;c__Alphaproteobacteria;o__Puniceispirillales | 0 | 0 | 0 | 0 | 0 | 0 | 0 | 0 | 0 | 0 | 0 | 0 |
| d__Bacteria;p__Proteobacteria;c__Alphaproteobacteria;o__Reyranellales | 0.004402 | 0.004327 | 0.007761 | 0.002948 | 0.020245 | 0.008425 | 0.008656 | 0.008225 | 0.007101 | 0.009006 | 0.009403 | 0.005562 |
| d__Bacteria;p__Proteobacteria;c__Alphaproteobacteria;o__Rhizobiales | 0.186832 | 0.221478 | 0.140864 | 0.197965 | 0.266645 | 0.270575 | 0.128482 | 0.098593 | 0.098031 | 0.130491 | 0.156746 | 0.131859 |
| d__Bacteria;p__Proteobacteria;c__Alphaproteobacteria;o__Rhodobacterales | 0.000104 | 0 | 0 | 0 | 0 | 0 | 0 | 0 | 0 | 0 | 0 | 0 |
| d__Bacteria;p__Proteobacteria;c__Alphaproteobacteria;o__Rhodospirillales | 0.002439 | 0.000898 | 0.000878 | 0.005315 | 0.002543 | 0.004025 | 0.005873 | 0.005639 | 0.007783 | 0.007137 | 0.003558 | 0.002272 |
| d__Bacteria;p__Proteobacteria;c__Alphaproteobacteria;o__Rickettsiales | 0.001487 | 0.000467 | 0.000637 | 0.001619 | 0.001562 | 0 | 0.002221 | 0.002481 | 0.004566 | 0.004262 | 0.003925 | 0.002472 |
| d__Bacteria;p__Proteobacteria;c__Alphaproteobacteria;o__Sphingomonadales | 0.000193 | 0 | 0.000368 | 0.000145 | 0.000661 | 0.00175 | 0.006307 | 0.009023 | 0.014559 | 0.013067 | 0.01184 | 0.015976 |
| d__Bacteria;p__Proteobacteria;c__Alphaproteobacteria;o__Thalassobaculales | 0 | 0 | 0 | 0 | 0 | 0 | 0 | 0 | 0 | 0 | 0 | 0 |
| d__Bacteria;p__Proteobacteria;c__Alphaproteobacteria;o__Tistrellales | 0 | 0 | 0 | 0 | 0 | 0 | 0 | 0 | 4.87E-05 | 0 | 0 | 0 |
| d__Bacteria;p__Proteobacteria;c__Alphaproteobacteria;o__unclassified_Alphaproteobacteria | 0.004938 | 0.002873 | 0.002761 | 0.002014 | 0.006127 | 0.005225 | 0.001123 | 0.001631 | 0.001674 | 0.001186 | 0.00207 | 0.002036 |
| d__Bacteria;p__Proteobacteria;c__Alphaproteobacteria;o__uncultured | 0.001011 | 0.000323 | 0.002351 | 0.00054 | 0.001802 | 0.0012 | 0.003319 | 0.004893 | 0.004712 | 0.007157 | 0.002847 | 0.003853 |
| d__Bacteria;p__Proteobacteria;c__Deltaproteobacteria;o__Bdellovibrionales | 0.001041 | 0.000898 | 0.00085 | 0.002325 | 0.001422 | 0.001075 | 0.001277 | 0.0017 | 0.0013 | 0.000623 | 0.001208 | 0.000527 |
| d__Bacteria;p__Proteobacteria;c__Deltaproteobacteria;o__Desulfarculales | 0.000149 | 0.000323 | 0.001926 | 0 | 0.002583 | 0.000725 | 0 | 0 | 0 | 0 | 0.000108 | 0.000145 |
| d__Bacteria;p__Proteobacteria;c__Deltaproteobacteria;o__Desulfobacterales | 0 | 0 | 0 | 0 | 0 | 0 | 0 | 0 | 0 | 0 | 0 | 0 |
| d__Bacteria;p__Proteobacteria;c__Deltaproteobacteria;o__Desulfovibrionales | 0 | 0 | 0 | 0 | 0 | 0 | 0 | 0 | 0 | 0 | 0 | 0 |
| d__Bacteria;p__Proteobacteria;c__Deltaproteobacteria;o__Desulfuromonadales | 0 | 0 | 0 | 0 | 0.0001 | 0 | 0 | 0 | 0 | 0 | 0 | 0 |
| d__Bacteria;p__Proteobacteria;c__Deltaproteobacteria;o__MBNT15 | 0.000759 | 0.000593 | 0.002747 | 0.000125 | 0.000641 | 0 | 0 | 0 | 0 | 0 | 0 | 0.000127 |
| d__Bacteria;p__Proteobacteria;c__Deltaproteobacteria;o__Myxococcales | 0.027559 | 0.013574 | 0.025377 | 0.034133 | 0.030437 | 0.015875 | 0.022418 | 0.023685 | 0.024747 | 0.026034 | 0.018116 | 0.017848 |
| d__Bacteria;p__Proteobacteria;c__Deltaproteobacteria;o__NB1-j | 0 | 0 | 0.000751 | 0 | 0 | 0 | 0 | 0 | 0 | 0 | 0 | 0 |
| d__Bacteria;p__Proteobacteria;c__Deltaproteobacteria;o__Oligoflexales | 0.003376 | 0.001544 | 0.002507 | 0.004609 | 0.002323 | 0.0024 | 0.002349 | 0.002048 | 0.002551 | 0.002734 | 0.001143 | 0.001618 |
| d__Bacteria;p__Proteobacteria;c__Deltaproteobacteria;o__PB19 | 0 | 0 | 0 | 0 | 0 | 0 | 0 | 0 | 0 | 0 | 0 | 0 |
| d__Bacteria;p__Proteobacteria;c__Deltaproteobacteria;o__RCP2-54 | 0.002305 | 0.006697 | 0.001968 | 0.002512 | 0.004786 | 0.00165 | 0.000664 | 0.000156 | 0.000325 | 0.000422 | 0 | 0.000818 |
| d__Bacteria;p__Proteobacteria;c__Deltaproteobacteria;o__SAR324_clade(Marine_group_B) | 7.44E-05 | 0 | 0.000184 | 0 | 0.0001 | 0 | 0 | 0 | 0 | 0 | 4.31E-05 | 0.000164 |
| d__Bacteria;p__Proteobacteria;c__Deltaproteobacteria;o__Syntrophobacterales | 0 | 0.000108 | 0 | 0 | 0 | 0 | 0 | 0 | 0 | 0 | 0 | 5.45E-05 |
| d__Bacteria;p__Proteobacteria;c__Deltaproteobacteria;o__unclassified_Deltaproteobacteria | 5.95E-05 | 1.80E-05 | 0.000864 | 4.15E-05 | 0.00024 | 5.00E-05 | 5.11E-05 | 3.47E-05 | 0 | 0 | 0 | 0.000164 |
| d__Bacteria;p__Proteobacteria;c__Gammaproteobacteria;o__Alteromonadales | 0 | 0 | 0 | 0 | 0 | 0 | 0 | 0 | 0 | 0 | 0 | 0 |
| d__Bacteria;p__Proteobacteria;c__Gammaproteobacteria;o__B2M28 | 0 | 0 | 0 | 0 | 0 | 0 | 0 | 0 | 0 | 0 | 0 | 0 |
| d__Bacteria;p__Proteobacteria;c__Gammaproteobacteria;o__BD72BR169 | 5.95E-05 | 0 | 0 | 0 | 0 | 0 | 0 | 0 | 0 | 0 | 0 | 0 |
| d__Bacteria;p__Proteobacteria;c__Gammaproteobacteria;o__Betaproteobacteriales | 0.013698 | 0.01115 | 0.02348 | 0.013599 | 0.026412 | 0.058525 | 0.056479 | 0.064896 | 0.074502 | 0.07002 | 0.069423 | 0.061595 |
| d__Bacteria;p__Proteobacteria;c__Gammaproteobacteria;o__CCD24 | 0 | 0 | 0.000198 | 0 | 0 | 0 | 0 | 0 | 0 | 0 | 0.000216 | 0.000145 |
| d__Bacteria;p__Proteobacteria;c__Gammaproteobacteria;o__Cellvibrionales | 0 | 0 | 0 | 0 | 0 | 0.00015 | 0 | 0 | 0 | 0 | 0 | 0 |
| d__Bacteria;p__Proteobacteria;c__Gammaproteobacteria;o__Competibacterales | 0 | 0 | 0 | 0 | 0 | 0 | 0 | 0 | 0 | 0 | 0 | 0 |
| d__Bacteria;p__Proteobacteria;c__Gammaproteobacteria;o__Coxiellales | 0.00113 | 0.000808 | 0.000524 | 0.001038 | 0.00012 | 0.000175 | 0.000587 | 0.000226 | 0.000292 | 0.000563 | 0.000194 | 0.000236 |
| d__Bacteria;p__Proteobacteria;c__Gammaproteobacteria;o__Diplorickettsiales | 0.008299 | 0.00492 | 0.002733 | 0.009322 | 0.003644 | 0.0029 | 0.008554 | 0.010133 | 0.016785 | 0.01753 | 0.012034 | 0.019138 |
| d__Bacteria;p__Proteobacteria;c__Gammaproteobacteria;o__EC3 | 0.000312 | 7.18E-05 | 0 | 0 | 0 | 0 | 0 | 0 | 0 | 6.03E-05 | 0 | 0 |
| d__Bacteria;p__Proteobacteria;c__Gammaproteobacteria;o__EV818SWSAP88 | 0 | 0 | 0 | 0 | 0 | 0 | 0 | 0 | 0 | 0 | 0 | 0 |
| d__Bacteria;p__Proteobacteria;c__Gammaproteobacteria;o__Enterobacteriales | 0 | 0.000144 | 0.000283 | 8.30E-05 | 0.001061 | 0.002425 | 0 | 3.47E-05 | 0 | 0 | 0 | 0.000491 |
| d__Bacteria;p__Proteobacteria;c__Gammaproteobacteria;o__GB102 | 0 | 0 | 0 | 0 | 0 | 0 | 0 | 0 | 0 | 0 | 0 | 0 |
| d__Bacteria;p__Proteobacteria;c__Gammaproteobacteria;o__Gammaproteobacteria_Incertae_Sedis | 0.013817 | 0.013502 | 0.013312 | 0.016672 | 0.007109 | 0.004 | 0.012562 | 0.010411 | 0.008612 | 0.00973 | 0.006168 | 0.005725 |
| d__Bacteria;p__Proteobacteria;c__Gammaproteobacteria;o__JG36-TzT-191 | 0.004417 | 0.001796 | 0.001983 | 0.011772 | 0.001181 | 0.001125 | 0 | 0.000312 | 0.00013 | 6.03E-05 | 0.00041 | 0.0012 |
| d__Bacteria;p__Proteobacteria;c__Gammaproteobacteria;o__KF-JG30-C25 | 5.95E-05 | 0 | 9.91E-05 | 0.000478 | 0.000441 | 0.000775 | 0.001455 | 0.001562 | 0.00065 | 0.000482 | 0.000496 | 0.000218 |
| d__Bacteria;p__Proteobacteria;c__Gammaproteobacteria;o__KI89A_clade | 0 | 0 | 0.000198 | 0 | 0.00016 | 0.0001 | 0 | 0 | 0 | 0 | 0 | 0 |
| d__Bacteria;p__Proteobacteria;c__Gammaproteobacteria;o__Legionellales | 0.000788 | 0.000269 | 0.00051 | 0.000727 | 0.000501 | 0.000525 | 0.001609 | 0.001475 | 0.003006 | 0.003458 | 0.002394 | 0.002963 |
| d__Bacteria;p__Proteobacteria;c__Gammaproteobacteria;o__Oceanospirillales | 0.036617 | 0.035605 | 0.001331 | 0.00353 | 0.003244 | 0.002075 | 0.006307 | 0.007166 | 0.025446 | 0.012464 | 0.006988 | 0.05338 |
| d__Bacteria;p__Proteobacteria;c__Gammaproteobacteria;o__PLTA13 | 0 | 0 | 0 | 0 | 0 | 0 | 0 | 0 | 0 | 0 | 0 | 0 |
| d__Bacteria;p__Proteobacteria;c__Gammaproteobacteria;o__Pseudomonadales | 0.000565 | 0.002011 | 0.001841 | 0.000914 | 0.002443 | 0.00225 | 0.000102 | 0.001579 | 0.001105 | 0.000201 | 0.001423 | 0.006507 |
| d__Bacteria;p__Proteobacteria;c__Gammaproteobacteria;o__R7C24 | 0 | 0 | 0 | 0 | 0 | 0 | 0 | 0 | 0 | 0 | 0 | 5.45E-05 |
| d__Bacteria;p__Proteobacteria;c__Gammaproteobacteria;o__Salinisphaerales | 2.97E-05 | 0.000108 | 0 | 8.30E-05 | 6.01E-05 | 0.000275 | 0.000153 | 0.000607 | 0.001056 | 0.000643 | 0.000561 | 0.000945 |
| d__Bacteria;p__Proteobacteria;c__Gammaproteobacteria;o__Steroidobacterales | 0.000268 | 5.39E-05 | 0.001062 | 0.000644 | 0.00034 | 0 | 0.000562 | 0.001336 | 0.000601 | 0.000342 | 0 | 0.000145 |
| d__Bacteria;p__Proteobacteria;c__Gammaproteobacteria;o__WD260 | 0.006604 | 0.007182 | 0.002705 | 0.012416 | 0.0168 | 0.014575 | 0.002758 | 0.00321 | 0.002649 | 0.00386 | 0.001596 | 0.001054 |
| d__Bacteria;p__Proteobacteria;c__Gammaproteobacteria;o__Xanthomonadales | 0.005533 | 0.001095 | 0.001841 | 0.006249 | 0.014658 | 0.010875 | 0.015064 | 0.022037 | 0.027445 | 0.023762 | 0.04818 | 0.02221 |
| d__Bacteria;p__Proteobacteria;c__Gammaproteobacteria;o__unclassified_Gammaproteobacteria | 0.000967 | 0.001005 | 0.001034 | 0.001661 | 0.001121 | 0.00035 | 0.000664 | 0.000538 | 0.001397 | 0.001568 | 0.000604 | 0.000545 |
| d__Bacteria;p__Proteobacteria;c__Gammaproteobacteria;o__uncultured | 0.000134 | 8.98E-05 | 0.000142 | 0.000187 | 0 | 0 | 7.66E-05 | 0.000312 | 0.000829 | 0.000382 | 0 | 0 |
| d__Bacteria;p__Proteobacteria;c__unclassified_Proteobacteria;o__unclassified_Proteobacteria | 4.46E-05 | 8.98E-05 | 0 | 0.000104 | 0.00012 | 0.0002 | 0.000306 | 0.000156 | 0.000146 | 0.000141 | 0.000129 | 7.27E-05 |
| d__Bacteria;p__Rokubacteria;c__NC10;o__Rokubacteriales | 0.001443 | 0.001688 | 0.004631 | 0 | 0.000541 | 0.0002 | 0 | 0 | 0 | 0 | 0.000108 | 0.000291 |
| d__Bacteria;p__Spirochaetes;c__Leptospirae;o__Leptospirales | 0 | 0 | 0 | 0 | 0 | 0 | 0.000102 | 6.94E-05 | 8.12E-05 | 0.000362 | 0.000151 | 3.63E-05 |
| d__Bacteria;p__Spirochaetes;c__Spirochaetia;o__Brevinematales | 0 | 0 | 0 | 0 | 0 | 0 | 0 | 0 | 0 | 0 | 0 | 0 |
| d__Bacteria;p__Spirochaetes;c__Spirochaetia;o__Spirochaetales | 0 | 5.39E-05 | 0 | 0 | 0 | 0 | 0 | 0 | 0.000114 | 0 | 0 | 0.0002 |
| d__Bacteria;p__Tenericutes;c__Mollicutes;o__Mycoplasmatales | 0 | 0 | 0 | 0 | 0 | 0 | 0 | 0 | 0 | 0 | 0 | 0 |
| d__Bacteria;p__Verrucomicrobia;c__Verrucomicrobiae;o__Chthoniobacterales | 0.009801 | 0.014957 | 0.018693 | 0.002533 | 0.010172 | 0.00395 | 0.001379 | 0.00026 | 0.000455 | 0.001146 | 0.002847 | 0.013413 |
| d__Bacteria;p__Verrucomicrobia;c__Verrucomicrobiae;o__Methylacidiphilales | 0.000164 | 0.000287 | 0.000184 | 0.000311 | 0.00034 | 0.00025 | 0 | 5.21E-05 | 0 | 0 | 8.63E-05 | 0.000127 |
| d__Bacteria;p__Verrucomicrobia;c__Verrucomicrobiae;o__Opitutales | 0.000327 | 0.000467 | 0.000156 | 0.000976 | 0.000421 | 0.000225 | 0.000894 | 0.000659 | 0.001202 | 0.000824 | 0.00041 | 0.000418 |
| d__Bacteria;p__Verrucomicrobia;c__Verrucomicrobiae;o__Pedosphaerales | 0.003659 | 0.001778 | 0.002096 | 0.00353 | 0.002042 | 0.0007 | 0.002681 | 0.001614 | 0.003087 | 0.004181 | 0.001057 | 0.001581 |
| d__Bacteria;p__Verrucomicrobia;c__Verrucomicrobiae;o__S-BQ2-57_soil_group | 0.000967 | 0.00149 | 0.000963 | 0.000914 | 0.00024 | 0 | 0 | 0 | 9.75E-05 | 0 | 0 | 0 |
| d__Bacteria;p__Verrucomicrobia;c__Verrucomicrobiae;o__Verrucomicrobiales | 0.000223 | 7.18E-05 | 0 | 0 | 4.00E-05 | 7.50E-05 | 0 | 6.94E-05 | 8.12E-05 | 0.000302 | 8.63E-05 | 7.27E-05 |
| d__Bacteria;p__Verrucomicrobia;c__Verrucomicrobiae;o__unclassified_Verrucomicrobiae | 0 | 3.59E-05 | 8.50E-05 | 0 | 0 | 0 | 5.11E-05 | 0 | 0 | 4.02E-05 | 0 | 0 |
| d__Bacteria;p__Verrucomicrobia;c__Verrucomicrobiae;o__uncultured | 0 | 0 | 0 | 0 | 0 | 0 | 0 | 0 | 0 | 0 | 0 | 0 |
| d__Bacteria;p__WPS-2;c__WPS-2;o__WPS-2 | 0.068697 | 0.038119 | 0.098564 | 0.040195 | 0.029236 | 0.016775 | 0.006996 | 0.006299 | 0.004079 | 0.003357 | 0.003364 | 0.002399 |
| d__Bacteria;p__WS2;c__WS2;o__WS2 | 0 | 0.000144 | 0 | 0 | 0 | 0 | 0 | 0.000243 | 0.000325 | 0.000342 | 0.000345 | 0.000782 |
| d__Bacteria;p__unclassified_Bacteria;c__unclassified_Bacteria;o__unclassified_Bacteria | 0.001145 | 0.000718 | 0.00092 | 0.001578 | 0.000581 | 0.00035 | 0.001941 | 0.001319 | 0.00195 | 0.010916 | 0.002027 | 0.00149 |

**Supplemental table 4b** Soil fungal_OTU_table at order

| Taxon | **WT_1** | **WT_2** | **WT_3** | **WT_4** | **WT_5** | **WT_6** | **CT_1** | **CT_2** | **CT_3** | **CT_4** | **CT_5** | **CT_6** |
| --- | --- | --- | --- | --- | --- | --- | --- | --- | --- | --- | --- | --- |
| d__Fungi;p__Ascomycota;c__Archaeorhizomycetes;o__Archaeorhizomycetales | 0.022921 | 0.090637 | 0.006408 | 0.796463 | 0.088946 | 0.007909 | 0.000208 | 0.000135 | 0 | 0.000234 | 0.001146 | 0 |
| d__Fungi;p__Ascomycota;c__Dothideomycetes;o__Capnodiales | 0.00104 | 4.88E-05 | 0 | 0.000276 | 0.000195 | 0 | 0.000167 | 0.001016 | 0.001402 | 5.86E-05 | 0.001488 | 0.000429 |
| d__Fungi;p__Ascomycota;c__Dothideomycetes;o__Minutisphaerales | 0 | 0 | 0 | 0 | 0 | 0 | 0 | 0 | 0 | 0 | 0 | 0 |
| d__Fungi;p__Ascomycota;c__Dothideomycetes;o__Mytilinidales | 0 | 0 | 0 | 0.000386 | 0 | 0 | 0 | 0 | 0 | 0 | 0 | 0 |
| d__Fungi;p__Ascomycota;c__Dothideomycetes;o__Pleosporales | 0.000867 | 3.25E-05 | 0.007752 | 0.000202 | 0.000162 | 9.61E-05 | 0.048924 | 0.018516 | 0.038699 | 0.013221 | 0.018817 | 0.012985 |
| d__Fungi;p__Ascomycota;c__Dothideomycetes;o__Tubeufiales | 0 | 0 | 0 | 0 | 0 | 0 | 0 | 0.000406 | 0 | 0.000352 | 0.000362 | 0 |
| d__Fungi;p__Ascomycota;c__Dothideomycetes;o__Venturiales | 0 | 0 | 0.000564 | 0 | 0 | 0 | 0.000229 | 0.000994 | 0 | 0 | 0.026597 | 0.000279 |
| d__Fungi;p__Ascomycota;c__Dothideomycetes;o__unclassified_Dothideomycetes | 0 | 0 | 0 | 0 | 0 | 0 | 0.000978 | 0 | 0 | 0 | 0 | 0 |
| d__Fungi;p__Ascomycota;c__Eurotiomycetes;o__Chaetothyriales | 0.00182 | 0.000325 | 0 | 0 | 0.000503 | 0 | 0.005288 | 0.000835 | 0.003253 | 0.00291 | 0.01164 | 0.003707 |
| d__Fungi;p__Ascomycota;c__Eurotiomycetes;o__Eurotiales | 0.000462 | 0.000699 | 0.01323 | 0.000258 | 0.000292 | 0.000112 | 0.022193 | 0.025155 | 0.08998 | 0.074483 | 0.014957 | 0.016499 |
| d__Fungi;p__Ascomycota;c__Eurotiomycetes;o__Onygenales | 0 | 0 | 0 | 0 | 0 | 0 | 0.002019 | 0 | 0 | 0.002285 | 0.001166 | 0 |
| d__Fungi;p__Ascomycota;c__GS35;o__GS35 | 0 | 0 | 0 | 0 | 0 | 0.000144 | 0 | 0 | 0 | 0 | 0 | 0 |
| d__Fungi;p__Ascomycota;c__GS37;o__GS37 | 0 | 0 | 0 | 0 | 0 | 0 | 0 | 0 | 0 | 0 | 0 | 0 |
| d__Fungi;p__Ascomycota;c__Geoglossomycetes;o__Geoglossales | 0 | 0 | 0 | 0 | 0 | 0 | 0 | 0 | 0 | 0 | 0 | 0 |
| d__Fungi;p__Ascomycota;c__Laboulbeniomycetes;o__Pyxidiophorales | 0 | 0 | 0 | 0 | 0 | 0 | 0 | 0 | 0 | 0 | 0 | 0 |
| d__Fungi;p__Ascomycota;c__Lecanoromycetes;o__GS36 | 0 | 0 | 0 | 0 | 0 | 0 | 0 | 0 | 0 | 0 | 0.000181 | 0 |
| d__Fungi;p__Ascomycota;c__Lecanoromycetes;o__Ostropales | 0 | 0 | 0 | 0 | 0 | 0 | 0 | 0 | 0 | 0 | 0 | 0 |
| d__Fungi;p__Ascomycota;c__Lecanoromycetes;o__unclassified_Lecanoromycetes | 0 | 0 | 0 | 0 | 0 | 0 | 0 | 0 | 0 | 0 | 0 | 0 |
| d__Fungi;p__Ascomycota;c__Lecanoromycetes;o__unidentified | 0 | 0 | 0 | 0 | 0 | 0 | 0 | 0 | 0 | 0 | 0 | 0 |
| d__Fungi;p__Ascomycota;c__Leotiomycetes;o__Erysiphales | 0 | 0 | 0 | 0 | 0 | 0 | 4.16E-05 | 0 | 0 | 0 | 0 | 0 |
| d__Fungi;p__Ascomycota;c__Leotiomycetes;o__Helotiales | 0.055503 | 0.014973 | 0.136817 | 0.007729 | 0.010836 | 0.0004 | 0.033518 | 0.046245 | 0.043393 | 0.109987 | 0.061839 | 0.049454 |
| d__Fungi;p__Ascomycota;c__Leotiomycetes;o__Phacidiales | 0 | 0 | 0 | 0 | 0 | 0 | 0 | 0 | 0 | 0 | 0.000181 | 0 |
| d__Fungi;p__Ascomycota;c__Leotiomycetes;o__Thelebolales | 0.129681 | 0.004715 | 0.103966 | 0.000865 | 0.002725 | 8.01E-05 | 0.0183 | 0.012871 | 0.044814 | 0.01451 | 0.065659 | 0.134905 |
| d__Fungi;p__Ascomycota;c__Leotiomycetes;o__unclassified_Leotiomycetes | 0.000303 | 0 | 0 | 3.68E-05 | 0.00047 | 0 | 0 | 0 | 0 | 0 | 0.000121 | 0 |
| d__Fungi;p__Ascomycota;c__Leotiomycetes;o__unidentified | 0 | 0 | 0 | 0 | 0 | 0 | 0.000791 | 0 | 0 | 0 | 0 | 0 |
| d__Fungi;p__Ascomycota;c__Orbiliomycetes;o__Orbiliales | 0 | 0 | 0 | 0 | 0 | 0 | 0.002061 | 0 | 0 | 0 | 0.000322 | 0 |
| d__Fungi;p__Ascomycota;c__Orbiliomycetes;o__unidentified | 0 | 0 | 0.004465 | 0 | 0 | 0 | 0 | 0 | 0 | 0 | 0 | 0 |
| d__Fungi;p__Ascomycota;c__Pezizomycetes;o__Pezizales | 0 | 0 | 0.001394 | 7.36E-05 | 0 | 0 | 0 | 0 | 3.90E-05 | 0.000156 | 0.000342 | 0 |
| d__Fungi;p__Ascomycota;c__Saccharomycetes;o__Saccharomycetales | 0.001387 | 0.001301 | 0.002971 | 0.000828 | 0.000941 | 0.001121 | 0.002852 | 0.002077 | 0.002746 | 0.001621 | 0.002292 | 0.001671 |
| d__Fungi;p__Ascomycota;c__Saccharomycetes;o__unidentified | 0 | 0 | 0 | 0 | 0.000438 | 0.000208 | 0 | 0 | 0 | 0 | 0 | 0 |
| d__Fungi;p__Ascomycota;c__Sordariomycetes;o__Chaetosphaeriales | 0.001618 | 0 | 0 | 3.68E-05 | 0.000308 | 0 | 0.000146 | 0.00061 | 0 | 0.001836 | 0 | 0 |
| d__Fungi;p__Ascomycota;c__Sordariomycetes;o__Coniochaetales | 0 | 4.88E-05 | 0.000847 | 0 | 0 | 0 | 0.001312 | 0.001061 | 0 | 0 | 0.00396 | 0.002443 |
| d__Fungi;p__Ascomycota;c__Sordariomycetes;o__Diaporthales | 0.000202 | 0 | 0 | 0 | 3.24E-05 | 0 | 0 | 0 | 0 | 0.000195 | 6.03E-05 | 0 |
| d__Fungi;p__Ascomycota;c__Sordariomycetes;o__Glomerellales | 0.000101 | 0.00013 | 0 | 0.000202 | 0.000162 | 0 | 0.000167 | 0 | 0.003856 | 0.001055 | 0.004061 | 0.000193 |
| d__Fungi;p__Ascomycota;c__Sordariomycetes;o__Hypocreales | 0.008247 | 0.001333 | 0.006109 | 0.00035 | 0.003861 | 0 | 0.172586 | 0.384185 | 0.365235 | 0.273464 | 0.214708 | 0.188922 |
| d__Fungi;p__Ascomycota;c__Sordariomycetes;o__Lulworthiales | 0 | 0 | 0 | 0 | 0 | 0 | 0 | 0.000226 | 0 | 0 | 0 | 0 |
| d__Fungi;p__Ascomycota;c__Sordariomycetes;o__Magnaporthales | 0 | 0 | 0 | 0 | 0 | 0 | 0.001978 | 0 | 0 | 0 | 0 | 0.00045 |
| d__Fungi;p__Ascomycota;c__Sordariomycetes;o__Microascales | 0.000173 | 9.75E-05 | 0 | 0 | 9.73E-05 | 0 | 0 | 4.52E-05 | 0 | 0 | 0.000141 | 0 |
| d__Fungi;p__Ascomycota;c__Sordariomycetes;o__Myrmecridiales | 0 | 0 | 0.001328 | 0 | 0 | 0 | 0.000375 | 0.001761 | 0 | 0 | 0.002654 | 0 |
| d__Fungi;p__Ascomycota;c__Sordariomycetes;o__Sordariales | 0.012305 | 0.001317 | 0.010757 | 0 | 0.000665 | 0 | 0.038827 | 0.099174 | 0.068965 | 0.070519 | 0.023622 | 0.025927 |
| d__Fungi;p__Ascomycota;c__Sordariomycetes;o__Xylariales | 0.001618 | 0.000179 | 0.008881 | 0 | 3.24E-05 | 0 | 0.000791 | 0 | 0.00111 | 0.000215 | 0.000101 | 0.000857 |
| d__Fungi;p__Ascomycota;c__Sordariomycetes;o__unclassified_Sordariomycetes | 0.00013 | 8.13E-05 | 0 | 0.000202 | 0.000276 | 0 | 0.020881 | 0.04918 | 0.035894 | 0.008456 | 0.028427 | 0.008999 |
| d__Fungi;p__Ascomycota;c__Sordariomycetes;o__unidentified | 0 | 0 | 0.001942 | 0 | 0 | 3.20E-05 | 0.003706 | 0.000429 | 0 | 0 | 0.000121 | 0 |
| d__Fungi;p__Ascomycota;c__Taphrinomycetes;o__Taphrinales | 0 | 0 | 0 | 0 | 0 | 0 | 0 | 0 | 0 | 0 | 0 | 0 |
| d__Fungi;p__Ascomycota;c__Xylonomycetes;o__GS34 | 0 | 0 | 0 | 3.68E-05 | 0 | 0 | 0 | 0 | 0 | 0 | 0 | 0 |
| d__Fungi;p__Ascomycota;c__unclassified_Ascomycota;o__unclassified_Ascomycota | 0.001343 | 0 | 0.001311 | 0 | 0.000552 | 9.61E-05 | 0.000479 | 0 | 0.001772 | 0.011385 | 0.006654 | 0.000879 |
| d__Fungi;p__Ascomycota;c__unidentified;o__unidentified | 0 | 0.012535 | 0 | 0 | 0 | 0 | 0 | 0 | 0 | 0 | 0 | 0 |
| d__Fungi;p__Basidiomycota;c__Agaricomycetes;o__Agaricales | 0.262063 | 0.032483 | 0.382397 | 0.113326 | 0.530952 | 0.949118 | 0.003019 | 0.00709 | 0.000721 | 0.001211 | 0.013208 | 0.012449 |
| d__Fungi;p__Basidiomycota;c__Agaricomycetes;o__Atheliales | 4.33E-05 | 0.056967 | 0.00083 | 0.014557 | 0.018834 | 0.002402 | 0.005912 | 0 | 0 | 0.00125 | 0.000885 | 0.001478 |
| d__Fungi;p__Basidiomycota;c__Agaricomycetes;o__Auriculariales | 0.079637 | 6.50E-05 | 6.64E-05 | 0 | 0 | 0.00032 | 0.21947 | 0 | 0 | 0 | 0.012484 | 0 |
| d__Fungi;p__Basidiomycota;c__Agaricomycetes;o__Boletales | 0.000376 | 0 | 0 | 0.003478 | 0.001265 | 0.003266 | 0.000104 | 0 | 0 | 0.001504 | 0.000462 | 0 |
| d__Fungi;p__Basidiomycota;c__Agaricomycetes;o__Cantharellales | 0 | 0.038125 | 0.000963 | 0.005318 | 0.015849 | 3.20E-05 | 6.25E-05 | 0.003613 | 0.010887 | 0.003847 | 0.001287 | 0.000386 |
| d__Fungi;p__Basidiomycota;c__Agaricomycetes;o__Geastrales | 0 | 0 | 0 | 0 | 6.49E-05 | 0 | 0.000229 | 0.001965 | 0.002824 | 0 | 0 | 0 |
| d__Fungi;p__Basidiomycota;c__Agaricomycetes;o__Gloeophyllales | 0 | 0 | 0 | 0 | 0 | 0 | 0 | 0 | 0 | 0 | 0 | 0 |
| d__Fungi;p__Basidiomycota;c__Agaricomycetes;o__Gomphales | 0 | 0 | 0.004947 | 0 | 0 | 0 | 0 | 0 | 0 | 0 | 0 | 0 |
| d__Fungi;p__Basidiomycota;c__Agaricomycetes;o__Hymenochaetales | 0.018747 | 0.001675 | 0 | 0 | 0 | 0 | 0 | 0 | 0 | 0 | 0.000181 | 0 |
| d__Fungi;p__Basidiomycota;c__Agaricomycetes;o__Jaapiales | 0 | 0 | 0 | 0 | 0 | 0 | 0 | 0 | 0 | 0 | 0 | 0 |
| d__Fungi;p__Basidiomycota;c__Agaricomycetes;o__Phallales | 0 | 6.50E-05 | 0 | 0 | 0 | 0 | 0 | 0 | 0 | 0 | 0 | 0 |
| d__Fungi;p__Basidiomycota;c__Agaricomycetes;o__Polyporales | 0.051849 | 0.003756 | 0.033482 | 0.000663 | 0.001152 | 0.000144 | 0.002561 | 0.000565 | 0.000584 | 3.91E-05 | 0.001468 | 0 |
| d__Fungi;p__Basidiomycota;c__Agaricomycetes;o__Russulales | 0.004809 | 0.279032 | 0.00239 | 0.014005 | 0.26314 | 0.00032 | 0.001374 | 0.000361 | 0.000214 | 7.81E-05 | 0.002915 | 0.000107 |
| d__Fungi;p__Basidiomycota;c__Agaricomycetes;o__Sebacinales | 0.000303 | 0.00039 | 0.000183 | 0.000386 | 0.003163 | 0.026114 | 0.000291 | 4.52E-05 | 0.002629 | 0.000605 | 0.001809 | 0 |
| d__Fungi;p__Basidiomycota;c__Agaricomycetes;o__Thelephorales | 0.003943 | 0.399909 | 0.005063 | 0.020722 | 0.007122 | 0.005444 | 0.000416 | 0.000248 | 0.003311 | 0 | 0.001568 | 0.000621 |
| d__Fungi;p__Basidiomycota;c__Agaricomycetes;o__Trechisporales | 0.000505 | 0.000602 | 0.00259 | 0.001417 | 0.004104 | 0 | 0.059541 | 0.12058 | 0.007654 | 0.009393 | 0.062563 | 0 |
| d__Fungi;p__Basidiomycota;c__Agaricomycetes;o__unclassified_Agaricomycetes | 0.008102 | 0 | 0.001693 | 0.006404 | 0.000552 | 0 | 0.169547 | 0.001355 | 0 | 0.044643 | 0.005388 | 0 |
| d__Fungi;p__Basidiomycota;c__Agaricomycetes;o__unidentified | 0 | 0.000894 | 0.027108 | 0.002172 | 0.000795 | 0 | 0.000146 | 0.000248 | 0 | 5.86E-05 | 0.005126 | 0 |
| d__Fungi;p__Basidiomycota;c__Cystobasidiomycetes;o__Cystobasidiales | 0 | 0 | 0 | 0 | 0 | 0 | 0 | 0 | 0 | 0 | 0.000342 | 0 |
| d__Fungi;p__Basidiomycota;c__Geminibasidiomycetes;o__Geminibasidiales | 0 | 0 | 0 | 0 | 0.00013 | 0 | 0 | 0 | 0 | 0 | 0 | 0 |
| d__Fungi;p__Basidiomycota;c__Malasseziomycetes;o__Malasseziales | 0 | 0 | 0.002606 | 0 | 0 | 0 | 0 | 0 | 0.000428 | 0 | 0 | 0 |
| d__Fungi;p__Basidiomycota;c__Microbotryomycetes;o__Kriegeriales | 0 | 0 | 0 | 0 | 0 | 0 | 0 | 0 | 0 | 0 | 0.000161 | 0 |
| d__Fungi;p__Basidiomycota;c__Microbotryomycetes;o__Leucosporidiales | 0.003813 | 0 | 0 | 0 | 0.001411 | 8.01E-05 | 0.000167 | 0.001377 | 0 | 0 | 0.000462 | 0 |
| d__Fungi;p__Basidiomycota;c__Microbotryomycetes;o__Microbotryomycetes_ord_Incertae_sedis | 0 | 0 | 0 | 0.000221 | 4.87E-05 | 0 | 0 | 0 | 0 | 0 | 0 | 0 |
| d__Fungi;p__Basidiomycota;c__Microbotryomycetes;o__Sporidiobolales | 0 | 8.13E-05 | 0 | 0 | 0 | 0.000384 | 0 | 0 | 0 | 0 | 0 | 0 |
| d__Fungi;p__Basidiomycota;c__Microbotryomycetes;o__unclassified_Microbotryomycetes | 0 | 0 | 0 | 0 | 0 | 0 | 0 | 0 | 0 | 0 | 0 | 0 |
| d__Fungi;p__Basidiomycota;c__Microbotryomycetes;o__unidentified | 0 | 0 | 0 | 0 | 0 | 0 | 0 | 0 | 0 | 0 | 0.000121 | 0 |
| d__Fungi;p__Basidiomycota;c__Pucciniomycetes;o__Platygloeales | 0 | 0 | 0 | 0 | 0 | 0 | 0 | 0 | 0 | 0 | 0 | 0 |
| d__Fungi;p__Basidiomycota;c__Pucciniomycetes;o__Pucciniales | 0 | 0.000163 | 0 | 0 | 0 | 0 | 0 | 0 | 0 | 0 | 0 | 0 |
| d__Fungi;p__Basidiomycota;c__Tremellomycetes;o__Cystofilobasidiales | 0 | 0 | 0.002424 | 0 | 0 | 0 | 6.25E-05 | 0.000248 | 0 | 0.001094 | 0 | 0 |
| d__Fungi;p__Basidiomycota;c__Tremellomycetes;o__Filobasidiales | 0.002138 | 0.001317 | 0.00176 | 0.00057 | 0.001703 | 0.000144 | 0.012679 | 0.009597 | 0.048067 | 0.013338 | 0.021612 | 0.009214 |
| d__Fungi;p__Basidiomycota;c__Tremellomycetes;o__Holtermanniales | 0 | 0 | 0 | 0 | 0 | 0 | 0.000479 | 0.000429 | 0.00037 | 0.002988 | 0.006473 | 0 |
| d__Fungi;p__Basidiomycota;c__Tremellomycetes;o__Tremellales | 0.001805 | 0.001366 | 0.000166 | 0.00173 | 0.004542 | 0.001393 | 0.00304 | 0.000384 | 0 | 0 | 0.009449 | 0 |
| d__Fungi;p__Basidiomycota;c__Tremellomycetes;o__Trichosporonales | 0 | 0 | 0 | 0 | 4.87E-05 | 0 | 0 | 0 | 0 | 0 | 0.000302 | 0 |
| d__Fungi;p__Basidiomycota;c__Tremellomycetes;o__unclassified_Tremellomycetes | 0 | 0 | 0 | 5.52E-05 | 0 | 0 | 0 | 0 | 0 | 0 | 8.04E-05 | 0 |
| d__Fungi;p__Basidiomycota;c__Ustilaginomycetes;o__Ustilaginales | 0 | 0 | 0 | 0 | 0 | 0 | 0 | 0 | 0 | 0 | 0 | 0 |
| d__Fungi;p__Basidiomycota;c__Wallemiomycetes;o__Wallemiales | 0 | 0 | 0 | 0 | 0 | 0 | 0 | 0.000406 | 0 | 0 | 0 | 0 |
| d__Fungi;p__Basidiomycota;c__unclassified_Basidiomycota;o__unclassified_Basidiomycota | 0.004102 | 0.000146 | 0.003851 | 0.00011 | 0.002563 | 0 | 0.000167 | 0 | 0 | 0 | 0.001166 | 0.000129 |
| d__Fungi;p__Blastocladiomycota;c__Blastocladiomycetes;o__GS15 | 0 | 0 | 0 | 0 | 0 | 0 | 0 | 0 | 0 | 0 | 0 | 0 |
| d__Fungi;p__Chytridiomycota;c__Chytridiomycetes;o__Chytridiales | 0 | 0 | 0 | 0 | 0 | 0 | 0.000187 | 0.000135 | 0 | 0 | 0 | 0 |
| d__Fungi;p__Chytridiomycota;c__Rhizophlyctidomycetes;o__Rhizophlyctidales | 0 | 0 | 0 | 0 | 0 | 0 | 0 | 0 | 0 | 0.007499 | 0 | 0 |
| d__Fungi;p__Chytridiomycota;c__Rhizophydiomycetes;o__Rhizophydiales | 0.000289 | 0 | 0.000714 | 0 | 0 | 0 | 0 | 0 | 0 | 0 | 0 | 0 |
| d__Fungi;p__Chytridiomycota;c__unclassified_Chytridiomycota;o__unclassified_Chytridiomycota | 0 | 0 | 0 | 0 | 0 | 0 | 0 | 0 | 0 | 0 | 0 | 0 |
| d__Fungi;p__Chytridiomycota;c__unidentified;o__unidentified | 0.003784 | 0 | 0.00083 | 0 | 6.49E-05 | 0 | 0 | 0 | 0 | 0 | 0 | 0.000343 |
| d__Fungi;p__GS01;c__unidentified;o__unidentified | 0 | 0 | 0 | 0 | 0 | 0 | 0 | 0 | 0 | 0 | 0 | 0 |
| d__Fungi;p__Glomeromycota;c__Archaeosporomycetes;o__Archaeosporales | 0.000635 | 0.000829 | 0.00083 | 0 | 0 | 0 | 0 | 0 | 0 | 0 | 0.000181 | 0.000236 |
| d__Fungi;p__Glomeromycota;c__Glomeromycetes;o__Diversisporales | 0 | 0 | 0 | 0 | 0 | 0 | 0 | 0 | 0 | 0 | 0 | 0 |
| d__Fungi;p__Glomeromycota;c__Glomeromycetes;o__Glomerales | 0.00091 | 0 | 0 | 3.68E-05 | 0 | 0 | 0 | 0 | 0 | 0.000234 | 0 | 0 |
| d__Fungi;p__Glomeromycota;c__Paraglomeromycetes;o__Paraglomerales | 0 | 0 | 0 | 0 | 0 | 0 | 0.000333 | 0.000294 | 0.000234 | 0 | 0.000322 | 0.000921 |
| d__Fungi;p__Glomeromycota;c__unclassified_Glomeromycota;o__unclassified_Glomeromycota | 0.002239 | 0 | 0.000747 | 0 | 0 | 0 | 0 | 0 | 0 | 0 | 0.000302 | 0.00135 |
| d__Fungi;p__Glomeromycota;c__unidentified;o__unidentified | 5.78E-05 | 0 | 0 | 0 | 0 | 0 | 0 | 0 | 0 | 0 | 0 | 0 |
| d__Fungi;p__Kickxellomycota;c__GS19;o__GS19 | 0 | 0 | 0 | 0 | 0 | 0 | 0 | 0 | 0 | 0 | 0 | 0 |
| d__Fungi;p__Kickxellomycota;c__Kickxellomycetes;o__Kickxellales | 0 | 0 | 0.000647 | 0 | 0 | 0 | 0 | 0 | 7.79E-05 | 0 | 0 | 0 |
| d__Fungi;p__Monoblepharomycota;c__Monoblepharidomycetes;o__unidentified | 0 | 0 | 0 | 0 | 0 | 0 | 0 | 0 | 0 | 0 | 0 | 0 |
| d__Fungi;p__Monoblepharomycota;c__Sanchytriomycetes;o__Sanchytriales | 0 | 0 | 0 | 0 | 0 | 0 | 0 | 0 | 0 | 0 | 0 | 0 |
| d__Fungi;p__Mortierellomycota;c__Mortierellomycetes;o__Mortierellales | 0.120915 | 0.032792 | 0.056506 | 0.001785 | 0.020764 | 0.00024 | 0.04578 | 0.06169 | 0.061564 | 0.155548 | 0.138434 | 0.250654 |
| d__Fungi;p__Mucoromycota;c__Mucoromycetes;o__Mucorales | 0.000188 | 0 | 0.000282 | 0.000258 | 9.73E-05 | 0 | 0.000229 | 6.77E-05 | 0.000623 | 0.00043 | 0.000744 | 0.000664 |
| d__Fungi;p__Mucoromycota;c__Umbelopsidomycetes;o__Umbelopsidales | 0.001069 | 0 | 0 | 0.000313 | 0.000552 | 0 | 0.001124 | 0.000519 | 0 | 0 | 0.000724 | 0 |
| d__Fungi;p__Olpidiomycota;c__Olpidiomycetes;o__Olpidiales | 0 | 0 | 0.00073 | 0 | 0 | 0 | 0 | 0 | 0.001617 | 0 | 0 | 0 |
| d__Fungi;p__Rozellomycota;c__Rozellomycotina_cls_Incertae_sedis;o__GS02 | 0 | 0 | 0 | 0 | 0 | 0 | 0 | 0 | 0 | 0 | 0 | 0.005464 |
| d__Fungi;p__Rozellomycota;c__Rozellomycotina_cls_Incertae_sedis;o__GS04 | 0 | 0.001398 | 0 | 0 | 0 | 0 | 0 | 0.0007 | 0 | 0.000508 | 0 | 0 |
| d__Fungi;p__Rozellomycota;c__Rozellomycotina_cls_Incertae_sedis;o__GS05 | 0 | 0 | 0 | 0 | 0 | 0 | 0 | 0 | 0 | 0.001445 | 0 | 0 |
| d__Fungi;p__Rozellomycota;c__Rozellomycotina_cls_Incertae_sedis;o__GS09 | 0 | 0 | 0.000166 | 0 | 0.000146 | 0 | 0 | 0 | 0 | 0 | 0 | 0 |
| d__Fungi;p__Rozellomycota;c__Rozellomycotina_cls_Incertae_sedis;o__GS10 | 0 | 0 | 0 | 0 | 0 | 0 | 0 | 0 | 0 | 0 | 0.000905 | 0 |
| d__Fungi;p__Rozellomycota;c__Rozellomycotina_cls_Incertae_sedis;o__GS11 | 0 | 0 | 0 | 0 | 0 | 0 | 0 | 0 | 0 | 0 | 0 | 0 |
| d__Fungi;p__Rozellomycota;c__unidentified;o__unidentified | 0.034735 | 0.001431 | 0.000232 | 0.001435 | 0.000406 | 0 | 0.000666 | 0.000474 | 0.000818 | 0.001836 | 0.007338 | 0 |
| d__Fungi;p__Zoopagomycota;c__Zoopagomycetes;o__Zoopagales | 0 | 0 | 0 | 0 | 0 | 0 | 0 | 0 | 0 | 0.003086 | 0.001829 | 0.000214 |
| d__Fungi;p__unclassified_Fungi;c__unclassified_Fungi;o__unclassified_Fungi | 0.10419 | 0.005755 | 0.040321 | 0.00276 | 0.007462 | 0.0004 | 0.035621 | 0.065506 | 0.074632 | 0.08208 | 0.145149 | 0.147204 |
| d__Fungi;p__unidentified;c__unidentified;o__unidentified | 0.049033 | 0.012307 | 0.116731 | 0.000331 | 0.001574 | 0 | 0.05798 | 0.07718 | 0.081585 | 0.080342 | 0.062945 | 0.11997 |

**Supplemental table 5a** Soil bacterial_OTU_table at family

| Taxon | **WT_1** | **WT_2** | **WT_3** | **WT_4** | **WT_5** | **WT_6** | **CT_1** | **CT_2** | **CT_3** | **CT_4** | **CT_5** | **CT_6** |
| --- | --- | --- | --- | --- | --- | --- | --- | --- | --- | --- | --- | --- |
| d__Bacteria;p__Acidobacteria;c__AT-s3-28;o__AT-s3-28;f__AT-s3-28 | 0 | 0 | 0.000113 | 0 | 0 | 0 | 0 | 0 | 0 | 0 | 0 | 0 |
| d__Bacteria;p__Acidobacteria;c__Acidobacteriia;o__Acidobacteriales;f__Acidobacteriaceae_(Subgroup_1) | 0.011616 | 0.008277 | 0.004418 | 0.022111 | 0.031819 | 0.10165 | 0.012511 | 0.010932 | 0.011683 | 0.012585 | 0.012293 | 0.006452 |
| d__Bacteria;p__Acidobacteria;c__Acidobacteriia;o__Acidobacteriales;f__Acidobacteriales | 0 | 3.59E-05 | 0 | 0 | 0 | 0 | 5.11E-05 | 0.000156 | 0 | 0 | 0 | 3.63E-05 |
| d__Bacteria;p__Acidobacteria;c__Acidobacteriia;o__Acidobacteriales;f__Koribacteraceae | 0.015215 | 0.00562 | 0.008327 | 0.005419 | 0.011434 | 0.0084 | 0.001966 | 0.001614 | 0.000747 | 0.001709 | 0.001704 | 0.001636 |
| d__Bacteria;p__Acidobacteria;c__Acidobacteriia;o__Acidobacteriales;f__unclassified_Acidobacteriales | 0.000461 | 0.001311 | 0.000609 | 6.23E-05 | 0.000421 | 0.0001 | 0.000894 | 0.000746 | 0.002454 | 0.002231 | 0.001531 | 0.001781 |
| d__Bacteria;p__Acidobacteria;c__Acidobacteriia;o__Acidobacteriales;f__uncultured | 0.060636 | 0.076328 | 0.062962 | 0.067622 | 0.051443 | 0.041 | 0.066922 | 0.040482 | 0.035569 | 0.024687 | 0.037246 | 0.054143 |
| d__Bacteria;p__Acidobacteria;c__Acidobacteriia;o__Solibacterales;f__Solibacteraceae_(Subgroup_3) | 0.067121 | 0.088771 | 0.068598 | 0.096107 | 0.08168 | 0.0495 | 0.03401 | 0.03479 | 0.040655 | 0.060471 | 0.042745 | 0.041911 |
| d__Bacteria;p__Acidobacteria;c__Acidobacteriia;o__Subgroup_12;f__Subgroup_12 | 0.00113 | 0.000503 | 0.000496 | 0.001163 | 0.00016 | 0.0001 | 0 | 0 | 0 | 0 | 0 | 0 |
| d__Bacteria;p__Acidobacteria;c__Acidobacteriia;o__Subgroup_13;f__Subgroup_13 | 0.002097 | 0.002532 | 0.001827 | 0.000893 | 0.00036 | 0.00035 | 0.000664 | 0.000798 | 0.000877 | 0.000945 | 0.000367 | 0.000727 |
| d__Bacteria;p__Acidobacteria;c__Acidobacteriia;o__Subgroup_2;f__Subgroup_2 | 0.077249 | 0.101016 | 0.04873 | 0.078376 | 0.066902 | 0.042975 | 0.015575 | 0.012337 | 0.013747 | 0.016485 | 0.010654 | 0.010323 |
| d__Bacteria;p__Acidobacteria;c__Acidobacteriia;o__unclassified_Acidobacteriia;f__unclassified_Acidobacteriia | 0 | 1.80E-05 | 0 | 0 | 0.0001 | 0 | 0 | 0 | 0 | 0 | 0 | 0 |
| d__Bacteria;p__Acidobacteria;c__Blastocatellia_(Subgroup_4);o__11-24;f__11-24 | 4.46E-05 | 0 | 5.66E-05 | 6.23E-05 | 0.000661 | 5.00E-05 | 5.11E-05 | 0.000278 | 0.000617 | 0.000804 | 0.000151 | 0.000164 |
| d__Bacteria;p__Acidobacteria;c__Blastocatellia_(Subgroup_4);o__Blastocatellales;f__Blastocatellaceae | 0 | 3.59E-05 | 0.000212 | 0 | 8.01E-05 | 0.00015 | 0.000613 | 0.000278 | 0.0013 | 0.005629 | 0.002782 | 0.005034 |
| d__Bacteria;p__Acidobacteria;c__Blastocatellia_(Subgroup_4);o__DS-100;f__DS-100 | 0 | 0 | 0 | 0 | 0 | 0 | 0 | 0 | 0 | 0 | 0 | 0 |
| d__Bacteria;p__Acidobacteria;c__Blastocatellia_(Subgroup_4);o__Elev-16S-573;f__Elev-16S-573 | 0 | 0 | 0 | 0 | 6.01E-05 | 0 | 0 | 0 | 0 | 0.000121 | 0 | 9.09E-05 |
| d__Bacteria;p__Acidobacteria;c__Blastocatellia_(Subgroup_4);o__Pyrinomonadales;f__Pyrinomonadaceae | 8.92E-05 | 0.000467 | 0.003654 | 0 | 0.0001 | 0 | 0 | 0 | 0 | 0.000281 | 0.000518 | 0.000963 |
| d__Bacteria;p__Acidobacteria;c__Blastocatellia_(Subgroup_4);o__unclassified_Blastocatellia_(Subgroup_4);f__unclassified_Blastocatellia_(Subgroup_4) | 0 | 0 | 0 | 0 | 0 | 0 | 0 | 0 | 0 | 0 | 0 | 5.45E-05 |
| d__Bacteria;p__Acidobacteria;c__Holophagae;o__Holophagales;f__Holophagaceae | 0 | 0 | 0 | 0 | 0 | 0 | 0.000204 | 0.000312 | 0.000829 | 0.000844 | 0.000216 | 0.000182 |
| d__Bacteria;p__Acidobacteria;c__Holophagae;o__Subgroup_7;f__Subgroup_7 | 0.011854 | 0.008277 | 0.022389 | 0.001453 | 0.002823 | 0.000975 | 0 | 0.000174 | 0.00052 | 0.002513 | 0.001467 | 0.004489 |
| d__Bacteria;p__Acidobacteria;c__Subgroup_11;o__Subgroup_11;f__Subgroup_11 | 0 | 0 | 0 | 0 | 0 | 0 | 0 | 0 | 0 | 0 | 0 | 0 |
| d__Bacteria;p__Acidobacteria;c__Subgroup_15;o__Subgroup_15;f__Subgroup_15 | 0 | 5.39E-05 | 0.000156 | 6.23E-05 | 0 | 0 | 0.000281 | 0.000121 | 0.000325 | 0.000462 | 0.000194 | 0.000454 |
| d__Bacteria;p__Acidobacteria;c__Subgroup_17;o__Subgroup_17;f__Subgroup_17 | 0.000134 | 0 | 0.000439 | 0 | 0.00032 | 0 | 0 | 0.000191 | 9.75E-05 | 0.000342 | 0 | 0.000309 |
| d__Bacteria;p__Acidobacteria;c__Subgroup_22;o__Subgroup_22;f__Subgroup_22 | 0 | 0 | 0 | 0 | 0 | 0 | 0 | 0 | 0 | 0 | 0 | 0 |
| d__Bacteria;p__Acidobacteria;c__Subgroup_25;o__Subgroup_25;f__Subgroup_25 | 0 | 0 | 0 | 0 | 0 | 0 | 0 | 0 | 0 | 0 | 0 | 7.27E-05 |
| d__Bacteria;p__Acidobacteria;c__Subgroup_5;o__Subgroup_5;f__Subgroup_5 | 0.004135 | 0.001688 | 0.002351 | 0.001952 | 0.000821 | 0.000275 | 0.000332 | 0.000399 | 0.00039 | 0.001387 | 0 | 0.000309 |
| d__Bacteria;p__Acidobacteria;c__Subgroup_6;o__Subgroup_6;f__Subgroup_6 | 0.022265 | 0.012856 | 0.031283 | 0.01144 | 0.011634 | 0.00405 | 0.01052 | 0.013881 | 0.021205 | 0.025571 | 0.019928 | 0.028044 |
| d__Bacteria;p__Acidobacteria;c__Subgroup_6;o__Unknown_Order;f__Unknown_Family | 0 | 0 | 0 | 0 | 0 | 0 | 5.11E-05 | 0 | 0 | 0 | 0.000129 | 5.45E-05 |
| d__Bacteria;p__Acidobacteria;c__Subgroup_6;o__unclassified_Subgroup_6;f__unclassified_Subgroup_6 | 8.92E-05 | 0.000126 | 8.50E-05 | 0.000187 | 4.00E-05 | 0.000225 | 0.000102 | 0.000156 | 4.87E-05 | 0.000241 | 0.000539 | 0.000145 |
| d__Bacteria;p__Acidobacteria;c__Thermoanaerobaculia;o__Thermoanaerobaculales;f__Thermoanaerobaculaceae | 0.006485 | 0.002424 | 0.002011 | 0.000664 | 0.000801 | 0.000425 | 0 | 0 | 0.00013 | 6.03E-05 | 0.000259 | 0.0002 |
| d__Bacteria;p__Acidobacteria;c__unclassified_Acidobacteria;o__unclassified_Acidobacteria;f__unclassified_Acidobacteria | 0.00058 | 0.000108 | 7.08E-05 | 0.000166 | 0 | 0 | 0 | 6.94E-05 | 3.25E-05 | 2.01E-05 | 0 | 5.45E-05 |
| d__Bacteria;p__Actinobacteria;c__Acidimicrobiia;o__Acidimicrobiales;f__Acidimicrobiaceae | 0 | 0 | 0 | 0 | 0 | 0 | 0 | 0 | 0 | 0 | 0 | 0 |
| d__Bacteria;p__Actinobacteria;c__Acidimicrobiia;o__IMCC26256;f__IMCC26256 | 0.011318 | 0.006248 | 0.010083 | 0.018686 | 0.009932 | 0.00475 | 0.018435 | 0.012476 | 0.010903 | 0.016746 | 0.014967 | 0.013867 |
| d__Bacteria;p__Actinobacteria;c__Acidimicrobiia;o__Microtrichales;f__Iamiaceae | 0 | 0 | 0 | 0 | 0 | 0 | 0 | 0 | 0.000211 | 0.000241 | 0.000323 | 0 |
| d__Bacteria;p__Actinobacteria;c__Acidimicrobiia;o__Microtrichales;f__Ilumatobacteraceae | 0.000134 | 0 | 0.000184 | 0 | 0.0001 | 0.000225 | 0 | 0.000156 | 0.000292 | 0 | 0.000108 | 0.000145 |
| d__Bacteria;p__Actinobacteria;c__Acidimicrobiia;o__Microtrichales;f__Microtrichaceae | 2.97E-05 | 0 | 0 | 0 | 4.00E-05 | 0 | 0 | 0 | 0 | 0 | 2.16E-05 | 0 |
| d__Bacteria;p__Actinobacteria;c__Acidimicrobiia;o__Microtrichales;f__Microtrichales | 0 | 0 | 0 | 0 | 0 | 0 | 0 | 0 | 0 | 0 | 0 | 0 |
| d__Bacteria;p__Actinobacteria;c__Acidimicrobiia;o__Microtrichales;f__unclassified_Microtrichales | 0.000134 | 0 | 9.91E-05 | 0.000249 | 0.00034 | 0 | 0 | 0 | 0.00039 | 0 | 0 | 0 |
| d__Bacteria;p__Actinobacteria;c__Acidimicrobiia;o__Microtrichales;f__uncultured | 0 | 0 | 0.000255 | 0 | 0.00022 | 0 | 0 | 0.000382 | 0.000162 | 0.000583 | 0 | 0.000672 |
| d__Bacteria;p__Actinobacteria;c__Acidimicrobiia;o__unclassified_Acidimicrobiia;f__unclassified_Acidimicrobiia | 0.000699 | 0.000539 | 0.00017 | 0.001682 | 0.00038 | 0 | 0 | 0.000278 | 4.87E-05 | 0 | 0.000216 | 0 |
| d__Bacteria;p__Actinobacteria;c__Acidimicrobiia;o__uncultured;f__uncultured | 0.012731 | 0.008547 | 0.004079 | 0.038825 | 0.008911 | 0.01415 | 0.002094 | 0.001735 | 0.00078 | 0.001347 | 0.001272 | 0.000691 |
| d__Bacteria;p__Actinobacteria;c__Actinobacteria;o__Bifidobacteriales;f__Bifidobacteriaceae | 0 | 0 | 0 | 6.23E-05 | 0 | 5.00E-05 | 0 | 0 | 6.50E-05 | 0 | 0 | 0 |
| d__Bacteria;p__Actinobacteria;c__Actinobacteria;o__Catenulisporales;f__Actinospicaceae | 0.000193 | 5.39E-05 | 0 | 0.000664 | 0.000561 | 0.0005 | 0.000766 | 0.000486 | 0.00104 | 0.000302 | 0.000151 | 0 |
| d__Bacteria;p__Actinobacteria;c__Actinobacteria;o__Catenulisporales;f__Catenulisporaceae | 0 | 8.98E-05 | 0.000142 | 0 | 0.001021 | 0.000375 | 0.000638 | 0.000816 | 0.000211 | 0.000181 | 0.001617 | 0.000291 |
| d__Bacteria;p__Actinobacteria;c__Actinobacteria;o__Corynebacteriales;f__Corynebacteriaceae | 0 | 0 | 0 | 0 | 0 | 0 | 0 | 0 | 0 | 0 | 0 | 0 |
| d__Bacteria;p__Actinobacteria;c__Actinobacteria;o__Corynebacteriales;f__Dietziaceae | 0 | 0 | 0 | 0 | 0 | 0 | 0 | 0 | 0 | 0 | 0 | 0 |
| d__Bacteria;p__Actinobacteria;c__Actinobacteria;o__Corynebacteriales;f__Mycobacteriaceae | 0.000387 | 0.001329 | 0.001204 | 0.002201 | 0.007529 | 0.003 | 0.000664 | 0.000382 | 0.000552 | 0.000382 | 0.004076 | 0.00109 |
| d__Bacteria;p__Actinobacteria;c__Actinobacteria;o__Corynebacteriales;f__Nocardiaceae | 0 | 0 | 0 | 0 | 6.01E-05 | 0 | 0 | 0 | 0 | 0.000141 | 0.000194 | 0.000418 |
| d__Bacteria;p__Actinobacteria;c__Actinobacteria;o__Corynebacteriales;f__unclassified_Corynebacteriales | 0 | 0 | 0 | 0 | 0 | 0 | 0 | 0 | 0 | 0 | 0 | 0 |
| d__Bacteria;p__Actinobacteria;c__Actinobacteria;o__Frankiales;f__Acidothermaceae | 0.03571 | 0.055428 | 0.050911 | 0.058383 | 0.051763 | 0.0404 | 0.003013 | 0.002499 | 0.000747 | 0.000744 | 0.003472 | 0.002199 |
| d__Bacteria;p__Actinobacteria;c__Actinobacteria;o__Frankiales;f__Cryptosporangiaceae | 0 | 0 | 0 | 0 | 0 | 0 | 0.000153 | 0.000243 | 0.00039 | 0.000261 | 0.000388 | 0.000127 |
| d__Bacteria;p__Actinobacteria;c__Actinobacteria;o__Frankiales;f__Frankiaceae | 0.000178 | 0 | 0.000227 | 0.001516 | 0.001242 | 0.00095 | 0.003907 | 0.001631 | 0.001722 | 0.001267 | 0.002264 | 0.001581 |
| d__Bacteria;p__Actinobacteria;c__Actinobacteria;o__Frankiales;f__Geodermatophilaceae | 0 | 0 | 0 | 0 | 0 | 0 | 0.004724 | 0.005483 | 0.004826 | 0.003176 | 0.005801 | 0.002345 |
| d__Bacteria;p__Actinobacteria;c__Actinobacteria;o__Frankiales;f__Nakamurellaceae | 0 | 0 | 0.000127 | 0 | 0 | 0 | 0 | 0 | 0 | 0 | 0.000582 | 0 |
| d__Bacteria;p__Actinobacteria;c__Actinobacteria;o__Frankiales;f__Sporichthyaceae | 0.001249 | 0.000198 | 0.000212 | 0.000997 | 0.000561 | 0 | 0.004749 | 0.005518 | 0.005508 | 0.004483 | 0.002092 | 0.001399 |
| d__Bacteria;p__Actinobacteria;c__Actinobacteria;o__Frankiales;f__unclassified_Frankiales | 0 | 0 | 8.50E-05 | 0 | 8.01E-05 | 0.00015 | 0.001838 | 0.000711 | 0.000682 | 0.002071 | 0.001553 | 0.001399 |
| d__Bacteria;p__Actinobacteria;c__Actinobacteria;o__Frankiales;f__uncultured | 0 | 0 | 0 | 0 | 0.00012 | 0 | 0.001277 | 0.001458 | 0.000601 | 0.000322 | 0.001143 | 0.000709 |
| d__Bacteria;p__Actinobacteria;c__Actinobacteria;o__Kineosporiales;f__Kineosporiaceae | 0 | 0 | 0.000184 | 0 | 6.01E-05 | 0.00025 | 0.001149 | 0.000607 | 0.000341 | 0.000583 | 0.001229 | 0.000418 |
| d__Bacteria;p__Actinobacteria;c__Actinobacteria;o__Micrococcales;f__AKAU3644 | 0 | 0 | 0 | 0 | 0 | 0 | 0 | 0 | 0 | 0 | 0 | 0 |
| d__Bacteria;p__Actinobacteria;c__Actinobacteria;o__Micrococcales;f__Cellulomonadaceae | 0 | 0 | 0 | 0 | 0 | 0 | 0 | 0 | 0 | 0 | 0 | 0 |
| d__Bacteria;p__Actinobacteria;c__Actinobacteria;o__Micrococcales;f__Dermatophilaceae | 0 | 0 | 0 | 0 | 0 | 0 | 0 | 0 | 0 | 0 | 4.31E-05 | 0 |
| d__Bacteria;p__Actinobacteria;c__Actinobacteria;o__Micrococcales;f__Intrasporangiaceae | 0 | 0 | 0 | 0 | 0 | 0 | 0.003753 | 0.003574 | 0.001219 | 0.002875 | 0.005737 | 0.003108 |
| d__Bacteria;p__Actinobacteria;c__Actinobacteria;o__Micrococcales;f__Microbacteriaceae | 0 | 0 | 0 | 0.000311 | 0.000581 | 0.001925 | 0.006153 | 0.008329 | 0.00273 | 0.00203 | 0.00688 | 0.002744 |
| d__Bacteria;p__Actinobacteria;c__Actinobacteria;o__Micrococcales;f__Micrococcaceae | 0.000565 | 0.000251 | 0.006783 | 0.000394 | 0.0002 | 0 | 0.018001 | 0.044473 | 0.010464 | 0.008182 | 0.009489 | 0.038385 |
| d__Bacteria;p__Actinobacteria;c__Actinobacteria;o__Micrococcales;f__unclassified_Micrococcales | 0 | 0 | 7.08E-05 | 0 | 0 | 0 | 0.000332 | 6.94E-05 | 0 | 0 | 0.000259 | 0.0002 |
| d__Bacteria;p__Actinobacteria;c__Actinobacteria;o__Micromonosporales;f__Micromonosporaceae | 0 | 0 | 0.000198 | 0 | 0.002683 | 0.001875 | 0.004341 | 0.005483 | 0.00364 | 0.002091 | 0.00509 | 0.002435 |
| d__Bacteria;p__Actinobacteria;c__Actinobacteria;o__PeM15;f__PeM15 | 0 | 0 | 0 | 0 | 0 | 0 | 0 | 0 | 0 | 0 | 0 | 0 |
| d__Bacteria;p__Actinobacteria;c__Actinobacteria;o__Propionibacteriales;f__Nocardioidaceae | 0 | 0 | 9.91E-05 | 0 | 0.00024 | 2.50E-05 | 0.003192 | 0.002742 | 0.00325 | 0.004041 | 0.007074 | 0.003017 |
| d__Bacteria;p__Actinobacteria;c__Actinobacteria;o__Propionibacteriales;f__Propionibacteriaceae | 0 | 0 | 0 | 0 | 0 | 0 | 0 | 0 | 0 | 0 | 0 | 7.27E-05 |
| d__Bacteria;p__Actinobacteria;c__Actinobacteria;o__Pseudonocardiales;f__Pseudonocardiaceae | 0.000387 | 0.000305 | 0.000368 | 0.004734 | 0.001221 | 0.0006 | 0.004392 | 0.002603 | 0.00351 | 0.003176 | 0.006944 | 0.003817 |
| d__Bacteria;p__Actinobacteria;c__Actinobacteria;o__Streptomycetales;f__Streptomycetaceae | 0.000342 | 0.000359 | 0.000142 | 0.000457 | 0.006528 | 0.000725 | 0.002553 | 0.001891 | 0.0013 | 0.000925 | 0.00619 | 0.002017 |
| d__Bacteria;p__Actinobacteria;c__Actinobacteria;o__Streptosporangiales;f__Streptosporangiaceae | 0 | 0 | 0 | 0 | 6.01E-05 | 0 | 0.00646 | 0.005848 | 0.006483 | 0.007056 | 0.00399 | 0.002617 |
| d__Bacteria;p__Actinobacteria;c__Actinobacteria;o__Streptosporangiales;f__Thermomonosporaceae | 0 | 7.18E-05 | 0.000142 | 0.000125 | 8.01E-05 | 0.000475 | 0.018486 | 0.01527 | 0.015388 | 0.0077 | 0.00785 | 0.003908 |
| d__Bacteria;p__Actinobacteria;c__Actinobacteria;o__unclassified_Actinobacteria;f__unclassified_Actinobacteria | 0.000134 | 3.59E-05 | 5.66E-05 | 8.30E-05 | 0.00018 | 0.0003 | 0.003089 | 0.001874 | 0.002567 | 0.001488 | 0.001682 | 0.000527 |
| d__Bacteria;p__Actinobacteria;c__Coriobacteriia;o__Coriobacteriales;f__Eggerthellaceae | 0 | 0 | 0 | 0 | 0 | 0 | 0 | 0 | 0 | 0 | 0 | 0 |
| d__Bacteria;p__Actinobacteria;c__MB-A2-108;o__MB-A2-108;f__MB-A2-108 | 0 | 0 | 0.000878 | 0 | 0 | 0 | 0 | 0 | 0 | 0.000181 | 0.000712 | 0.001836 |
| d__Bacteria;p__Actinobacteria;c__Thermoleophilia;o__Gaiellales;f__Gaiellaceae | 0 | 0 | 0 | 0 | 0 | 0 | 0.002681 | 0.002863 | 0.00416 | 0.003056 | 0.004335 | 0.00478 |
| d__Bacteria;p__Actinobacteria;c__Thermoleophilia;o__Gaiellales;f__unclassified_Gaiellales | 0.000223 | 0.000198 | 0.000807 | 0 | 8.01E-05 | 2.50E-05 | 0.000638 | 0.000382 | 0.000292 | 0.000442 | 0.000669 | 0.001145 |
| d__Bacteria;p__Actinobacteria;c__Thermoleophilia;o__Gaiellales;f__uncultured | 0.016717 | 0.008367 | 0.01436 | 0.009613 | 0.007469 | 0.003225 | 0.044223 | 0.04095 | 0.042735 | 0.04089 | 0.044147 | 0.040475 |
| d__Bacteria;p__Actinobacteria;c__Thermoleophilia;o__Solirubrobacterales;f__67-14 | 0.000982 | 0.000126 | 0.002223 | 0.00328 | 0.001882 | 0.000375 | 0.004009 | 0.002273 | 0.002047 | 0.003418 | 0.004594 | 0.003544 |
| d__Bacteria;p__Actinobacteria;c__Thermoleophilia;o__Solirubrobacterales;f__Solirubrobacteraceae | 0.010575 | 0.008403 | 0.008214 | 0.025101 | 0.00797 | 0.006175 | 0.011771 | 0.005986 | 0.005443 | 0.005287 | 0.006384 | 0.005253 |
| d__Bacteria;p__Actinobacteria;c__Thermoleophilia;o__Solirubrobacterales;f__unclassified_Solirubrobacterales | 0.000238 | 0.00097 | 0.000184 | 0 | 0 | 0 | 7.66E-05 | 0 | 0 | 0 | 0.000108 | 0 |
| d__Bacteria;p__Actinobacteria;c__Thermoleophilia;o__unclassified_Thermoleophilia;f__unclassified_Thermoleophilia | 0 | 0 | 0 | 0 | 0 | 0 | 0 | 0 | 0 | 0 | 0 | 0 |
| d__Bacteria;p__Actinobacteria;c__Thermoleophilia;o__uncultured;f__uncultured | 0 | 0 | 0 | 0 | 0 | 0 | 0 | 0 | 0 | 0 | 8.63E-05 | 0.000145 |
| d__Bacteria;p__Actinobacteria;c__unclassified_Actinobacteria;o__unclassified_Actinobacteria;f__unclassified_Actinobacteria | 0 | 0 | 1.42E-05 | 4.15E-05 | 2.00E-05 | 7.50E-05 | 7.66E-05 | 1.74E-05 | 1.62E-05 | 6.03E-05 | 4.31E-05 | 0 |
| d__Bacteria;p__Armatimonadetes;c__Armatimonadia;o__Armatimonadales;f__Armatimonadales | 0.000134 | 3.59E-05 | 0.000269 | 0.000498 | 0.00014 | 0 | 0.00023 | 0.000174 | 0.000926 | 0.000402 | 0.00069 | 0.000418 |
| d__Bacteria;p__Armatimonadetes;c__Armatimonadia;o__Armatimonadales;f__unclassified_Armatimonadales | 0 | 0 | 0 | 0 | 0 | 0 | 0 | 0 | 0 | 0 | 0 | 0 |
| d__Bacteria;p__Armatimonadetes;c__Chthonomonadetes;o__Chthonomonadales;f__Chthonomonadaceae | 0.000625 | 0.000359 | 0.000453 | 0.000561 | 0.00024 | 0.00025 | 0.000204 | 0.000104 | 0.000991 | 0.000623 | 0.000388 | 0.000763 |
| d__Bacteria;p__Armatimonadetes;c__Chthonomonadetes;o__Chthonomonadales;f__Chthonomonadales | 0 | 0 | 7.08E-05 | 0 | 0 | 0 | 0 | 0 | 0 | 0 | 0 | 0.000145 |
| d__Bacteria;p__Armatimonadetes;c__Fimbriimonadia;o__Fimbriimonadales;f__Fimbriimonadaceae | 0.000297 | 0.000108 | 7.08E-05 | 0.000311 | 0.000581 | 0 | 0.00023 | 6.94E-05 | 0.000942 | 0.000684 | 0 | 0.00129 |
| d__Bacteria;p__Armatimonadetes;c__uncultured;o__uncultured;f__uncultured | 0.000625 | 0 | 5.66E-05 | 8.30E-05 | 0 | 0 | 7.66E-05 | 0.000174 | 0.000504 | 0.000503 | 0.000345 | 0.001054 |
| d__Bacteria;p__Atribacteria;c__Caldatribacteriia;o__Caldatribacteriales;f__Caldatribacteriaceae | 0 | 0 | 0 | 0 | 0 | 0 | 5.11E-05 | 0 | 0 | 0 | 0 | 0 |
| d__Bacteria;p__BRC1;c__BRC1;o__BRC1;f__BRC1 | 0 | 0 | 0 | 0 | 4.00E-05 | 0 | 0 | 5.21E-05 | 0.00026 | 0.000181 | 0 | 0.000545 |
| d__Bacteria;p__Bacteroidetes;c__Bacteroidia;o__Bacteroidales;f__Bacteroidaceae | 0 | 0 | 0.00034 | 0 | 0 | 0 | 0 | 0 | 0.000114 | 0 | 0 | 0.000291 |
| d__Bacteria;p__Bacteroidetes;c__Bacteroidia;o__Bacteroidales;f__Dysgonomonadaceae | 0 | 0 | 0 | 0 | 0 | 0 | 0 | 0 | 6.50E-05 | 0 | 0 | 0 |
| d__Bacteria;p__Bacteroidetes;c__Bacteroidia;o__Bacteroidales;f__F082 | 0 | 0 | 0 | 0 | 0 | 0 | 0 | 0 | 0 | 0 | 0 | 0 |
| d__Bacteria;p__Bacteroidetes;c__Bacteroidia;o__Bacteroidales;f__Muribaculaceae | 4.46E-05 | 0 | 0 | 0 | 0 | 0.000175 | 0.000204 | 0.000121 | 0 | 6.03E-05 | 0.000151 | 0 |
| d__Bacteria;p__Bacteroidetes;c__Bacteroidia;o__Bacteroidales;f__Porphyromonadaceae | 0 | 0 | 0 | 0 | 0 | 0 | 0 | 0 | 0 | 0 | 0 | 0 |
| d__Bacteria;p__Bacteroidetes;c__Bacteroidia;o__Bacteroidales;f__Prevotellaceae | 5.95E-05 | 0 | 0.000283 | 0 | 0 | 0 | 0 | 0 | 0 | 0 | 0 | 0 |
| d__Bacteria;p__Bacteroidetes;c__Bacteroidia;o__Bacteroidales;f__Rikenellaceae | 0 | 0 | 0.000453 | 0 | 0 | 0 | 0 | 0 | 0 | 4.02E-05 | 0 | 0 |
| d__Bacteria;p__Bacteroidetes;c__Bacteroidia;o__Bacteroidales;f__SB-5 | 0 | 0 | 0 | 0 | 0 | 0 | 0 | 0 | 0 | 0 | 0 | 0 |
| d__Bacteria;p__Bacteroidetes;c__Bacteroidia;o__Bacteroidales;f__p-251-o5 | 0 | 0 | 0 | 0 | 0 | 0 | 0 | 0 | 0 | 0 | 0 | 0 |
| d__Bacteria;p__Bacteroidetes;c__Bacteroidia;o__Bacteroidales;f__uncultured | 0 | 0 | 0 | 0 | 0 | 0 | 0 | 0 | 0 | 0 | 0 | 0 |
| d__Bacteria;p__Bacteroidetes;c__Bacteroidia;o__Bacteroidetes_VC2.1_Bac22;f__Bacteroidetes_VC2.1_Bac22 | 0 | 0 | 0 | 0 | 0 | 0 | 0 | 0 | 0 | 0 | 0 | 0 |
| d__Bacteria;p__Bacteroidetes;c__Bacteroidia;o__Chitinophagales;f__37-13 | 0.000461 | 0.000413 | 0.000142 | 0.000561 | 0 | 0.000275 | 0.000179 | 0.000416 | 0.000244 | 0.000503 | 0.000259 | 0.000145 |
| d__Bacteria;p__Bacteroidetes;c__Bacteroidia;o__Chitinophagales;f__Chitinophagaceae | 0.007927 | 0.004471 | 0.009219 | 0.008533 | 0.009832 | 0.006375 | 0.008324 | 0.010151 | 0.009311 | 0.009469 | 0.009381 | 0.01076 |
| d__Bacteria;p__Bacteroidetes;c__Bacteroidia;o__Chitinophagales;f__Saprospiraceae | 0 | 0 | 0 | 0 | 0 | 0 | 0 | 0 | 0 | 0 | 0.000151 | 0.000109 |
| d__Bacteria;p__Bacteroidetes;c__Bacteroidia;o__Chitinophagales;f__unclassified_Chitinophagales | 0 | 0 | 0.00126 | 0 | 0.00038 | 0 | 0 | 0 | 0 | 0.000121 | 0 | 0 |
| d__Bacteria;p__Bacteroidetes;c__Bacteroidia;o__Chitinophagales;f__uncultured | 0.001115 | 0.00018 | 0.000793 | 0.000623 | 0.000601 | 0.000275 | 0.00046 | 0.000729 | 0.000747 | 0.000362 | 0.000798 | 0.000891 |
| d__Bacteria;p__Bacteroidetes;c__Bacteroidia;o__Cytophagales;f__Amoebophilaceae | 0 | 0 | 0 | 0 | 0 | 0 | 0 | 0 | 0 | 0 | 0 | 0.000145 |
| d__Bacteria;p__Bacteroidetes;c__Bacteroidia;o__Cytophagales;f__Cytophagaceae | 0 | 0 | 0 | 0.000145 | 4.00E-05 | 0.0001 | 0 | 0 | 0 | 0 | 0 | 0 |
| d__Bacteria;p__Bacteroidetes;c__Bacteroidia;o__Cytophagales;f__Hymenobacteraceae | 0 | 0 | 0 | 0 | 0 | 5.00E-05 | 0 | 0 | 0 | 0 | 0 | 0 |
| d__Bacteria;p__Bacteroidetes;c__Bacteroidia;o__Cytophagales;f__Microscillaceae | 0 | 0.000126 | 0.000467 | 0.000311 | 0.000941 | 0.000175 | 0.000281 | 0.000694 | 0.001592 | 0.002352 | 0.000647 | 0.000582 |
| d__Bacteria;p__Bacteroidetes;c__Bacteroidia;o__Cytophagales;f__Spirosomaceae | 0 | 0 | 0.000113 | 0 | 0 | 7.50E-05 | 0 | 0 | 0 | 0 | 0 | 0 |
| d__Bacteria;p__Bacteroidetes;c__Bacteroidia;o__Flavobacteriales;f__Crocinitomicaceae | 0 | 0 | 0 | 0 | 0 | 0 | 0 | 0 | 0 | 0 | 0 | 0 |
| d__Bacteria;p__Bacteroidetes;c__Bacteroidia;o__Flavobacteriales;f__Cryomorphaceae | 0 | 0 | 0 | 0 | 0 | 0 | 0 | 0 | 0 | 0 | 0 | 0 |
| d__Bacteria;p__Bacteroidetes;c__Bacteroidia;o__Flavobacteriales;f__Flavobacteriaceae | 0 | 0.000108 | 0 | 0 | 0.00012 | 7.50E-05 | 0 | 0.000104 | 3.25E-05 | 0 | 0.00028 | 0 |
| d__Bacteria;p__Bacteroidetes;c__Bacteroidia;o__Flavobacteriales;f__NS9_marine_group | 0 | 0 | 0 | 0 | 0 | 0 | 0 | 0 | 0 | 0 | 0 | 0 |
| d__Bacteria;p__Bacteroidetes;c__Bacteroidia;o__Flavobacteriales;f__Weeksellaceae | 0 | 0 | 5.66E-05 | 0 | 0 | 0 | 0 | 0 | 0.000146 | 0 | 0 | 0 |
| d__Bacteria;p__Bacteroidetes;c__Bacteroidia;o__SM1A07;f__SM1A07 | 0 | 0 | 0 | 0 | 0 | 0 | 0 | 0 | 6.50E-05 | 4.02E-05 | 4.31E-05 | 0 |
| d__Bacteria;p__Bacteroidetes;c__Bacteroidia;o__Sphingobacteriales;f__AKYH767 | 0.000744 | 0.000718 | 0.000878 | 0.000623 | 0.00026 | 0.0001 | 0.000179 | 0.000208 | 0.000292 | 0.000824 | 0.000151 | 0.000345 |
| d__Bacteria;p__Bacteroidetes;c__Bacteroidia;o__Sphingobacteriales;f__CWT_CU03-E12 | 0.002335 | 0.000844 | 0.001175 | 0.001972 | 0.000621 | 0.00145 | 0.000689 | 0.000416 | 0 | 0.000141 | 0.000949 | 0.000182 |
| d__Bacteria;p__Bacteroidetes;c__Bacteroidia;o__Sphingobacteriales;f__KD3-93 | 0 | 0 | 0 | 0.000436 | 0.00026 | 0.000175 | 0 | 0.000104 | 0 | 0 | 0 | 7.27E-05 |
| d__Bacteria;p__Bacteroidetes;c__Bacteroidia;o__Sphingobacteriales;f__Lentimicrobiaceae | 0 | 0 | 0 | 0 | 0 | 0 | 0 | 0 | 0 | 0 | 0 | 0 |
| d__Bacteria;p__Bacteroidetes;c__Bacteroidia;o__Sphingobacteriales;f__LiUU-11-161 | 0 | 0 | 0 | 0 | 0 | 0 | 0 | 0 | 0 | 8.04E-05 | 0 | 0 |
| d__Bacteria;p__Bacteroidetes;c__Bacteroidia;o__Sphingobacteriales;f__NS11-12_marine_group | 0 | 0.000162 | 0 | 0 | 6.01E-05 | 0 | 0 | 0.000208 | 0.000227 | 0.000382 | 0.000453 | 0.0004 |
| d__Bacteria;p__Bacteroidetes;c__Bacteroidia;o__Sphingobacteriales;f__S15-21 | 0.000357 | 0 | 2.83E-05 | 0.000311 | 6.01E-05 | 0 | 0 | 0 | 0 | 0 | 0 | 0 |
| d__Bacteria;p__Bacteroidetes;c__Bacteroidia;o__Sphingobacteriales;f__ST-12K33 | 0 | 0 | 0 | 0 | 0 | 0 | 0 | 0 | 0 | 0 | 0 | 0 |
| d__Bacteria;p__Bacteroidetes;c__Bacteroidia;o__Sphingobacteriales;f__Sphingobacteriaceae | 0.001443 | 0.000198 | 0.000326 | 0.001391 | 0.003224 | 0.0223 | 0.002579 | 0.002481 | 0.00273 | 0.004202 | 0.003968 | 0.001509 |
| d__Bacteria;p__Bacteroidetes;c__Bacteroidia;o__Sphingobacteriales;f__Sphingobacteriales | 7.44E-05 | 0 | 0 | 0 | 0 | 0 | 0 | 0.000174 | 0 | 0 | 0 | 0.000164 |
| d__Bacteria;p__Bacteroidetes;c__Bacteroidia;o__Sphingobacteriales;f__env.OPS_17 | 0.000759 | 0.000359 | 0.000425 | 0.000561 | 0.00032 | 0.0008 | 0.00074 | 0.000295 | 0.001089 | 0.000362 | 0.000453 | 0.0004 |
| d__Bacteria;p__Bacteroidetes;c__Bacteroidia;o__Sphingobacteriales;f__unclassified_Sphingobacteriales | 0 | 0.000162 | 0 | 4.15E-05 | 0 | 0 | 0 | 0 | 4.87E-05 | 4.02E-05 | 0.000151 | 0.000164 |
| d__Bacteria;p__Bacteroidetes;c__Bacteroidia;o__unclassified_Bacteroidia;f__unclassified_Bacteroidia | 0 | 0 | 0 | 4.15E-05 | 0 | 0 | 0 | 0 | 0 | 0 | 4.31E-05 | 0 |
| d__Bacteria;p__Bacteroidetes;c__Ignavibacteria;o__Kryptoniales;f__BSV26 | 0 | 0 | 0 | 0 | 0 | 0 | 0 | 0 | 0 | 4.02E-05 | 0 | 0 |
| d__Bacteria;p__Bacteroidetes;c__Ignavibacteria;o__OPB56;f__OPB56 | 0.00116 | 0.000628 | 0.000198 | 0.000914 | 0.00016 | 5.00E-05 | 0.000638 | 0.000902 | 0.000747 | 0.000322 | 0.000388 | 0.000291 |
| d__Bacteria;p__Bacteroidetes;c__Ignavibacteria;o__SJA-28;f__SJA-28 | 0 | 0 | 9.91E-05 | 0 | 0 | 0 | 0 | 0 | 0 | 0.000402 | 0.000173 | 0.000309 |
| d__Bacteria;p__Chlamydiae;c__Chlamydiae;o__Chlamydiales;f__Parachlamydiaceae | 0.000149 | 0.000736 | 4.25E-05 | 0.000478 | 8.01E-05 | 5.00E-05 | 0.000511 | 0.000312 | 0.000406 | 0.000241 | 0.000345 | 0.000254 |
| d__Bacteria;p__Chlamydiae;c__Chlamydiae;o__Chlamydiales;f__Simkaniaceae | 0.000892 | 0.002119 | 0.000552 | 0.000727 | 0.0003 | 0.000475 | 0.000587 | 0.000468 | 0.0026 | 0.000342 | 0.000173 | 0.000872 |
| d__Bacteria;p__Chlamydiae;c__Chlamydiae;o__Chlamydiales;f__cvE6 | 0.00119 | 0.000593 | 0.000467 | 0.00027 | 6.01E-05 | 0 | 0.000409 | 0.000503 | 0.000504 | 0.00189 | 0.000323 | 0.001072 |
| d__Bacteria;p__Chlamydiae;c__Chlamydiae;o__Chlamydiales;f__unclassified_Chlamydiales | 0 | 0 | 0.000241 | 0 | 0 | 5.00E-05 | 0 | 0 | 0 | 0 | 6.47E-05 | 0.000145 |
| d__Bacteria;p__Chloroflexi;c__AD3;o__AD3;f__AD3 | 0.017461 | 0.030255 | 0.01715 | 0.012955 | 0.003204 | 0.0033 | 0.074276 | 0.092832 | 0.048097 | 0.019158 | 0.026441 | 0.038858 |
| d__Bacteria;p__Chloroflexi;c__Anaerolineae;o__Anaerolineae;f__Anaerolineae | 0 | 0 | 0 | 0 | 0 | 0 | 0 | 0 | 0 | 0 | 0 | 0 |
| d__Bacteria;p__Chloroflexi;c__Anaerolineae;o__Anaerolineales;f__Anaerolineaceae | 0 | 0.000413 | 0.000552 | 0 | 0.00024 | 5.00E-05 | 7.66E-05 | 0.000399 | 0.000666 | 0.001367 | 0.000863 | 0.000963 |
| d__Bacteria;p__Chloroflexi;c__Anaerolineae;o__Caldilineales;f__Caldilineaceae | 0 | 0 | 0 | 0 | 0 | 0 | 0 | 0 | 8.12E-05 | 0 | 0 | 0.0002 |
| d__Bacteria;p__Chloroflexi;c__Anaerolineae;o__RBG-13-54-9;f__RBG-13-54-9 | 0 | 0 | 0.000651 | 0 | 0 | 0 | 0 | 5.21E-05 | 0 | 0 | 0.000884 | 0.000872 |
| d__Bacteria;p__Chloroflexi;c__Anaerolineae;o__SBR1031;f__A4b | 0 | 0 | 0.000326 | 0 | 0.00016 | 0.00015 | 0.000434 | 0.000798 | 0.000731 | 0.000885 | 0.00097 | 0.002508 |
| d__Bacteria;p__Chloroflexi;c__Anaerolineae;o__SBR1031;f__SBR1031 | 0.000134 | 0.000251 | 0.002039 | 4.15E-05 | 0.001041 | 0.000375 | 0.000562 | 0.000226 | 0.001917 | 0.003719 | 0.001143 | 0.002036 |
| d__Bacteria;p__Chloroflexi;c__Anaerolineae;o__SBR1031;f__unclassified_SBR1031 | 0 | 0 | 0 | 0 | 0 | 0 | 0.000511 | 8.68E-05 | 0 | 0 | 0 | 0 |
| d__Bacteria;p__Chloroflexi;c__Anaerolineae;o__unclassified_Anaerolineae;f__unclassified_Anaerolineae | 0 | 0 | 0 | 0 | 0 | 0 | 0 | 0 | 0 | 0 | 0 | 0 |
| d__Bacteria;p__Chloroflexi;c__Chloroflexia;o__Chloroflexales;f__Chloroflexaceae | 0 | 0 | 0 | 0 | 0 | 0 | 0 | 0 | 0 | 0 | 0 | 0 |
| d__Bacteria;p__Chloroflexi;c__Chloroflexia;o__Chloroflexales;f__Roseiflexaceae | 0 | 0 | 0 | 0 | 0 | 5.00E-05 | 7.66E-05 | 0 | 0 | 6.03E-05 | 0 | 0 |
| d__Bacteria;p__Chloroflexi;c__Chloroflexia;o__Elev-1554;f__Elev-1554 | 7.44E-05 | 7.18E-05 | 0.000227 | 0 | 0 | 0 | 0.000485 | 0.000226 | 0.000341 | 0.000221 | 0 | 0.000109 |
| d__Bacteria;p__Chloroflexi;c__Chloroflexia;o__Kallotenuales;f__AKIW781 | 0 | 0 | 0 | 0 | 0 | 0 | 0 | 0 | 0 | 0 | 0 | 5.45E-05 |
| d__Bacteria;p__Chloroflexi;c__Chloroflexia;o__Thermomicrobiales;f__AKYG1722 | 0 | 0 | 0 | 0 | 0 | 0 | 0 | 8.68E-05 | 0 | 0 | 0.000561 | 0.000563 |
| d__Bacteria;p__Chloroflexi;c__Chloroflexia;o__Thermomicrobiales;f__JG30-KF-CM45 | 0 | 0 | 0 | 0 | 0 | 0 | 0.00097 | 0.000659 | 0.000552 | 0.000804 | 0.000755 | 0.000909 |
| d__Bacteria;p__Chloroflexi;c__Chloroflexia;o__Thermomicrobiales;f__Thermomicrobiaceae | 0 | 0 | 0 | 0 | 0 | 0 | 0.002426 | 0.003054 | 0.00247 | 0.001588 | 0.000625 | 0.000345 |
| d__Bacteria;p__Chloroflexi;c__Dehalococcoidia;o__S085;f__S085 | 0.000134 | 0.000323 | 0.000113 | 0 | 0.00026 | 0.000275 | 0.001864 | 0.001527 | 0.001495 | 0.002231 | 0.002588 | 0.003253 |
| d__Bacteria;p__Chloroflexi;c__Dehalococcoidia;o__SAR202_clade;f__SAR202_clade | 0 | 3.59E-05 | 0 | 0 | 0 | 0 | 0 | 0 | 0 | 0.000101 | 0 | 0 |
| d__Bacteria;p__Chloroflexi;c__Dehalococcoidia;o__unclassified_Dehalococcoidia;f__unclassified_Dehalococcoidia | 0 | 0 | 0 | 0 | 0 | 0 | 0 | 0 | 0 | 0 | 0 | 0 |
| d__Bacteria;p__Chloroflexi;c__Gitt-GS-136;o__Gitt-GS-136;f__Gitt-GS-136 | 0 | 0 | 0 | 0 | 0 | 0 | 0.000383 | 0.000174 | 0 | 0 | 0.000561 | 0.002345 |
| d__Bacteria;p__Chloroflexi;c__JG30-KF-CM66;o__JG30-KF-CM66;f__JG30-KF-CM66 | 0.003793 | 0.002639 | 0.003526 | 0.001744 | 0.001061 | 0.00045 | 0.002732 | 0.002707 | 0.002047 | 0.003578 | 0.003429 | 0.00369 |
| d__Bacteria;p__Chloroflexi;c__KD4-96;o__KD4-96;f__KD4-96 | 0.003227 | 0.004273 | 0.006882 | 0.001017 | 0.001842 | 0.0005 | 0.007354 | 0.008121 | 0.006353 | 0.021249 | 0.022106 | 0.021465 |
| d__Bacteria;p__Chloroflexi;c__Ktedonobacteria;o__B10-SB3A;f__B10-SB3A | 0 | 0 | 0 | 0 | 0 | 0 | 0 | 8.68E-05 | 0 | 0 | 0 | 0 |
| d__Bacteria;p__Chloroflexi;c__Ktedonobacteria;o__B12-WMSP1;f__B12-WMSP1 | 4.46E-05 | 0 | 0.000411 | 0.000228 | 0 | 0 | 0 | 8.68E-05 | 0 | 0 | 0 | 0 |
| d__Bacteria;p__Chloroflexi;c__Ktedonobacteria;o__C0119;f__C0119 | 0 | 0 | 0 | 0 | 0 | 0 | 0.000613 | 0.000295 | 0.00026 | 0.000744 | 0.001035 | 0.0004 |
| d__Bacteria;p__Chloroflexi;c__Ktedonobacteria;o__Ktedonobacterales;f__JG30-KF-AS9 | 0.001026 | 0.003519 | 0.002337 | 8.30E-05 | 0.002063 | 0.000925 | 0.000128 | 0.000399 | 0.000406 | 0.000302 | 0.000194 | 0.000309 |
| d__Bacteria;p__Chloroflexi;c__Ktedonobacteria;o__Ktedonobacterales;f__Ktedonobacteraceae | 0.022532 | 0.015783 | 0.063458 | 0.001453 | 0.002283 | 0.00175 | 0.00337 | 0.002256 | 0.002161 | 0.004463 | 0.001553 | 0.001145 |
| d__Bacteria;p__Chloroflexi;c__OLB14;o__OLB14;f__OLB14 | 0 | 0 | 0 | 0 | 0 | 0 | 0.000562 | 0.000954 | 0.001186 | 0.000824 | 0.000561 | 0.000981 |
| d__Bacteria;p__Chloroflexi;c__P2-11E;o__P2-11E;f__P2-11E | 0 | 0 | 0 | 0 | 0 | 0 | 0 | 0 | 0 | 0 | 4.31E-05 | 0.000127 |
| d__Bacteria;p__Chloroflexi;c__SHA-26;o__SHA-26;f__SHA-26 | 0 | 0 | 0 | 0 | 0 | 0 | 0 | 0 | 8.12E-05 | 0 | 0 | 0 |
| d__Bacteria;p__Chloroflexi;c__TK10;o__TK10;f__TK10 | 0.003421 | 0.00255 | 0.008327 | 0.000893 | 0.002103 | 0.00065 | 0.004851 | 0.004824 | 0.003217 | 0.003176 | 0.004184 | 0.005798 |
| d__Bacteria;p__Chloroflexi;c__unclassified_Chloroflexi;o__unclassified_Chloroflexi;f__unclassified_Chloroflexi | 0 | 0 | 0 | 4.15E-05 | 0 | 0 | 0 | 0 | 6.50E-05 | 0.000221 | 4.31E-05 | 0 |
| d__Bacteria;p__Cyanobacteria;c__Melainabacteria;o__Obscuribacterales;f__Obscuribacterales | 0.002186 | 0.000844 | 0.001459 | 0.002159 | 0.00036 | 0.00035 | 0.000204 | 0.000833 | 0.000504 | 0.000764 | 0.000453 | 0.000254 |
| d__Bacteria;p__Cyanobacteria;c__Melainabacteria;o__Vampirovibrionales;f__Vampirovibrionales | 4.46E-05 | 0 | 7.08E-05 | 8.30E-05 | 6.01E-05 | 0 | 0 | 0 | 9.75E-05 | 0.000663 | 0 | 0 |
| d__Bacteria;p__Cyanobacteria;c__Oxyphotobacteria;o__Nostocales;f__Phormidiaceae | 0 | 0 | 0 | 0 | 0 | 0 | 0 | 0 | 0 | 0 | 0 | 0 |
| d__Bacteria;p__Cyanobacteria;c__Oxyphotobacteria;o__Synechococcales;f__Cyanobiaceae | 0 | 0 | 0 | 0 | 0.00014 | 0 | 0 | 0 | 0 | 0 | 0 | 0 |
| d__Bacteria;p__Cyanobacteria;c__Sericytochromatia;o__Sericytochromatia;f__Sericytochromatia | 0.000357 | 7.18E-05 | 0 | 0.000145 | 0.00018 | 0.000175 | 0 | 0.000139 | 0.000146 | 0.000241 | 0.000108 | 0 |
| d__Bacteria;p__Deinococcus-Thermus;c__Deinococci;o__Thermales;f__Thermaceae | 8.92E-05 | 0.000126 | 0.000963 | 0 | 0 | 0 | 0 | 0 | 0 | 0 | 6.47E-05 | 0.000109 |
| d__Bacteria;p__Dependentiae;c__Babeliae;o__Babeliales;f__Babeliaceae | 0.000431 | 0.000215 | 4.25E-05 | 0 | 0 | 0 | 0 | 0.000243 | 0 | 0 | 0 | 5.45E-05 |
| d__Bacteria;p__Dependentiae;c__Babeliae;o__Babeliales;f__Babeliales | 0.000446 | 0.001041 | 0.000751 | 0.000145 | 0.0002 | 0.000125 | 0 | 0 | 0.00052 | 0.000382 | 0.000367 | 0.000309 |
| d__Bacteria;p__Dependentiae;c__Babeliae;o__Babeliales;f__UBA12409 | 0.000164 | 7.18E-05 | 0.000411 | 0.000353 | 0.00022 | 0 | 5.11E-05 | 0.001145 | 0.000747 | 0.000623 | 0.001057 | 0.001381 |
| d__Bacteria;p__Dependentiae;c__Babeliae;o__Babeliales;f__UBA12411 | 0 | 0 | 0 | 0 | 0 | 0 | 0 | 0 | 0 | 0 | 0 | 0 |
| d__Bacteria;p__Dependentiae;c__Babeliae;o__Babeliales;f__Vermiphilaceae | 0.002722 | 0.002496 | 0.000581 | 0.002741 | 0.000801 | 0.0006 | 0.00217 | 0.002724 | 0.007247 | 0.005227 | 0.003451 | 0.005343 |
| d__Bacteria;p__Dependentiae;c__Babeliae;o__Babeliales;f__unclassified_Babeliales | 0.000372 | 0.000144 | 0.000411 | 0.000623 | 0 | 7.50E-05 | 0 | 0.000503 | 0.002031 | 0.002312 | 0.003019 | 0.002908 |
| d__Bacteria;p__Elusimicrobia;c__Elusimicrobia;o__Elusimicrobia;f__Elusimicrobia | 2.97E-05 | 0.000359 | 0.000184 | 8.30E-05 | 0 | 0 | 0 | 0 | 0 | 0 | 0 | 0 |
| d__Bacteria;p__Elusimicrobia;c__Elusimicrobia;o__FCPU453;f__FCPU453 | 0.000283 | 0.000162 | 0.000156 | 0 | 0 | 0 | 0 | 0 | 0 | 0 | 0 | 0 |
| d__Bacteria;p__Elusimicrobia;c__Elusimicrobia;o__Lineage_IV;f__Lineage_IV | 0.001443 | 0.000862 | 0.000496 | 0.00054 | 0.000441 | 0.000525 | 0.00046 | 0.000243 | 4.87E-05 | 6.03E-05 | 0.000237 | 5.45E-05 |
| d__Bacteria;p__Elusimicrobia;c__Elusimicrobia;o__MVP-88;f__MVP-88 | 0 | 0 | 0 | 0 | 0 | 0 | 0 | 0 | 4.87E-05 | 0 | 0 | 0 |
| d__Bacteria;p__Elusimicrobia;c__Elusimicrobia;o__unclassified_Elusimicrobia;f__unclassified_Elusimicrobia | 0 | 0 | 0 | 0 | 0 | 0 | 0 | 0 | 3.25E-05 | 0 | 0 | 0 |
| d__Bacteria;p__Elusimicrobia;c__Lineage_IIa;o__Lineage_IIa;f__Lineage_IIa | 0.003376 | 0.002244 | 0.004376 | 0.003675 | 0.001542 | 0.0004 | 0.00046 | 0.000538 | 0.000471 | 0.001206 | 0.000647 | 0.000654 |
| d__Bacteria;p__Elusimicrobia;c__Lineage_IIb;o__Lineage_IIb;f__Lineage_IIb | 0.000149 | 0.000215 | 5.66E-05 | 0.000125 | 0 | 0 | 5.11E-05 | 8.68E-05 | 6.50E-05 | 8.04E-05 | 6.47E-05 | 5.45E-05 |
| d__Bacteria;p__Elusimicrobia;c__Lineage_IIc;o__Lineage_IIc;f__Lineage_IIc | 0 | 0 | 0 | 0 | 0 | 0 | 0 | 0 | 0 | 0 | 0 | 0 |
| d__Bacteria;p__Entotheonellaeota;c__Entotheonellia;o__Entotheonellales;f__Entotheonellaceae | 0 | 0 | 0 | 0 | 0 | 0 | 0 | 0 | 0 | 0 | 0 | 0 |
| d__Bacteria;p__FBP;c__FBP;o__FBP;f__FBP | 0 | 0 | 0 | 0 | 0 | 0 | 5.11E-05 | 0 | 0.000146 | 8.04E-05 | 0 | 0.0002 |
| d__Bacteria;p__FCPU426;c__FCPU426;o__FCPU426;f__FCPU426 | 0.000654 | 0.000251 | 0.000198 | 0.000561 | 0.000481 | 0.000725 | 0.000204 | 0.000399 | 0.000211 | 0.000101 | 0 | 0.000109 |
| d__Bacteria;p__Fibrobacteres;c__Fibrobacteria;o__Fibrobacterales;f__Fibrobacteraceae | 8.92E-05 | 0 | 0 | 0 | 0 | 0 | 0.000383 | 0.000208 | 0.000114 | 0.000543 | 0.000194 | 0.000145 |
| d__Bacteria;p__Firmicutes;c__Bacilli;o__Bacillales;f__Alicyclobacillaceae | 0 | 0 | 0 | 8.30E-05 | 0 | 0 | 0.011286 | 0.011313 | 0.009668 | 0.004181 | 0.002653 | 0.002108 |
| d__Bacteria;p__Firmicutes;c__Bacilli;o__Bacillales;f__Bacillaceae | 0 | 5.39E-05 | 0.000439 | 0.000208 | 0.0001 | 0 | 0.035976 | 0.028769 | 0.026307 | 0.017068 | 0.018138 | 0.021792 |
| d__Bacteria;p__Firmicutes;c__Bacilli;o__Bacillales;f__Paenibacillaceae | 0.000565 | 0 | 8.50E-05 | 0.000332 | 0.000541 | 0 | 0.011796 | 0.012615 | 0.01321 | 0.006574 | 0.006384 | 0.00587 |
| d__Bacteria;p__Firmicutes;c__Bacilli;o__Bacillales;f__Pasteuriaceae | 0 | 0 | 0 | 0 | 0 | 0 | 0 | 0 | 0 | 0 | 0 | 0 |
| d__Bacteria;p__Firmicutes;c__Bacilli;o__Bacillales;f__Planococcaceae | 5.95E-05 | 0 | 0.000269 | 0.000644 | 0 | 0.00065 | 0.00429 | 0.004078 | 0.001966 | 0.000543 | 0 | 0.000909 |
| d__Bacteria;p__Firmicutes;c__Bacilli;o__Bacillales;f__Sporolactobacillaceae | 0 | 0 | 0 | 0 | 0 | 0 | 0.00526 | 0.005153 | 0.006825 | 0.005468 | 0.002566 | 0.003471 |
| d__Bacteria;p__Firmicutes;c__Bacilli;o__Bacillales;f__Staphylococcaceae | 0.000208 | 0 | 0 | 0 | 0 | 0.000175 | 0 | 0 | 0 | 0 | 0.000431 | 0.000145 |
| d__Bacteria;p__Firmicutes;c__Bacilli;o__Bacillales;f__Thermoactinomycetaceae | 0 | 0 | 0 | 6.23E-05 | 0 | 0 | 0.00023 | 0.000121 | 0.000731 | 0.000261 | 0.000108 | 0.000145 |
| d__Bacteria;p__Firmicutes;c__Bacilli;o__Bacillales;f__unclassified_Bacillales | 0 | 0 | 0 | 0 | 0 | 0 | 0.000332 | 0.000104 | 0.00026 | 0 | 8.63E-05 | 0.000145 |
| d__Bacteria;p__Firmicutes;c__Bacilli;o__Lactobacillales;f__Aerococcaceae | 0 | 0 | 0 | 0 | 0 | 0 | 0 | 0 | 0 | 0 | 6.47E-05 | 0.000145 |
| d__Bacteria;p__Firmicutes;c__Bacilli;o__Lactobacillales;f__Carnobacteriaceae | 0 | 0 | 0 | 0 | 0 | 0 | 0 | 0 | 0 | 0 | 0 | 0 |
| d__Bacteria;p__Firmicutes;c__Bacilli;o__Lactobacillales;f__Enterococcaceae | 5.95E-05 | 0 | 0 | 0 | 0 | 0 | 0 | 0 | 6.50E-05 | 0 | 0 | 0.000109 |
| d__Bacteria;p__Firmicutes;c__Bacilli;o__Lactobacillales;f__Lactobacillaceae | 0.000297 | 0 | 0.000283 | 0 | 0 | 0.0001 | 0 | 0.001353 | 0.002096 | 0.000684 | 0.000108 | 0.000345 |
| d__Bacteria;p__Firmicutes;c__Bacilli;o__Lactobacillales;f__Leuconostocaceae | 0 | 0 | 0 | 0 | 0 | 0 | 0 | 0 | 0 | 0.000382 | 0 | 0 |
| d__Bacteria;p__Firmicutes;c__Bacilli;o__Lactobacillales;f__Streptococcaceae | 0 | 8.98E-05 | 0 | 0 | 0 | 0.000175 | 0 | 0 | 0 | 0 | 0 | 0.000182 |
| d__Bacteria;p__Firmicutes;c__Bacilli;o__unclassified_Bacilli;f__unclassified_Bacilli | 0 | 0 | 0 | 0 | 0 | 7.50E-05 | 0.000204 | 0.000156 | 0.000146 | 4.02E-05 | 0.000216 | 3.63E-05 |
| d__Bacteria;p__Firmicutes;c__Clostridia;o__Clostridiales;f__Caldicoprobacteraceae | 0 | 0 | 0 | 0 | 0 | 0 | 0 | 0 | 8.12E-05 | 0 | 0 | 0 |
| d__Bacteria;p__Firmicutes;c__Clostridia;o__Clostridiales;f__Christensenellaceae | 0 | 0 | 0 | 0 | 0 | 0 | 7.66E-05 | 0 | 0 | 0 | 0 | 0 |
| d__Bacteria;p__Firmicutes;c__Clostridia;o__Clostridiales;f__Clostridiaceae_1 | 0 | 0 | 5.66E-05 | 0.000311 | 8.01E-05 | 0 | 0.001813 | 0.00203 | 0.002047 | 0.000684 | 0.001467 | 0.000927 |
| d__Bacteria;p__Firmicutes;c__Clostridia;o__Clostridiales;f__Family_XI | 0 | 0 | 0 | 0 | 0 | 0 | 0 | 0 | 9.75E-05 | 0 | 0 | 0 |
| d__Bacteria;p__Firmicutes;c__Clostridia;o__Clostridiales;f__Family_XIII | 0 | 0 | 0 | 0 | 0 | 0 | 0 | 0 | 0 | 0 | 0 | 0 |
| d__Bacteria;p__Firmicutes;c__Clostridia;o__Clostridiales;f__Family_XVII | 0 | 0 | 0 | 0 | 0 | 0 | 0 | 0.000243 | 0.000114 | 4.02E-05 | 0 | 0 |
| d__Bacteria;p__Firmicutes;c__Clostridia;o__Clostridiales;f__Family_XVIII | 0 | 0 | 0 | 0 | 0 | 0 | 0 | 0 | 0.000114 | 0 | 6.47E-05 | 0 |
| d__Bacteria;p__Firmicutes;c__Clostridia;o__Clostridiales;f__Gracilibacteraceae | 0 | 0 | 0 | 0 | 0 | 0 | 0.000102 | 6.94E-05 | 0 | 6.03E-05 | 0 | 0 |
| d__Bacteria;p__Firmicutes;c__Clostridia;o__Clostridiales;f__Heliobacteriaceae | 0 | 0 | 0 | 0 | 0 | 0 | 0 | 0.000156 | 0.00065 | 0 | 0 | 0 |
| d__Bacteria;p__Firmicutes;c__Clostridia;o__Clostridiales;f__Lachnospiraceae | 8.92E-05 | 0.000269 | 0.001232 | 0 | 0.00018 | 7.50E-05 | 0.000153 | 3.47E-05 | 6.50E-05 | 0 | 0.000151 | 7.27E-05 |
| d__Bacteria;p__Firmicutes;c__Clostridia;o__Clostridiales;f__Peptococcaceae | 0 | 0 | 0 | 0 | 0 | 0 | 0.000128 | 0.000191 | 9.75E-05 | 0.000121 | 0.000669 | 0.000327 |
| d__Bacteria;p__Firmicutes;c__Clostridia;o__Clostridiales;f__Peptostreptococcaceae | 0 | 0 | 0 | 0 | 0 | 0 | 0.000306 | 0.00033 | 0.000276 | 6.03E-05 | 0 | 0 |
| d__Bacteria;p__Firmicutes;c__Clostridia;o__Clostridiales;f__Ruminococcaceae | 0.000268 | 0 | 0.001062 | 0 | 0 | 0.000475 | 0.000817 | 0.000121 | 0.000179 | 8.04E-05 | 0.000625 | 0.000473 |
| d__Bacteria;p__Firmicutes;c__Clostridia;o__Clostridiales;f__Syntrophomonadaceae | 0 | 0 | 0 | 0 | 0 | 0 | 7.66E-05 | 0 | 0 | 0 | 0 | 0 |
| d__Bacteria;p__Firmicutes;c__Clostridia;o__Clostridiales;f__unclassified_Clostridiales | 0 | 0 | 0 | 0 | 6.01E-05 | 0 | 0 | 0 | 0 | 0 | 0 | 0 |
| d__Bacteria;p__Firmicutes;c__Clostridia;o__DTU014;f__DTU014 | 0 | 0 | 0 | 0 | 0 | 0 | 0 | 0 | 8.12E-05 | 0 | 0 | 0 |
| d__Bacteria;p__Firmicutes;c__Clostridia;o__unclassified_Clostridia;f__unclassified_Clostridia | 0 | 3.59E-05 | 0 | 0 | 0 | 0 | 0 | 0.000121 | 0 | 0 | 0 | 0 |
| d__Bacteria;p__Firmicutes;c__Erysipelotrichia;o__Erysipelotrichales;f__Erysipelotrichaceae | 0.00058 | 8.98E-05 | 9.91E-05 | 0.000457 | 0.000541 | 0.00015 | 0 | 0 | 0.000211 | 0 | 0.000453 | 0.000436 |
| d__Bacteria;p__Firmicutes;c__Limnochordia;o__Limnochordales;f__Limnochordaceae | 0 | 0 | 0 | 0 | 0 | 0 | 0 | 0 | 0 | 0 | 8.63E-05 | 0 |
| d__Bacteria;p__Firmicutes;c__Negativicutes;o__Selenomonadales;f__Veillonellaceae | 0 | 0 | 0 | 0 | 0 | 0 | 7.66E-05 | 0 | 0 | 0 | 0 | 0 |
| d__Bacteria;p__Firmicutes;c__unclassified_Firmicutes;o__unclassified_Firmicutes;f__unclassified_Firmicutes | 0 | 0 | 0.000255 | 6.23E-05 | 0 | 0 | 0 | 6.94E-05 | 0 | 0.000261 | 0.000302 | 0 |
| d__Bacteria;p__GAL15;c__GAL15;o__GAL15;f__GAL15 | 0.000149 | 0.00061 | 0.003994 | 0.000208 | 6.01E-05 | 0 | 0 | 0 | 0 | 0 | 0 | 5.45E-05 |
| d__Bacteria;p__Gemmatimonadetes;c__BD2-11_terrestrial_group;o__BD2-11_terrestrial_group;f__BD2-11_terrestrial_group | 0 | 0 | 0 | 0 | 0 | 0 | 0 | 0.000104 | 0.000325 | 0.000322 | 0 | 7.27E-05 |
| d__Bacteria;p__Gemmatimonadetes;c__Gemmatimonadetes;o__Gemmatimonadales;f__Gemmatimonadaceae | 0.016836 | 0.012317 | 0.018552 | 0.009509 | 0.008651 | 0.009125 | 0.062454 | 0.084017 | 0.092246 | 0.070925 | 0.063859 | 0.045437 |
| d__Bacteria;p__Gemmatimonadetes;c__Longimicrobia;o__Longimicrobiales;f__Longimicrobiaceae | 0 | 0 | 0 | 0 | 0 | 0 | 0 | 0 | 0 | 0 | 0 | 3.63E-05 |
| d__Bacteria;p__Gemmatimonadetes;c__S0134_terrestrial_group;o__S0134_terrestrial_group;f__S0134_terrestrial_group | 0 | 0 | 0 | 0 | 0 | 0 | 0 | 0.000121 | 0 | 0.000121 | 0.000108 | 9.09E-05 |
| d__Bacteria;p__Hydrogenedentes;c__Hydrogenedentia;o__Hydrogenedentiales;f__Hydrogenedensaceae | 0 | 0 | 0 | 0 | 0 | 0 | 0 | 0 | 0 | 0.000181 | 0 | 0 |
| d__Bacteria;p__Latescibacteria;c__Latescibacteria;o__Latescibacteria;f__Latescibacteria | 0 | 0 | 7.08E-05 | 0 | 0 | 0 | 0.000102 | 0 | 3.25E-05 | 0.000583 | 0.000453 | 0.001636 |
| d__Bacteria;p__Margulisbacteria;c__Margulisbacteria;o__Margulisbacteria;f__Margulisbacteria | 0 | 0 | 0 | 0 | 0 | 0 | 0 | 0 | 0 | 0 | 0 | 0 |
| d__Bacteria;p__Nitrospirae;c__Nitrospira;o__Nitrospirales;f__Nitrospiraceae | 0.000297 | 0.000305 | 0.00364 | 0.000291 | 6.01E-05 | 0 | 0.00023 | 0.000139 | 0.000504 | 0.001568 | 0.001531 | 0.001927 |
| d__Bacteria;p__Omnitrophicaeota;c__Omnitrophia;o__Omnitrophales;f__Omnitrophaceae | 0 | 0 | 0 | 0 | 0 | 0 | 0 | 0 | 0 | 0 | 0 | 0 |
| d__Bacteria;p__Omnitrophicaeota;c__Omnitrophicaeota;o__Omnitrophicaeota;f__Omnitrophicaeota | 2.97E-05 | 0.000108 | 0.000113 | 0 | 0 | 0 | 0 | 0 | 0 | 0.000101 | 0 | 0 |
| d__Bacteria;p__Patescibacteria;c__ABY1;o__Candidatus_Kerfeldbacteria;f__Candidatus_Kerfeldbacteria | 0 | 0 | 0 | 0 | 0 | 0 | 0 | 0 | 0 | 0 | 0 | 0 |
| d__Bacteria;p__Patescibacteria;c__ABY1;o__Candidatus_Magasanikbacteria;f__Candidatus_Magasanikbacteria | 0 | 0 | 0 | 0 | 0 | 0 | 0.000128 | 0 | 0 | 0 | 0 | 9.09E-05 |
| d__Bacteria;p__Patescibacteria;c__ABY1;o__unclassified_ABY1;f__unclassified_ABY1 | 0 | 0 | 0 | 0 | 0 | 0 | 0 | 0 | 0 | 4.02E-05 | 0 | 0 |
| d__Bacteria;p__Patescibacteria;c__Berkelbacteria;o__Berkelbacteria;f__Berkelbacteria | 0.000164 | 0 | 0 | 4.15E-05 | 4.00E-05 | 7.50E-05 | 0.002834 | 0.002429 | 0.008953 | 0.008021 | 0.001553 | 0.002944 |
| d__Bacteria;p__Patescibacteria;c__Gracilibacteria;o__Candidatus_Peregrinibacteria;f__Candidatus_Peregrinibacteria | 0 | 0.000108 | 0 | 0 | 0 | 0 | 0 | 0 | 0 | 0 | 0.000129 | 0 |
| d__Bacteria;p__Patescibacteria;c__Gracilibacteria;o__Candidatus_Peribacteria;f__Candidatus_Peribacteria | 0 | 0 | 0.000113 | 0 | 0 | 0 | 0 | 0.000104 | 4.87E-05 | 0.000281 | 6.47E-05 | 3.63E-05 |
| d__Bacteria;p__Patescibacteria;c__Gracilibacteria;o__Gracilibacteria;f__Gracilibacteria | 0 | 0 | 0 | 0 | 0 | 0 | 0 | 0 | 0.000569 | 0 | 0 | 0 |
| d__Bacteria;p__Patescibacteria;c__Kazania;o__Kazania;f__Kazania | 0 | 0 | 0 | 0 | 0 | 0 | 0 | 0 | 0.000309 | 0.000322 | 0.000323 | 0 |
| d__Bacteria;p__Patescibacteria;c__Microgenomatia;o__Candidatus_Daviesbacteria;f__Candidatus_Daviesbacteria | 0 | 0 | 0 | 0 | 0 | 0 | 0 | 0 | 6.50E-05 | 0 | 0 | 0 |
| d__Bacteria;p__Patescibacteria;c__Microgenomatia;o__Candidatus_Levybacteria;f__Candidatus_Levybacteria | 0 | 0.000126 | 0.000212 | 0 | 0.00018 | 0 | 0 | 0 | 0.00013 | 0 | 0.000129 | 0.000182 |
| d__Bacteria;p__Patescibacteria;c__Microgenomatia;o__Candidatus_Pacebacteria;f__Candidatus_Pacebacteria | 0 | 0 | 0 | 0 | 0 | 0 | 0 | 0 | 0 | 0.000221 | 4.31E-05 | 0.000236 |
| d__Bacteria;p__Patescibacteria;c__Microgenomatia;o__Candidatus_Woesebacteria;f__Candidatus_Woesebacteria | 0 | 0 | 5.66E-05 | 0 | 0 | 0 | 0.000153 | 3.47E-05 | 3.25E-05 | 4.02E-05 | 4.31E-05 | 0.000254 |
| d__Bacteria;p__Patescibacteria;c__Microgenomatia;o__Candidatus_Woykebacteria;f__Candidatus_Woykebacteria | 0 | 0 | 0 | 0 | 0 | 0 | 0 | 0 | 0 | 0 | 0 | 0 |
| d__Bacteria;p__Patescibacteria;c__Microgenomatia;o__Microgenomatia;f__Microgenomatia | 0 | 0 | 0 | 0 | 0 | 0 | 0 | 0 | 0 | 0 | 0 | 0 |
| d__Bacteria;p__Patescibacteria;c__Microgenomatia;o__unclassified_Microgenomatia;f__unclassified_Microgenomatia | 0 | 0 | 0 | 0 | 0 | 0 | 0 | 0 | 0 | 0 | 0 | 0 |
| d__Bacteria;p__Patescibacteria;c__Parcubacteria;o__Candidatus_Adlerbacteria;f__Candidatus_Adlerbacteria | 4.46E-05 | 0 | 0 | 0 | 0 | 0 | 0 | 0 | 0 | 0 | 0 | 0 |
| d__Bacteria;p__Patescibacteria;c__Parcubacteria;o__Candidatus_Azambacteria;f__Candidatus_Azambacteria | 0.000535 | 0 | 8.50E-05 | 0 | 0 | 0 | 0 | 0 | 0 | 0 | 0 | 3.63E-05 |
| d__Bacteria;p__Patescibacteria;c__Parcubacteria;o__Candidatus_Jorgensenbacteria;f__Candidatus_Jorgensenbacteria | 0.000744 | 0.000503 | 0.001175 | 0.000768 | 0.00014 | 0.000375 | 0.001277 | 0.001249 | 0.001462 | 0.001749 | 0.001359 | 0.001018 |
| d__Bacteria;p__Patescibacteria;c__Parcubacteria;o__Candidatus_Kaiserbacteria;f__Candidatus_Kaiserbacteria | 0.000178 | 0 | 0.000241 | 0 | 0.00012 | 0.000325 | 0.000945 | 0.000364 | 0.000731 | 0.000885 | 0.000173 | 0.000345 |
| d__Bacteria;p__Patescibacteria;c__Parcubacteria;o__Candidatus_Liptonbacteria;f__Candidatus_Liptonbacteria | 0 | 0 | 0 | 0 | 0 | 0 | 0 | 0 | 0 | 0 | 0 | 0 |
| d__Bacteria;p__Patescibacteria;c__Parcubacteria;o__Candidatus_Moranbacteria;f__Candidatus_Moranbacteria | 0 | 0 | 0 | 0 | 0 | 0 | 0 | 0 | 0 | 0 | 0 | 7.27E-05 |
| d__Bacteria;p__Patescibacteria;c__Parcubacteria;o__Candidatus_Nomurabacteria;f__Candidatus_Nomurabacteria | 2.97E-05 | 0 | 0 | 0 | 0 | 5.00E-05 | 0 | 0 | 0 | 0 | 0 | 0 |
| d__Bacteria;p__Patescibacteria;c__Parcubacteria;o__Candidatus_Staskawiczbacteria;f__Candidatus_Staskawiczbacteria | 0 | 0 | 7.08E-05 | 0 | 0 | 0 | 0 | 0 | 4.87E-05 | 6.03E-05 | 0 | 0 |
| d__Bacteria;p__Patescibacteria;c__Parcubacteria;o__Candidatus_Yanofskybacteria;f__Candidatus_Yanofskybacteria | 0.000149 | 0 | 0 | 0 | 0 | 0 | 0.000102 | 0.000104 | 9.75E-05 | 0.000221 | 0 | 7.27E-05 |
| d__Bacteria;p__Patescibacteria;c__Parcubacteria;o__Candidatus_Zambryskibacteria;f__Candidatus_Zambryskibacteria | 0 | 0 | 0 | 0 | 0 | 0 | 0 | 0 | 0 | 0 | 0 | 0 |
| d__Bacteria;p__Patescibacteria;c__Parcubacteria;o__Parcubacteria;f__Parcubacteria | 0.001532 | 0.000341 | 0.001827 | 0.000291 | 0.000481 | 0.000375 | 0.002885 | 0.002499 | 0.003201 | 0.004362 | 0.001682 | 0.00149 |
| d__Bacteria;p__Patescibacteria;c__Parcubacteria;o__unclassified_Parcubacteria;f__unclassified_Parcubacteria | 0 | 0 | 0 | 0 | 0 | 0 | 0 | 3.47E-05 | 0 | 0.000281 | 0 | 0.000182 |
| d__Bacteria;p__Patescibacteria;c__Saccharimonadia;o__Saccharimonadales;f__Saccharimonadaceae | 2.97E-05 | 5.39E-05 | 0 | 0 | 0 | 0 | 0 | 0.000243 | 0.000162 | 0 | 0.000194 | 0 |
| d__Bacteria;p__Patescibacteria;c__Saccharimonadia;o__Saccharimonadales;f__Saccharimonadales | 0.008418 | 0.003088 | 0.005565 | 0.007765 | 0.007449 | 0.005625 | 0.024205 | 0.03106 | 0.018426 | 0.018777 | 0.036857 | 0.02161 |
| d__Bacteria;p__Patescibacteria;c__Saccharimonadia;o__Saccharimonadales;f__unclassified_Saccharimonadales | 0 | 0 | 0 | 0 | 0 | 0 | 0.000945 | 0 | 0 | 0 | 0.001359 | 0.000327 |
| d__Bacteria;p__Patescibacteria;c__WS6_(Dojkabacteria);o__WS6_(Dojkabacteria);f__WS6_(Dojkabacteria) | 0 | 3.59E-05 | 0.000198 | 0 | 0 | 0 | 0.000255 | 0 | 0.000179 | 0.000161 | 6.47E-05 | 0.000345 |
| d__Bacteria;p__Patescibacteria;c__WWE3;o__WWE3;f__WWE3 | 0.000119 | 7.18E-05 | 0.000113 | 8.30E-05 | 0 | 0 | 0.000281 | 0.000416 | 0.00117 | 0.002473 | 0.000798 | 0.001127 |
| d__Bacteria;p__Planctomycetes;c__BD7-11;o__BD7-11;f__BD7-11 | 0.000446 | 0.000664 | 0.000255 | 0.000415 | 0.000441 | 0.0001 | 0.000434 | 8.68E-05 | 0.000341 | 0.000281 | 0.000302 | 0.000291 |
| d__Bacteria;p__Planctomycetes;c__OM190;o__OM190;f__OM190 | 0 | 0 | 0.000269 | 0 | 0 | 0 | 0 | 0 | 0 | 0 | 0 | 0.000109 |
| d__Bacteria;p__Planctomycetes;c__Phycisphaerae;o__Phycisphaerales;f__Phycisphaeraceae | 4.46E-05 | 5.39E-05 | 4.25E-05 | 6.23E-05 | 4.00E-05 | 0 | 0 | 0.000382 | 8.12E-05 | 0.000523 | 8.63E-05 | 0 |
| d__Bacteria;p__Planctomycetes;c__Phycisphaerae;o__Tepidisphaerales;f__WD2101_soil_group | 0 | 0 | 0 | 0.000104 | 0 | 0 | 7.66E-05 | 0.000174 | 0 | 0 | 0.000474 | 0 |
| d__Bacteria;p__Planctomycetes;c__Pla4_lineage;o__Pla4_lineage;f__Pla4_lineage | 0 | 0 | 0 | 0 | 0 | 0 | 0 | 0 | 0.000179 | 0 | 0 | 0 |
| d__Bacteria;p__Planctomycetes;c__Planctomycetacia;o__Gemmatales;f__Gemmataceae | 0.006187 | 0.006518 | 0.009432 | 0.002886 | 0.005527 | 0.002725 | 0.000409 | 5.21E-05 | 0.000731 | 0.000543 | 0.000539 | 0.001072 |
| d__Bacteria;p__Planctomycetes;c__Planctomycetacia;o__Isosphaerales;f__Isosphaeraceae | 0.002112 | 0.002927 | 0.004093 | 0.000997 | 0.004505 | 0.00435 | 0.000894 | 0.000416 | 0.000877 | 0.000663 | 0.000496 | 0.000945 |
| d__Bacteria;p__Planctomycetes;c__Planctomycetacia;o__Pirellulales;f__Pirellulaceae | 0 | 0 | 0 | 0 | 0 | 0 | 0 | 0 | 0 | 0 | 0 | 0 |
| d__Bacteria;p__Planctomycetes;c__Planctomycetacia;o__Planctomycetales;f__uncultured | 0 | 0 | 0 | 0 | 0 | 0 | 0 | 0 | 0 | 0 | 0 | 0 |
| d__Bacteria;p__Planctomycetes;c__unclassified_Planctomycetes;o__unclassified_Planctomycetes;f__unclassified_Planctomycetes | 0 | 0 | 0 | 0 | 0 | 0 | 0 | 0 | 0 | 0 | 0 | 0 |
| d__Bacteria;p__Planctomycetes;c__vadinHA49;o__vadinHA49;f__vadinHA49 | 0 | 0 | 0.00017 | 0.000187 | 6.01E-05 | 0 | 0.000204 | 0.000121 | 0 | 8.04E-05 | 8.63E-05 | 9.09E-05 |
| d__Bacteria;p__Proteobacteria;c__Alphaproteobacteria;o__Acetobacterales;f__Acetobacteraceae | 0.012746 | 0.006536 | 0.009672 | 0.02234 | 0.039949 | 0.1004 | 0.008528 | 0.005934 | 0.006386 | 0.006132 | 0.005543 | 0.003053 |
| d__Bacteria;p__Proteobacteria;c__Alphaproteobacteria;o__Acetobacterales;f__Acetobacterales_Incertae_Sedis | 0 | 0 | 0 | 0 | 0 | 0.000175 | 0 | 0 | 0 | 0 | 0 | 0 |
| d__Bacteria;p__Proteobacteria;c__Alphaproteobacteria;o__Azospirillales;f__Azospirillaceae | 0 | 0 | 0 | 0 | 0 | 0 | 0 | 0 | 0.000162 | 0.000181 | 0 | 0.000182 |
| d__Bacteria;p__Proteobacteria;c__Alphaproteobacteria;o__Azospirillales;f__Inquilinaceae | 0.000164 | 0.000162 | 0.000297 | 0.000789 | 0.001262 | 0.00155 | 0.000511 | 0.000278 | 0.000325 | 0.000503 | 0.000841 | 0.000164 |
| d__Bacteria;p__Proteobacteria;c__Alphaproteobacteria;o__Azospirillales;f__unclassified_Azospirillales | 0 | 0 | 0 | 0 | 0 | 0 | 0 | 0 | 0 | 0.000101 | 0 | 0 |
| d__Bacteria;p__Proteobacteria;c__Alphaproteobacteria;o__Azospirillales;f__uncultured | 0 | 0 | 0 | 0 | 0 | 0 | 0 | 0 | 0 | 0 | 0.000151 | 0.000436 |
| d__Bacteria;p__Proteobacteria;c__Alphaproteobacteria;o__Caedibacterales;f__Caedibacteraceae | 0 | 0 | 0 | 0.000104 | 0 | 0 | 0 | 0 | 0 | 0 | 0 | 0 |
| d__Bacteria;p__Proteobacteria;c__Alphaproteobacteria;o__Caulobacterales;f__Caulobacteraceae | 0.011288 | 0.007182 | 0.004942 | 0.010402 | 0.009672 | 0.01465 | 0.008834 | 0.008659 | 0.009506 | 0.010192 | 0.010503 | 0.006906 |
| d__Bacteria;p__Proteobacteria;c__Alphaproteobacteria;o__Caulobacterales;f__Hyphomonadaceae | 0.003912 | 0.002927 | 0.000439 | 0.000415 | 0.00038 | 0.00025 | 0.000664 | 0.000902 | 0.002145 | 0.001327 | 0.00317 | 0.006107 |
| d__Bacteria;p__Proteobacteria;c__Alphaproteobacteria;o__Dongiales;f__Dongiaceae | 0 | 0 | 0 | 4.15E-05 | 0 | 0.000625 | 0.001992 | 0.002325 | 0.003071 | 0.004986 | 0.002178 | 0.00229 |
| d__Bacteria;p__Proteobacteria;c__Alphaproteobacteria;o__Elsterales;f__Elsteraceae | 0.000193 | 0 | 0.000397 | 0.002138 | 0.001322 | 0.001375 | 0.002272 | 0.003349 | 0.003949 | 0.004161 | 0.003731 | 0.001945 |
| d__Bacteria;p__Proteobacteria;c__Alphaproteobacteria;o__Elsterales;f__URHD0088 | 0 | 0.000305 | 0.00051 | 0.000208 | 0.000421 | 0.001025 | 0.00646 | 0.006472 | 0.006581 | 0.006956 | 0.004702 | 0.003799 |
| d__Bacteria;p__Proteobacteria;c__Alphaproteobacteria;o__Elsterales;f__unclassified_Elsterales | 0 | 3.59E-05 | 0.000127 | 4.15E-05 | 0 | 0 | 7.66E-05 | 0 | 0 | 0 | 4.31E-05 | 0 |
| d__Bacteria;p__Proteobacteria;c__Alphaproteobacteria;o__Elsterales;f__uncultured | 0.033806 | 0.063167 | 0.051435 | 0.02888 | 0.025111 | 0.02015 | 0.013686 | 0.012216 | 0.013275 | 0.020787 | 0.017404 | 0.018084 |
| d__Bacteria;p__Proteobacteria;c__Alphaproteobacteria;o__Holosporales;f__Holosporaceae | 0.000208 | 0.000467 | 0.000212 | 0.000415 | 0 | 0 | 0.000766 | 0.000312 | 0.000877 | 0.00201 | 0.000733 | 0.001454 |
| d__Bacteria;p__Proteobacteria;c__Alphaproteobacteria;o__Micavibrionales;f__Micavibrionaceae | 0 | 0 | 0 | 0 | 0 | 0.00015 | 0.000562 | 0.00033 | 0.00013 | 0.000342 | 0.000216 | 0.000418 |
| d__Bacteria;p__Proteobacteria;c__Alphaproteobacteria;o__Micavibrionales;f__unclassified_Micavibrionales | 0 | 0 | 0 | 0 | 0 | 0 | 0 | 0 | 0 | 0 | 0 | 0 |
| d__Bacteria;p__Proteobacteria;c__Alphaproteobacteria;o__Micavibrionales;f__uncultured | 0 | 0 | 0 | 0 | 6.01E-05 | 0 | 0 | 0 | 0 | 0 | 0 | 0 |
| d__Bacteria;p__Proteobacteria;c__Alphaproteobacteria;o__Micropepsales;f__Micropepsaceae | 0.021476 | 0.01352 | 0.009474 | 0.020139 | 0.01654 | 0.02225 | 0.035236 | 0.030348 | 0.038559 | 0.038056 | 0.035197 | 0.02559 |
| d__Bacteria;p__Proteobacteria;c__Alphaproteobacteria;o__Paracaedibacterales;f__Paracaedibacteraceae | 0.000342 | 0 | 0.000142 | 0.000291 | 0 | 5.00E-05 | 0 | 3.47E-05 | 0.000114 | 0.000382 | 0.000453 | 3.63E-05 |
| d__Bacteria;p__Proteobacteria;c__Alphaproteobacteria;o__Parvibaculales;f__Parvibaculaceae | 0 | 0 | 0 | 0 | 0 | 0 | 0 | 0 | 0.000114 | 4.02E-05 | 8.63E-05 | 0 |
| d__Bacteria;p__Proteobacteria;c__Alphaproteobacteria;o__Puniceispirillales;f__Puniceispirillales | 0 | 0 | 0 | 0 | 0 | 0 | 0 | 0 | 0 | 0 | 0 | 0 |
| d__Bacteria;p__Proteobacteria;c__Alphaproteobacteria;o__Reyranellales;f__Reyranellaceae | 0.004402 | 0.004327 | 0.007761 | 0.002948 | 0.020245 | 0.008425 | 0.008656 | 0.008225 | 0.007101 | 0.009006 | 0.009403 | 0.005562 |
| d__Bacteria;p__Proteobacteria;c__Alphaproteobacteria;o__Rhizobiales;f__A0839 | 0 | 0 | 0.000241 | 0 | 0 | 0 | 0 | 0 | 9.75E-05 | 0.000201 | 0.000712 | 0.001218 |
| d__Bacteria;p__Proteobacteria;c__Alphaproteobacteria;o__Rhizobiales;f__Amb-16S-1323 | 0 | 0 | 0 | 0 | 0 | 0 | 7.66E-05 | 0.00033 | 0 | 0.000181 | 0.00097 | 0.000709 |
| d__Bacteria;p__Proteobacteria;c__Alphaproteobacteria;o__Rhizobiales;f__Beijerinckiaceae | 0.031144 | 0.044601 | 0.023352 | 0.048998 | 0.067322 | 0.04815 | 0.005822 | 0.003037 | 0.003071 | 0.001769 | 0.003429 | 0.001399 |
| d__Bacteria;p__Proteobacteria;c__Alphaproteobacteria;o__Rhizobiales;f__D05-2 | 0 | 0 | 0 | 0 | 0 | 0 | 0.000613 | 0.000486 | 0.000357 | 0.000442 | 0.000259 | 0.000509 |
| d__Bacteria;p__Proteobacteria;c__Alphaproteobacteria;o__Rhizobiales;f__Devosiaceae | 0.000268 | 0.000287 | 0.000184 | 0.000311 | 0.000661 | 0.00145 | 0.00932 | 0.005171 | 0.005086 | 0.004423 | 0.010438 | 0.004671 |
| d__Bacteria;p__Proteobacteria;c__Alphaproteobacteria;o__Rhizobiales;f__Hyphomicrobiaceae | 0.001056 | 0.00088 | 0.001147 | 0.001453 | 0.001462 | 0.00055 | 0.001558 | 0.000642 | 0.000325 | 0.001769 | 0.005845 | 0.004653 |
| d__Bacteria;p__Proteobacteria;c__Alphaproteobacteria;o__Rhizobiales;f__KF-JG30-B3 | 0.000788 | 0.002101 | 0.001784 | 0.00054 | 0.001382 | 0.00055 | 0.004187 | 0.002742 | 0.004712 | 0.004724 | 0.001467 | 0.003035 |
| d__Bacteria;p__Proteobacteria;c__Alphaproteobacteria;o__Rhizobiales;f__Kaistiaceae | 0 | 0 | 0 | 0 | 0 | 0 | 0 | 0.000139 | 0 | 0 | 0.000539 | 5.45E-05 |
| d__Bacteria;p__Proteobacteria;c__Alphaproteobacteria;o__Rhizobiales;f__Labraceae | 0 | 7.18E-05 | 0 | 2.08E-05 | 0.000501 | 0.000325 | 0.000357 | 3.47E-05 | 0.00026 | 0.000261 | 0.000453 | 0.000127 |
| d__Bacteria;p__Proteobacteria;c__Alphaproteobacteria;o__Rhizobiales;f__Methyloligellaceae | 0.000714 | 0.000772 | 0.003314 | 0 | 0.000881 | 0.0004 | 0.000511 | 0.000816 | 9.75E-05 | 0.000442 | 0.002006 | 0.003471 |
| d__Bacteria;p__Proteobacteria;c__Alphaproteobacteria;o__Rhizobiales;f__Methylopilaceae | 0 | 0 | 0 | 0 | 0 | 0 | 0 | 0 | 0 | 0 | 0 | 0 |
| d__Bacteria;p__Proteobacteria;c__Alphaproteobacteria;o__Rhizobiales;f__Pleomorphomonadaceae | 0 | 0 | 0 | 0 | 0 | 0 | 0 | 0 | 0 | 0 | 0 | 0 |
| d__Bacteria;p__Proteobacteria;c__Alphaproteobacteria;o__Rhizobiales;f__Rhizobiaceae | 0.001695 | 0.001221 | 0.000736 | 0 | 0.000881 | 0.000525 | 0.002298 | 0.001596 | 0.002584 | 0.00193 | 0.006017 | 0.004544 |
| d__Bacteria;p__Proteobacteria;c__Alphaproteobacteria;o__Rhizobiales;f__Rhizobiales_Incertae_Sedis | 0.00061 | 0.000808 | 0.000949 | 0.000374 | 0.00032 | 0.0001 | 0.011439 | 0.006923 | 0.004598 | 0.005629 | 0.008584 | 0.005525 |
| d__Bacteria;p__Proteobacteria;c__Alphaproteobacteria;o__Rhizobiales;f__Rhodomicrobiaceae | 0.000491 | 0.000305 | 0.000354 | 0.000208 | 0.000621 | 0.000475 | 0 | 0.000382 | 0.000146 | 0.000121 | 0.000259 | 0.000109 |
| d__Bacteria;p__Proteobacteria;c__Alphaproteobacteria;o__Rhizobiales;f__Xanthobacteraceae | 0.149308 | 0.169534 | 0.108463 | 0.144919 | 0.190292 | 0.21665 | 0.08837 | 0.073155 | 0.073105 | 0.1012 | 0.11247 | 0.098999 |
| d__Bacteria;p__Proteobacteria;c__Alphaproteobacteria;o__Rhizobiales;f__unclassified_Rhizobiales | 4.46E-05 | 0.000108 | 5.66E-05 | 0.000166 | 0.00016 | 0.000275 | 0.000817 | 6.94E-05 | 0.000179 | 4.02E-05 | 0.000474 | 0.000127 |
| d__Bacteria;p__Proteobacteria;c__Alphaproteobacteria;o__Rhizobiales;f__uncultured | 0.000714 | 0.00079 | 0.000283 | 0.000976 | 0.002163 | 0.001125 | 0.003115 | 0.003071 | 0.003412 | 0.007358 | 0.002825 | 0.002708 |
| d__Bacteria;p__Proteobacteria;c__Alphaproteobacteria;o__Rhodobacterales;f__Rhodobacteraceae | 0.000104 | 0 | 0 | 0 | 0 | 0 | 0 | 0 | 0 | 0 | 0 | 0 |
| d__Bacteria;p__Proteobacteria;c__Alphaproteobacteria;o__Rhodospirillales;f__Magnetospiraceae | 0 | 0 | 0 | 0 | 0 | 0 | 0 | 0 | 0 | 0 | 6.47E-05 | 0 |
| d__Bacteria;p__Proteobacteria;c__Alphaproteobacteria;o__Rhodospirillales;f__Magnetospirillaceae | 0.000134 | 0 | 0 | 0.000394 | 0 | 0.000425 | 0.000638 | 0.000868 | 0.000845 | 0.000523 | 0 | 0 |
| d__Bacteria;p__Proteobacteria;c__Alphaproteobacteria;o__Rhodospirillales;f__Rhodopirillaceae | 0 | 0 | 0 | 0 | 0 | 0 | 0 | 0.000156 | 0.000276 | 0.000101 | 0 | 7.27E-05 |
| d__Bacteria;p__Proteobacteria;c__Alphaproteobacteria;o__Rhodospirillales;f__Rhodospirillaceae | 0.000535 | 0.000233 | 0.000368 | 0.000851 | 0.000821 | 0.00045 | 0.001685 | 0.001163 | 0.002909 | 0.002151 | 0.001402 | 0.000963 |
| d__Bacteria;p__Proteobacteria;c__Alphaproteobacteria;o__Rhodospirillales;f__uncultured | 0.00177 | 0.000664 | 0.00051 | 0.004069 | 0.001722 | 0.00315 | 0.003549 | 0.003453 | 0.003754 | 0.004362 | 0.002092 | 0.001236 |
| d__Bacteria;p__Proteobacteria;c__Alphaproteobacteria;o__Rickettsiales;f__AB1 | 0 | 0 | 0 | 0 | 8.01E-05 | 0 | 0 | 0 | 4.87E-05 | 0.000342 | 0.000151 | 0 |
| d__Bacteria;p__Proteobacteria;c__Alphaproteobacteria;o__Rickettsiales;f__Midichloriaceae | 8.92E-05 | 0 | 0 | 0 | 0 | 0 | 0 | 0 | 0 | 0 | 0 | 0 |
| d__Bacteria;p__Proteobacteria;c__Alphaproteobacteria;o__Rickettsiales;f__Rickettsiaceae | 5.95E-05 | 0 | 4.25E-05 | 0 | 0 | 0 | 0 | 0 | 0.00026 | 8.04E-05 | 0 | 0.000127 |
| d__Bacteria;p__Proteobacteria;c__Alphaproteobacteria;o__Rickettsiales;f__SM2D12 | 0.001339 | 0.000467 | 0.000595 | 0.001619 | 0.001482 | 0 | 0.002221 | 0.002481 | 0.00416 | 0.00384 | 0.003774 | 0.002345 |
| d__Bacteria;p__Proteobacteria;c__Alphaproteobacteria;o__Rickettsiales;f__bac2nit3 | 0 | 0 | 0 | 0 | 0 | 0 | 0 | 0 | 9.75E-05 | 0 | 0 | 0 |
| d__Bacteria;p__Proteobacteria;c__Alphaproteobacteria;o__Rickettsiales;f__unclassified_Rickettsiales | 0 | 0 | 0 | 0 | 0 | 0 | 0 | 0 | 0 | 0 | 0 | 0 |
| d__Bacteria;p__Proteobacteria;c__Alphaproteobacteria;o__Sphingomonadales;f__Sphingomonadaceae | 0.000193 | 0 | 0.000368 | 0.000145 | 0.000661 | 0.00175 | 0.006307 | 0.009023 | 0.014559 | 0.013067 | 0.01184 | 0.015976 |
| d__Bacteria;p__Proteobacteria;c__Alphaproteobacteria;o__Thalassobaculales;f__uncultured | 0 | 0 | 0 | 0 | 0 | 0 | 0 | 0 | 0 | 0 | 0 | 0 |
| d__Bacteria;p__Proteobacteria;c__Alphaproteobacteria;o__Tistrellales;f__Geminicoccaceae | 0 | 0 | 0 | 0 | 0 | 0 | 0 | 0 | 4.87E-05 | 0 | 0 | 0 |
| d__Bacteria;p__Proteobacteria;c__Alphaproteobacteria;o__unclassified_Alphaproteobacteria;f__unclassified_Alphaproteobacteria | 0.004938 | 0.002873 | 0.002761 | 0.002014 | 0.006127 | 0.005225 | 0.001123 | 0.001631 | 0.001674 | 0.001186 | 0.00207 | 0.002036 |
| d__Bacteria;p__Proteobacteria;c__Alphaproteobacteria;o__uncultured;f__uncultured | 0.001011 | 0.000323 | 0.002351 | 0.00054 | 0.001802 | 0.0012 | 0.003319 | 0.004893 | 0.004712 | 0.007157 | 0.002847 | 0.003853 |
| d__Bacteria;p__Proteobacteria;c__Deltaproteobacteria;o__Bdellovibrionales;f__Bacteriovoracaceae | 0 | 0.000108 | 0 | 0 | 0 | 0.0001 | 0 | 0 | 0 | 0 | 0 | 0 |
| d__Bacteria;p__Proteobacteria;c__Deltaproteobacteria;o__Bdellovibrionales;f__Bdellovibrionaceae | 0.001041 | 0.00079 | 0.00085 | 0.002325 | 0.001422 | 0.000975 | 0.001277 | 0.0017 | 0.0013 | 0.000623 | 0.001208 | 0.000527 |
| d__Bacteria;p__Proteobacteria;c__Deltaproteobacteria;o__Desulfarculales;f__Desulfarculaceae | 0.000149 | 0.000323 | 0.001926 | 0 | 0.002583 | 0.000725 | 0 | 0 | 0 | 0 | 0.000108 | 0.000145 |
| d__Bacteria;p__Proteobacteria;c__Deltaproteobacteria;o__Desulfobacterales;f__Desulfobacteraceae | 0 | 0 | 0 | 0 | 0 | 0 | 0 | 0 | 0 | 0 | 0 | 0 |
| d__Bacteria;p__Proteobacteria;c__Deltaproteobacteria;o__Desulfovibrionales;f__Desulfovibrionaceae | 0 | 0 | 0 | 0 | 0 | 0 | 0 | 0 | 0 | 0 | 0 | 0 |
| d__Bacteria;p__Proteobacteria;c__Deltaproteobacteria;o__Desulfuromonadales;f__Geobacteraceae | 0 | 0 | 0 | 0 | 0.0001 | 0 | 0 | 0 | 0 | 0 | 0 | 0 |
| d__Bacteria;p__Proteobacteria;c__Deltaproteobacteria;o__MBNT15;f__MBNT15 | 0.000759 | 0.000593 | 0.002747 | 0.000125 | 0.000641 | 0 | 0 | 0 | 0 | 0 | 0 | 0.000127 |
| d__Bacteria;p__Proteobacteria;c__Deltaproteobacteria;o__Myxococcales;f__27F-1492R | 0.000193 | 0 | 0 | 0.00054 | 0 | 0 | 0.000766 | 0.000798 | 0 | 0.000382 | 0 | 0 |
| d__Bacteria;p__Proteobacteria;c__Deltaproteobacteria;o__Myxococcales;f__Amb-16S-1034 | 0 | 0 | 0.000269 | 0 | 0.000441 | 0 | 0 | 0 | 0 | 0 | 0 | 0 |
| d__Bacteria;p__Proteobacteria;c__Deltaproteobacteria;o__Myxococcales;f__Archangiaceae | 0.0018 | 0.000305 | 0.00119 | 0.000997 | 0.00022 | 0.0005 | 0.001302 | 0.001891 | 0.000682 | 0.000482 | 0.000712 | 0.000363 |
| d__Bacteria;p__Proteobacteria;c__Deltaproteobacteria;o__Myxococcales;f__BIrii41 | 0 | 0 | 0.000708 | 0 | 0.001782 | 0.000725 | 0.003319 | 0.000538 | 0.00065 | 0.001548 | 0.001165 | 0.000218 |
| d__Bacteria;p__Proteobacteria;c__Deltaproteobacteria;o__Myxococcales;f__Blfdi19 | 0.00058 | 0.000413 | 0.000581 | 0.000228 | 0.000661 | 0.00035 | 0.000281 | 0.00026 | 0.001917 | 0.000684 | 0.000388 | 0.000491 |
| d__Bacteria;p__Proteobacteria;c__Deltaproteobacteria;o__Myxococcales;f__FFCH16767 | 0 | 0 | 0 | 0 | 0 | 0 | 0 | 0 | 0 | 0 | 0 | 0 |
| d__Bacteria;p__Proteobacteria;c__Deltaproteobacteria;o__Myxococcales;f__Haliangiaceae | 0.006737 | 0.003088 | 0.010678 | 0.005647 | 0.012195 | 0.0058 | 0.009473 | 0.012771 | 0.012658 | 0.01361 | 0.009878 | 0.010687 |
| d__Bacteria;p__Proteobacteria;c__Deltaproteobacteria;o__Myxococcales;f__KD3-10 | 5.95E-05 | 0.000108 | 0 | 0.000498 | 0.00036 | 0 | 0.000306 | 6.94E-05 | 4.87E-05 | 0.000101 | 0 | 3.63E-05 |
| d__Bacteria;p__Proteobacteria;c__Deltaproteobacteria;o__Myxococcales;f__MSB-4B10 | 0 | 0 | 0 | 0 | 0 | 0 | 0 | 3.47E-05 | 0 | 0 | 0.000129 | 0 |
| d__Bacteria;p__Proteobacteria;c__Deltaproteobacteria;o__Myxococcales;f__Nannocystaceae | 0 | 0 | 0 | 0 | 0 | 0 | 0 | 0 | 0 | 0 | 0 | 0 |
| d__Bacteria;p__Proteobacteria;c__Deltaproteobacteria;o__Myxococcales;f__P3OB-42 | 0.00055 | 0.000305 | 0.000708 | 0.000519 | 0.00012 | 0.000175 | 0.00046 | 0.000434 | 0.000325 | 0.000643 | 0.000237 | 0.000491 |
| d__Bacteria;p__Proteobacteria;c__Deltaproteobacteria;o__Myxococcales;f__Phaselicystidaceae | 0 | 0.000198 | 0.000142 | 0.000291 | 0.00024 | 0.000225 | 0.000485 | 0.000677 | 0.000292 | 0.000985 | 0.000237 | 0.000418 |
| d__Bacteria;p__Proteobacteria;c__Deltaproteobacteria;o__Myxococcales;f__Polyangiaceae | 0.012478 | 0.007918 | 0.006458 | 0.022049 | 0.011454 | 0.006775 | 0.004443 | 0.004581 | 0.006435 | 0.005187 | 0.004356 | 0.003362 |
| d__Bacteria;p__Proteobacteria;c__Deltaproteobacteria;o__Myxococcales;f__Sandaracinaceae | 0 | 0 | 0.000326 | 0 | 0.00022 | 0.000475 | 0.000434 | 0.000486 | 0.000309 | 0.000462 | 0.000216 | 0.000218 |
| d__Bacteria;p__Proteobacteria;c__Deltaproteobacteria;o__Myxococcales;f__Vulgatibacteraceae | 0 | 0 | 0 | 0 | 0 | 0 | 0 | 0 | 0 | 0 | 0 | 0 |
| d__Bacteria;p__Proteobacteria;c__Deltaproteobacteria;o__Myxococcales;f__bacteriap25 | 0 | 0 | 2.83E-05 | 0 | 0 | 0 | 0 | 0 | 0 | 8.04E-05 | 0 | 0.000618 |
| d__Bacteria;p__Proteobacteria;c__Deltaproteobacteria;o__Myxococcales;f__mle1-27 | 8.92E-05 | 1.80E-05 | 0.000198 | 0.000166 | 0.00038 | 0 | 0.000102 | 0.000364 | 0.000991 | 0.000824 | 0.000151 | 0.000491 |
| d__Bacteria;p__Proteobacteria;c__Deltaproteobacteria;o__Myxococcales;f__unclassified_Myxococcales | 0.000416 | 0.00018 | 0.000212 | 0.001017 | 0.00032 | 0 | 0.00023 | 0.000382 | 9.75E-05 | 0.000261 | 0.000216 | 0.000218 |
| d__Bacteria;p__Proteobacteria;c__Deltaproteobacteria;o__Myxococcales;f__uncultured | 0.004655 | 0.001041 | 0.00388 | 0.00218 | 0.002042 | 0.00085 | 0.000817 | 0.000399 | 0.000341 | 0.000784 | 0.000431 | 0.000236 |
| d__Bacteria;p__Proteobacteria;c__Deltaproteobacteria;o__NB1-j;f__NB1-j | 0 | 0 | 0.000751 | 0 | 0 | 0 | 0 | 0 | 0 | 0 | 0 | 0 |
| d__Bacteria;p__Proteobacteria;c__Deltaproteobacteria;o__Oligoflexales;f__0319-6G20 | 0.003213 | 0.001401 | 0.002337 | 0.004152 | 0.001922 | 0.00155 | 0.002094 | 0.001943 | 0.002112 | 0.00203 | 0.001143 | 0.001345 |
| d__Bacteria;p__Proteobacteria;c__Deltaproteobacteria;o__Oligoflexales;f__053A03-B-DI-P58 | 0 | 0 | 0 | 0 | 0 | 0 | 0 | 0 | 0 | 4.02E-05 | 0 | 7.27E-05 |
| d__Bacteria;p__Proteobacteria;c__Deltaproteobacteria;o__Oligoflexales;f__Oligoflexaceae | 0.000164 | 0.000144 | 0.00017 | 0.000457 | 0.0004 | 0.00085 | 0.000255 | 0.000104 | 0.000439 | 0.000663 | 0 | 0.0002 |
| d__Bacteria;p__Proteobacteria;c__Deltaproteobacteria;o__PB19;f__PB19 | 0 | 0 | 0 | 0 | 0 | 0 | 0 | 0 | 0 | 0 | 0 | 0 |
| d__Bacteria;p__Proteobacteria;c__Deltaproteobacteria;o__RCP2-54;f__RCP2-54 | 0.002305 | 0.006697 | 0.001968 | 0.002512 | 0.004786 | 0.00165 | 0.000664 | 0.000156 | 0.000325 | 0.000422 | 0 | 0.000818 |
| d__Bacteria;p__Proteobacteria;c__Deltaproteobacteria;o__SAR324_clade(Marine_group_B);f__SAR324_clade(Marine_group_B) | 7.44E-05 | 0 | 0.000184 | 0 | 0.0001 | 0 | 0 | 0 | 0 | 0 | 4.31E-05 | 0.000164 |
| d__Bacteria;p__Proteobacteria;c__Deltaproteobacteria;o__Syntrophobacterales;f__Syntrophaceae | 0 | 0.000108 | 0 | 0 | 0 | 0 | 0 | 0 | 0 | 0 | 0 | 0 |
| d__Bacteria;p__Proteobacteria;c__Deltaproteobacteria;o__Syntrophobacterales;f__Syntrophobacteraceae | 0 | 0 | 0 | 0 | 0 | 0 | 0 | 0 | 0 | 0 | 0 | 5.45E-05 |
| d__Bacteria;p__Proteobacteria;c__Deltaproteobacteria;o__unclassified_Deltaproteobacteria;f__unclassified_Deltaproteobacteria | 5.95E-05 | 1.80E-05 | 0.000864 | 4.15E-05 | 0.00024 | 5.00E-05 | 5.11E-05 | 3.47E-05 | 0 | 0 | 0 | 0.000164 |
| d__Bacteria;p__Proteobacteria;c__Gammaproteobacteria;o__Alteromonadales;f__Colwelliaceae | 0 | 0 | 0 | 0 | 0 | 0 | 0 | 0 | 0 | 0 | 0 | 0 |
| d__Bacteria;p__Proteobacteria;c__Gammaproteobacteria;o__B2M28;f__B2M28 | 0 | 0 | 0 | 0 | 0 | 0 | 0 | 0 | 0 | 0 | 0 | 0 |
| d__Bacteria;p__Proteobacteria;c__Gammaproteobacteria;o__BD72BR169;f__BD72BR169 | 5.95E-05 | 0 | 0 | 0 | 0 | 0 | 0 | 0 | 0 | 0 | 0 | 0 |
| d__Bacteria;p__Proteobacteria;c__Gammaproteobacteria;o__Betaproteobacteriales;f__A21b | 0.003465 | 0.004309 | 0.005041 | 0.003343 | 0.004746 | 0.001175 | 0.001455 | 0.001718 | 0.001316 | 0.00201 | 0.003019 | 0.005525 |
| d__Bacteria;p__Proteobacteria;c__Gammaproteobacteria;o__Betaproteobacteriales;f__B1-7BS | 0 | 0 | 0 | 0 | 0 | 0 | 0 | 0 | 6.50E-05 | 0.000503 | 0.000345 | 0.000309 |
| d__Bacteria;p__Proteobacteria;c__Gammaproteobacteria;o__Betaproteobacteriales;f__Burkholderiaceae | 0.002261 | 0.003501 | 0.003413 | 0.006021 | 0.015158 | 0.051925 | 0.007558 | 0.01107 | 0.010903 | 0.009006 | 0.016477 | 0.009869 |
| d__Bacteria;p__Proteobacteria;c__Gammaproteobacteria;o__Betaproteobacteriales;f__Chitinibacteraceae | 0 | 0 | 0 | 0 | 0 | 0 | 0 | 0 | 0 | 0 | 0 | 0 |
| d__Bacteria;p__Proteobacteria;c__Gammaproteobacteria;o__Betaproteobacteriales;f__Chromobacteriaceae | 0 | 0 | 0 | 0 | 0 | 0 | 0 | 0 | 0.000114 | 0 | 0 | 0 |
| d__Bacteria;p__Proteobacteria;c__Gammaproteobacteria;o__Betaproteobacteriales;f__Gallionellaceae | 0 | 0 | 0 | 0 | 0 | 0 | 0 | 3.47E-05 | 0 | 0 | 0 | 0 |
| d__Bacteria;p__Proteobacteria;c__Gammaproteobacteria;o__Betaproteobacteriales;f__Hydrogenophilaceae | 0 | 0 | 0 | 0 | 0 | 0 | 0 | 0 | 0 | 0 | 0 | 0 |
| d__Bacteria;p__Proteobacteria;c__Gammaproteobacteria;o__Betaproteobacteriales;f__Methylophilaceae | 0 | 0 | 0 | 0 | 0 | 0 | 0.000434 | 0.000278 | 0.000211 | 0.000422 | 0.00069 | 0.000527 |
| d__Bacteria;p__Proteobacteria;c__Gammaproteobacteria;o__Betaproteobacteriales;f__Neisseriaceae | 5.95E-05 | 0 | 0 | 6.23E-05 | 0.00018 | 0 | 0.00023 | 8.68E-05 | 0.000487 | 0.000181 | 0 | 0 |
| d__Bacteria;p__Proteobacteria;c__Gammaproteobacteria;o__Betaproteobacteriales;f__Nitrosomonadaceae | 0.005652 | 0.002604 | 0.009545 | 0.000145 | 0.003024 | 0.0013 | 0.034163 | 0.034235 | 0.040444 | 0.039523 | 0.033665 | 0.027371 |
| d__Bacteria;p__Proteobacteria;c__Gammaproteobacteria;o__Betaproteobacteriales;f__Rhodocyclaceae | 0.000134 | 0 | 0.000283 | 0 | 8.01E-05 | 0.0003 | 0.000102 | 0 | 0.000861 | 0.000141 | 0.000108 | 0.000509 |
| d__Bacteria;p__Proteobacteria;c__Gammaproteobacteria;o__Betaproteobacteriales;f__SC-I-84 | 0.000402 | 0.000359 | 0.003654 | 0 | 0.001842 | 0.0008 | 0.010622 | 0.013465 | 0.017062 | 0.013268 | 0.013199 | 0.014849 |
| d__Bacteria;p__Proteobacteria;c__Gammaproteobacteria;o__Betaproteobacteriales;f__Sulfuricellaceae | 0 | 0 | 0 | 0 | 0 | 0 | 0 | 0 | 0 | 0 | 0 | 0 |
| d__Bacteria;p__Proteobacteria;c__Gammaproteobacteria;o__Betaproteobacteriales;f__TRA3-20 | 0 | 0 | 0.00017 | 0 | 0.00032 | 0.000325 | 0.001455 | 0.003071 | 0.002129 | 0.004584 | 0.001833 | 0.002381 |
| d__Bacteria;p__Proteobacteria;c__Gammaproteobacteria;o__Betaproteobacteriales;f__unclassified_Betaproteobacteriales | 0.001725 | 0.000126 | 0.001034 | 0.001869 | 0.000481 | 0.0027 | 0.000128 | 6.94E-05 | 0.000162 | 0.000382 | 8.63E-05 | 0.000254 |
| d__Bacteria;p__Proteobacteria;c__Gammaproteobacteria;o__Betaproteobacteriales;f__uncultured | 0 | 0.000251 | 0.00034 | 0.002159 | 0.000581 | 0 | 0.000332 | 0.000868 | 0.000747 | 0 | 0 | 0 |
| d__Bacteria;p__Proteobacteria;c__Gammaproteobacteria;o__CCD24;f__CCD24 | 0 | 0 | 0.000198 | 0 | 0 | 0 | 0 | 0 | 0 | 0 | 0.000216 | 0.000145 |
| d__Bacteria;p__Proteobacteria;c__Gammaproteobacteria;o__Cellvibrionales;f__Cellvibrionaceae | 0 | 0 | 0 | 0 | 0 | 0.00015 | 0 | 0 | 0 | 0 | 0 | 0 |
| d__Bacteria;p__Proteobacteria;c__Gammaproteobacteria;o__Cellvibrionales;f__Halieaceae | 0 | 0 | 0 | 0 | 0 | 0 | 0 | 0 | 0 | 0 | 0 | 0 |
| d__Bacteria;p__Proteobacteria;c__Gammaproteobacteria;o__Competibacterales;f__Competibacteraceae | 0 | 0 | 0 | 0 | 0 | 0 | 0 | 0 | 0 | 0 | 0 | 0 |
| d__Bacteria;p__Proteobacteria;c__Gammaproteobacteria;o__Coxiellales;f__Coxiellaceae | 0.00113 | 0.000808 | 0.000524 | 0.001038 | 0.00012 | 0.000175 | 0.000587 | 0.000226 | 0.000292 | 0.000563 | 0.000194 | 0.000236 |
| d__Bacteria;p__Proteobacteria;c__Gammaproteobacteria;o__Diplorickettsiales;f__Diplorickettsiaceae | 0.008299 | 0.00492 | 0.002733 | 0.009322 | 0.003644 | 0.0029 | 0.008554 | 0.010133 | 0.016785 | 0.01753 | 0.012034 | 0.019138 |
| d__Bacteria;p__Proteobacteria;c__Gammaproteobacteria;o__EC3;f__EC3 | 0.000312 | 7.18E-05 | 0 | 0 | 0 | 0 | 0 | 0 | 0 | 6.03E-05 | 0 | 0 |
| d__Bacteria;p__Proteobacteria;c__Gammaproteobacteria;o__EV818SWSAP88;f__EV818SWSAP88 | 0 | 0 | 0 | 0 | 0 | 0 | 0 | 0 | 0 | 0 | 0 | 0 |
| d__Bacteria;p__Proteobacteria;c__Gammaproteobacteria;o__Enterobacteriales;f__Enterobacteriaceae | 0 | 0.000144 | 0.000283 | 8.30E-05 | 0.001061 | 0.002425 | 0 | 3.47E-05 | 0 | 0 | 0 | 0.000491 |
| d__Bacteria;p__Proteobacteria;c__Gammaproteobacteria;o__GB102;f__GB102 | 0 | 0 | 0 | 0 | 0 | 0 | 0 | 0 | 0 | 0 | 0 | 0 |
| d__Bacteria;p__Proteobacteria;c__Gammaproteobacteria;o__Gammaproteobacteria_Incertae_Sedis;f__Unknown_Family | 0.013817 | 0.013502 | 0.013312 | 0.016672 | 0.007109 | 0.004 | 0.012562 | 0.010411 | 0.008612 | 0.00973 | 0.006168 | 0.005725 |
| d__Bacteria;p__Proteobacteria;c__Gammaproteobacteria;o__JG36-TzT-191;f__JG36-TzT-191 | 0.004417 | 0.001796 | 0.001983 | 0.011772 | 0.001181 | 0.001125 | 0 | 0.000312 | 0.00013 | 6.03E-05 | 0.00041 | 0.0012 |
| d__Bacteria;p__Proteobacteria;c__Gammaproteobacteria;o__KF-JG30-C25;f__KF-JG30-C25 | 5.95E-05 | 0 | 9.91E-05 | 0.000478 | 0.000441 | 0.000775 | 0.001455 | 0.001562 | 0.00065 | 0.000482 | 0.000496 | 0.000218 |
| d__Bacteria;p__Proteobacteria;c__Gammaproteobacteria;o__KI89A_clade;f__KI89A_clade | 0 | 0 | 0.000198 | 0 | 0.00016 | 0.0001 | 0 | 0 | 0 | 0 | 0 | 0 |
| d__Bacteria;p__Proteobacteria;c__Gammaproteobacteria;o__Legionellales;f__Legionellaceae | 0.000788 | 0.000269 | 0.00051 | 0.000727 | 0.000501 | 0.000525 | 0.001609 | 0.001475 | 0.003006 | 0.003458 | 0.002394 | 0.002963 |
| d__Bacteria;p__Proteobacteria;c__Gammaproteobacteria;o__Oceanospirillales;f__Halomonadaceae | 0.036617 | 0.035605 | 0.001331 | 0.00353 | 0.003244 | 0.002075 | 0.006307 | 0.007166 | 0.025446 | 0.012464 | 0.006988 | 0.05338 |
| d__Bacteria;p__Proteobacteria;c__Gammaproteobacteria;o__Oceanospirillales;f__Pseudohongiellaceae | 0 | 0 | 0 | 0 | 0 | 0 | 0 | 0 | 0 | 0 | 0 | 0 |
| d__Bacteria;p__Proteobacteria;c__Gammaproteobacteria;o__Oceanospirillales;f__unclassified_Oceanospirillales | 0 | 0 | 0 | 0 | 0 | 0 | 0 | 0 | 0 | 0 | 0 | 0 |
| d__Bacteria;p__Proteobacteria;c__Gammaproteobacteria;o__PLTA13;f__PLTA13 | 0 | 0 | 0 | 0 | 0 | 0 | 0 | 0 | 0 | 0 | 0 | 0 |
| d__Bacteria;p__Proteobacteria;c__Gammaproteobacteria;o__Pseudomonadales;f__Moraxellaceae | 0.000193 | 0.000198 | 0.001841 | 0.000187 | 0.00012 | 0.00015 | 0 | 0.000104 | 0.000244 | 0 | 0 | 0.000691 |
| d__Bacteria;p__Proteobacteria;c__Gammaproteobacteria;o__Pseudomonadales;f__Pseudomonadaceae | 0.000372 | 0.001813 | 0 | 0.000727 | 0.002323 | 0.0021 | 0.000102 | 0.001475 | 0.000861 | 0.000201 | 0.001423 | 0.00578 |
| d__Bacteria;p__Proteobacteria;c__Gammaproteobacteria;o__Pseudomonadales;f__unclassified_Pseudomonadales | 0 | 0 | 0 | 0 | 0 | 0 | 0 | 0 | 0 | 0 | 0 | 3.63E-05 |
| d__Bacteria;p__Proteobacteria;c__Gammaproteobacteria;o__R7C24;f__R7C24 | 0 | 0 | 0 | 0 | 0 | 0 | 0 | 0 | 0 | 0 | 0 | 5.45E-05 |
| d__Bacteria;p__Proteobacteria;c__Gammaproteobacteria;o__Salinisphaerales;f__Solimonadaceae | 2.97E-05 | 0.000108 | 0 | 8.30E-05 | 6.01E-05 | 0.000275 | 0.000153 | 0.000607 | 0.001056 | 0.000643 | 0.000561 | 0.000945 |
| d__Bacteria;p__Proteobacteria;c__Gammaproteobacteria;o__Steroidobacterales;f__Steroidobacteraceae | 0.000268 | 5.39E-05 | 0.001062 | 0.000644 | 0.00034 | 0 | 0.000562 | 0.001336 | 0.000601 | 0.000342 | 0 | 0.000145 |
| d__Bacteria;p__Proteobacteria;c__Gammaproteobacteria;o__WD260;f__WD260 | 0.006604 | 0.007182 | 0.002705 | 0.012416 | 0.0168 | 0.014575 | 0.002758 | 0.00321 | 0.002649 | 0.00386 | 0.001596 | 0.001054 |
| d__Bacteria;p__Proteobacteria;c__Gammaproteobacteria;o__Xanthomonadales;f__Rhodanobacteraceae | 0.004402 | 0.000736 | 0.001529 | 0.005938 | 0.013937 | 0.010875 | 0.013022 | 0.019885 | 0.023415 | 0.018998 | 0.043047 | 0.017339 |
| d__Bacteria;p__Proteobacteria;c__Gammaproteobacteria;o__Xanthomonadales;f__Xanthomonadaceae | 0.00113 | 0.000359 | 0.000312 | 0.000311 | 0.000721 | 0 | 0.001915 | 0.002134 | 0.003965 | 0.004624 | 0.004852 | 0.004871 |
| d__Bacteria;p__Proteobacteria;c__Gammaproteobacteria;o__Xanthomonadales;f__unclassified_Xanthomonadales | 0 | 0 | 0 | 0 | 0 | 0 | 0.000102 | 1.74E-05 | 6.50E-05 | 8.04E-05 | 0.00028 | 0 |
| d__Bacteria;p__Proteobacteria;c__Gammaproteobacteria;o__Xanthomonadales;f__uncultured | 0 | 0 | 0 | 0 | 0 | 0 | 2.55E-05 | 0 | 0 | 6.03E-05 | 0 | 0 |
| d__Bacteria;p__Proteobacteria;c__Gammaproteobacteria;o__unclassified_Gammaproteobacteria;f__unclassified_Gammaproteobacteria | 0.000967 | 0.001005 | 0.001034 | 0.001661 | 0.001121 | 0.00035 | 0.000664 | 0.000538 | 0.001397 | 0.001568 | 0.000604 | 0.000545 |
| d__Bacteria;p__Proteobacteria;c__Gammaproteobacteria;o__uncultured;f__uncultured | 0.000134 | 8.98E-05 | 0.000142 | 0.000187 | 0 | 0 | 7.66E-05 | 0.000312 | 0.000829 | 0.000382 | 0 | 0 |
| d__Bacteria;p__Proteobacteria;c__unclassified_Proteobacteria;o__unclassified_Proteobacteria;f__unclassified_Proteobacteria | 4.46E-05 | 8.98E-05 | 0 | 0.000104 | 0.00012 | 0.0002 | 0.000306 | 0.000156 | 0.000146 | 0.000141 | 0.000129 | 7.27E-05 |
| d__Bacteria;p__Rokubacteria;c__NC10;o__Rokubacteriales;f__Rokubacteriales | 0.001443 | 0.001688 | 0.004631 | 0 | 0.000541 | 0.0002 | 0 | 0 | 0 | 0 | 0.000108 | 0.000291 |
| d__Bacteria;p__Spirochaetes;c__Leptospirae;o__Leptospirales;f__Leptospiraceae | 0 | 0 | 0 | 0 | 0 | 0 | 0.000102 | 6.94E-05 | 8.12E-05 | 0.000362 | 0.000151 | 3.63E-05 |
| d__Bacteria;p__Spirochaetes;c__Spirochaetia;o__Brevinematales;f__Brevinemataceae | 0 | 0 | 0 | 0 | 0 | 0 | 0 | 0 | 0 | 0 | 0 | 0 |
| d__Bacteria;p__Spirochaetes;c__Spirochaetia;o__Spirochaetales;f__Spirochaetaceae | 0 | 5.39E-05 | 0 | 0 | 0 | 0 | 0 | 0 | 0.000114 | 0 | 0 | 0.0002 |
| d__Bacteria;p__Tenericutes;c__Mollicutes;o__Mycoplasmatales;f__Mycoplasmataceae | 0 | 0 | 0 | 0 | 0 | 0 | 0 | 0 | 0 | 0 | 0 | 0 |
| d__Bacteria;p__Verrucomicrobia;c__Verrucomicrobiae;o__Chthoniobacterales;f__Chthoniobacteraceae | 0.009295 | 0.014023 | 0.017334 | 0.001599 | 0.008771 | 0.001825 | 0.001302 | 0.00026 | 0.000374 | 0.001005 | 0.002847 | 0.013231 |
| d__Bacteria;p__Verrucomicrobia;c__Verrucomicrobiae;o__Chthoniobacterales;f__Terrimicrobiaceae | 0 | 0 | 0 | 0 | 0 | 0 | 0 | 0 | 8.12E-05 | 0.000141 | 0 | 0 |
| d__Bacteria;p__Verrucomicrobia;c__Verrucomicrobiae;o__Chthoniobacterales;f__Xiphinematobacteraceae | 0.000506 | 0.000934 | 0.00136 | 0.000934 | 0.001362 | 0.002125 | 7.66E-05 | 0 | 0 | 0 | 0 | 0.000182 |
| d__Bacteria;p__Verrucomicrobia;c__Verrucomicrobiae;o__Chthoniobacterales;f__unclassified_Chthoniobacterales | 0 | 0 | 0 | 0 | 4.00E-05 | 0 | 0 | 0 | 0 | 0 | 0 | 0 |
| d__Bacteria;p__Verrucomicrobia;c__Verrucomicrobiae;o__Methylacidiphilales;f__Methylacidiphilaceae | 0.000164 | 0.000287 | 0.000184 | 0.000311 | 0.00034 | 0.00025 | 0 | 5.21E-05 | 0 | 0 | 8.63E-05 | 0.000127 |
| d__Bacteria;p__Verrucomicrobia;c__Verrucomicrobiae;o__Opitutales;f__Opitutaceae | 0.000327 | 0.000467 | 0.000156 | 0.000934 | 0.000421 | 0.000225 | 0.000817 | 0.000659 | 0.001202 | 0.000824 | 0.00041 | 0.000418 |
| d__Bacteria;p__Verrucomicrobia;c__Verrucomicrobiae;o__Opitutales;f__Puniceicoccaceae | 0 | 0 | 0 | 4.15E-05 | 0 | 0 | 7.66E-05 | 0 | 0 | 0 | 0 | 0 |
| d__Bacteria;p__Verrucomicrobia;c__Verrucomicrobiae;o__Pedosphaerales;f__Pedosphaeraceae | 0.003659 | 0.001778 | 0.002096 | 0.00353 | 0.002042 | 0.0007 | 0.002681 | 0.001614 | 0.003087 | 0.004181 | 0.001057 | 0.001581 |
| d__Bacteria;p__Verrucomicrobia;c__Verrucomicrobiae;o__S-BQ2-57_soil_group;f__S-BQ2-57_soil_group | 0.000967 | 0.00149 | 0.000963 | 0.000914 | 0.00024 | 0 | 0 | 0 | 9.75E-05 | 0 | 0 | 0 |
| d__Bacteria;p__Verrucomicrobia;c__Verrucomicrobiae;o__Verrucomicrobiales;f__Akkermansiaceae | 4.46E-05 | 7.18E-05 | 0 | 0 | 0 | 7.50E-05 | 0 | 6.94E-05 | 0 | 4.02E-05 | 0 | 0 |
| d__Bacteria;p__Verrucomicrobia;c__Verrucomicrobiae;o__Verrucomicrobiales;f__Rubritaleaceae | 0 | 0 | 0 | 0 | 0 | 0 | 0 | 0 | 0 | 0 | 0 | 7.27E-05 |
| d__Bacteria;p__Verrucomicrobia;c__Verrucomicrobiae;o__Verrucomicrobiales;f__Verrucomicrobiaceae | 0.000178 | 0 | 0 | 0 | 4.00E-05 | 0 | 0 | 0 | 8.12E-05 | 0.000261 | 8.63E-05 | 0 |
| d__Bacteria;p__Verrucomicrobia;c__Verrucomicrobiae;o__unclassified_Verrucomicrobiae;f__unclassified_Verrucomicrobiae | 0 | 3.59E-05 | 8.50E-05 | 0 | 0 | 0 | 5.11E-05 | 0 | 0 | 4.02E-05 | 0 | 0 |
| d__Bacteria;p__Verrucomicrobia;c__Verrucomicrobiae;o__uncultured;f__uncultured | 0 | 0 | 0 | 0 | 0 | 0 | 0 | 0 | 0 | 0 | 0 | 0 |
| d__Bacteria;p__WPS-2;c__WPS-2;o__WPS-2;f__WPS-2 | 0.068697 | 0.038119 | 0.098564 | 0.040195 | 0.029236 | 0.016775 | 0.006996 | 0.006299 | 0.004079 | 0.003357 | 0.003364 | 0.002399 |
| d__Bacteria;p__WS2;c__WS2;o__WS2;f__WS2 | 0 | 0.000144 | 0 | 0 | 0 | 0 | 0 | 0.000243 | 0.000325 | 0.000342 | 0.000345 | 0.000782 |
| d__Bacteria;p__unclassified_Bacteria;c__unclassified_Bacteria;o__unclassified_Bacteria;f__unclassified_Bacteria | 0.001145 | 0.000718 | 0.00092 | 0.001578 | 0.000581 | 0.00035 | 0.001941 | 0.001319 | 0.00195 | 0.010916 | 0.002027 | 0.00149 |

**Supplemental table 5b** Soil fungal_OTU_table at family

| Taxon | **WT_1** | **WT_2** | **WT_3** | **WT_4** | **WT_5** | **WT_6** | **CT_1** | **CT_2** | **CT_3** | **CT_4** | **CT_5** | **CT_6** |
| --- | --- | --- | --- | --- | --- | --- | --- | --- | --- | --- | --- | --- |
| d__Fungi;p__Ascomycota;c__Archaeorhizomycetes;o__Archaeorhizomycetales;f__Archaeorhizomycetaceae | 0.022921 | 0.090637 | 0.006408 | 0.796463 | 0.088946 | 0.007909 | 0.000208 | 0.000135 | 0 | 0.000234 | 0.001146 | 0 |
| d__Fungi;p__Ascomycota;c__Dothideomycetes;o__Capnodiales;f__Capnodiales_fam_Incertae_sedis | 0 | 0 | 0 | 0 | 0 | 0 | 0 | 0 | 0 | 0 | 0 | 0 |
| d__Fungi;p__Ascomycota;c__Dothideomycetes;o__Capnodiales;f__Mycosphaerellaceae | 0.000318 | 4.88E-05 | 0 | 0 | 0.000146 | 0 | 0.000167 | 0.000452 | 0.001402 | 5.86E-05 | 0.000985 | 0.000429 |
| d__Fungi;p__Ascomycota;c__Dothideomycetes;o__Capnodiales;f__Teratosphaeriaceae | 0.000491 | 0 | 0 | 0 | 4.87E-05 | 0 | 0 | 0.000565 | 0 | 0 | 0 | 0 |
| d__Fungi;p__Ascomycota;c__Dothideomycetes;o__Capnodiales;f__unclassified_Capnodiales | 0.000231 | 0 | 0 | 0 | 0 | 0 | 0 | 0 | 0 | 0 | 0 | 0 |
| d__Fungi;p__Ascomycota;c__Dothideomycetes;o__Capnodiales;f__unidentified | 0 | 0 | 0 | 0.000276 | 0 | 0 | 0 | 0 | 0 | 0 | 0.000503 | 0 |
| d__Fungi;p__Ascomycota;c__Dothideomycetes;o__Minutisphaerales;f__Minutisphaeraceae | 0 | 0 | 0 | 0 | 0 | 0 | 0 | 0 | 0 | 0 | 0 | 0 |
| d__Fungi;p__Ascomycota;c__Dothideomycetes;o__Mytilinidales;f__Gloniaceae | 0 | 0 | 0 | 0.000386 | 0 | 0 | 0 | 0 | 0 | 0 | 0 | 0 |
| d__Fungi;p__Ascomycota;c__Dothideomycetes;o__Pleosporales;f__Cucurbitariaceae | 5.78E-05 | 0 | 0.002739 | 3.68E-05 | 0 | 0 | 0.002831 | 0.00289 | 0.014159 | 0.002617 | 0.00963 | 0.003278 |
| d__Fungi;p__Ascomycota;c__Dothideomycetes;o__Pleosporales;f__Didymellaceae | 0.00052 | 0 | 0.002639 | 7.36E-05 | 0 | 0 | 0.000312 | 0.003206 | 0.004577 | 0.00164 | 0.005689 | 4.29E-05 |
| d__Fungi;p__Ascomycota;c__Dothideomycetes;o__Pleosporales;f__Didymosphaeriaceae | 0 | 0 | 0 | 0 | 0 | 0 | 0 | 0 | 0 | 0 | 0 | 0 |
| d__Fungi;p__Ascomycota;c__Dothideomycetes;o__Pleosporales;f__Leptosphaeriaceae | 0 | 0 | 0 | 0 | 0 | 0 | 8.33E-05 | 0.000587 | 0.000214 | 0.001016 | 0.000462 | 0 |
| d__Fungi;p__Ascomycota;c__Dothideomycetes;o__Pleosporales;f__Lophiostomataceae | 0 | 0 | 0.000465 | 0 | 0 | 0 | 0 | 0 | 0 | 0 | 0 | 0 |
| d__Fungi;p__Ascomycota;c__Dothideomycetes;o__Pleosporales;f__Lophiotremataceae | 0 | 0 | 0 | 0 | 0 | 0 | 0 | 0 | 0 | 0 | 0 | 0 |
| d__Fungi;p__Ascomycota;c__Dothideomycetes;o__Pleosporales;f__Melanommataceae | 0 | 0 | 0 | 0 | 0 | 0 | 0 | 0 | 0.000156 | 0 | 0.000342 | 0.009514 |
| d__Fungi;p__Ascomycota;c__Dothideomycetes;o__Pleosporales;f__Morosphaeriaceae | 0 | 0 | 0 | 0 | 0 | 0 | 0 | 0 | 0 | 0 | 0 | 0 |
| d__Fungi;p__Ascomycota;c__Dothideomycetes;o__Pleosporales;f__Periconiaceae | 0 | 0 | 0 | 0 | 0 | 0 | 0 | 0 | 0 | 0 | 0 | 0 |
| d__Fungi;p__Ascomycota;c__Dothideomycetes;o__Pleosporales;f__Phaeosphaeriaceae | 0 | 3.25E-05 | 0 | 0 | 0 | 0 | 0.000479 | 0.000948 | 0.006914 | 0 | 0.002111 | 0 |
| d__Fungi;p__Ascomycota;c__Dothideomycetes;o__Pleosporales;f__Pleosporaceae | 0 | 0 | 0.001909 | 0 | 3.24E-05 | 9.61E-05 | 0.012928 | 0.000903 | 0.00705 | 0.001308 | 6.03E-05 | 0.00015 |
| d__Fungi;p__Ascomycota;c__Dothideomycetes;o__Pleosporales;f__Pleosporales_fam_Incertae_sedis | 0 | 0 | 0 | 0 | 0 | 0 | 0 | 0 | 0 | 0 | 0 | 0 |
| d__Fungi;p__Ascomycota;c__Dothideomycetes;o__Pleosporales;f__Sporormiaceae | 0.000289 | 0 | 0 | 0 | 0 | 0 | 0.000146 | 0 | 0.001441 | 0.004882 | 0 | 0 |
| d__Fungi;p__Ascomycota;c__Dothideomycetes;o__Pleosporales;f__unclassified_Pleosporales | 0 | 0 | 0 | 0 | 3.24E-05 | 0 | 0.000291 | 0 | 0.004187 | 0.001152 | 0.000523 | 0 |
| d__Fungi;p__Ascomycota;c__Dothideomycetes;o__Pleosporales;f__unidentified | 0 | 0 | 0 | 9.20E-05 | 9.73E-05 | 0 | 0.031852 | 0.009981 | 0 | 0.000605 | 0 | 0 |
| d__Fungi;p__Ascomycota;c__Dothideomycetes;o__Tubeufiales;f__Tubeufiaceae | 0 | 0 | 0 | 0 | 0 | 0 | 0 | 0.000406 | 0 | 0.000352 | 0.000362 | 0 |
| d__Fungi;p__Ascomycota;c__Dothideomycetes;o__Venturiales;f__Sympoventuriaceae | 0 | 0 | 0 | 0 | 0 | 0 | 0 | 0.000158 | 0 | 0 | 0 | 0 |
| d__Fungi;p__Ascomycota;c__Dothideomycetes;o__Venturiales;f__Venturiaceae | 0 | 0 | 0.000564 | 0 | 0 | 0 | 0.000229 | 0.000835 | 0 | 0 | 0.026477 | 0.000279 |
| d__Fungi;p__Ascomycota;c__Dothideomycetes;o__Venturiales;f__unidentified | 0 | 0 | 0 | 0 | 0 | 0 | 0 | 0 | 0 | 0 | 0.000121 | 0 |
| d__Fungi;p__Ascomycota;c__Dothideomycetes;o__unclassified_Dothideomycetes;f__unclassified_Dothideomycetes | 0 | 0 | 0 | 0 | 0 | 0 | 0.000978 | 0 | 0 | 0 | 0 | 0 |
| d__Fungi;p__Ascomycota;c__Eurotiomycetes;o__Chaetothyriales;f__Chaetothyriaceae | 0 | 0 | 0 | 0 | 0 | 0 | 0 | 0 | 0 | 0 | 0 | 0 |
| d__Fungi;p__Ascomycota;c__Eurotiomycetes;o__Chaetothyriales;f__Chaetothyriales_fam_Incertae_sedis | 0 | 0 | 0 | 0 | 0 | 0 | 0 | 0 | 0 | 0 | 0 | 0 |
| d__Fungi;p__Ascomycota;c__Eurotiomycetes;o__Chaetothyriales;f__Cyphellophoraceae | 0 | 0 | 0 | 0 | 0 | 0 | 0 | 0.000226 | 0 | 0 | 0 | 0 |
| d__Fungi;p__Ascomycota;c__Eurotiomycetes;o__Chaetothyriales;f__Herpotrichiellaceae | 0.001488 | 0.000195 | 0 | 0 | 0.000503 | 0 | 0.003664 | 0.000497 | 0.002551 | 0.001972 | 0.007579 | 0.002121 |
| d__Fungi;p__Ascomycota;c__Eurotiomycetes;o__Chaetothyriales;f__Trichomeriaceae | 0 | 0 | 0 | 0 | 0 | 0 | 0.001041 | 0 | 0 | 0.00084 | 0.0039 | 0 |
| d__Fungi;p__Ascomycota;c__Eurotiomycetes;o__Chaetothyriales;f__unclassified_Chaetothyriales | 0.000116 | 0 | 0 | 0 | 0 | 0 | 0.000375 | 0.000113 | 0.000701 | 9.76E-05 | 0.000161 | 0.001586 |
| d__Fungi;p__Ascomycota;c__Eurotiomycetes;o__Chaetothyriales;f__unidentified | 0.000217 | 0.00013 | 0 | 0 | 0 | 0 | 0.000208 | 0 | 0 | 0 | 0 | 0 |
| d__Fungi;p__Ascomycota;c__Eurotiomycetes;o__Eurotiales;f__Aspergillaceae | 0.00039 | 0.00013 | 0.007022 | 0.000184 | 0.000292 | 0 | 0.022151 | 0.025155 | 0.089123 | 0.074483 | 0.014555 | 0.015985 |
| d__Fungi;p__Ascomycota;c__Eurotiomycetes;o__Eurotiales;f__Elaphomycetaceae | 0 | 0.000406 | 0 | 7.36E-05 | 0 | 0.000112 | 0 | 0 | 0 | 0 | 0.000101 | 0 |
| d__Fungi;p__Ascomycota;c__Eurotiomycetes;o__Eurotiales;f__Thermoascaceae | 7.22E-05 | 0.000163 | 0.000714 | 0 | 0 | 0 | 0 | 0 | 5.84E-05 | 0 | 0 | 0 |
| d__Fungi;p__Ascomycota;c__Eurotiomycetes;o__Eurotiales;f__Trichocomaceae | 0 | 0 | 0 | 0 | 0 | 0 | 4.16E-05 | 0 | 0.000799 | 0 | 0.000302 | 0.000514 |
| d__Fungi;p__Ascomycota;c__Eurotiomycetes;o__Eurotiales;f__unclassified_Eurotiales | 0 | 0 | 0.005495 | 0 | 0 | 0 | 0 | 0 | 0 | 0 | 0 | 0 |
| d__Fungi;p__Ascomycota;c__Eurotiomycetes;o__Onygenales;f__Gymnoascaceae | 0 | 0 | 0 | 0 | 0 | 0 | 0.001874 | 0 | 0 | 0 | 0 | 0 |
| d__Fungi;p__Ascomycota;c__Eurotiomycetes;o__Onygenales;f__Onygenales_fam_Incertae_sedis | 0 | 0 | 0 | 0 | 0 | 0 | 0.000146 | 0 | 0 | 0.002285 | 0.001166 | 0 |
| d__Fungi;p__Ascomycota;c__GS35;o__GS35;f__unidentified | 0 | 0 | 0 | 0 | 0 | 0.000144 | 0 | 0 | 0 | 0 | 0 | 0 |
| d__Fungi;p__Ascomycota;c__GS37;o__GS37;f__unidentified | 0 | 0 | 0 | 0 | 0 | 0 | 0 | 0 | 0 | 0 | 0 | 0 |
| d__Fungi;p__Ascomycota;c__Geoglossomycetes;o__Geoglossales;f__Geoglossaceae | 0 | 0 | 0 | 0 | 0 | 0 | 0 | 0 | 0 | 0 | 0 | 0 |
| d__Fungi;p__Ascomycota;c__Laboulbeniomycetes;o__Pyxidiophorales;f__unidentified | 0 | 0 | 0 | 0 | 0 | 0 | 0 | 0 | 0 | 0 | 0 | 0 |
| d__Fungi;p__Ascomycota;c__Lecanoromycetes;o__GS36;f__unidentified | 0 | 0 | 0 | 0 | 0 | 0 | 0 | 0 | 0 | 0 | 0.000181 | 0 |
| d__Fungi;p__Ascomycota;c__Lecanoromycetes;o__Ostropales;f__Stictidaceae | 0 | 0 | 0 | 0 | 0 | 0 | 0 | 0 | 0 | 0 | 0 | 0 |
| d__Fungi;p__Ascomycota;c__Lecanoromycetes;o__unclassified_Lecanoromycetes;f__unclassified_Lecanoromycetes | 0 | 0 | 0 | 0 | 0 | 0 | 0 | 0 | 0 | 0 | 0 | 0 |
| d__Fungi;p__Ascomycota;c__Lecanoromycetes;o__unidentified;f__unidentified | 0 | 0 | 0 | 0 | 0 | 0 | 0 | 0 | 0 | 0 | 0 | 0 |
| d__Fungi;p__Ascomycota;c__Leotiomycetes;o__Erysiphales;f__Erysiphaceae | 0 | 0 | 0 | 0 | 0 | 0 | 4.16E-05 | 0 | 0 | 0 | 0 | 0 |
| d__Fungi;p__Ascomycota;c__Leotiomycetes;o__Helotiales;f__Dermateaceae | 0 | 0 | 0 | 0 | 0 | 0 | 0 | 0 | 0 | 0 | 0 | 0 |
| d__Fungi;p__Ascomycota;c__Leotiomycetes;o__Helotiales;f__Helotiaceae | 0.000809 | 0.001479 | 0.011222 | 0.000184 | 0.000438 | 0 | 0.000229 | 0.001061 | 7.79E-05 | 0.000488 | 0.001709 | 0.001907 |
| d__Fungi;p__Ascomycota;c__Leotiomycetes;o__Helotiales;f__Helotiales_fam_Incertae_sedis | 0.026546 | 0.001447 | 0.009379 | 7.36E-05 | 0.000503 | 0.000256 | 0.029437 | 0.042451 | 0.037784 | 0.084014 | 0.048189 | 0.043433 |
| d__Fungi;p__Ascomycota;c__Leotiomycetes;o__Helotiales;f__Hyaloscyphaceae | 0.007944 | 0.007966 | 0.00425 | 0.003257 | 0.006635 | 0.000112 | 0.00077 | 0.000158 | 0.000117 | 0.002207 | 0.000965 | 0 |
| d__Fungi;p__Ascomycota;c__Leotiomycetes;o__Helotiales;f__Leotiaceae | 0.003466 | 4.88E-05 | 0.016749 | 0 | 0.000114 | 0 | 0 | 9.03E-05 | 3.90E-05 | 0.000156 | 0.000101 | 0 |
| d__Fungi;p__Ascomycota;c__Leotiomycetes;o__Helotiales;f__Myxotrichaceae | 0 | 0 | 0 | 0.000386 | 0.000389 | 0 | 0.00025 | 0.000384 | 0 | 0 | 0.000965 | 0 |
| d__Fungi;p__Ascomycota;c__Leotiomycetes;o__Helotiales;f__Sclerotiniaceae | 0.000679 | 0 | 0 | 0 | 0 | 0 | 0 | 0 | 0 | 0 | 0.000523 | 0 |
| d__Fungi;p__Ascomycota;c__Leotiomycetes;o__Helotiales;f__Vibrisseaceae | 0 | 0 | 0.00239 | 0.002006 | 0 | 0 | 0 | 0 | 0.001071 | 0 | 0.004664 | 4.29E-05 |
| d__Fungi;p__Ascomycota;c__Leotiomycetes;o__Helotiales;f__unclassified_Helotiales | 0.012291 | 0.001577 | 0.092827 | 0.001509 | 0.002141 | 0 | 0.000104 | 0 | 0.00076 | 0.00041 | 0.000844 | 0 |
| d__Fungi;p__Ascomycota;c__Leotiomycetes;o__Helotiales;f__unidentified | 0.00377 | 0.002455 | 0 | 0.000313 | 0.000616 | 3.20E-05 | 0.002727 | 0.0021 | 0.003545 | 0.022712 | 0.00388 | 0.004071 |
| d__Fungi;p__Ascomycota;c__Leotiomycetes;o__Phacidiales;f__Phacidiaceae | 0 | 0 | 0 | 0 | 0 | 0 | 0 | 0 | 0 | 0 | 0.000181 | 0 |
| d__Fungi;p__Ascomycota;c__Leotiomycetes;o__Phacidiales;f__unidentified | 0 | 0 | 0 | 0 | 0 | 0 | 0 | 0 | 0 | 0 | 0 | 0 |
| d__Fungi;p__Ascomycota;c__Leotiomycetes;o__Thelebolales;f__Pseudeurotiaceae | 0.129681 | 0.004715 | 0.103966 | 0.000865 | 0.002725 | 8.01E-05 | 0.0183 | 0.012871 | 0.044814 | 0.01451 | 0.065659 | 0.134905 |
| d__Fungi;p__Ascomycota;c__Leotiomycetes;o__Thelebolales;f__unidentified | 0 | 0 | 0 | 0 | 0 | 0 | 0 | 0 | 0 | 0 | 0 | 0 |
| d__Fungi;p__Ascomycota;c__Leotiomycetes;o__unclassified_Leotiomycetes;f__unclassified_Leotiomycetes | 0.000303 | 0 | 0 | 3.68E-05 | 0.00047 | 0 | 0 | 0 | 0 | 0 | 0.000121 | 0 |
| d__Fungi;p__Ascomycota;c__Leotiomycetes;o__unidentified;f__unidentified | 0 | 0 | 0 | 0 | 0 | 0 | 0.000791 | 0 | 0 | 0 | 0 | 0 |
| d__Fungi;p__Ascomycota;c__Orbiliomycetes;o__Orbiliales;f__Orbiliaceae | 0 | 0 | 0 | 0 | 0 | 0 | 0.002061 | 0 | 0 | 0 | 0.000322 | 0 |
| d__Fungi;p__Ascomycota;c__Orbiliomycetes;o__unidentified;f__unidentified | 0 | 0 | 0.004465 | 0 | 0 | 0 | 0 | 0 | 0 | 0 | 0 | 0 |
| d__Fungi;p__Ascomycota;c__Pezizomycetes;o__Pezizales;f__Chorioactidaceae | 0 | 0 | 0 | 0 | 0 | 0 | 0 | 0 | 0 | 0 | 0 | 0 |
| d__Fungi;p__Ascomycota;c__Pezizomycetes;o__Pezizales;f__Discinaceae | 0 | 0 | 0 | 7.36E-05 | 0 | 0 | 0 | 0 | 0 | 0 | 0 | 0 |
| d__Fungi;p__Ascomycota;c__Pezizomycetes;o__Pezizales;f__Pezizaceae | 0 | 0 | 0.001394 | 0 | 0 | 0 | 0 | 0 | 0 | 0.000156 | 0.000342 | 0 |
| d__Fungi;p__Ascomycota;c__Pezizomycetes;o__Pezizales;f__Pyronemataceae | 0 | 0 | 0 | 0 | 0 | 0 | 0 | 0 | 3.90E-05 | 0 | 0 | 0 |
| d__Fungi;p__Ascomycota;c__Pezizomycetes;o__Pezizales;f__Tuberaceae | 0 | 0 | 0 | 0 | 0 | 0 | 0 | 0 | 0 | 0 | 0 | 0 |
| d__Fungi;p__Ascomycota;c__Pezizomycetes;o__Pezizales;f__unclassified_Pezizales | 0 | 0 | 0 | 0 | 0 | 0 | 0 | 0 | 0 | 0 | 0 | 0 |
| d__Fungi;p__Ascomycota;c__Saccharomycetes;o__Saccharomycetales;f__Debaryomycetaceae | 0.000116 | 0 | 0.000315 | 0 | 0 | 8.01E-05 | 0.000437 | 0.000384 | 7.79E-05 | 0 | 0.000161 | 0.000129 |
| d__Fungi;p__Ascomycota;c__Saccharomycetes;o__Saccharomycetales;f__Lipomycetaceae | 0 | 0 | 0 | 0 | 0 | 0 | 0 | 0 | 0 | 0 | 0 | 0 |
| d__Fungi;p__Ascomycota;c__Saccharomycetes;o__Saccharomycetales;f__Pichiaceae | 0.001271 | 0.001301 | 0.002656 | 0.000736 | 0.000941 | 0.001041 | 0.002415 | 0.001603 | 0.002668 | 0.001621 | 0.002131 | 0.001543 |
| d__Fungi;p__Ascomycota;c__Saccharomycetes;o__Saccharomycetales;f__Saccharomycetaceae | 0 | 0 | 0 | 0 | 0 | 0 | 0 | 0 | 0 | 0 | 0 | 0 |
| d__Fungi;p__Ascomycota;c__Saccharomycetes;o__Saccharomycetales;f__Saccharomycetales_fam_Incertae_sedis | 0 | 0 | 0 | 5.52E-05 | 0 | 0 | 0 | 9.03E-05 | 0 | 0 | 0 | 0 |
| d__Fungi;p__Ascomycota;c__Saccharomycetes;o__Saccharomycetales;f__Trichomonascaceae | 0 | 0 | 0 | 0 | 0 | 0 | 0 | 0 | 0 | 0 | 0 | 0 |
| d__Fungi;p__Ascomycota;c__Saccharomycetes;o__Saccharomycetales;f__unclassified_Saccharomycetales | 0 | 0 | 0 | 3.68E-05 | 0 | 0 | 0 | 0 | 0 | 0 | 0 | 0 |
| d__Fungi;p__Ascomycota;c__Saccharomycetes;o__Saccharomycetales;f__unidentified | 0 | 0 | 0 | 0 | 0 | 0 | 0 | 0 | 0 | 0 | 0 | 0 |
| d__Fungi;p__Ascomycota;c__Saccharomycetes;o__unidentified;f__unidentified | 0 | 0 | 0 | 0 | 0.000438 | 0.000208 | 0 | 0 | 0 | 0 | 0 | 0 |
| d__Fungi;p__Ascomycota;c__Sordariomycetes;o__Chaetosphaeriales;f__Chaetosphaeriaceae | 0.001618 | 0 | 0 | 3.68E-05 | 0.000308 | 0 | 0.000146 | 0.00061 | 0 | 0.001836 | 0 | 0 |
| d__Fungi;p__Ascomycota;c__Sordariomycetes;o__Coniochaetales;f__Coniochaetaceae | 0 | 4.88E-05 | 0.000847 | 0 | 0 | 0 | 0.001312 | 0.001061 | 0 | 0 | 0.00396 | 0.002443 |
| d__Fungi;p__Ascomycota;c__Sordariomycetes;o__Diaporthales;f__Diaporthaceae | 0 | 0 | 0 | 0 | 3.24E-05 | 0 | 0 | 0 | 0 | 0 | 0 | 0 |
| d__Fungi;p__Ascomycota;c__Sordariomycetes;o__Diaporthales;f__Gnomoniaceae | 0.000202 | 0 | 0 | 0 | 0 | 0 | 0 | 0 | 0 | 0 | 0 | 0 |
| d__Fungi;p__Ascomycota;c__Sordariomycetes;o__Diaporthales;f__Valsaceae | 0 | 0 | 0 | 0 | 0 | 0 | 0 | 0 | 0 | 0.000195 | 0 | 0 |
| d__Fungi;p__Ascomycota;c__Sordariomycetes;o__Diaporthales;f__unclassified_Diaporthales | 0 | 0 | 0 | 0 | 0 | 0 | 0 | 0 | 0 | 0 | 6.03E-05 | 0 |
| d__Fungi;p__Ascomycota;c__Sordariomycetes;o__Glomerellales;f__Glomerellaceae | 0 | 8.13E-05 | 0 | 0 | 0 | 0 | 0 | 0 | 0 | 0 | 0.000161 | 0 |
| d__Fungi;p__Ascomycota;c__Sordariomycetes;o__Glomerellales;f__Plectosphaerellaceae | 0.000101 | 4.88E-05 | 0 | 0.000202 | 0.000162 | 0 | 0.000167 | 0 | 0.003856 | 0.001055 | 0.0039 | 0.000193 |
| d__Fungi;p__Ascomycota;c__Sordariomycetes;o__Hypocreales;f__Bionectriaceae | 0 | 0 | 0 | 0 | 0 | 0 | 0.000416 | 0.001445 | 0.00187 | 0.001094 | 0.000462 | 0.00105 |
| d__Fungi;p__Ascomycota;c__Sordariomycetes;o__Hypocreales;f__Clavicipitaceae | 0.004651 | 0 | 0 | 0 | 0 | 0 | 0.000208 | 0.000655 | 0.00296 | 0.000234 | 0.023743 | 0.022606 |
| d__Fungi;p__Ascomycota;c__Sordariomycetes;o__Hypocreales;f__Cordycipitaceae | 0.000765 | 0.00026 | 0 | 7.36E-05 | 0.003034 | 0 | 6.25E-05 | 0.000542 | 0.001052 | 0.000234 | 0.000201 | 0 |
| d__Fungi;p__Ascomycota;c__Sordariomycetes;o__Hypocreales;f__Hypocreaceae | 4.33E-05 | 0.000114 | 0 | 0.000239 | 6.49E-05 | 0 | 0.139838 | 0.362982 | 0.320654 | 0.152248 | 0.100338 | 0.135933 |
| d__Fungi;p__Ascomycota;c__Sordariomycetes;o__Hypocreales;f__Hypocreales_fam_Incertae_sedis | 0.000751 | 0 | 0 | 3.68E-05 | 4.87E-05 | 0 | 0.000812 | 0.000135 | 0.005531 | 0.002051 | 0.008906 | 0.000836 |
| d__Fungi;p__Ascomycota;c__Sordariomycetes;o__Hypocreales;f__Nectriaceae | 0.000202 | 0.000927 | 0.006076 | 0 | 0.000487 | 0 | 0.030603 | 0.018426 | 0.031921 | 0.115924 | 0.074464 | 0.025284 |
| d__Fungi;p__Ascomycota;c__Sordariomycetes;o__Hypocreales;f__Ophiocordycipitaceae | 0.001112 | 0 | 0 | 0 | 0.000227 | 0 | 0.000146 | 0 | 0 | 3.91E-05 | 0 | 0 |
| d__Fungi;p__Ascomycota;c__Sordariomycetes;o__Hypocreales;f__Stachybotryaceae | 0 | 0 | 0 | 0 | 0 | 0 | 0 | 0 | 0.000954 | 0.000566 | 0.002392 | 4.29E-05 |
| d__Fungi;p__Ascomycota;c__Sordariomycetes;o__Hypocreales;f__unclassified_Hypocreales | 0 | 0 | 0 | 0 | 0 | 0 | 0.000187 | 0 | 0 | 0.000176 | 0.002232 | 0.003171 |
| d__Fungi;p__Ascomycota;c__Sordariomycetes;o__Hypocreales;f__unidentified | 0.000722 | 3.25E-05 | 3.32E-05 | 0 | 0 | 0 | 0.000312 | 0 | 0.000292 | 0.000898 | 0.00197 | 0 |
| d__Fungi;p__Ascomycota;c__Sordariomycetes;o__Lulworthiales;f__Lulworthiaceae | 0 | 0 | 0 | 0 | 0 | 0 | 0 | 0.000226 | 0 | 0 | 0 | 0 |
| d__Fungi;p__Ascomycota;c__Sordariomycetes;o__Magnaporthales;f__Magnaporthaceae | 0 | 0 | 0 | 0 | 0 | 0 | 0.001978 | 0 | 0 | 0 | 0 | 0.00045 |
| d__Fungi;p__Ascomycota;c__Sordariomycetes;o__Microascales;f__Microascaceae | 0.000173 | 9.75E-05 | 0 | 0 | 9.73E-05 | 0 | 0 | 0 | 0 | 0 | 0.000141 | 0 |
| d__Fungi;p__Ascomycota;c__Sordariomycetes;o__Microascales;f__Microascales_fam_Incertae_sedis | 0 | 0 | 0 | 0 | 0 | 0 | 0 | 0 | 0 | 0 | 0 | 0 |
| d__Fungi;p__Ascomycota;c__Sordariomycetes;o__Microascales;f__unidentified | 0 | 0 | 0 | 0 | 0 | 0 | 0 | 4.52E-05 | 0 | 0 | 0 | 0 |
| d__Fungi;p__Ascomycota;c__Sordariomycetes;o__Myrmecridiales;f__Myrmecridiaceae | 0 | 0 | 0.001328 | 0 | 0 | 0 | 0.000375 | 0.001761 | 0 | 0 | 0.002654 | 0 |
| d__Fungi;p__Ascomycota;c__Sordariomycetes;o__Myrmecridiales;f__unclassified_Myrmecridiales | 0 | 0 | 0 | 0 | 0 | 0 | 0 | 0 | 0 | 0 | 0 | 0 |
| d__Fungi;p__Ascomycota;c__Sordariomycetes;o__Myrmecridiales;f__unidentified | 0 | 0 | 0 | 0 | 0 | 0 | 0 | 0 | 0 | 0 | 0 | 0 |
| d__Fungi;p__Ascomycota;c__Sordariomycetes;o__Sordariales;f__Cephalothecaceae | 0 | 6.50E-05 | 0 | 0 | 0 | 0 | 0.000583 | 0.000632 | 0 | 0 | 0 | 0 |
| d__Fungi;p__Ascomycota;c__Sordariomycetes;o__Sordariales;f__Chaetomiaceae | 0.010355 | 0.001154 | 0.008267 | 0 | 0.000665 | 0 | 0.035204 | 0.088245 | 0.05985 | 0.06632 | 0.020667 | 0.02327 |
| d__Fungi;p__Ascomycota;c__Sordariomycetes;o__Sordariales;f__Helminthosphaeriaceae | 0.000231 | 0 | 0.000249 | 0 | 0 | 0 | 0 | 0.000271 | 0 | 0 | 0 | 0 |
| d__Fungi;p__Ascomycota;c__Sordariomycetes;o__Sordariales;f__Lasiosphaeriaceae | 0.001719 | 9.75E-05 | 0.001544 | 0 | 0 | 0 | 0.001312 | 0.003387 | 0.009115 | 0.001601 | 0.002433 | 0.00105 |
| d__Fungi;p__Ascomycota;c__Sordariomycetes;o__Sordariales;f__Sordariaceae | 0 | 0 | 0 | 0 | 0 | 0 | 0.001728 | 0.006548 | 0 | 0.002597 | 0 | 0 |
| d__Fungi;p__Ascomycota;c__Sordariomycetes;o__Sordariales;f__Sordariales_fam_Incertae_sedis | 0 | 0 | 0 | 0 | 0 | 0 | 0 | 9.03E-05 | 0 | 0 | 0 | 0 |
| d__Fungi;p__Ascomycota;c__Sordariomycetes;o__Sordariales;f__unclassified_Sordariales | 0 | 0 | 0.000697 | 0 | 0 | 0 | 0 | 0 | 0 | 0 | 0 | 0.001607 |
| d__Fungi;p__Ascomycota;c__Sordariomycetes;o__Sordariales;f__unidentified | 0 | 0 | 0 | 0 | 0 | 0 | 0 | 0 | 0 | 0 | 0.000523 | 0 |
| d__Fungi;p__Ascomycota;c__Sordariomycetes;o__Xylariales;f__Bartaliniaceae | 0 | 0 | 0 | 0 | 0 | 0 | 0 | 0 | 0 | 0 | 0 | 0 |
| d__Fungi;p__Ascomycota;c__Sordariomycetes;o__Xylariales;f__Hyponectriaceae | 0 | 0.000179 | 9.96E-05 | 0 | 0 | 0 | 0 | 0 | 0 | 0 | 0 | 0 |
| d__Fungi;p__Ascomycota;c__Sordariomycetes;o__Xylariales;f__Microdochiaceae | 0 | 0 | 0 | 0 | 0 | 0 | 0 | 0 | 0.00111 | 0 | 0 | 0 |
| d__Fungi;p__Ascomycota;c__Sordariomycetes;o__Xylariales;f__Sporocadaceae | 0 | 0 | 0 | 0 | 0 | 0 | 0 | 0 | 0 | 0 | 0 | 0 |
| d__Fungi;p__Ascomycota;c__Sordariomycetes;o__Xylariales;f__Xylariaceae | 0.001502 | 0 | 0.005926 | 0 | 0 | 0 | 0 | 0 | 0 | 0 | 0 | 0 |
| d__Fungi;p__Ascomycota;c__Sordariomycetes;o__Xylariales;f__Xylariales_fam_Incertae_sedis | 0 | 0 | 0.000996 | 0 | 0 | 0 | 8.33E-05 | 0 | 0 | 0 | 0.000101 | 0.000857 |
| d__Fungi;p__Ascomycota;c__Sordariomycetes;o__Xylariales;f__unclassified_Xylariales | 0.000116 | 0 | 0 | 0 | 3.24E-05 | 0 | 0.000708 | 0 | 0 | 0 | 0 | 0 |
| d__Fungi;p__Ascomycota;c__Sordariomycetes;o__Xylariales;f__unidentified | 0 | 0 | 0.001859 | 0 | 0 | 0 | 0 | 0 | 0 | 0.000215 | 0 | 0 |
| d__Fungi;p__Ascomycota;c__Sordariomycetes;o__unclassified_Sordariomycetes;f__unclassified_Sordariomycetes | 0.00013 | 8.13E-05 | 0 | 0.000202 | 0.000276 | 0 | 0.020881 | 0.04918 | 0.035894 | 0.008456 | 0.028427 | 0.008999 |
| d__Fungi;p__Ascomycota;c__Sordariomycetes;o__unidentified;f__unidentified | 0 | 0 | 0.001942 | 0 | 0 | 3.20E-05 | 0.003706 | 0.000429 | 0 | 0 | 0.000121 | 0 |
| d__Fungi;p__Ascomycota;c__Taphrinomycetes;o__Taphrinales;f__Taphrinaceae | 0 | 0 | 0 | 0 | 0 | 0 | 0 | 0 | 0 | 0 | 0 | 0 |
| d__Fungi;p__Ascomycota;c__Xylonomycetes;o__GS34;f__unidentified | 0 | 0 | 0 | 3.68E-05 | 0 | 0 | 0 | 0 | 0 | 0 | 0 | 0 |
| d__Fungi;p__Ascomycota;c__unclassified_Ascomycota;o__unclassified_Ascomycota;f__unclassified_Ascomycota | 0.001343 | 0 | 0.001311 | 0 | 0.000552 | 9.61E-05 | 0.000479 | 0 | 0.001772 | 0.011385 | 0.006654 | 0.000879 |
| d__Fungi;p__Ascomycota;c__unidentified;o__unidentified;f__unidentified | 0 | 0.012535 | 0 | 0 | 0 | 0 | 0 | 0 | 0 | 0 | 0 | 0 |
| d__Fungi;p__Basidiomycota;c__Agaricomycetes;o__Agaricales;f__Agaricaceae | 0.000361 | 0 | 0 | 0 | 0 | 0 | 0 | 0 | 0 | 0 | 0 | 0 |
| d__Fungi;p__Basidiomycota;c__Agaricomycetes;o__Agaricales;f__Amanitaceae | 0 | 0.001528 | 0.0083 | 0.000184 | 0.051084 | 0.948013 | 0.000146 | 0.00079 | 0 | 0.000508 | 0.000362 | 0 |
| d__Fungi;p__Basidiomycota;c__Agaricomycetes;o__Agaricales;f__Bolbitiaceae | 0 | 0 | 0 | 0 | 0 | 0 | 6.25E-05 | 0 | 0 | 0 | 0.000241 | 0 |
| d__Fungi;p__Basidiomycota;c__Agaricomycetes;o__Agaricales;f__Clavariaceae | 0.154378 | 0.003967 | 0.025647 | 0.001527 | 0.007478 | 0.000112 | 0 | 0 | 0 | 0 | 0.000342 | 0 |
| d__Fungi;p__Basidiomycota;c__Agaricomycetes;o__Agaricales;f__Cortinariaceae | 0.000823 | 0.000845 | 0 | 0.045419 | 0.447473 | 0.000224 | 4.16E-05 | 0.000135 | 0.000175 | 0.000195 | 0.000241 | 0 |
| d__Fungi;p__Basidiomycota;c__Agaricomycetes;o__Agaricales;f__Entolomataceae | 0.001964 | 0.001707 | 0.001062 | 0.000129 | 0.008225 | 0 | 0.000146 | 0.001558 | 0.000234 | 0.000508 | 0 | 0.001736 |
| d__Fungi;p__Basidiomycota;c__Agaricomycetes;o__Agaricales;f__Hebelomataceae | 0 | 0 | 0 | 0 | 0 | 0 | 0 | 0 | 0 | 0 | 0 | 0 |
| d__Fungi;p__Basidiomycota;c__Agaricomycetes;o__Agaricales;f__Hydnangiaceae | 0 | 0.000325 | 0.000581 | 0 | 0.014762 | 3.20E-05 | 0 | 0 | 0 | 0 | 0 | 0 |
| d__Fungi;p__Basidiomycota;c__Agaricomycetes;o__Agaricales;f__Hygrophoraceae | 0.00026 | 0.00026 | 0.077804 | 0.00011 | 0.00047 | 0.000432 | 4.16E-05 | 0 | 3.90E-05 | 0 | 0.000241 | 0 |
| d__Fungi;p__Basidiomycota;c__Agaricomycetes;o__Agaricales;f__Hymenogastraceae | 0 | 0 | 0 | 0 | 0 | 0 | 0 | 0 | 0 | 0 | 0.000281 | 0 |
| d__Fungi;p__Basidiomycota;c__Agaricomycetes;o__Agaricales;f__Inocybaceae | 0.00013 | 0.022273 | 0.000249 | 0.064705 | 8.11E-05 | 0 | 0 | 0 | 0 | 0 | 0.000663 | 0 |
| d__Fungi;p__Basidiomycota;c__Agaricomycetes;o__Agaricales;f__Lycoperdaceae | 0.000303 | 0 | 0 | 0 | 0 | 0 | 0 | 0 | 0 | 0 | 0 | 0 |
| d__Fungi;p__Basidiomycota;c__Agaricomycetes;o__Agaricales;f__Lyophyllaceae | 0 | 0 | 0 | 0 | 0 | 0 | 0 | 0 | 0 | 0 | 0 | 0 |
| d__Fungi;p__Basidiomycota;c__Agaricomycetes;o__Agaricales;f__Physalacriaceae | 0 | 0 | 0 | 0 | 0 | 0 | 0.000916 | 0.000226 | 0 | 0 | 0.000804 | 0.00225 |
| d__Fungi;p__Basidiomycota;c__Agaricomycetes;o__Agaricales;f__Pluteaceae | 0.007351 | 0 | 0 | 0 | 0 | 0 | 0 | 0 | 0 | 0 | 0 | 0 |
| d__Fungi;p__Basidiomycota;c__Agaricomycetes;o__Agaricales;f__Psathyrellaceae | 0 | 0 | 0 | 0 | 0 | 0 | 0 | 0 | 0 | 0 | 0 | 0 |
| d__Fungi;p__Basidiomycota;c__Agaricomycetes;o__Agaricales;f__Strophariaceae | 0.093892 | 0.001414 | 0.260935 | 0.001067 | 0.001249 | 0 | 0 | 0 | 3.90E-05 | 0 | 0.000121 | 0 |
| d__Fungi;p__Basidiomycota;c__Agaricomycetes;o__Agaricales;f__Tricholomataceae | 0.000116 | 0 | 0.004515 | 0.000184 | 0 | 0 | 6.25E-05 | 0 | 0 | 0 | 0.00973 | 0.008464 |
| d__Fungi;p__Basidiomycota;c__Agaricomycetes;o__Agaricales;f__unclassified_Agaricales | 0.002123 | 0.000163 | 0.003303 | 0 | 0.00013 | 0.000304 | 0 | 4.52E-05 | 0.000234 | 0 | 4.02E-05 | 0 |
| d__Fungi;p__Basidiomycota;c__Agaricomycetes;o__Agaricales;f__unidentified | 0.000361 | 0 | 0 | 0 | 0 | 0 | 0.001603 | 0.004335 | 0 | 0 | 0.000141 | 0 |
| d__Fungi;p__Basidiomycota;c__Agaricomycetes;o__Atheliales;f__Atheliaceae | 4.33E-05 | 0.056967 | 0.00083 | 0.014557 | 0.018834 | 0.002402 | 0.005912 | 0 | 0 | 0.00125 | 0.000885 | 0.001478 |
| d__Fungi;p__Basidiomycota;c__Agaricomycetes;o__Auriculariales;f__Auriculariaceae | 0 | 0 | 0 | 0 | 0 | 0 | 0.21897 | 0 | 0 | 0 | 0 | 0 |
| d__Fungi;p__Basidiomycota;c__Agaricomycetes;o__Auriculariales;f__Exidiaceae | 0.079059 | 6.50E-05 | 6.64E-05 | 0 | 0 | 0.00032 | 0 | 0 | 0 | 0 | 0 | 0 |
| d__Fungi;p__Basidiomycota;c__Agaricomycetes;o__Auriculariales;f__Hyaloriaceae | 0.000578 | 0 | 0 | 0 | 0 | 0 | 0 | 0 | 0 | 0 | 0 | 0 |
| d__Fungi;p__Basidiomycota;c__Agaricomycetes;o__Auriculariales;f__unclassified_Auriculariales | 0 | 0 | 0 | 0 | 0 | 0 | 0.0005 | 0 | 0 | 0 | 0.012022 | 0 |
| d__Fungi;p__Basidiomycota;c__Agaricomycetes;o__Auriculariales;f__unidentified | 0 | 0 | 0 | 0 | 0 | 0 | 0 | 0 | 0 | 0 | 0.000462 | 0 |
| d__Fungi;p__Basidiomycota;c__Agaricomycetes;o__Boletales;f__Boletaceae | 0 | 0 | 0 | 0 | 0.001119 | 0 | 0 | 0 | 0 | 0 | 0 | 0 |
| d__Fungi;p__Basidiomycota;c__Agaricomycetes;o__Boletales;f__Boletales_fam_Incertae_sedis | 0 | 0 | 0 | 0 | 0 | 0.003266 | 0 | 0 | 0 | 0 | 0 | 0 |
| d__Fungi;p__Basidiomycota;c__Agaricomycetes;o__Boletales;f__Paxillaceae | 0 | 0 | 0 | 0 | 0.000146 | 0 | 0 | 0 | 0 | 0 | 0 | 0 |
| d__Fungi;p__Basidiomycota;c__Agaricomycetes;o__Boletales;f__Rhizopogonaceae | 0 | 0 | 0 | 0 | 0 | 0 | 0 | 0 | 0 | 0 | 0 | 0 |
| d__Fungi;p__Basidiomycota;c__Agaricomycetes;o__Boletales;f__Sclerodermataceae | 0 | 0 | 0 | 0.003478 | 0 | 0 | 0 | 0 | 0 | 0 | 0.000141 | 0 |
| d__Fungi;p__Basidiomycota;c__Agaricomycetes;o__Boletales;f__Suillaceae | 0.000376 | 0 | 0 | 0 | 0 | 0 | 0.000104 | 0 | 0 | 0.001504 | 0.000322 | 0 |
| d__Fungi;p__Basidiomycota;c__Agaricomycetes;o__Cantharellales;f__Botryobasidiaceae | 0 | 0 | 0 | 0 | 0 | 0 | 0 | 0 | 0 | 0 | 0 | 0 |
| d__Fungi;p__Basidiomycota;c__Agaricomycetes;o__Cantharellales;f__Cantharellales_fam_Incertae_sedis | 0 | 0 | 0 | 0 | 0 | 0 | 0 | 0 | 5.84E-05 | 0 | 0 | 0 |
| d__Fungi;p__Basidiomycota;c__Agaricomycetes;o__Cantharellales;f__Ceratobasidiaceae | 0 | 0 | 0 | 0 | 3.24E-05 | 0 | 0 | 0.0035 | 0.010829 | 0.003652 | 0.001206 | 0 |
| d__Fungi;p__Basidiomycota;c__Agaricomycetes;o__Cantharellales;f__Clavulinaceae | 0 | 0.038125 | 0.000963 | 0.005318 | 0.015817 | 3.20E-05 | 0 | 0 | 0 | 0.000195 | 8.04E-05 | 0.000386 |
| d__Fungi;p__Basidiomycota;c__Agaricomycetes;o__Cantharellales;f__Hydnaceae | 0 | 0 | 0 | 0 | 0 | 0 | 0 | 0 | 0 | 0 | 0 | 0 |
| d__Fungi;p__Basidiomycota;c__Agaricomycetes;o__Cantharellales;f__Tulasnellaceae | 0 | 0 | 0 | 0 | 0 | 0 | 6.25E-05 | 0.000113 | 0 | 0 | 0 | 0 |
| d__Fungi;p__Basidiomycota;c__Agaricomycetes;o__Geastrales;f__Geastraceae | 0 | 0 | 0 | 0 | 0 | 0 | 0 | 0 | 0 | 0 | 0 | 0 |
| d__Fungi;p__Basidiomycota;c__Agaricomycetes;o__Geastrales;f__Sphaerobolaceae | 0 | 0 | 0 | 0 | 0 | 0 | 0.000229 | 0 | 0 | 0 | 0 | 0 |
| d__Fungi;p__Basidiomycota;c__Agaricomycetes;o__Geastrales;f__unclassified_Geastrales | 0 | 0 | 0 | 0 | 6.49E-05 | 0 | 0 | 0.001965 | 0.002824 | 0 | 0 | 0 |
| d__Fungi;p__Basidiomycota;c__Agaricomycetes;o__Gloeophyllales;f__Gloeophyllaceae | 0 | 0 | 0 | 0 | 0 | 0 | 0 | 0 | 0 | 0 | 0 | 0 |
| d__Fungi;p__Basidiomycota;c__Agaricomycetes;o__Gomphales;f__Gomphaceae | 0 | 0 | 0.004947 | 0 | 0 | 0 | 0 | 0 | 0 | 0 | 0 | 0 |
| d__Fungi;p__Basidiomycota;c__Agaricomycetes;o__Hymenochaetales;f__Hymenochaetales_fam_Incertae_sedis | 0.001242 | 0 | 0 | 0 | 0 | 0 | 0 | 0 | 0 | 0 | 0 | 0 |
| d__Fungi;p__Basidiomycota;c__Agaricomycetes;o__Hymenochaetales;f__Schizoporaceae | 0.017505 | 0.001675 | 0 | 0 | 0 | 0 | 0 | 0 | 0 | 0 | 0.000181 | 0 |
| d__Fungi;p__Basidiomycota;c__Agaricomycetes;o__Jaapiales;f__Jaapiaceae | 0 | 0 | 0 | 0 | 0 | 0 | 0 | 0 | 0 | 0 | 0 | 0 |
| d__Fungi;p__Basidiomycota;c__Agaricomycetes;o__Phallales;f__Phallaceae | 0 | 6.50E-05 | 0 | 0 | 0 | 0 | 0 | 0 | 0 | 0 | 0 | 0 |
| d__Fungi;p__Basidiomycota;c__Agaricomycetes;o__Polyporales;f__Fomitopsidaceae | 0 | 0.000406 | 0 | 0 | 0 | 0 | 0 | 0 | 0 | 0 | 0 | 0 |
| d__Fungi;p__Basidiomycota;c__Agaricomycetes;o__Polyporales;f__Ganodermataceae | 0.051604 | 0.003121 | 0.033482 | 0.00046 | 0.000795 | 0.000144 | 0.002561 | 0.000565 | 0.000584 | 3.91E-05 | 0.001166 | 0 |
| d__Fungi;p__Basidiomycota;c__Agaricomycetes;o__Polyporales;f__Hyphodermataceae | 0 | 0.000228 | 0 | 0 | 0 | 0 | 0 | 0 | 0 | 0 | 0 | 0 |
| d__Fungi;p__Basidiomycota;c__Agaricomycetes;o__Polyporales;f__Meripilaceae | 0 | 0 | 0 | 0 | 0 | 0 | 0 | 0 | 0 | 0 | 0.000302 | 0 |
| d__Fungi;p__Basidiomycota;c__Agaricomycetes;o__Polyporales;f__Meruliaceae | 0.000246 | 0 | 0 | 0 | 0 | 0 | 0 | 0 | 0 | 0 | 0 | 0 |
| d__Fungi;p__Basidiomycota;c__Agaricomycetes;o__Polyporales;f__Podoscyphaceae | 0 | 0 | 0 | 0 | 0 | 0 | 0 | 0 | 0 | 0 | 0 | 0 |
| d__Fungi;p__Basidiomycota;c__Agaricomycetes;o__Polyporales;f__Polyporaceae | 0 | 0 | 0 | 0 | 0 | 0 | 0 | 0 | 0 | 0 | 0 | 0 |
| d__Fungi;p__Basidiomycota;c__Agaricomycetes;o__Polyporales;f__Steccherinaceae | 0 | 0 | 0 | 0 | 0 | 0 | 0 | 0 | 0 | 0 | 0 | 0 |
| d__Fungi;p__Basidiomycota;c__Agaricomycetes;o__Polyporales;f__Xenasmataceae | 0 | 0 | 0 | 0.000202 | 0.000357 | 0 | 0 | 0 | 0 | 0 | 0 | 0 |
| d__Fungi;p__Basidiomycota;c__Agaricomycetes;o__Polyporales;f__unclassified_Polyporales | 0 | 0 | 0 | 0 | 0 | 0 | 0 | 0 | 0 | 0 | 0 | 0 |
| d__Fungi;p__Basidiomycota;c__Agaricomycetes;o__Russulales;f__Auriscalpiaceae | 0 | 0 | 0 | 0 | 0 | 0 | 0 | 0 | 0 | 0 | 0 | 0 |
| d__Fungi;p__Basidiomycota;c__Agaricomycetes;o__Russulales;f__Bondarzewiaceae | 0 | 0 | 0 | 0 | 4.87E-05 | 0 | 0 | 0 | 0 | 0 | 0 | 0 |
| d__Fungi;p__Basidiomycota;c__Agaricomycetes;o__Russulales;f__Lachnocladiaceae | 0 | 0 | 0 | 0 | 0 | 0 | 0 | 0 | 0 | 0 | 0 | 0 |
| d__Fungi;p__Basidiomycota;c__Agaricomycetes;o__Russulales;f__Peniophoraceae | 2.89E-05 | 0 | 0 | 0 | 0 | 0 | 0 | 0 | 0 | 0 | 0 | 0 |
| d__Fungi;p__Basidiomycota;c__Agaricomycetes;o__Russulales;f__Russulaceae | 0.004781 | 0.279032 | 0.00239 | 0.014005 | 0.263091 | 0.00032 | 0.000666 | 0.000361 | 0.000214 | 7.81E-05 | 0.002915 | 0.000107 |
| d__Fungi;p__Basidiomycota;c__Agaricomycetes;o__Russulales;f__Stereaceae | 0 | 0 | 0 | 0 | 0 | 0 | 0 | 0 | 0 | 0 | 0 | 0 |
| d__Fungi;p__Basidiomycota;c__Agaricomycetes;o__Russulales;f__unclassified_Russulales | 0 | 0 | 0 | 0 | 0 | 0 | 0.000708 | 0 | 0 | 0 | 0 | 0 |
| d__Fungi;p__Basidiomycota;c__Agaricomycetes;o__Sebacinales;f__Sebacinaceae | 0.000303 | 0.000341 | 0.000183 | 0.000386 | 0.003163 | 0.026114 | 0.000291 | 4.52E-05 | 3.90E-05 | 0.000605 | 0.000302 | 0 |
| d__Fungi;p__Basidiomycota;c__Agaricomycetes;o__Sebacinales;f__Serendipitaceae | 0 | 4.88E-05 | 0 | 0 | 0 | 0 | 0 | 0 | 0.00259 | 0 | 0 | 0 |
| d__Fungi;p__Basidiomycota;c__Agaricomycetes;o__Sebacinales;f__unclassified_Sebacinales | 0 | 0 | 0 | 0 | 0 | 0 | 0 | 0 | 0 | 0 | 0 | 0 |
| d__Fungi;p__Basidiomycota;c__Agaricomycetes;o__Sebacinales;f__unidentified | 0 | 0 | 0 | 0 | 0 | 0 | 0 | 0 | 0 | 0 | 0.001508 | 0 |
| d__Fungi;p__Basidiomycota;c__Agaricomycetes;o__Thelephorales;f__Thelephoraceae | 0.003943 | 0.399909 | 0.005063 | 0.020722 | 0.007122 | 0.005444 | 0.000416 | 0.000248 | 0.003311 | 0 | 0.001568 | 0.000621 |
| d__Fungi;p__Basidiomycota;c__Agaricomycetes;o__Trechisporales;f__Hydnodontaceae | 0.000505 | 0.000146 | 0.00259 | 0.001417 | 0.004104 | 0 | 0.059541 | 0.12058 | 0.007654 | 0.000976 | 0.062563 | 0 |
| d__Fungi;p__Basidiomycota;c__Agaricomycetes;o__Trechisporales;f__unidentified | 0 | 0.000455 | 0 | 0 | 0 | 0 | 0 | 0 | 0 | 0.008417 | 0 | 0 |
| d__Fungi;p__Basidiomycota;c__Agaricomycetes;o__unclassified_Agaricomycetes;f__unclassified_Agaricomycetes | 0.008102 | 0 | 0.001693 | 0.006404 | 0.000552 | 0 | 0.169547 | 0.001355 | 0 | 0.044643 | 0.005388 | 0 |
| d__Fungi;p__Basidiomycota;c__Agaricomycetes;o__unidentified;f__unidentified | 0 | 0.000894 | 0.027108 | 0.002172 | 0.000795 | 0 | 0.000146 | 0.000248 | 0 | 5.86E-05 | 0.005126 | 0 |
| d__Fungi;p__Basidiomycota;c__Cystobasidiomycetes;o__Cystobasidiales;f__Cystobasidiaceae | 0 | 0 | 0 | 0 | 0 | 0 | 0 | 0 | 0 | 0 | 0.000342 | 0 |
| d__Fungi;p__Basidiomycota;c__Geminibasidiomycetes;o__Geminibasidiales;f__Geminibasidiaceae | 0 | 0 | 0 | 0 | 0.00013 | 0 | 0 | 0 | 0 | 0 | 0 | 0 |
| d__Fungi;p__Basidiomycota;c__Malasseziomycetes;o__Malasseziales;f__Malasseziaceae | 0 | 0 | 0.002606 | 0 | 0 | 0 | 0 | 0 | 0.000428 | 0 | 0 | 0 |
| d__Fungi;p__Basidiomycota;c__Microbotryomycetes;o__Kriegeriales;f__Kriegeriaceae | 0 | 0 | 0 | 0 | 0 | 0 | 0 | 0 | 0 | 0 | 0.000161 | 0 |
| d__Fungi;p__Basidiomycota;c__Microbotryomycetes;o__Leucosporidiales;f__Leucosporidiaceae | 0.003813 | 0 | 0 | 0 | 0 | 0 | 0.000167 | 0.001377 | 0 | 0 | 0.000462 | 0 |
| d__Fungi;p__Basidiomycota;c__Microbotryomycetes;o__Leucosporidiales;f__unidentified | 0 | 0 | 0 | 0 | 0.001411 | 8.01E-05 | 0 | 0 | 0 | 0 | 0 | 0 |
| d__Fungi;p__Basidiomycota;c__Microbotryomycetes;o__Microbotryomycetes_ord_Incertae_sedis;f__Chrysozymaceae | 0 | 0 | 0 | 0.000221 | 4.87E-05 | 0 | 0 | 0 | 0 | 0 | 0 | 0 |
| d__Fungi;p__Basidiomycota;c__Microbotryomycetes;o__Microbotryomycetes_ord_Incertae_sedis;f__Microbotryomycetes_fam_Incertae_sedis | 0 | 0 | 0 | 0 | 0 | 0 | 0 | 0 | 0 | 0 | 0 | 0 |
| d__Fungi;p__Basidiomycota;c__Microbotryomycetes;o__Sporidiobolales;f__Sporidiobolaceae | 0 | 8.13E-05 | 0 | 0 | 0 | 0.000384 | 0 | 0 | 0 | 0 | 0 | 0 |
| d__Fungi;p__Basidiomycota;c__Microbotryomycetes;o__unclassified_Microbotryomycetes;f__unclassified_Microbotryomycetes | 0 | 0 | 0 | 0 | 0 | 0 | 0 | 0 | 0 | 0 | 0 | 0 |
| d__Fungi;p__Basidiomycota;c__Microbotryomycetes;o__unidentified;f__unidentified | 0 | 0 | 0 | 0 | 0 | 0 | 0 | 0 | 0 | 0 | 0.000121 | 0 |
| d__Fungi;p__Basidiomycota;c__Pucciniomycetes;o__Platygloeales;f__unidentified | 0 | 0 | 0 | 0 | 0 | 0 | 0 | 0 | 0 | 0 | 0 | 0 |
| d__Fungi;p__Basidiomycota;c__Pucciniomycetes;o__Pucciniales;f__Uropyxidaceae | 0 | 0 | 0 | 0 | 0 | 0 | 0 | 0 | 0 | 0 | 0 | 0 |
| d__Fungi;p__Basidiomycota;c__Pucciniomycetes;o__Pucciniales;f__unidentified | 0 | 0.000163 | 0 | 0 | 0 | 0 | 0 | 0 | 0 | 0 | 0 | 0 |
| d__Fungi;p__Basidiomycota;c__Tremellomycetes;o__Cystofilobasidiales;f__Cystofilobasidiales_fam_Incertae_sedis | 0 | 0 | 0 | 0 | 0 | 0 | 6.25E-05 | 0.000135 | 0 | 0 | 0 | 0 |
| d__Fungi;p__Basidiomycota;c__Tremellomycetes;o__Cystofilobasidiales;f__Mrakiaceae | 0 | 0 | 0.002424 | 0 | 0 | 0 | 0 | 0.000113 | 0 | 0.001094 | 0 | 0 |
| d__Fungi;p__Basidiomycota;c__Tremellomycetes;o__Filobasidiales;f__Filobasidiaceae | 0.000505 | 0 | 0.000548 | 0 | 0 | 0 | 0 | 0.001919 | 0.000292 | 0.002422 | 0.000623 | 0 |
| d__Fungi;p__Basidiomycota;c__Tremellomycetes;o__Filobasidiales;f__Piskurozymaceae | 0.001632 | 0.001317 | 0.001212 | 0.00057 | 0.001703 | 0.000144 | 0.012679 | 0.007677 | 0.047775 | 0.010917 | 0.020988 | 0.009214 |
| d__Fungi;p__Basidiomycota;c__Tremellomycetes;o__Holtermanniales;f__Holtermanniales_fam_Incertae_sedis | 0 | 0 | 0 | 0 | 0 | 0 | 0.000479 | 0.000429 | 0.00037 | 0.002988 | 0.006473 | 0 |
| d__Fungi;p__Basidiomycota;c__Tremellomycetes;o__Tremellales;f__Bulleraceae | 0 | 0 | 0 | 0 | 0 | 0 | 0 | 0 | 0 | 0 | 0 | 0 |
| d__Fungi;p__Basidiomycota;c__Tremellomycetes;o__Tremellales;f__Bulleribasidiaceae | 0 | 0 | 0 | 0 | 0 | 0 | 0 | 4.52E-05 | 0 | 0 | 0.000985 | 0 |
| d__Fungi;p__Basidiomycota;c__Tremellomycetes;o__Tremellales;f__Tremellaceae | 7.22E-05 | 0 | 0 | 0 | 0 | 0 | 0 | 0 | 0 | 0 | 0.001709 | 0 |
| d__Fungi;p__Basidiomycota;c__Tremellomycetes;o__Tremellales;f__Trimorphomycetaceae | 0.001733 | 0.001366 | 0.000166 | 0.00173 | 0.004542 | 0.001393 | 0.00304 | 0 | 0 | 0 | 0.006051 | 0 |
| d__Fungi;p__Basidiomycota;c__Tremellomycetes;o__Tremellales;f__unclassified_Tremellales | 0 | 0 | 0 | 0 | 0 | 0 | 0 | 0.000339 | 0 | 0 | 0.000704 | 0 |
| d__Fungi;p__Basidiomycota;c__Tremellomycetes;o__Tremellales;f__unidentified | 0 | 0 | 0 | 0 | 0 | 0 | 0 | 0 | 0 | 0 | 0 | 0 |
| d__Fungi;p__Basidiomycota;c__Tremellomycetes;o__Trichosporonales;f__Trichosporonaceae | 0 | 0 | 0 | 0 | 4.87E-05 | 0 | 0 | 0 | 0 | 0 | 0.000302 | 0 |
| d__Fungi;p__Basidiomycota;c__Tremellomycetes;o__unclassified_Tremellomycetes;f__unclassified_Tremellomycetes | 0 | 0 | 0 | 5.52E-05 | 0 | 0 | 0 | 0 | 0 | 0 | 8.04E-05 | 0 |
| d__Fungi;p__Basidiomycota;c__Ustilaginomycetes;o__Ustilaginales;f__Ustilaginaceae | 0 | 0 | 0 | 0 | 0 | 0 | 0 | 0 | 0 | 0 | 0 | 0 |
| d__Fungi;p__Basidiomycota;c__Wallemiomycetes;o__Wallemiales;f__Wallemiaceae | 0 | 0 | 0 | 0 | 0 | 0 | 0 | 0.000406 | 0 | 0 | 0 | 0 |
| d__Fungi;p__Basidiomycota;c__unclassified_Basidiomycota;o__unclassified_Basidiomycota;f__unclassified_Basidiomycota | 0.004102 | 0.000146 | 0.003851 | 0.00011 | 0.002563 | 0 | 0.000167 | 0 | 0 | 0 | 0.001166 | 0.000129 |
| d__Fungi;p__Blastocladiomycota;c__Blastocladiomycetes;o__GS15;f__unidentified | 0 | 0 | 0 | 0 | 0 | 0 | 0 | 0 | 0 | 0 | 0 | 0 |
| d__Fungi;p__Chytridiomycota;c__Chytridiomycetes;o__Chytridiales;f__Chytridiaceae | 0 | 0 | 0 | 0 | 0 | 0 | 0.000187 | 0.000135 | 0 | 0 | 0 | 0 |
| d__Fungi;p__Chytridiomycota;c__Rhizophlyctidomycetes;o__Rhizophlyctidales;f__Rhizophlyctidaceae | 0 | 0 | 0 | 0 | 0 | 0 | 0 | 0 | 0 | 0.007499 | 0 | 0 |
| d__Fungi;p__Chytridiomycota;c__Rhizophydiomycetes;o__Rhizophydiales;f__Terramycetaceae | 0.000289 | 0 | 0.000714 | 0 | 0 | 0 | 0 | 0 | 0 | 0 | 0 | 0 |
| d__Fungi;p__Chytridiomycota;c__unclassified_Chytridiomycota;o__unclassified_Chytridiomycota;f__unclassified_Chytridiomycota | 0 | 0 | 0 | 0 | 0 | 0 | 0 | 0 | 0 | 0 | 0 | 0 |
| d__Fungi;p__Chytridiomycota;c__unidentified;o__unidentified;f__unidentified | 0.003784 | 0 | 0.00083 | 0 | 6.49E-05 | 0 | 0 | 0 | 0 | 0 | 0 | 0.000343 |
| d__Fungi;p__GS01;c__unidentified;o__unidentified;f__unidentified | 0 | 0 | 0 | 0 | 0 | 0 | 0 | 0 | 0 | 0 | 0 | 0 |
| d__Fungi;p__Glomeromycota;c__Archaeosporomycetes;o__Archaeosporales;f__Ambisporaceae | 0 | 0 | 0 | 0 | 0 | 0 | 0 | 0 | 0 | 0 | 0.000181 | 0 |
| d__Fungi;p__Glomeromycota;c__Archaeosporomycetes;o__Archaeosporales;f__Archaeosporaceae | 8.67E-05 | 0 | 0 | 0 | 0 | 0 | 0 | 0 | 0 | 0 | 0 | 0 |
| d__Fungi;p__Glomeromycota;c__Archaeosporomycetes;o__Archaeosporales;f__unidentified | 0.000549 | 0.000829 | 0.00083 | 0 | 0 | 0 | 0 | 0 | 0 | 0 | 0 | 0.000236 |
| d__Fungi;p__Glomeromycota;c__Glomeromycetes;o__Diversisporales;f__unidentified | 0 | 0 | 0 | 0 | 0 | 0 | 0 | 0 | 0 | 0 | 0 | 0 |
| d__Fungi;p__Glomeromycota;c__Glomeromycetes;o__Glomerales;f__Glomeraceae | 0.00091 | 0 | 0 | 3.68E-05 | 0 | 0 | 0 | 0 | 0 | 0.000234 | 0 | 0 |
| d__Fungi;p__Glomeromycota;c__Paraglomeromycetes;o__Paraglomerales;f__Paraglomeraceae | 0 | 0 | 0 | 0 | 0 | 0 | 0 | 0.000294 | 0 | 0 | 0.000322 | 0.000707 |
| d__Fungi;p__Glomeromycota;c__Paraglomeromycetes;o__Paraglomerales;f__unclassified_Paraglomerales | 0 | 0 | 0 | 0 | 0 | 0 | 0.000333 | 0 | 0.000234 | 0 | 0 | 0.000214 |
| d__Fungi;p__Glomeromycota;c__unclassified_Glomeromycota;o__unclassified_Glomeromycota;f__unclassified_Glomeromycota | 0.002239 | 0 | 0.000747 | 0 | 0 | 0 | 0 | 0 | 0 | 0 | 0.000302 | 0.00135 |
| d__Fungi;p__Glomeromycota;c__unidentified;o__unidentified;f__unidentified | 5.78E-05 | 0 | 0 | 0 | 0 | 0 | 0 | 0 | 0 | 0 | 0 | 0 |
| d__Fungi;p__Kickxellomycota;c__GS19;o__GS19;f__unidentified | 0 | 0 | 0 | 0 | 0 | 0 | 0 | 0 | 0 | 0 | 0 | 0 |
| d__Fungi;p__Kickxellomycota;c__Kickxellomycetes;o__Kickxellales;f__Kickxellaceae | 0 | 0 | 0.000647 | 0 | 0 | 0 | 0 | 0 | 7.79E-05 | 0 | 0 | 0 |
| d__Fungi;p__Monoblepharomycota;c__Monoblepharidomycetes;o__unidentified;f__unidentified | 0 | 0 | 0 | 0 | 0 | 0 | 0 | 0 | 0 | 0 | 0 | 0 |
| d__Fungi;p__Monoblepharomycota;c__Sanchytriomycetes;o__Sanchytriales;f__Sanchytriaceae | 0 | 0 | 0 | 0 | 0 | 0 | 0 | 0 | 0 | 0 | 0 | 0 |
| d__Fungi;p__Mortierellomycota;c__Mortierellomycetes;o__Mortierellales;f__Mortierellaceae | 0.120915 | 0.032792 | 0.056506 | 0.001785 | 0.020651 | 0.00024 | 0.04578 | 0.06169 | 0.061564 | 0.155548 | 0.138434 | 0.250654 |
| d__Fungi;p__Mortierellomycota;c__Mortierellomycetes;o__Mortierellales;f__unidentified | 0 | 0 | 0 | 0 | 0.000114 | 0 | 0 | 0 | 0 | 0 | 0 | 0 |
| d__Fungi;p__Mucoromycota;c__Mucoromycetes;o__Mucorales;f__Lichtheimiaceae | 0 | 0 | 0.000282 | 0.000258 | 0 | 0 | 0 | 0 | 0 | 0.000234 | 0.000402 | 0.000129 |
| d__Fungi;p__Mucoromycota;c__Mucoromycetes;o__Mucorales;f__Mucoraceae | 0.000188 | 0 | 0 | 0 | 9.73E-05 | 0 | 0.000229 | 6.77E-05 | 0.000623 | 0.000195 | 0.000342 | 0.000536 |
| d__Fungi;p__Mucoromycota;c__Mucoromycetes;o__Mucorales;f__Rhizopodaceae | 0 | 0 | 0 | 0 | 0 | 0 | 0 | 0 | 0 | 0 | 0 | 0 |
| d__Fungi;p__Mucoromycota;c__Umbelopsidomycetes;o__Umbelopsidales;f__Umbelopsidaceae | 0.001069 | 0 | 0 | 0.000313 | 0.000552 | 0 | 0.001124 | 0.000519 | 0 | 0 | 0.000724 | 0 |
| d__Fungi;p__Olpidiomycota;c__Olpidiomycetes;o__Olpidiales;f__Olpidiaceae | 0 | 0 | 0.00073 | 0 | 0 | 0 | 0 | 0 | 0.001617 | 0 | 0 | 0 |
| d__Fungi;p__Rozellomycota;c__Rozellomycotina_cls_Incertae_sedis;o__GS02;f__unidentified | 0 | 0 | 0 | 0 | 0 | 0 | 0 | 0 | 0 | 0 | 0 | 0.005464 |
| d__Fungi;p__Rozellomycota;c__Rozellomycotina_cls_Incertae_sedis;o__GS04;f__unidentified | 0 | 0.001398 | 0 | 0 | 0 | 0 | 0 | 0.0007 | 0 | 0.000508 | 0 | 0 |
| d__Fungi;p__Rozellomycota;c__Rozellomycotina_cls_Incertae_sedis;o__GS05;f__unidentified | 0 | 0 | 0 | 0 | 0 | 0 | 0 | 0 | 0 | 0.001445 | 0 | 0 |
| d__Fungi;p__Rozellomycota;c__Rozellomycotina_cls_Incertae_sedis;o__GS09;f__unidentified | 0 | 0 | 0.000166 | 0 | 0.000146 | 0 | 0 | 0 | 0 | 0 | 0 | 0 |
| d__Fungi;p__Rozellomycota;c__Rozellomycotina_cls_Incertae_sedis;o__GS10;f__unidentified | 0 | 0 | 0 | 0 | 0 | 0 | 0 | 0 | 0 | 0 | 0.000905 | 0 |
| d__Fungi;p__Rozellomycota;c__Rozellomycotina_cls_Incertae_sedis;o__GS11;f__unidentified | 0 | 0 | 0 | 0 | 0 | 0 | 0 | 0 | 0 | 0 | 0 | 0 |
| d__Fungi;p__Rozellomycota;c__unidentified;o__unidentified;f__unidentified | 0.034735 | 0.001431 | 0.000232 | 0.001435 | 0.000406 | 0 | 0.000666 | 0.000474 | 0.000818 | 0.001836 | 0.007338 | 0 |
| d__Fungi;p__Zoopagomycota;c__Zoopagomycetes;o__Zoopagales;f__Piptocephalidaceae | 0 | 0 | 0 | 0 | 0 | 0 | 0 | 0 | 0 | 0.003086 | 0.001829 | 0.000214 |
| d__Fungi;p__unclassified_Fungi;c__unclassified_Fungi;o__unclassified_Fungi;f__unclassified_Fungi | 0.10419 | 0.005755 | 0.040321 | 0.00276 | 0.007462 | 0.0004 | 0.035621 | 0.065506 | 0.074632 | 0.08208 | 0.145149 | 0.147204 |
| d__Fungi;p__unidentified;c__unidentified;o__unidentified;f__unidentified | 0.049033 | 0.012307 | 0.116731 | 0.000331 | 0.001574 | 0 | 0.05798 | 0.07718 | 0.081585 | 0.080342 | 0.062945 | 0.11997 |
