# Supplementary material for: Shifts in rhizosphere microbial communities in Oplopanax elatus Nakai are related to soil chemical properties under different growth conditions
Source: Sci Rep. 2022 Jul 7;12:11485. doi: 10.1038/s41598-022-15340-1 (PMC9262954; doi:10.1038/s41598-022-15340-1)
Supplement: Supplementary file 2 — Supplementary Information 2. [file 41598_2022_15340_MOESM2_ESM.pptx]

## Slide 1
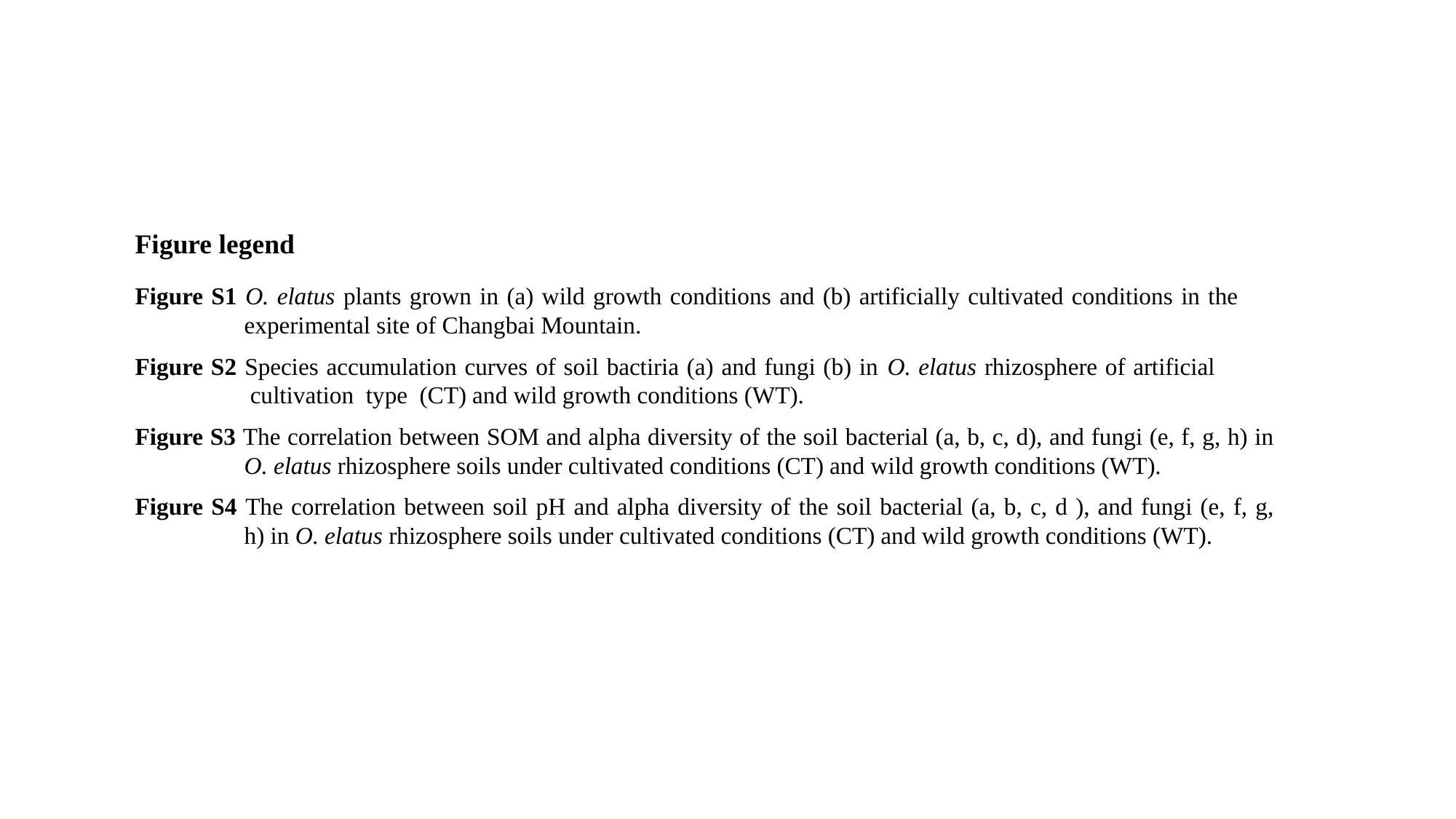

Figure legend
Figure S1 O. elatus plants grown in (a) wild growth conditions and (b) artificially cultivated conditions in the 	 	experimental site of Changbai Mountain.
Figure S2 Species accumulation curves of soil bactiria (a) and fungi (b) in O. elatus rhizosphere of artificial 	 	 cultivation type (CT) and wild growth conditions (WT).
Figure S3 The correlation between SOM and alpha diversity of the soil bacterial (a, b, c, d), and fungi (e, f, g, h) in 	O. elatus rhizosphere soils under cultivated conditions (CT) and wild growth conditions (WT).
Figure S4 The correlation between soil pH and alpha diversity of the soil bacterial (a, b, c, d ), and fungi (e, f, g, 	h) in O. elatus rhizosphere soils under cultivated conditions (CT) and wild growth conditions (WT).

## Slide 2
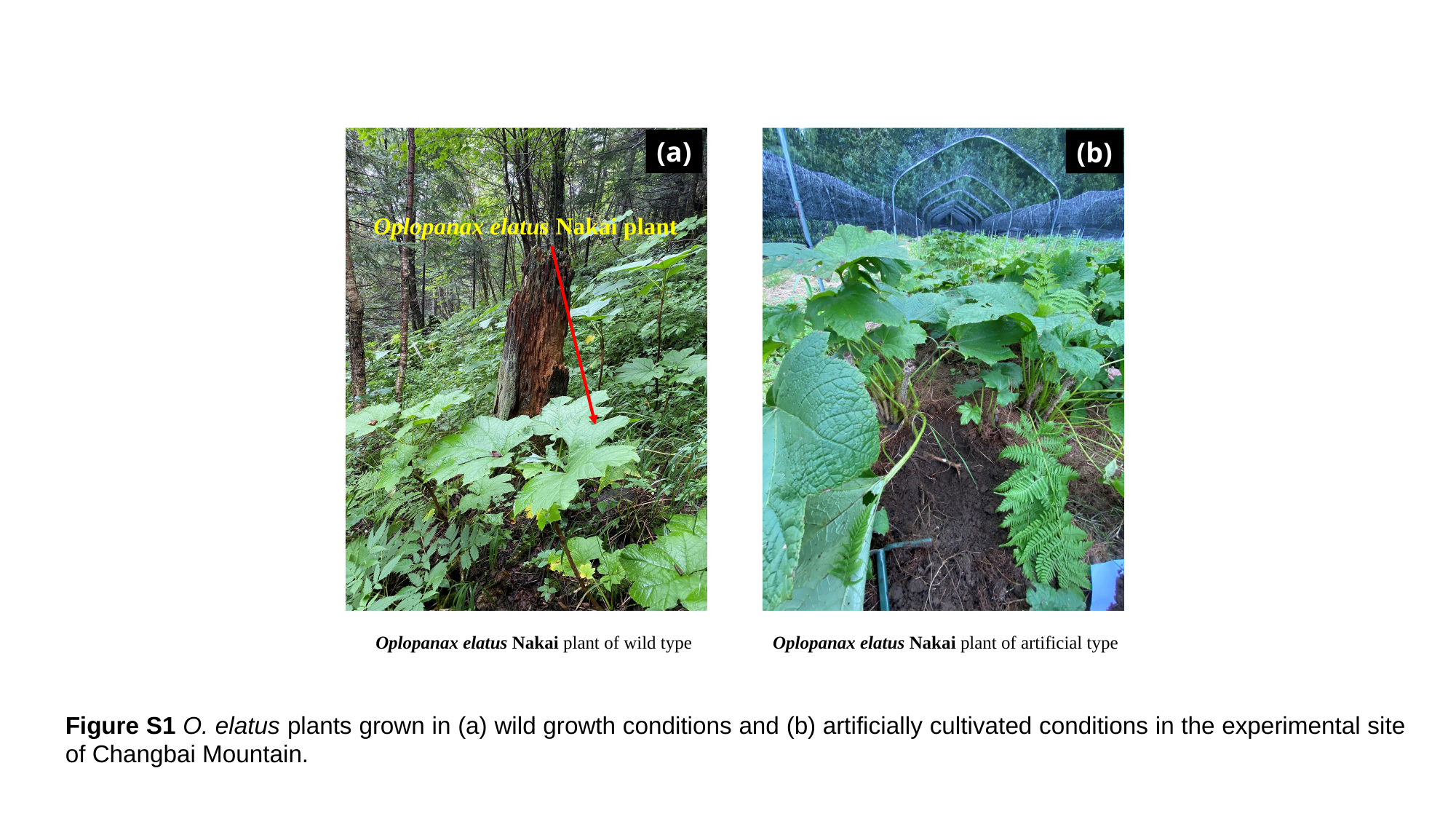

(a)
Oplopanax elatus Nakai plant of wild type
(b)
Oplopanax elatus Nakai plant of artificial type
Oplopanax elatus Nakai plant
Oplopanax elatus Nakai plant
Figure S1 O. elatus plants grown in (a) wild growth conditions and (b) artificially cultivated conditions in the experimental site of Changbai Mountain.

## Slide 3
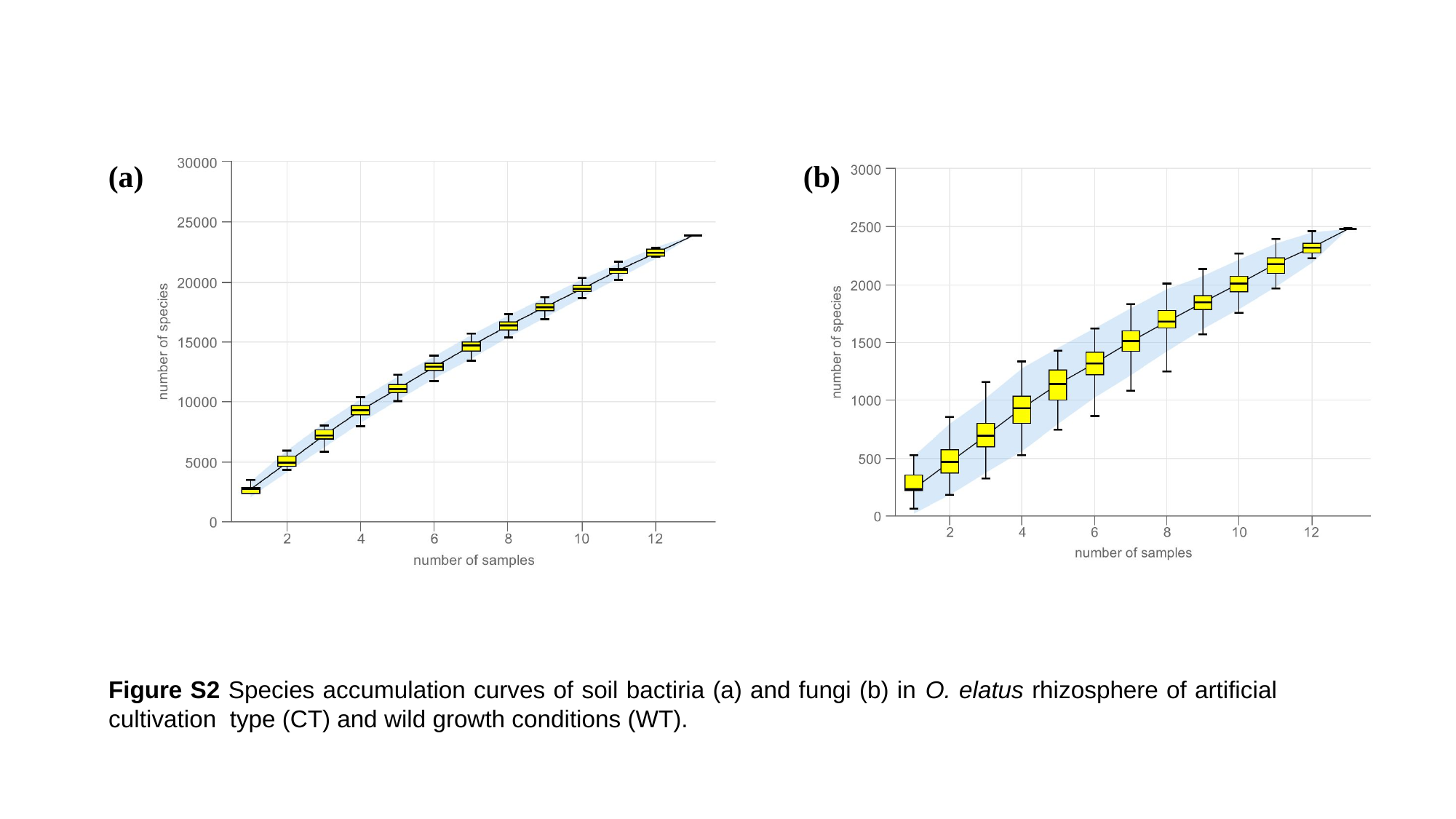

(a)
(b)
Figure S2 Species accumulation curves of soil bactiria (a) and fungi (b) in O. elatus rhizosphere of artificial cultivation type (CT) and wild growth conditions (WT).

## Slide 4
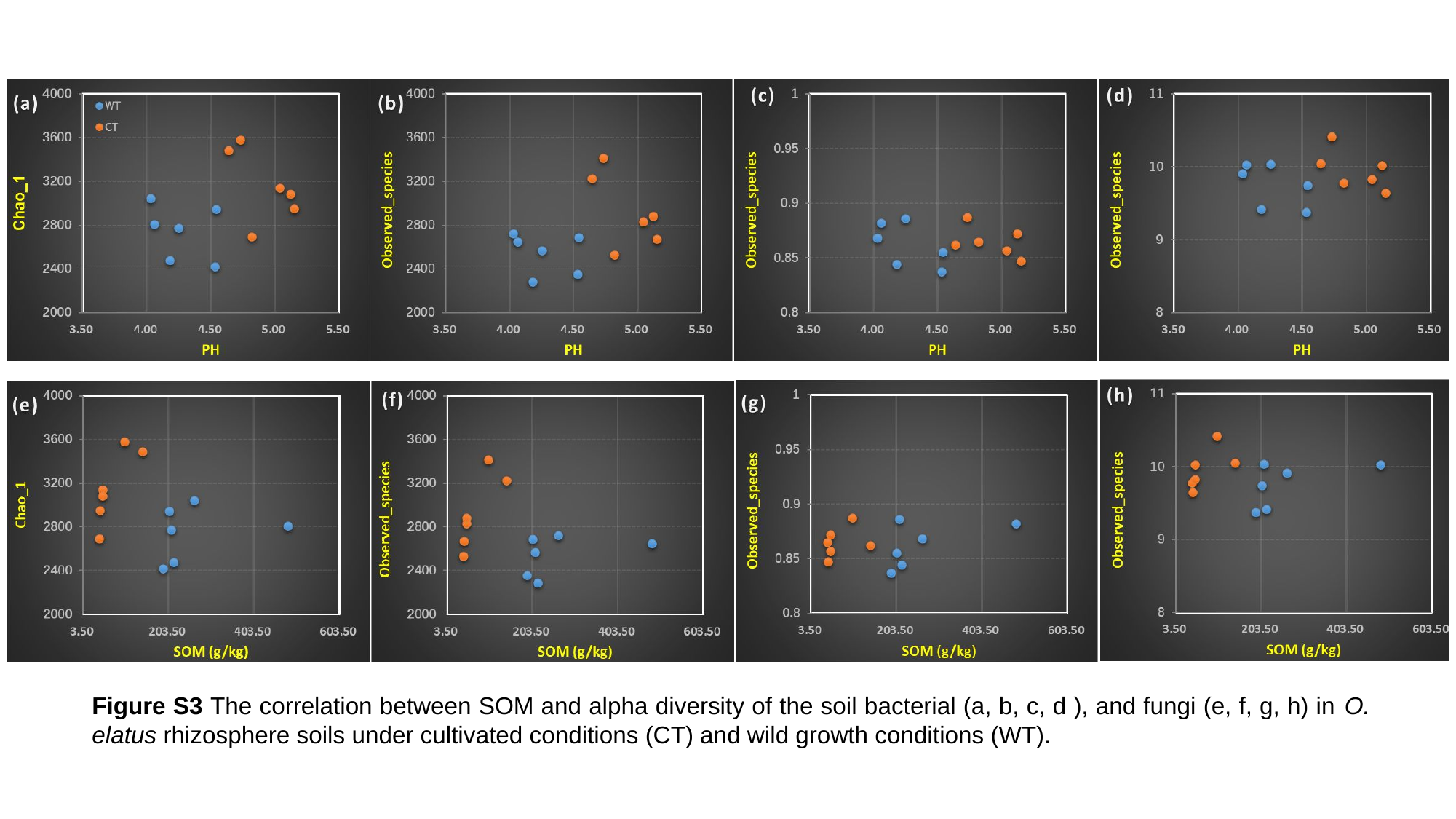

Figure S3 The correlation between SOM and alpha diversity of the soil bacterial (a, b, c, d ), and fungi (e, f, g, h) in O. elatus rhizosphere soils under cultivated conditions (CT) and wild growth conditions (WT).

## Slide 5
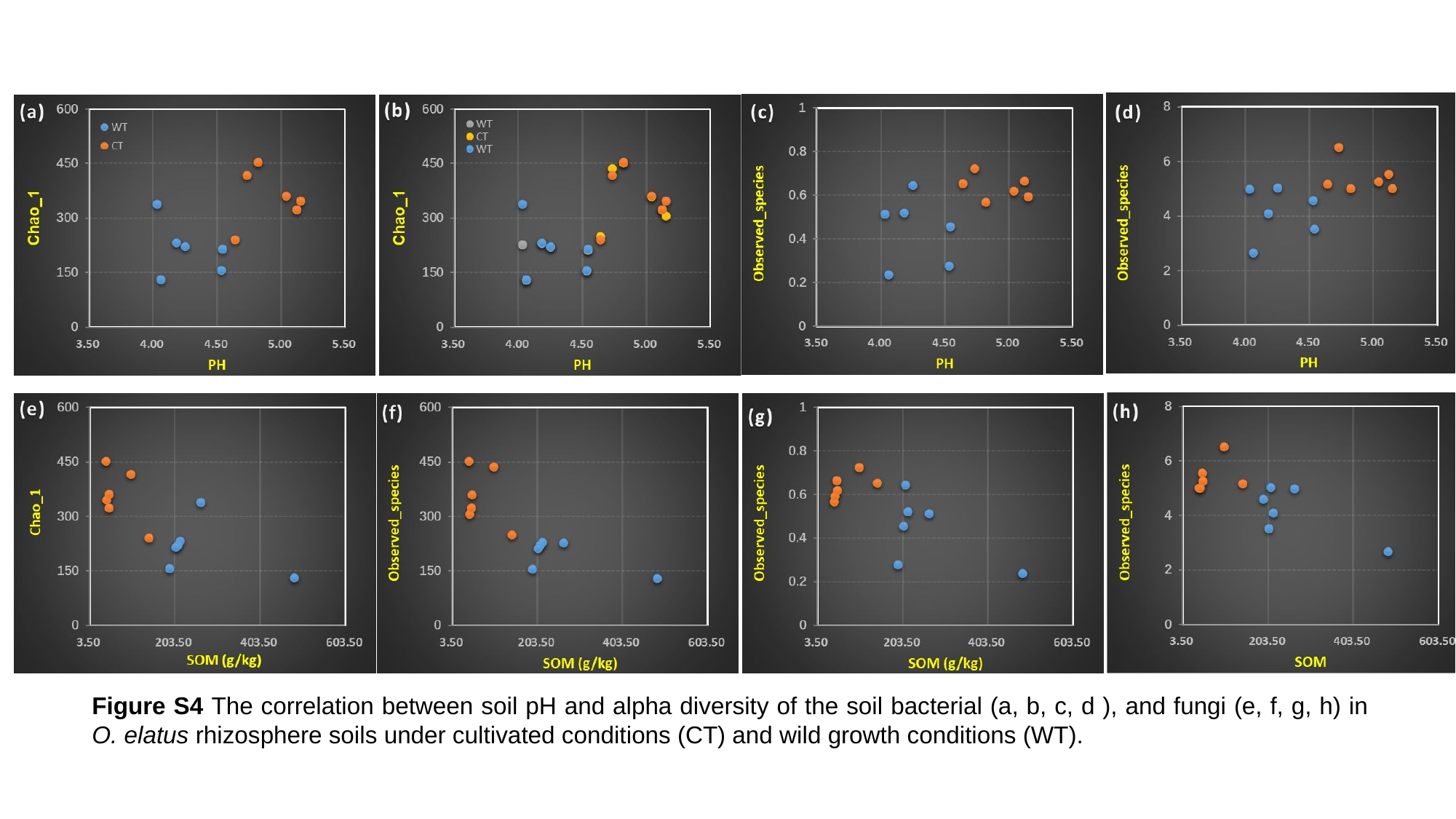

Figure S4 The correlation between soil pH and alpha diversity of the soil bacterial (a, b, c, d ), and fungi (e, f, g, h) in O. elatus rhizosphere soils under cultivated conditions (CT) and wild growth conditions (WT).
